# De Novo Synthesis of Dihydrobenzofurans and Indolines and Its Application to a Modular, Asymmetric Synthesis of Beraprost

**DOI:** 10.1021/jacs.3c04582

**Published:** 2023-06-16

**Authors:** Ze-Shu Wang, Steven H. Bennett, Bilal Kicin, Changcheng Jing, Johan A. Pradeilles, Karen Thai, James R. Smith, P. David Bacoş, Valerio Fasano, Carla M. Saunders, Varinder K. Aggarwal

**Affiliations:** School of Chemistry, University of Bristol, Cantock’s Close, Bristol BS8 1TS, U.K.

## Abstract

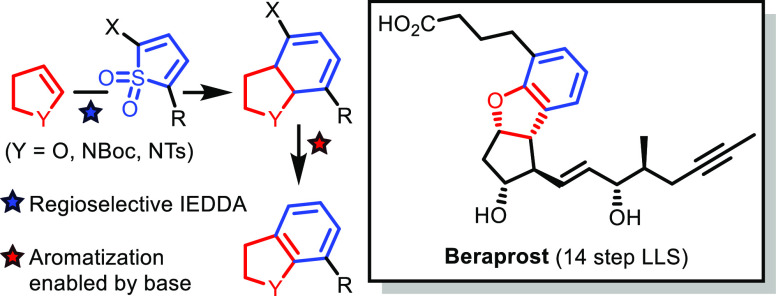

Dihydrobenzofurans and indolines are important constituents of pharmaceuticals. Herein, we describe a novel strategy for their construction in which the aromatic ring is created *de novo* through an inverse-electron demand Diels–Alder reaction and cheletropic extrusion sequence of a 2-halothiophene-1,1-dioxide with an enol ether/enamide, followed by aromatization. Unusually, the aromatization process proved to be highly challenging, but it was discovered that treatment of the halocyclohexadienes with a base effected an α-elimination–aromatization reaction. Mechanistic investigation of this step using deuterium-labeling studies indicated the intermediacy of a carbene which undergoes a 1,2-hydrogen shift and subsequent aromatization. The methodology was applied to a modular and stereoselective total synthesis of the antiplatelet drug beraprost in only 8 steps from a key enal-lactone. This lactone provided the core of beraprost to which both its sidechains could be appended through a 1,4-conjugate addition process (lower ω-sidechain), followed by *de novo* construction of beraprost’s dihydrobenzofuran (upper α-sidechain) using our newly developed methodology. Additionally, we have demonstrated the breadth of our newly established protocol in the synthesis of functionalized indolines, which occurred with high levels of regiocontrol. According to density-functional theory (DFT) calculations, the high selectivity originates from attractive London dispersion interactions in the TS of the Diels–Alder reaction.

## Introduction

**Figure 1:**
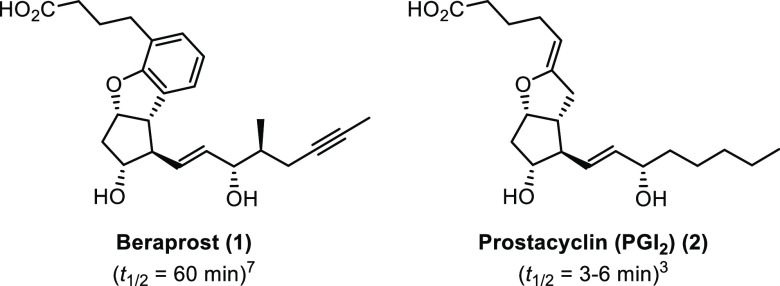
Structural comparison of beraprost (**1**) vs prostacyclin (PGI_2_, **2**).

Pulmonary arterial hypertension (PAH) is a rare and incurable cardiovascular disease (CVD) that is characterized by high blood pressure in the arteries of the lungs, which ultimately leads to right ventricular heart failure and premature death.^1^ Typical treatment for this life-threatening illness involves the administration of the potent anti-hypertensive, prostacyclin (PGI_2_, epoprostenol, **2**).^2^ Unfortunately, due to the short metabolic half-life of PGI_2_ in blood (*t*_1/2_ = 3–6 min)^3^ continuous intravenous (IV) infusion is required.^2^ To address the practical issues associated with such treatment, a number of more stable PGI_2_ analogues have been developed, e.g., cicaprost,^4^ iloprost^5^ and more recently, beraprost (**1**)^6^ (Figure 1).

Beraprost (beraprost sodium, **1**)^7^ is a more stable (*t*_1/2_ = 60 min) and less cytotoxic prostacyclin analogue and can be administered orally. It has been used successfully in the treatment of peripheral arterial disease (Buerger’s disease and arteriosclerosis obliterans) and shows promise as a potential therapeutic for the treatment of PAH due to its ability to drastically reduce pulmonary arterial pressure and resistance.^7^

Most strategies for the enantioselective synthesis of beraprost have focused on using either resolution strategies,^8^ or the enantiopure (−)-Corey lactone.^9^ Recently, Hayashi,^10^ reported an asymmetric organocatalytic synthesis of beraprost through a formal [3+2] cycloaddition reaction catalyzed by diphenylprolinol silyl ether as the key step to construct the cyclopentane core.^11^ We have previously reported enantioselective syntheses of several prostanoids from enal-lactone **7** (or the related hemi-acetal), which is prepared by a stereocontrolled organocatalytic dimerization of succinaldehyde in 3 steps with >99% e.e.^12^ We were keen to broaden the reach of this chemistry further and now report its use in a 14 step [longest linear sequence (LLS)] synthesis of beraprost. Furthermore, during the course of the synthesis, we discovered a conceptually novel route to access dihydrobenzofurans, which we demonstrate is more general and can also be applied to the *de novo* synthesis of indolines.

### Retrosynthetic Analysis

**Scheme 1:**
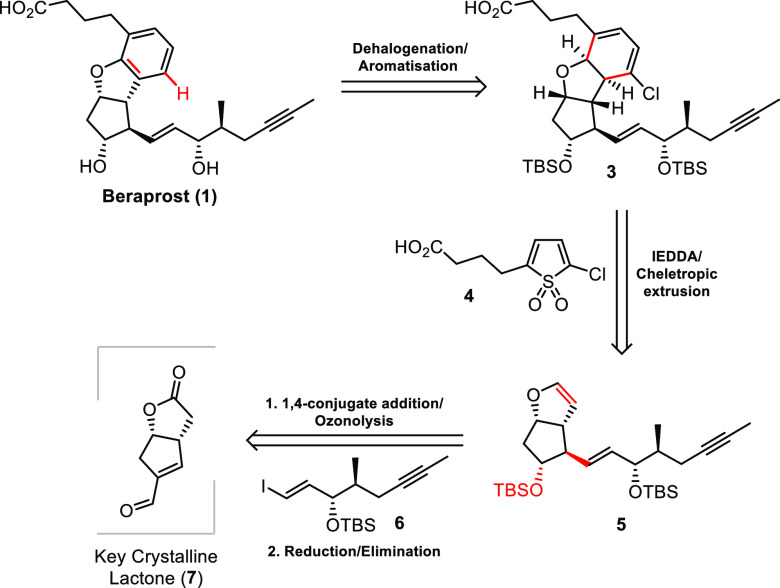
Proposed Modular Retrosynthetic Analysis of Beraprost

In terms of retrosynthesis, we envisaged a modular approach involving three key building blocks: enal-lactone **7**, upper α-sidechain **4**, and lower ω-sidechain **6**. The upper α-sidechain was proposed to be installed through an inverse-electron demand Diels–Alder reaction (IEDDA) between enol ether **5** and thiophene-1,1-dioxide **4**, followed by cheletropic extrusion of SO_2_. Subsequent dehalogenation and aromatization would furnish beraprost’s dihydrobenzofuran core. The lower ω-sidechain could be introduced through a 1,4-conjugate addition of vinyl iodide **6** and key enal-lactone **7** (Scheme 1). A critical issue that we were uncertain about initially was the regiochemistry of the IEDDA process, as most Diels–Alder reactions of thiophene 1,1-dioxides use symmetrical substrates.^13a−13d^ Additionally, monosubstituted thiophene 1,1-dioxides are known to be unstable and prone to rapid cyclodimerization.^13e,13f^ We therefore decided to introduce a halogen substituent in the 2-position to stabilize the thiophene 1,1-dioxide, which, in addition, would also lower the LUMO of the diene for the IEDDA.^14^ Such a substituent would also be easily removable at a later stage.

## Results and Discussion

### Asymmetric Synthesis of Beraprost

**Scheme 2:**
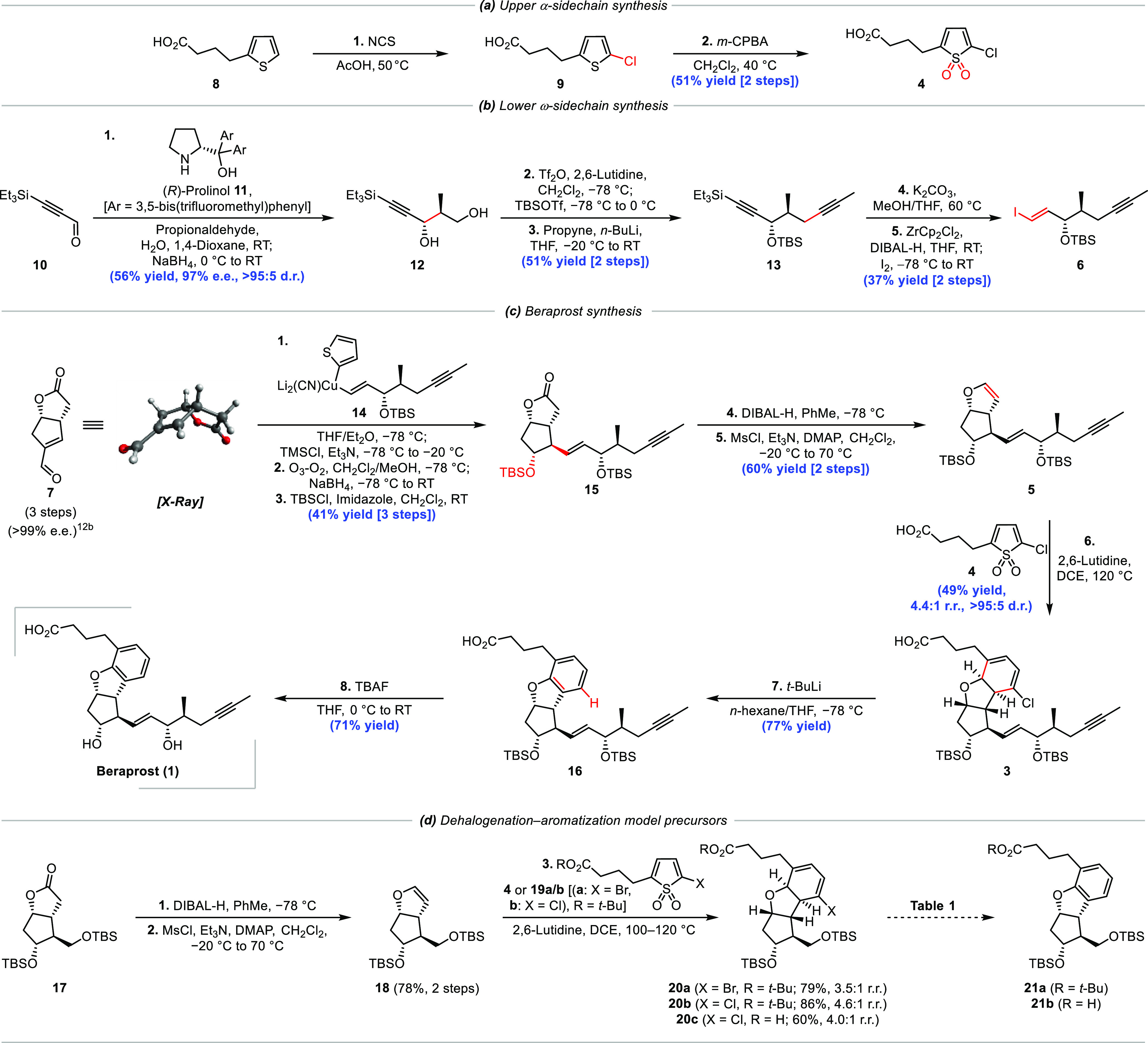
Modular Asymmetric Synthesis of Beraprost

Starting from commercially available thiophene **8**, we prepared thiophene 1,1-dioxide **4** in two steps following a regioselective chlorination using *N*-chlorosuccinimide (NCS) and subsequent oxidation with *meta*-chloroperoxybenzoic acid (*m*-CPBA) (Scheme 2a). This afforded the desired upper α-sidechain in 51% yield on a gram scale. The lower ω-sidechain (Scheme 2b) required for 1,4-conjugate addition was prepared from ynal **10**,^15^ which itself could be synthesized on a decagram scale from (triethylsilyl)acetylene. Ynal **10** readily underwent a prolinol-catalyzed aldol reaction with propionaldehyde,^16^ followed by *in situ* reduction with NaBH_4_ to give diol **12** with near-perfect enantioselectivity (98.5:1.5 e.r.) and high diastereoselectivity (9.1:1 d.r.) that was improved to >95:5 d.r. following flash column chromatography. The primary alcohol of diol **12** was then selectively transformed into a triflate and the remaining secondary alcohol was protected as its corresponding *tert*-butylsilyl (TBS) ether. This allowed for the selective substitution of the triflate with propynyllithium to afford di-alkyne **13** in 51% over two steps. Subsequent alkyne deprotection under basic conditions followed by hydrozirconation/iodination of the resultant terminal alkyne produced vinyl iodide **6** in 37% over two steps.

Following gram-scale synthesis of enal-lactone **7**,^12b^ and with vinyl iodide **6** in hand, the 1,4-conjugate addition of *in situ*-generated higher-order cyanocuprate **14** was performed (Scheme 2c). Following 1,4-addition onto the enal of **7** and trapping with trimethylsilyl chloride (TMSCl), a TMS enol ether was produced, which was perfectly set up to undergo selective ozonolysis of the more electron-rich alkene. Subsequent reductive work-up with NaBH_4_ and TBSCl protection delivered protected secondary alcohol **15** in 41% yield over 3 steps as a single diastereomer. The high stereocontrol observed in this sequence is a consequence of the convex shape of enal-lactone **7**, which favors attack from the more exposed *exo* face (see the X-ray structure). The lactone moiety of **15** was reduced with DIBAL-H to give a hemiacetal, which, following mesylation and subsequent heating under reflux, underwent elimination to produce enol ether **5** in 60% yield over 2 steps. Heating enol ether **5** in the presence of thiophene 1,1-dioxide **4** and 2,6-lutidine effected the desired IEDDA/cheletropic extrusion process giving chlorocyclohexadiene **3** in 49% yield and 4.4:1 regioisomeric ratio (r.r.). We believe the selectivity is controlled by steric repulsion between the two long linear sidechains rather than by any possible electronic effects (*vide infra*). Indeed, the more hindered *t*-Bu ester of thiophene 1,1-dioxide **4** gave the corresponding chlorocyclohexadiene with an improved 5.2:1 r.r. The success of the IEDDA reaction provided vindication of our strategy since we were only a few steps from beraprost. However, the seemingly facile oxidation of the diene to the aromatic ring, which can often occur spontaneously in air,^17^ was unexpectedly found to be the most challenging step in the synthesis.

### Dehalogenation–Aromatization of the Model System

To further explore and optimize our dehalogenation–aromatization step, we used the simple model compounds **20a–c**, prepared from cycloaddition of (−)-Corey lactone derived enol ether **18** with various thiophene-1,1-dioxides in moderate regioselectivity (**20a**, 3.5:1 r.r.; **20b**, 4.6:1 r.r.; **20c**, 4.0:1 r.r.) (Scheme 2d). We initially tested the bromo-diene and *tert*-butyl protected ester form (**20a**), which we believed would be easier to guide our initial efforts toward aromatization. At first, we focused on the use of palladium or platinum catalysis to effect simultaneous aromatization and dehalogenation (a redox neutral process), but unfortunately, we observed decomposition under the majority of conditions.^9,18^ No aromatization was observed under a number of other conditions: DBU/air,^19^ DDQ,^20^ KMnO_4_·Al_2_O_3_,^21^ MnO_2_,^22^ and PdCl_2_/HSi(OEt)_3_.^23^ In fact, in many cases, aromatization by elimination of the alcohol occurred rather than oxidation.^24^ Indeed, a survey of the literature revealed that similar *cis*-fused 5,6-cyclohexadiene ring systems often show remarkable resistance toward aromatization, requiring either novel strategies^25a^ or harsh conditions^25b,25c^ to effect the transformation. Furthermore, our system incorporated an additional steric challenge with the ring-junction hydrogens presenting into the concave face of the molecule, making their removal more difficult. At this point, we wanted to determine if the halogen (bromide) was somehow inhibiting aromatization and as such sought the bromide-free cyclohexadiene. Thus, bromocyclohexadiene **20a** was subjected to a lithium–halogen exchange reaction using 2.0 equiv of *tert*-butyllithium (*t*-BuLi) in THF at −78 °C, followed by quenching with MeOH. However, surprisingly, the expected lithium–halogen exchange product was not observed. Instead, the highly sought-after aromatic product **21a** was found in 7% yield alongside the *tert*-butyl ketone derivative **22**, formed by over-reaction of the excess *t*-BuLi (Table 1, entry 1).

**Table 1:**
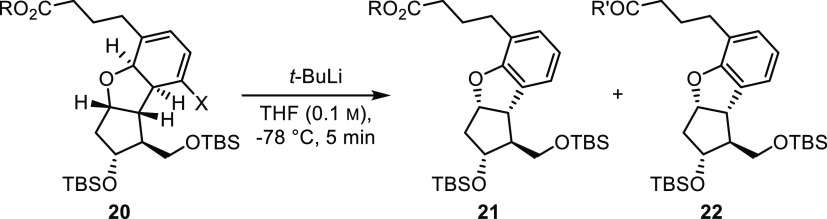
Optimization of the Dehalogenation–Aromatization Model Reaction^a^

			yield (%)^b^		
entry	substrate	*t-*BuLi (equiv)	**20**	**21**	**22**
1	**20a** (R = R’ = *t*-Bu, X = Br)	2.0	29	7	2
2^c^	**20a** (R = R′ = *t*-Bu, X = Br)	2.0	21	21	8
3^c^^,^^d^	**20a** (R = R′ = *t*-Bu, X = Br)	3.0	23	33	13
4^c^^,^^d^	**20b** (R = R′ = *t*-Bu, X = Cl)	3.0	0	56	32
5^c^^,^^d^	**20c** (R = H, R′ = *t*-Bu, X = Cl)	4.0	20	38	N/A
6^c^	**20c (R = H, R**′ **= *t*-Bu, X = Cl)**	**4.0**	***trace***	**66**^e^	N/A
7^c^	**20c** (R = H, R′ = *t*-Bu, X = Cl)	3.0	*trace*	49^e^	N/A
8^c^	**20c** (R = H, R′ = *t*-Bu, X = Cl)	*n*-BuLi (4.0)	59^e^	0	N/A
9^c^	**20c** (R = H, R′ = *t*-Bu, X = CI)	*s*-BuLi (4.0)	*trace*	60^e^	N/A

a Reaction conditions: **20** (0.05 mmol), THF (0.5 mL).

b Measured by ^1^H NMR spectroscopy using 1,3,5-trimethoxybenzene as an internal standard.

c *n*-hexane:THF (95:5) was used instead of THF.

d Inverse addition of lithium reagent was operated.

e Isolated yields.

### Optimization of Dehalogenation–Aromatization and Completion of the Synthesis

This discovery provided a potential solution to our problem and so we set about optimizing the process. Through optimization, we found that our desired aromatic product could be obtained in increasing yield by (i) switching from THF to a cosolvent system of *n*-hexane:THF (95:5) (entry 2), (ii) increasing the equivalents of *t*-BuLi (entry 3), and (iii) replacing the bromide with a chloride (entry 4). This afforded a 56% yield of the desired product but together with an increased 32% yield of the undesired *tert*-butyl ketone product. Using the carboxylic acid instead of the ester, the undesired *tert*-butyl ketone product could be avoided, giving the desired aromatic product in 66% yield (entry 6). Finally, *n*-BuLi and *s*-BuLi were both tested under the optimized conditions (entries 8 and 9) with only *s*-BuLi providing the desired compound in a slightly reduced yield compared to *t*-BuLi (60 vs 66%). Applying these optimized conditions to our total synthesis worked effectively, and the desired aromatic product **16** was obtained in 77% yield (Scheme 2c). Finally, treatment with TBAF effected deprotection of the TBS groups and afforded beraprost **1** in only 8 steps from enal-lactone **7** (14 steps LLS).

### Mechanistic Studies of Aromatization

**Scheme 3:**
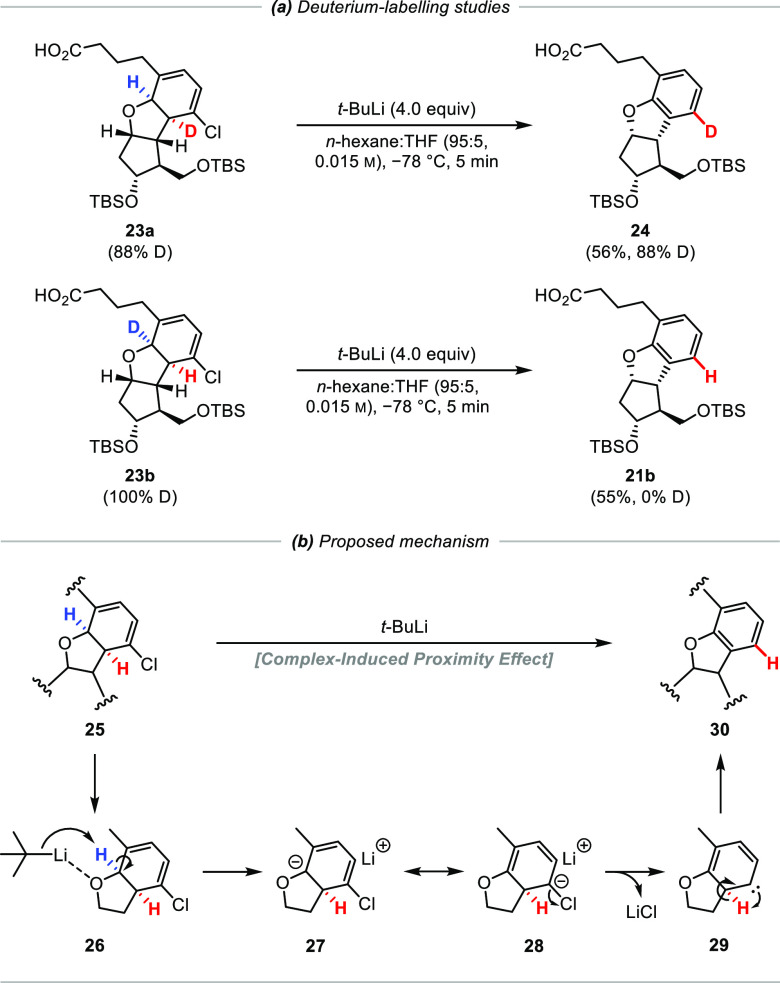
Mechanistic Studies of Aromatization

Having completed the total synthesis, we wanted to understand the mechanism of the intriguing dehalogenation–aromatization process and in particular the fate of the two hydrogen atoms at the pre-aromatized ring junction (Scheme 3a). Thus, mono-deuterated chlorocyclohexadienes **23a** and **23b** were prepared (see the Supporting Information for details), and each was treated with *t*-BuLi under the optimized reaction conditions. Chlorocyclohexadiene **23a** gave aromatized product **24** with complete incorporation of deuterium into the aromatic ring. In contrast, no deuteration was observed when isomer **23b** was used nor when the reaction was quenched with methanol-*d*_4_. The 1,2-hydrogen atom shift that clearly occurred is likely to have resulted from the intermediacy of a carbene species.

Such a species could arise from an ether-directed deprotonation [complex-induced proximity effect (CIPE)] to give **27**/**28** followed by α-elimination to form carbene intermediate **29** (Scheme 3b).^26^ The energy barrier for the resultant 1,2-hydrogen shift of this carbene was calculated to be almost barrierless (1 kcal mol^–1^), which would make deprotonation the slowest step along this pathway. The fact that the chloride gave higher yields than the bromide suggests that lithium–halogen exchange, which would be more efficient with the bromide, is unlikely to be involved. Improved results using non-coordinating solvents also point to the importance of a complex-induced proximity effect to both break up any organolithium aggregates and direct deprotonation adjacent to the ether. The observation that *s*-BuLi was less effective and *n*-BuLi was ineffective to producing the desired aromatic product provides further evidence for our proposed mechanism since the reactivity of the various alkyllithiums to effect α-ether deprotonation decreases in the order of *t*-BuLi > *s*-BuLi ≫ *n*-BuLi.^27^

### Modular Synthesis of Functionalized Indolines

We wanted to explore the generality of the novel IEDDA/cheletropic extrusion and dehalogenation–aromatization process, in particular to determine whether it could be applied to the synthesis of the all-important indoline skeletons as well. In fact, this methodology could prove valuable in the synthesis of C-4/C-7 functionalized indoline scaffolds, which are commonly found in natural products and medicinal chemistry programs.^28^ Most strategies for the synthesis of indolines focus either on the reduction of indoles or the synthesis of the 5-membered nitrogen heterocycle.^29^ Very few of these approaches target the aromatic ring, which would allow for a greater degree of modularity to be accessed in the synthesis of these important scaffolds.

**Scheme 4:**
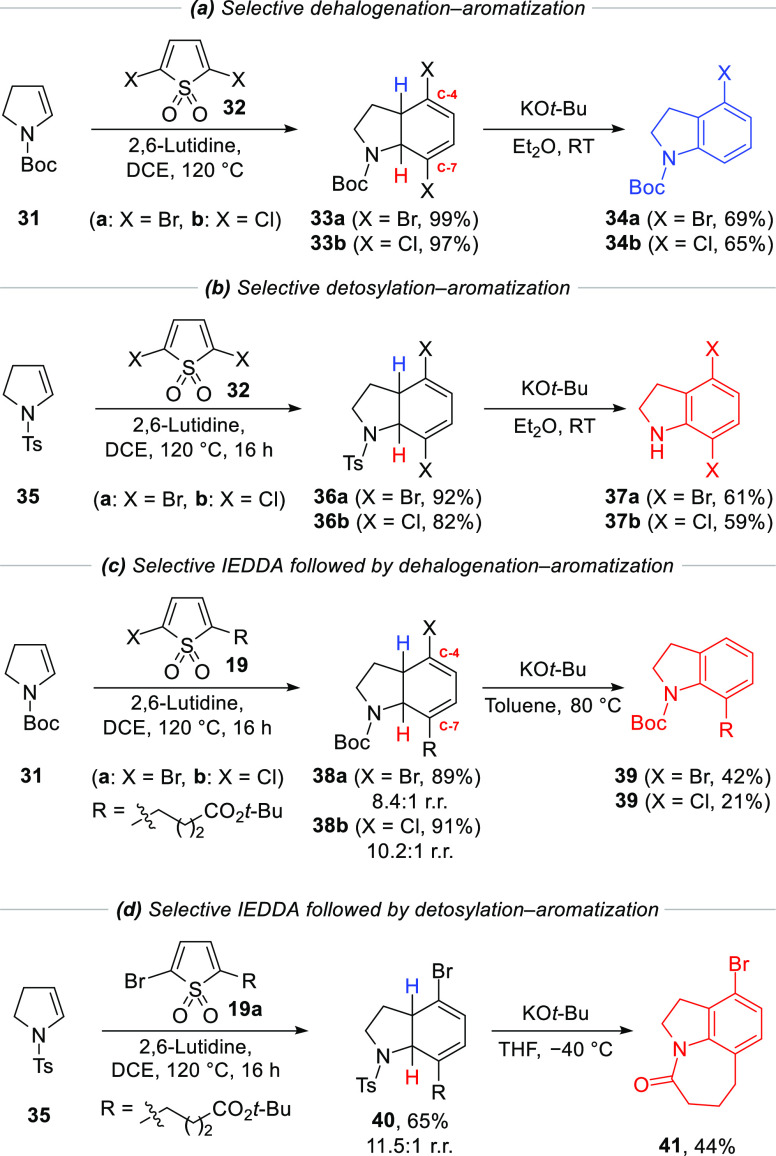
Synthesis of Mono- and Di-Halodienes and Their Selective Dehalogenation– or Detosylation–Aromatization Reactions

The reaction of symmetrical 2,5-dihalogenated thiophene 1,1-dioxides **32a/b** with *N*-Boc-2,3-dihydropyrrole **31** gave di-halodienes **33a/b** in almost quantitative yield (Scheme 4).

However, while subsequent treatment with *t*-BuLi led to a complex mixture, we found that reaction with potassium *tert*-butoxide (KO*t*-Bu) was able to effect dehalogenation–aromatization. In contrast to the dihydrofuran cycloadducts where deprotonation occurred adjacent to the oxygen, in this case, the proton distal to the nitrogen was removed giving 4-bromoindoline **34a** in good yield and as a single regioisomer (Scheme 4). The chlorodiene **33b** also worked equally well, giving **34b** as a single regioisomer. Using *N*-Ts-2,3-dihydropyrrole **35**, a different pathway for elimination–aromatization was available, enabling us to retain both halogens. Thus, following another high yielding IEDDA/cheletropic extrusion, cycloadducts **36a**/**b**, were obtained in 92% yield. Treatment with KO*t*-Bu now resulted in elimination of the tosyl group and led to dibromo- and dichloroindoline **37a** and **37b** in 61 and 59% yield, respectively. It is noteworthy that in the two substrate classes a different proton is removed: with **33a/b** the proton distal to the *N*-Boc group (blue proton) is removed, whereas in the case of the **36a/b**, it is the proton proximal to the *N*-Ts group (red proton) that is removed.

Finally, we explored the IEDDA/cheletropic extrusion of unsymmetrical thiophene 1,1-dioxides **19a/b** with *N*-Boc-2,3-dihydropyrrole **31** and *N*-Ts-2,3-dihydropyrrole **35**. Surprisingly, in each case, this gave the adduct with high regioselectivity in which the alkyl chain was *syn* to the *N*-Boc/*N*-Ts group (the more hindered adduct). Subsequent treatment of cycloadducts **38a/b** with KO*t*-Bu unfortunately resulted in substantial quantities of elimination of the *N*-Boc group, likely due to the fact that the proton distal to the *N*-Boc group (blue proton) would be preferentially removed, by analogy with substrate **33a/b**. A brief optimization revealed that increased preference for the desired dehalogenation–aromatization process was observed in non-polar solvents and that the bromide behaved better than the chloride giving indoline **39** in 42% yield. A Similar treatment of cycloadduct **40** with KO*t*-Bu also resulted in competing elimination, but nevertheless, elimination of the tosyl group followed by tautomerization and intramolecular amidation delivered aza-tricycle **41** in 44% yield. It is notable that such aza-tricyclic skeletons are prevalent in many natural products and bioactive molecules.^30^

### Computational Studies of Regioselective IEDDA

**Scheme 5:**
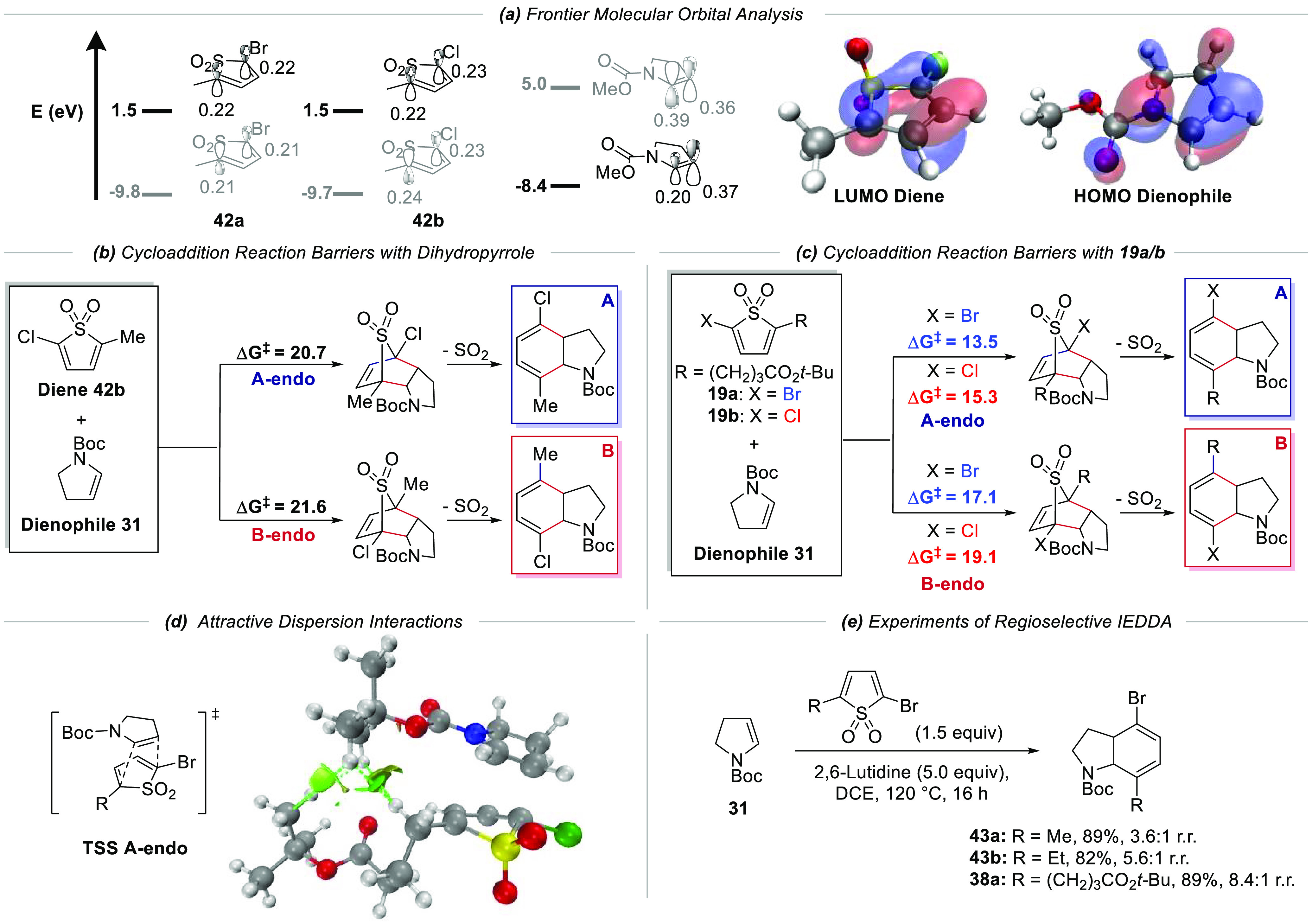
Computational Studies of Regioselective IEDDA (a) Frontier molecular orbital energies and coefficients (HF/6-31G). The lobes of reacting orbital coefficients are expected to match in magnitude to have a good overlap for reactivity. (b) Reaction energies for the cycloaddition reaction with dihydropyrrole. (c) Reaction energies for the cycloaddition reaction with **19a/19b**. (d) Plot of noncovalent interactions (NCI)^32^ showing the weakly attractive van der Waals interaction consistent with LD forces in the TS of major isomer formation, which is absent in the TS of minor isomer formation (B3LYP/6-31G* with D3 dispersion correction and PCM solvation for dichloroethane). Dashed green lines have been included to indicate the location of the surfaces between the bulky alkyl groups. (e) Experiments of regioselective IEDDA.

The high regioselectivity observed in the IEDDA/cheletropic extrusion in favor of the more hindered adduct was unexpected and so we performed computational analysis to try to understand the origin of the selectivity. One possibility was that the cycloaddition was governed by electronic factors in which the polarity of the HOMO of the enamide matched the polarity of the LUMO of the diene. However, frontier molecular orbital calculations^31^ on a model dienophile, *N*-CO_2_Me-2,3-dihydro-1*H*-pyrrole (truncated **31**), and model dienes, 2-methyl-5-halothiophene 1,1-dioxide, **42a/b** (truncated **19a/b**), showed that the LUMO lobes of the unsymmetrical diene, despite having very different electron donating and withdrawing groups attached, had similar coefficients at the two ends, indicating that there would be no preferred orientation, which can be seen when visualizing the relevant orbitals (Scheme 5a). Modeling the cycloaddition reaction between **31** and the same diene **42b** gave rise to two endo TSs (we established that the *endo* TSs were lower than the *exo* TSs in the reaction of diene **42b** with dihydrofuran – see ESI) in which the one where the Boc group was *syn* to the alkyl chain, leading to the observed and more hindered product, was 0.9 kcal mol^–1^ lower in energy than the alternative where the Boc group was *anti* to the alkyl chain (Scheme 5b). This was not enough to account for the experimental selectivity seen, and so the endo TSs for the full system, **19a/b** as the diene and **31** as the dienophile, were modeled. They now showed a substantial difference in energy of ∼3.5 kcal mol^–1^ when London dispersion interactions were included. The London dispersion interactions were found to have contributed between 0.6 and 3.9 kcal mol^–1^, depending on the level of theory with which they were studied. This suggests that a significant portion of the selectivity can be accounted for by London dispersion interactions, stabilizing the former TS (Scheme 5d), which is not present in the transition state with truncated diene 2-methyl-5-chlorothiophene 1,1-dioxide **42b**. This was verified experimentally: using smaller substituents on the diene (Me, Et) in place of the longer propanyl ester led to progressively lower regioselectivity (Scheme 5e). It may seem unusual that LD interactions could lead to such high selectivity, but there are numerous cases where it is surprisingly large and can dominate selectivity.^33,34^ Our observations add to the growing recognition of the importance of London dispersion interactions contributing to selectivity in chemical reactions.^33^

## Conclusions

In conclusion, we have developed a highly stereoselective and modular synthesis of the potent anti-platelet agent, beraprost, in only 14 steps (LLS) in >99% e.e. The two sidechains of beraprost were readily introduced through a 1,4-conjugate addition of a higher-order cyanocuprate and a powerful IEDDA/cheletropic extrusion process, which produced a chloro-diene system that set the stage for a unique dehalogenation–aromatization reaction using *t*-BuLi. The chloride present in the thiophene 1,1-dioxide served a dual role: it inhibited cyclodimerization of the thiophene 1,1-dioxide and enabled a unique dehalogenation–aromatization. Mechanistic analysis of this step through deuterium-labeling studies showed the intermediacy of a carbene which underwent an aromatizing 1,2-hydrogen shift. Finally, we showed the applicability of our IEDDA/cheletropic extrusion and dehalogenation–aromatization process to the synthesis of indolines. Reaction of symmetrical 2,5-dihalogenated thiophene 1,1-dioxides and 2,3-dihydropyrroles gave cycloadducts which underwent selective elimination–aromatization to afford either C-4 halogenated indolines or C-4/C-7 dihaloindolines depending on the nature of the nitrogen protecting group. Using nonsymmetric 2-halo,5-alkyl thiophene 1,1-dioxides with 2,3-dihydropyrroles gave cycloadducts with high regioselectivity. Subsequent dehalogenation–aromatization then gave the C-7 indoline, providing a unique and modular approach to these medicinally relevant scaffolds.

## Supporting Information Available

Experimental procedures, spectroscopic data (1H NMR, 13C NMR, IR, HRMS) (PDF)
Geometry coordinates for calculated structures (XYZ)

## Supplementary Material

ja3c04582_si_001.pdf

ja3c04582_si_002.xyz
