# Supplementary material for: De Novo Synthesis of Dihydrobenzofurans and Indolines and Its Application to a Modular, Asymmetric Synthesis of Beraprost
Source: J Am Chem Soc. 2023 Jun 16;145(25):14124–32. doi: 10.1021/jacs.3c04582 (PMC10311537; doi:10.1021/jacs.3c04582)
Supplement: Supplementary file 1 — ja3c04582_si_001.pdf [file ja3c04582_si_001.pdf]

De Novo Synthesis of Dihydrobenzofurans and Indolines and Its Application to a Modular, Asymmetric Synthesis of Beraprost

Ze-Shu Wang, Steven H. Bennett, Bilal Kicin, Changcheng Jing, Johan A. Pradeilles, Karen Thai, James R. Smith, P. David Bacoş, Valerio Fasano, Carla M. Saunders, and Varinder K. Aggarwal*

University of Bristol, School of Chemistry, Cantock's Close, Bristol BS8 1TS, UK

*e-mail: v.aggarwal@bristol.ac.uk

Table of Contents

1. General Experimental .... - 4 -
1.1. Thin-Layer Chromatography (TLC) .... - 4 -
1.2. Gas Chromatography–Mass Spectrometry (GCMS) .... - 4 -
1.3. Liquid Chromatography–Mass Spectrometry (LCMS) .... - 5 -
1.4. Column Chromatography........ - 5 -
1.5. Solvents........ - 5 -
1.6. Analysis........ - 5 -
1.7 Naming of Compounds .... - 7 -
2. All Procedures........ - 8 -
2.1. General Procedure A .... - 8 -
2.2. General Procedure B .... - 9 -
2.3. General Procedure C .... - 10 -
3. The Total Synthesis of Beraprost........ - 11 -
3.1. Synthesis of Beraprost α-Sidechain........ - 11 -
3.2. Synthesis of Beraprost ω-Sidechain .... - 13 -
3.3. Synthesis of Beraprost .... - 21 -
4. Dehalogenation–Aromatization Model Studies .... - 32 -
4.1. Synthesis of *tert*-Butyl Protected Brominated Thiophene 1,1-Dioxide........ - 32 -
4.2. Synthesis of *tert*-Butyl Protected Chlorinated Thiophene 1,1-Dioxide........ - 34 -
4.3. Synthesis of Corey Lactone Dienes .... - 37 -
4.4. Optimization of Corey Lactone Diene Dehalogenation–Aromatization .... - 44 -
5. Deuterium-Labelling Studies .... - 50 -
6. Substrate Scope........ - 59 -
6.1 Synthesis of Halogenated Thiophene 1,1-Dioxides........ - 59 -
6.2. Inverse-Electron Demand Diels–Alder/Cheletropic Extrusion .... - 60 -
6.3. Selective Dehalogenation–Aromatization .... - 69 -

6.4. Selective Detosylation–Aromatization .... - 71 -

6.5. Dehalogenation–Aromatization of Mono-Halodienes .... - 72 -

6.6. Detosylation–Aromatization of Mono-Halodienes .... - 73 -

7. DFT Calculations .... - 74 -

8. NMR Spectra (with Table of Contents) .... - 81 -

8.1. The Total Synthesis of Beraprost .... - 82 -

8.2. Dehalogenation–Aromatization Model Studies .... - 100 -

8.3. Deuterium-Labelling Studies .... - 111 -

8.4. Substrate Scope .... - 117 -

9. References .... - 135 -

1. General Experimental

Unless otherwise stated, all reactions were carried out using oven- or flame-dried glassware under a positive pressure of dry nitrogen or argon. All starting materials were bought from commercial suppliers (e.g. Sigma-Aldrich, Fisher Scientific, Alfa Aesar, Acros Organics etc.) and used without further purification unless indicated otherwise. *tert*-Butyl lithium (1.7 M in pentane) [CAS: 594-19-4] was purchased from Sigma-Aldrich and used as received. Methyl lithium (1.6 M in Et_2O) [CAS: 917-54-4] was purchased from Acros Organics and used as received. All organolithiums should be carefully, and regularly, titrated.[1] Potassium tert-butoxide (sublimed grade, 99.99% trace metals basis) was purchased from Sigma-Aldrich and used as received. *N*-Bromo- and *N*-chlorosuccinimide were recrystallized prior to use (NBS from water; NCS from chloroform) and stored under an inert atmosphere, in the dark, in a freezer.[2] Prior to use, 2,6-lutidine was treated with anhydrous $AlCl_3$ and distilled under reduced pressure.[2] Triethylamine (Et_3N) and trimethylsilyl chloride (TMS-Cl) were both distilled over CaH_2 under reduced pressure prior to use.[2]

Room (ambient) temperature (RT) means 18–25 °C as determined by the oil bath. Reactions were stirred magnetically using a temperature-regulated hotplate/stirrer and monitored by thin-layer chromatography (TLC), gas chromatography–mass spectrometry (GCMS), reverse-phase liquid chromatography–mass spectrometry (LCMS) or crude nuclear magnetic resonance spectroscopy (NMR) where appropriate.

Procedures using ozone were conducted with a C-Lasky C-L010-DT ozone generator, which had an internal air pump that generated 2 g/h ozone at maximum output (internal air pump 10 L/min) *via* corona discharge between the surfaces of two quartz tubes. In the case of reactions using O_3-$O_{2(g)}$, an external oxygen cylinder was connected to the ozone generator that produced 10 g/h ozone (oxygen fed 6 L/min).

1.1. Thin-Layer Chromatography (TLC)

Thin-layer chromatography (TLC) was performed using aluminum-backed silica plates (Merck Keiselgel 60 F_{254}) and visualised by UV light (λ = 254–312 nm) and/or by staining with anisaldehyde, phosphomolybdic acid, potassium permanganate or vanillin solutions, which were subsequently heat-treated.

1.2. Gas Chromatography–Mass Spectrometry (GCMS)

Gas chromatography–mass spectrometry (GCMS) was performed on an Agilent 7820A GCMS equipped with a HP-5MS UI column (30 m × 0.25 mm × 0.25 μm).

1.3. Liquid Chromatography–Mass Spectrometry (LCMS)

Reverse-phase liquid chromatography–mass spectrometry (LCMS) was performed on an Agilent 1260 Infinity II system with Agilent PoroShell 120 EC-C18 column (3.0 × 50 mm, 2.7 µm) using MeCN–H_2O gradients (10→90% MeCN in H_2O) and observing at 214 nm.

1.4. Column Chromatography

Flash column chromatography was carried out using either Sigma-Aldrich silica gel 60 (40 – 63 µm, 230 – 400 mesh) or using a Biotage® Isolera™ Spektra automated flash column chromatography system.

1.5. Solvents

An activated alumina-based *Anhydrous Engineering* drying system was used to obtain the following dry solvents for reactions: tetrahydrofuran (THF), diethyl ether (Et_2O), dichloromethane (DCM), acetonitrile (MeCN), Hexane and Toluene. These solvents were transferred, stored and used under a positive pressure of nitrogen in a Young's valve-sealed, flame-dried and nitrogen purged Strauss flask containing previously activated 3 Å molecular sieves, which were activated by microwave irradiation (150 °C, 30 s cycles for 4 mins) followed by flame-drying with a Bunsen burner. Anhydrous solvents different to those stated above were used as commercially supplied and all reactions were carried out using dry solvents unless stated in the procedure.

Solvents for purification or non-anhydrous reactions such as dichloromethane (DCM), diethyl ether (Et_2O), ethyl acetate (EtOAc), acetonitrile (MeCN), pentane, hexane, heptane and petroleum ether (40 – 60 °C) were used as obtained from suppliers (e.g. Sigma-Aldrich, Fisher Scientific, VWR) without further purification (> 98%).

1.6. Analysis

Nuclear Magnetic Resonance (NMR) data (^{1}H, ^{11}B, ^{13}C, ^{19}F, HSQC, HMBC, COSY) was recorded using either a JEOL ECS 300 MHz, Bruker 400 MHz, JEOL 400 MHz, Varian 400 MHz or Bruker 500 MHz NMR spectrometer. Data was recorded at 298K unless specified and all ^{13}C NMR spectra were broadband ^{1}H decoupled. Deuterated solvents (e.g. $CDCl_3$, MeOH-d_4, D_2O, DMSO-d_6, C_6D_6, etc.) were used as obtained from Sigma-Aldrich. Chemical shifts (δ / ppm) are reported relative to the solvent's reference peaks (e.g. ^{1}H - $CDCl_3$, 7.26 ppm; ^{13}C - $CDCl_3$, 77.16 ppm). Data for ^{1}H NMR spectra are reported as follows: chemical shift (δ / ppm), peak multiplicity (s, singlet; d, doublet; t, triplet; q, quartet; quin, quintet; m, multiplet or unresolved, *br* s, broad singlet), integration, coupling constants (Hz) and peak assignment.

High Resolution Mass Spectroscopy (HRMS) data was recorded by the University of Bristol Mass Spectrometry Services Laboratory using electrospray ionisation (ESI; Brüker Daltonics micrOTOF II), chemical ionisation (CI; VG AutoSpec), electron impact (EI; VG Micromass AutoSpec (Triple-sector)) or matrix-assisted laser desorption/ionisation (MALDI) techniques. Low resolution mass spectroscopy (LRMS) was obtained on an Agilent 1260 Infinity II system with Agilent PoroShell 120 EC-C18 columns (3.0 × 50 mm, 2.7 μm) using MeCN-H_2O gradients (typically, 10$\rightarrow$ 90% MeCN-H_2O) at wavelengths of 190, 214 or 254 nm.

Fourier Transformed Infra-Red (FTIR) spectra were obtained using a Perkin-Elmer Spectrum One ATR FT-IR spectrometer, loading compounds as either thin films or solids, with select peak values being quoted in wave numbers (cm^{-1}). Only selected absorption maxima (ν_{max}) are reported.

Optical Rotations ([a]$_D$ values) were obtained using a Bellingham and Stanley Ltd. ADP 220 Polarimeter.

Melting Points were collected using a Kofler hot-stage microscope apparatus or a Cole-Parmer Stuart SMP30 melting point apparatus and are reported in degrees Celsius (°C).

X-Ray Diffraction on **7** was carried out at 100(2) K on a Bruker D8 Venture diffractometer using Cu-K_α (λ = 1.54178 Å) while **41** was carried out at 150(2) K on a Bruker APEX II diffractometer using Mo-K_α radiation (λ = 0.71073 Å). Intensities were integrated in SAINT[3] and absorption corrections based on equivalent reflections were applied using SADABS.[4] Structure **7** was solved using ShelXT[5] and structure **41** was solved using ShelXS (G. M. Sheldrick, *Acta Crystallogr., Sect. A: Found. Crystallogr.*, 2008, **64**, 112-122.), both structures were refined by full matrix least squares against F^2 in ShelXL[6,7] using Olex2.[8] All of the non-hydrogen atoms were refined anisotropically. While all of the hydrogen atoms were located geometrically and refined using a riding model. In **41** C10A and C11A were disordered across a symmetry element, the occupancies were necessarily fixed at 0.5. Restraints and constraints were applied to maintain chemically sensible geometric and thermal parameters. The absolute structure for **7** and **41** were not able to be conclusively determined crystallographically. Crystal structure and refinement data is given below. Crystallographic data for **7** and **41** has been deposited with the Cambridge Crystallographic Data Centre as supplementary publication CCDC 2172054 and 2226750. Copies of the data can be obtained free of charge on application to CCDC, 12 Union Road, Cambridge CB2 1EZ, UK [fax(+44) 1223 336033, e-mail: deposit@ccdc.cam.ac.uk].

1.7 Naming of Compounds

Compound names are those generated by ChemDraw Professional 16.0 software (PerkinElmer), following the IUPAC nomenclature.

2. All Procedures

2.1. General Procedure A: Synthesis of Bromothiophene 1,1-Dioxides

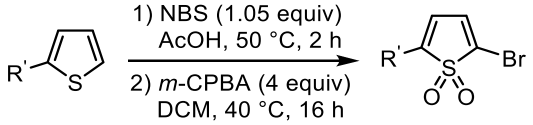

Thiophene (10.0 mmol, 1.00 equiv) was added to acetic acid (50.0 mL, 0.20 M) at room temperature and the mixture was stirred (ca. 30 s) to provide a homogenous solution. To this was added *N*-bromosuccinimide (NBS, 1.87 g, 10.5 mmol, 1.05 equiv) and following its complete dissolution, the reaction was placed into a preheated oil bath at 50 °C and stirred at this temperature for 2 h. The reaction was then cooled to room temperature then added to 3 M NaOH (350 mL) dropwise at 0 °C. Following this, the mixture was extracted with Et_2O (3 × 150 mL) and the organic phases were combined, dried with $MgSO_4$, and concentrated under reduced pressure. The crude residue was used in next step without further purification.

The crude bromothiophene (10 mmol) was dissolved in CH_2Cl_2 (55 mL) and cooled to 0 °C. To this was added a solution of *m*-CPBA (≥70% purity, 11.6 g, 46.8 mmol, 4.00 equiv) in CH_2Cl_2 (126 mL) dropwise. Following this, the reaction was warmed to room temperature, a reflux condenser was attached, and the reaction mixture placed into a pre-heated oil bath at 40 °C and stirred at this temperature for 16 h. The reaction was cooled to room temperature and then to −78 °C (dry ice/acetone) for 15 min to precipitate out both *m*-CPBA and *m*-chlorobenzoic acid. A Teflon cannula for filtration under a positive pressure of nitrogen was then prepared by inserting a Teflon cannula through a Suba-Seal® and wrapping a small piece of filter paper around this end using PTFE tape.[A] The cannula was placed into the flask containing the crude product at −78 °C and a nitrogen balloon was inserted to facilitate cannula transfer into a 500 mL receiving flask. Once completed, further portions of CH_2Cl_2 at −78 °C (2 × 50 mL) were added to the transferring flask and the cannula transfer repeated. The filtrate was concentrated under reduced pressure to ca. ~30 mL and silica gel was added in order to dry-load on to the column. The dry-loaded crude material was purified by silica gel column chromatography to afford desired products.

Notes: (**A**) A Teflon cannula for filtration under an inert atmosphere is prepared by cutting a piece of HPLC tubing to the required length and threading this through two appropriately sized Suba-Seals®. In our case, BOLA PTFE tubing (ID 1.5 mm, OD 2.5 mm) from Sigma-Aldrich was used (**Figure 1**).

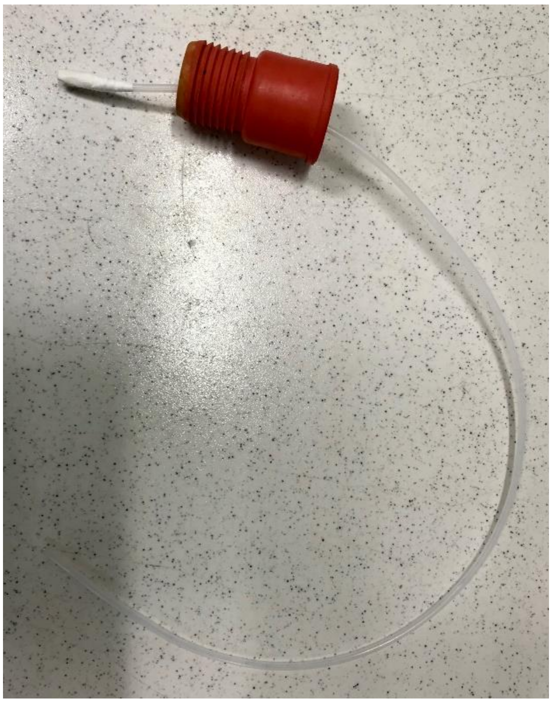

Figure 1. Image of the teflon cannula used for inert atmosphere filtration of *m*-CPBA and *m*-chlorobenzoic acid from the crude reaction mixture.

2.2. General Procedure B: Inverse-Electron Demand Diels–Alder/Cheletropic Extrusion of 2,3-Dihydropyrroles and Halogenated Thiophene 1,1-Dioxides

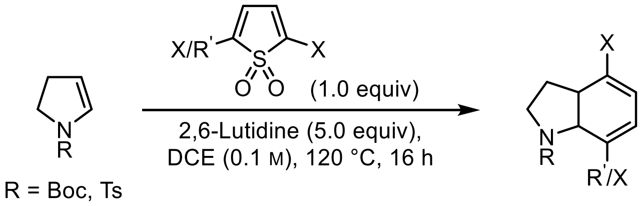

To a flame-dried microwave vial containing a solution of the thiophene 1,1-dioxide (0.20 mmol, 1.00 equiv) and corresponding protected 2,3-dihydropyrrole (0.20 mmol, 1.00 equiv) in 1,2-dichloroethane (DCE, 2.00 mL) was added 2,6-lutidine (0.12 mL, 1.00 mmol, 5.00 equiv). The microwave vial was capped and placed into a pre-heated oil bath at 120 °C and stirred for 16 h. Following this, the reaction was allowed to cool to room temperature, concentrated under reduced pressure and purified directly by flash column chromatography to afford the desired halodienes.

2.3. General Procedure C: Selective Dehalogenation–Aromatization (R = Boc) or Detosylation–Aromatization (R = Ts) using Potassium *tert*-Butoxide (KO*t*-Bu)

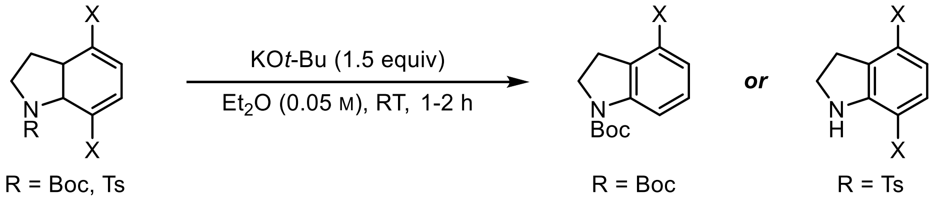

In a glove box, to a 7.5 mL glass sample vial containing a solution of the di-halodiene (0.10 mmol, 1.00 equiv) in Et_2O (2.00 mL) at room temperature was added KO*t*-Bu (16.8 mg, 0.15 mmol, 1.50 equiv) and the reaction was removed from glove box and stirred at this temperature for 1-2 h. Following this, the reaction was quenched with H_2O (2 mL), extracted with Et_2O (3 × 4 mL) and the organic phases were combined, dried with Na_2SO_4, filtered, and concentrated under reduced pressure. The crude residue obtained was purified by flash column chromatography to afford the desired dehalogenated or detosylated indoline products.

3. The Total Synthesis of Beraprost

3.1. Synthesis of Beraprost α-Sidechain

4-(5-chloro-1,1-dioxidothiophen-2-yl)butanoic acid (4)

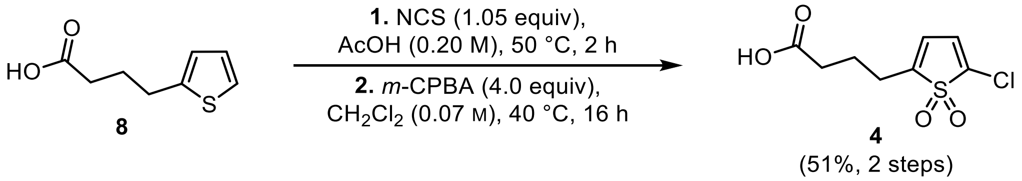

4-(2-Thienyl)butyric acid **8** (1.71 mL, 11.7 mmol, 1.00 equiv) was added to acetic acid (59.0 mL, 0.20 M) at room temperature and the mixture was stirred (ca. 30 s) to provide a homogenous solution. To this was added *N*-chlorosuccinimide (NCS, 1.65 g, 12.3 mmol, 1.05 equiv)[A] and following its complete dissolution, the reaction was placed into a pre-heated oil bath at 50 °C and stirred at this temperature for 2 h. The reaction was then cooled to room temperature and concentrated under reduced pressure. Et_2O (20 mL) was then added, and the white solid (succinimide) was filtered, washing with further portions of Et_2O (2 × 20 mL). The filtrate was concentrated under reduced pressure and the precipitation/filtration/concentration procedure was repeated a further 2× to give a red oil.

The crude material was dissolved in CH_2Cl_2 (55 mL) and cooled to 0 °C. To this was added a solution of *m*-CPBA (≥70% purity, 11.6 g, 46.8 mmol, 4.00 equiv) in CH_2Cl_2 (126 mL) dropwise (syringe pump: 1 mL/min). Following this, the reaction was warmed to room temperature, a reflux condenser was attached, and the reaction mixture placed into a pre-heated oil bath at 40 °C and stirred at this temperature for 16 h. The reaction was cooled to room temperature and then to −78 °C (dry ice/acetone) for 15 min to precipitate out both *m*-CPBA and *m*-chlorobenzoic acid.[B] A Teflon cannula for filtration under a positive pressure of nitrogen was then prepared by inserting a Teflon cannula through a Suba-Seal® and wrapping a small piece of filter paper around this end using PTFE tape.[C] The cannula was placed into the flask containing the crude product at −78 °C and a nitrogen balloon was inserted to facilitate cannula transfer into a 500 mL receiving flask. Once completed, further portions of CH_2Cl_2 at −78 °C (2 × 50 mL) were added to the transferring flask and the cannula transfer repeated. The filtrate was concentrated under reduced pressure to ca. ~30 mL and silica gel was added in order to dry-load on to the column. The dry-loaded crude material was purified by silica gel column chromatography (10–70% EtOAc in *n*-pentane + 1% AcOH) to afford 4-(5-chloro-1,1-dioxidothiophen-2-yl)butanoic acid **4** (1.43 g, 51% over two steps) as a colourless crystalline solid.

Notes: (**A**) *N*-Chlorosuccinimide was recrystallised from chloroform prior to use.[2] (**B**) Other temperatures (e.g., 0 °C, −20 °C) were also attempted for the precipitation of *m*-CPBA and *m*-chlorobenzoic acid but proved more ineffective compared to −78 °C. (**C**) A Teflon cannula for filtration under an inert atmosphere is prepared by cutting a piece of HPLC tubing to the required length and threading this through two appropriately sized Suba-Seals®. In our case, BOLA PTFE tubing (ID 1.5 mm, OD 2.5 mm) from Sigma-Aldrich was used (**Figure 1**).

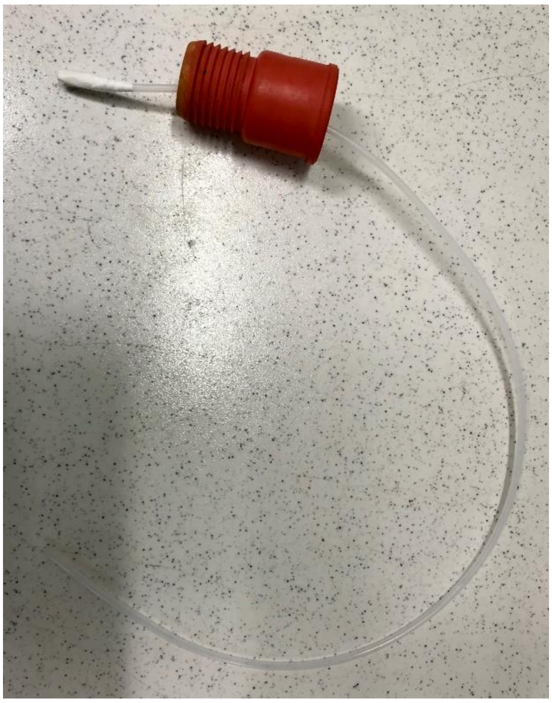

Figure 1. Image of the teflon cannula used for inert atmosphere filtration of *m*-CPBA and *m*-chlorobenzoic acid from the crude reaction mixture.

MP: 110–112 °C

TLC: R_f = 0.16 (50% EtOAc in *n*-hexane)

^{1}H NMR (500 MHz, Chloroform-*d*) *δ* 6.59 (d, *J* = 4.9 Hz, 1H), 6.45 (dt, *J* = 4.9, 1.9 Hz, 1H), 2.61 (td, *J* = 7.4, 1.9 Hz, 2H), 2.48 (t, *J* = 7.4 Hz, 2H), 2.03 (p, *J* = 7.4 Hz, 2H) ppm

^{13}C NMR (126 MHz, Chloroform-*d*) *δ* 178.8, 142.5, 132.4, 123.0, 122.6, 32.8, 24.6, 21.7 ppm

HRMS (*m/z*): (ESI) calculated for $C_8H_9ClNaO_4S$ $[M+Na]^+$: 258.9808, found: 258.9803

IR (thin film) ν_{max}: 3079, 2962, 2919, 2683, 1698, 1565, 1443, 1300, 1140, 931, 843, 720 and 629 cm^{-1}

3.2. Synthesis of Beraprost ω-Sidechain

3-(Triethylsilyl)propiolaldehyde (10)

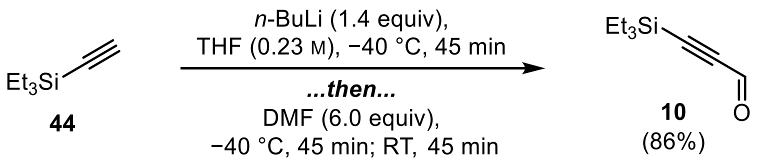

According to a procedure outlined by Lee and co-workers,[9] *n*-butyllithium (*n*-BuLi, 62.4 mL, 99.8 mmol, 1.40 equiv, 1.60 M in hexanes) was added slowly dropwise (syringe pump: 1 mL/min) to a stirred solution of (triethylsilyl)acetylene **44** (10.0 g, 71.3 mmol, 1.00 equiv) in anhydrous THF (310 mL, 0.23 M) at −40 °C. The resulting light-yellow solution was stirred at this temperature for 45 min before anhydrous DMF (33.1 mL, 428 mmol, 6.00 equiv) was added dropwise (syringe pump: 3 mL/min) and the reaction was left to stir for a further 45 min. The cooling bath was then removed, and the solution was allowed to warm to room temperature for 1 hour before stirring for an additional 45 min. The reaction was then poured into a separating funnel containing 1 M aqueous HCl (300 mL) and extracted with Et_2O (3 × 200 mL). The organic phases were combined and washed with 1 M aqueous HCl (300 mL), brine (150 mL), and H_2O (150 mL). Finally, the organic phase was dried with $MgSO_4$, filtered, and concentrated under reduced pressure to afford a crude oil, which was purified by flash column chromatography (0–10% Et_2O in *n*-hexane) to give ynal **10** (10.4 g, 86%) as a colourless oil.

TLC: R_f = 0.56 (5% Et_2O in *n*-hexane); anisaldehyde stained

^{1}H NMR (400 MHz, Chloroform-*d*) δ 9.16 (s, 1H), 1.00 (t, *J* = 7.9 Hz, 9H), 0.68 (qd, *J* = 7.8, 0.7 Hz, 6H) ppm

^{13}C NMR (101 MHz, Chloroform-*d*) δ 176.7, 103.6, 101.4, 7.3, 3.8 ppm

HRMS (*m/z*): (APCI) calculated for $C_9H_{17}OSi$ $[M+H]^+$: 169.1043, found: 169.1041

Analytical data is consistent with that reported in the literature.[9]

(2*S*,3*S*)-2-Methyl-5-(triethylsilyl)pent-4-yne-1,3-diol (12)

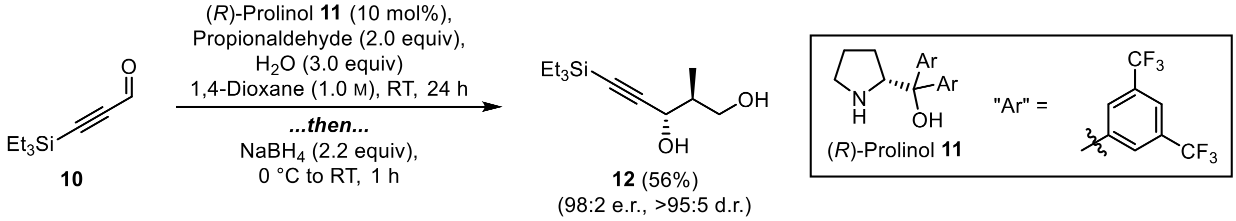

According to a procedure outlined by Kishi and co-workers, [10] (*R*)-2-[bis(3,5-bis-trifluoromethyl-phenyl)hydroxymethyl]pyrrolidine [11] ((*R*)-Prolinol **11**, 780 mg, 1.49 mmol, 10.0 mol%), H_2O (0.80 mL, 44.6 mmol, 3.00 equiv) and propionaldehyde (2.14 mL, 29.7 mmol, 2.00 equiv)[A] were added sequentially to a stirring solution of 3-(triethylsilyl)propiolaldehyde **10** (2.50 g, 14.9 mmol, 1.00 equiv) in 1,4-dioxane (14.9 mL, 1.00 M) at RT. The reaction mixture was stirred at this temperature for 24 h before cooling to 0 °C and adding $NaBH_4$ (1.24 g, 32.7 mol. 2.20 equiv) portionwise before warming to RT and stirring for 1 h. The reaction was then cooled to 0 °C and carefully quenched with 1.00 M pH 7 NaH_2PO_4 buffer (100 mL) before transferring to a separating funnel and extracting with EtOAc (4 × 100 mL).[B] The organic phases were combined, washed with H_2O (50 mL) then brine (100 mL), dried with Na_2SO_4, filtered, and concentrated under reduced pressure to give a crude residue.[C] This crude material was then purified by column chromatography (5–30% EtOAc in *n*-hexane)[D] to give diol **12** (1.89 g, 56%, 98.5:1.5 e.r., >95:5 d.r.)[E] as a colourless oil.

Notes: (**A**) Propionaldehyde was freshly distilled prior to use. (**B**) Excess salts can precipitate out at this stage; however, this can be overcome by diluting with water and using an equivalent volume of EtOAc for the extraction. (**C**) ^1H NMR analysis of the crude product revealed the diastereoselectivity of the process (anti:syn) to be 9.1:1.0. (**D**) The diastereomeric ratio was improved during flash column chromatography to give the desired anti product in >95:5 d.r.. (**E**) The ^1H and ^{13}C NMR data of diol **12** match that reported in the literature; however, it should be noted that at high concentrations, diol **12** forms a hydrogen bond network in benzene-d_6 and this causes the NMR peaks to shift. Thus, it is important when comparing with the literature data to take into account this concentration dependence and run the NMR at lower concentration (~10 mg/mL is sufficient).

TLC: R_f = 0.38 (20% acetone in toluene); anisaldehyde stained

^{1}H NMR (500 MHz, Benzene-d_6) δ 4.29 (dd, J = 7.0, 3.6 Hz, 1H), 3.61 (d, J = 10.7 Hz, 1H), 3.42 (dd, J = 10.7, 7.0 Hz, 1H), 2.78 (br. s, 1H), 2.11 (br. s, 1H), 1.85 (hd, J = 7.0, 4.0 Hz, 1H), 1.06 (t, J = 7.9 Hz, 9H), 0.95 (d, J = 7.0 Hz, 3H), 0.62 (q, J = 7.9 Hz, 6H) ppm

^{13}C NMR (126 MHz, Benzene-d_6) δ 108.3, 87.2, 67.0, 66.1, 41.8, 13.2, 7.8, 4.8 ppm

HRMS (*m/z*): (ESI) calculated for $C_{12}H_{24}NaO_2Si$ $[M+Na]^+$: 251.1438, found: 251.1442

Analytical data is consistent with that reported in the literature.[10]

The e.r. of **12** was determined by benzoylation and subsequent analysis by chiral HPLC.

(2*S*,3*S*)-3-Hydroxy-2-methyl-5-(triethylsilyl)pent-4-yn-1-yl (45)

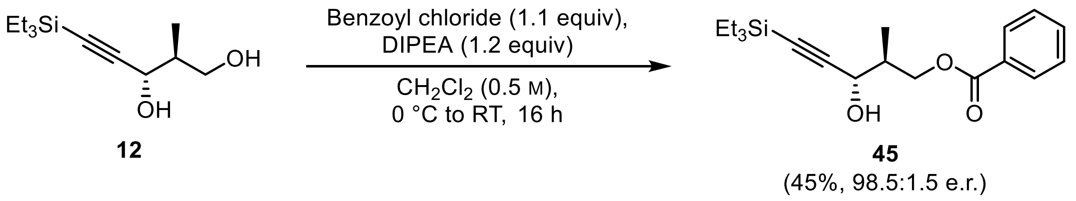

To a solution of diol **12** (50.0 mg, 0.22 mmol, 1.00 equiv) in anhydrous CH_2Cl_2 (2.20 mL, 0.50 M) at 0 °C (ice bath) was added *N*,*N*-diisopropylethylamine (DIPEA, 46.0 µL, 0.26 mmol, 1.20 equiv) followed by benzoyl chloride (28.0 µL, 0.24 mmol, 1.10 equiv). The reaction was slowly warmed to room temperature (the ice bath was not removed) and the solution was stirred at this temperature for 16 h before methanol (0.50 mL) was added. The reaction was then concentrated under reduced pressure and the crude residue obtained was purified by column chromatography (0–20% EtOAc in *n*-hexane) to give benzoyl ester **45** (33 mg, 45%, 98.5:1.5 e.r.) as a colourless oil.

TLC: R_f = 0.55 (20% EtOAc in *n*-hexane)

^{1}H NMR (400 MHz, Benzene-d_6) δ 8.07 (d, J = 7.5 Hz, 2H), 7.11 (t, J = 7.5 Hz, 1H), 7.03 (t, J = 7.5 Hz, 2H), 4.40 – 4.30 (m, 3H), 2.09 (app. hept, J = 6.1 Hz, 1H), 1.79 – 1.69 (br. m, 1H), 1.09 – 1.01 (m, 12H), 0.63 – 0.55 (m, 6H) ppm

^{13}C NMR (101 MHz, Benzene-d_6) δ 166.4, 132.9, 130.9, 129.9, 128.6, 107.2, 87.8, 66.4, 64.6, 39.7, 12.7, 7.7, 4.7 ppm

HRMS (*m/z*): (ESI) calculated for $C_{19}H_{29}O_3Si$ $[M+H]^+$: 333.1880, found: 333.1876

IR (thin film) ν_{max}: 3456, 2955, 2910, 2875, 1721, 1703, 1602, 1452, 1388, 1270, 1070, 1025 and 708 cm^{-1}

$[\alpha]_D^{24}$**:** +33.0 (c = 1.00, $CHCl_3$)

Chiral HPLC: Daicel Chiralpak® IA column with guard, 2% *i*-PrOH in *n*-hexane, 1.0 mL/min, ambient temperature, 230 nm, t_R = 25.2 min (minor), t_R = 22.6 min (major), 98.5:1.5 e.r.

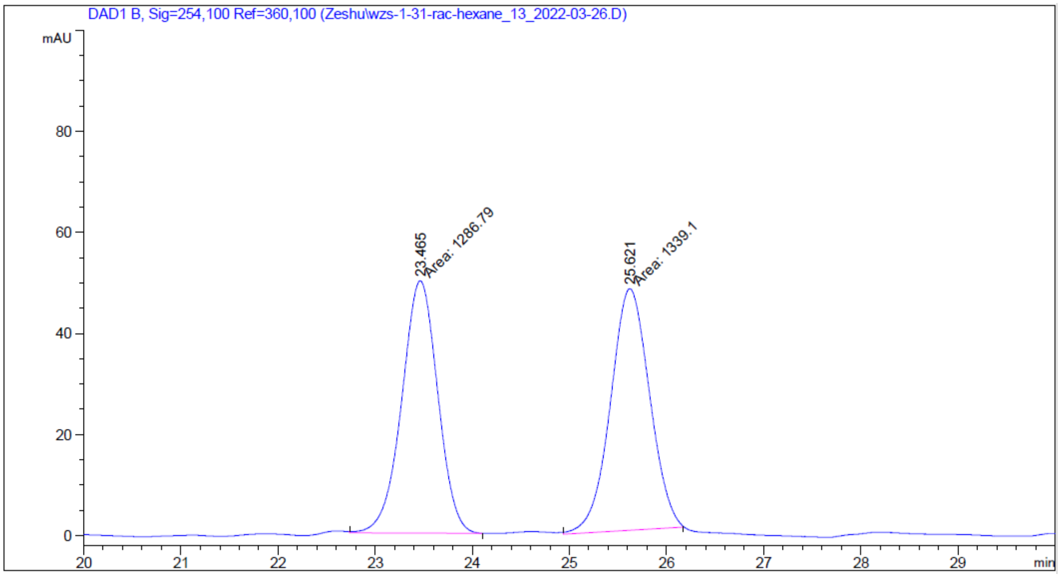

Peak #	RetTime [min]	Type	Width [min]	Area [mAU*s]	Height [mAU]	Area %
1	23.465	MM	0.4292	1286.79309	49.97092	49.0040
2	25.621	MM	0.4663	1339.10303	47.86012	50.9960

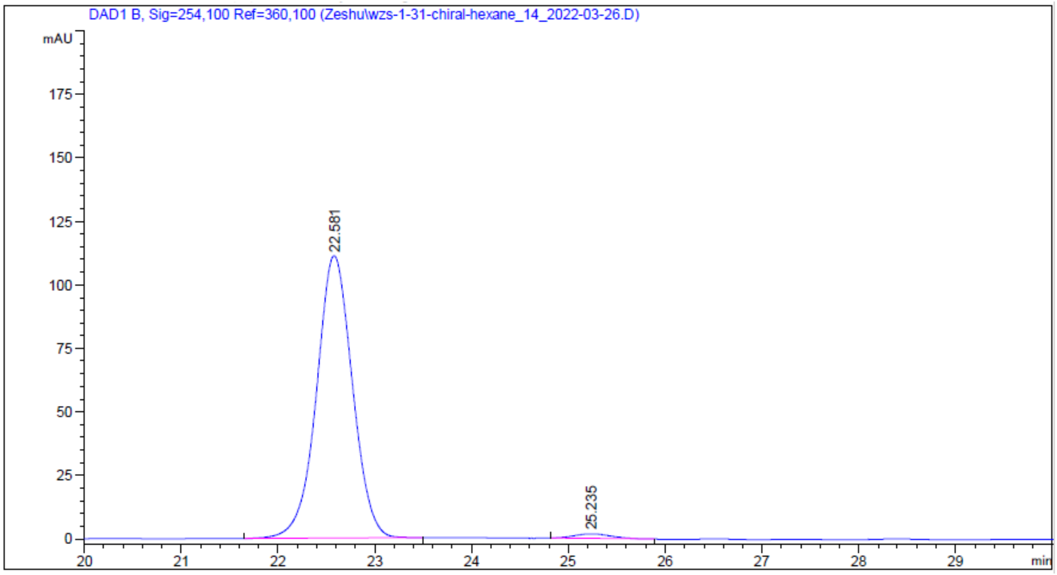

Peak #	RetTime [min]	Type	Width [min]	Area [mAU*s]	Height [mAU]	Area %
1	22.581	BB	0.3848	2866.94287	111.19862	98.3451
2	25.235	BB	0.3109	48.24269	1.82823	1.6549

(2*S*,3*S*)-3-((*tert*-Butyldimethylsilyl)oxy)-2-methyl-5-(triethylsilyl)pent-4-yn-1-yl trifluoromethanesulfonate (46)

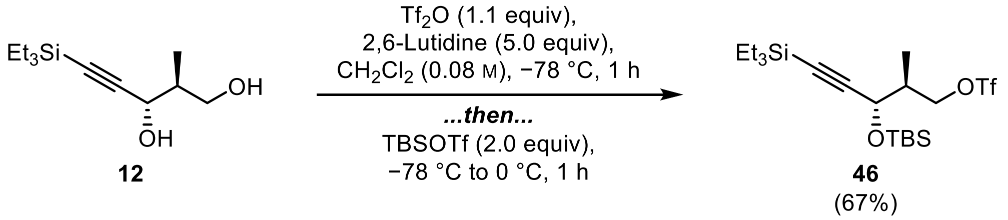

To a solution of diol **12** (2.28 g, 10.0 mmol, 1.00 equiv) and 2,6-lutidine (5.8 mL, 50 mmol, 5.00 equiv) in anhydrous CH_2Cl_2 (100 mL) at −78° C was added trifluoromethanesulfonic anhydride (Tf_2O, 1 M in CH_2Cl_2, 11.0 mL, 11 mmol, 1.10 equiv)[A] in anhydrous CH_2Cl_2 (28 mL) dropwise by syringe pump (rate: 1 mL/min). The reaction mixture was then stirred at this temperature for 1 h before *tert*-butyldimethylsilyl trifluoromethanesulfonate (TBSOTf, 4.6 mL, 20.0 mmol, 2.00 equiv)[A] was added slowly dropwise (rate: 1 mL/min). Following this, the reaction was warmed to 0 °C and stirred for 1 h before quenching with saturated aqueous NH_4Cl (15 mL) and transferring to a separating funnel containing saturated aqueous NH_4Cl (125 mL), washing the transferring flask with CH_2Cl_2 (15 mL). The aqueous phase was then extracted with CH_2Cl_2 (3 × 125 mL) and the organic phases were combined, washed with brine (125 mL), dried with Na_2SO_4, filtered, and concentrated under reduced pressure to provide a crude residue. The crude product was purified by flash column chromatography (0–4% EtOAc in *n*-hexane) to give triflate **46** (3.20 g, 67%) as a colourless oil.

Notes: (**A**) To ensure high yields, recently purchased/unopened bottles of Tf_2O and TBSOTf work best for this transformation.

TLC: R_f = 0.58 (4% EtOAc in *n*-hexane)

^{1}H NMR (500 MHz, Benzene-d_6) δ 4.42 (dd, J = 9.7, 4.9 Hz, 1H), 4.31 (dd, J = 9.7, 5.9 Hz, 1H), 4.17 (d, J = 6.3 Hz, 1H), 1.85 (app. hept, J = 6.3 Hz, 1H), 1.03 (t, J = 8.0 Hz, 9H), 0.93 (s, 9H), 0.87 (d, J = 6.9 Hz, 3H), 0.59 (q, J = 8.0 Hz, 6H), 0.18 (s, 3H), 0.07 (s, 3H) ppm

^{13}C NMR (126 MHz, Benzene-d_6) δ 119.4 (q, J = 319.7 Hz), 106.3, 88.8, 78.4, 64.5, 40.8, 25.9, 18.3, 12.4, 7.7, 4.6, −4.3, −5.2 ppm

^{19}F NMR (377 MHz, Benzene-d_6) δ −75.1 (s) ppm

HRMS (*m/z*): (ESI) calculated for $C_{19}H_{37}F_3NaO_4SSi_2$ $[M+Na]^+$: 497.1795, found: 497.1796

IR (thin film) ν_{max}: 2957, 2934, 2878, 2860, 2173, 1464, 1416, 1390, 1362, 1337, 1246, 1202, 1146, 1090, 1020, 1006, 983, 950, 930, 837, 812, 779 and 724 cm^{-1}

$[\alpha]_D^{23}$**:** –27.0 (*c* = 1.00, $CHCl_3$)

***tert*-Butyldimethyl(((3*S*,4*S*)-4-methyl-1-(triethylsilyl)octa-1,6-diyn-3-yl)oxy)silane (13)**

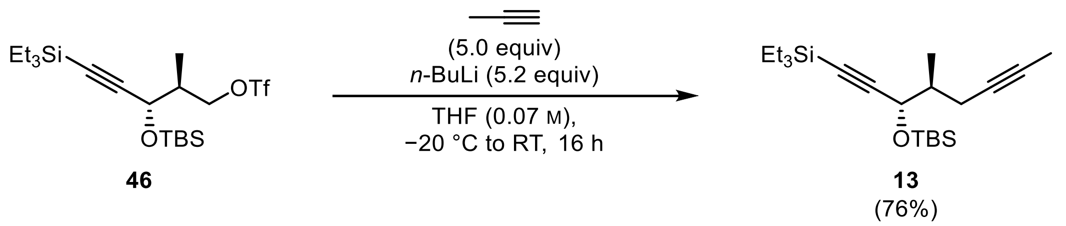

Propyne (2.55 mL, 33.7 mmol, 5.00 equiv) was condensed into a flame dried Schlenk flask at −78 °C and diluted with anhydrous THF (45 mL). To this solution was added *n*-butyllithium (21.9 mL, 35.1 mmol, 5.20 equiv, 1.60 M in hexanes) slowly dropwise (rate: 1 mL/min) and the reaction was stirred at this temperature for 2 h. The cloudy-white suspension produced was then warmed to –20 °C and a solution of triflate **46** (3.20 g, 6.74 mmol, 1.00 equiv) in anhydrous THF (45 mL) was added slowly dropwise (rate: 1 mL/min). Following this, the reaction was warmed to room temperature and stirred for 16 h. The reaction was then cooled to 0 °C and carefully quenched with water (15 mL). The solution was then transferred to a separating funnel containing H_2O (125 mL) and extracted with Et_2O (3 × 125 mL). The organic phases were combined, washed with brine (125 mL), dried over Na_2SO_4, filtered, and concentrated under reduced pressure. The crude residue was purified by flash column chromatography (0%–1% EtOAc in *n*-hexane) to give di-alkyne **13** (1.86 g, 76%) as a colourless oil.

TLC: R_f = 0.44 (100% *n*-heptane)

^{1}H NMR (500 MHz, Benzene-d_6) δ 4.49 (d, J = 6.7 Hz, 1H), 2.47 – 2.36 (m, 2H), 2.03 (app. hept, J = 6.7 Hz, 1H), 1.52 (s, 3H), 1.25 (d, J = 6.7 Hz, 3H), 1.06 (t, J = 7.9 Hz, 9H), 1.01 (s, 9H), 0.60 (q, J = 7.9 Hz, 6H), 0.28 (s, 3H), 0.19 (s, 3H) ppm

^{13}C NMR (126 MHz, Benzene-d_6) δ 108.3, 87.4, 77.4, 77.2, 67.2, 40.6, 26.1, 22.5, 18.5, 15.5, 7.8, 4.8, 3.3, −4.2, −4.9 ppm

HRMS (*m/z*): (MALDI) calculated for $C_{21}H_{40}NaOSi_2$ $[M+Na]^+$: 387.2510, found: 387.2502

IR (thin film) ν_{max}: 2956, 2930, 2876, 2858, 2169, 1462, 1416, 1378, 1361, 1251, 1079, 1018, 981, 938, 836, 777, 725 and 673 cm^{-1}

$[\alpha]_D^{18}$**:** –24.0 (*c* = 1.00, $CHCl_3$)

***tert*-Butyldimethyl(((3*S*,4*S*)-4-methylocta-1,6-diyn-3-yl)oxy)silane (47)**

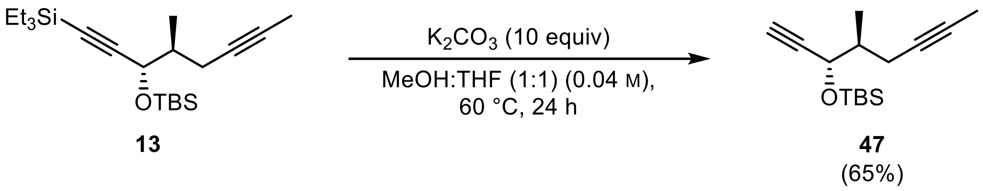

To a solution of di-alkyne **13** (1.86 g, 5.10 mmol, 1.00 equiv) in THF/MeOH (120 mL, 1:1, 0.04 M) at room temperature was added K_2CO_3 (7.05 g, 51.0 mmol, 10.0 equiv) and the reaction was placed into a pre-heated oil bath at 60 °C and stirred for 24 h. The reaction was then cooled to room temperature and the solvents were removed under reduced pressure. Water (100 mL) was added, and the crude material was extracted with EtOAc (3 × 100 mL). The organic phases were then combined, washed with brine (100 mL), dried over Na_2SO_4, filtered, and concentrated under reduced pressure. The crude residue obtained was purified by column chromatography (0%–1% EtOAc in *n*-hexane) to give terminal alkyne **47** (829 mg, 65%) as a colourless oil.

TLC: R_f = 0.43 (*n*-hexane)

^{1}H NMR (400 MHz, Chloroform-*d*) *δ* 4.32 (dd, *J* = 6.4, 2.1 Hz, 1H), 2.37 (d, *J* = 2.1 Hz, 1H), 2.30 – 2.22 (m, 1H), 2.21 – 2.14 (m, 1H), 1.89 – 1.83 (m, 1H), 1.77 (t, *J* = 2.6 Hz, 3H), 1.06 (d, *J* = 6.8 Hz, 3H), 0.90 (s, 9H), 0.14 (s, 3H), 0.11 (s, 3H) ppm

^{13}C NMR (101 MHz, Chloroform-*d*) *δ* 84.2, 77.3, 76.9, 73.3, 66.0, 40.1, 25.9, 22.0, 18.3, 15.1, 3.6, –4.5, –5.1 ppm

Note: Two alkyne peaks in the ^{13}C NMR reside in between the chloroform peak.

HRMS (*m/z*): (ESI) calculated for $C_{15}H_{27}OSi$ $[M+H]^+$: 251.1826, found: 251.1820

IR (thin film) ν_{max}: 3310, 2957, 2929, 2857, 1472, 1462, 1361, 1252, 1081, 1028, 1006, 836 and 777 cm^{-1}

$[\alpha]_D^{18}$**:** –24.0 (*c* = 1.00, $CHCl_3$)

tert-Butyl((3_S_,4_S_,_E_)-1-iodo-4-methyloct-1-en-6-yn-3-yloxy)dimethylsilane (6)

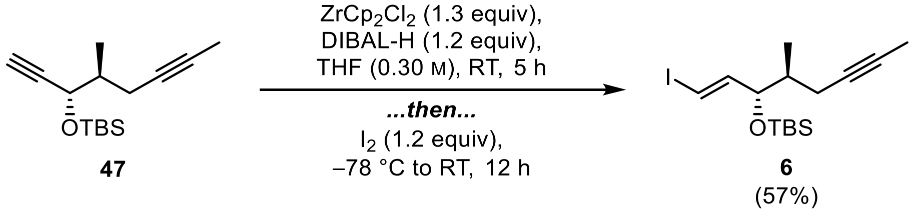

To a stirring suspension of bis(cyclopentadienyl)zirconium(IV) dichloride (Cp_2ZrCl_2, 623 mg, 2.14 mmol, 1.30 equiv) in anhydrous THF (3.60 mL) at 0 °C was added diisobutylaluminum hydride (DIBAL-H, 1.0 M in hexanes, 1.98 mL, 1.98 mmol, 1.20 equiv) slowly dropwise. The resulting suspension was stirred at this temperature for 1 h before a solution of alkyne **47** (413 mg, 1.65 mmol, 1.00 equiv) in anhydrous THF (1.5 mL) was added dropwise. The reaction was then warmed to room temperature and stirred for 5 h, during which time it became a yellow solution. The reaction was then cooled down to −78 °C and a solution of iodine (503 mg, 1.98 mmol, 1.20 equiv) in THF (1.5 mL) was added slowly dropwise. The mixture was then allowed to gently warm from −78 °C to room temperature overnight (the cooling bath was not removed). The reaction was then cooled to 0 °C, quenched with water (5 mL) and stirred for 15 min before the crude material was poured into a separating funnel containing a mixture of saturated aqueous $Na_2S_2O_3$ and saturated aqueous $NaHCO_3$ solution (20 mL, 2:1). The aqueous phase was extracted with EtOAc (3 × 25 mL) and the organic phases were combined, washed with brine (25 mL), dried over $MgSO_4$, filtered, and concentrated under reduced pressure. The crude residue obtained was purified by column chromatography (100% _n_-hexane) to give vinyl iodide **6**[A] (354 mg, 57%) as a colourless oil.

Notes: (**A**) Vinyl iodide **6** is light-sensitive. Therefore, the reaction flask was covered in aluminium foil for the duration of the reaction and was handled in a fume hood with the light off.

TLC: R_f = 0.33 (_n_-hexane)

^{1}H NMR (400 MHz, Benzene-d_6) δ 6.41 (dd, J = 14.5, 7.1 Hz, 1H), 6.02 (dd, J = 14.5, 0.8 Hz, 1H), 3.85 (t, J = 6.7 Hz, 1H), 2.17 – 2.09 (m, 3H), 1.64 – 1.59 (m, 1H), 1.57 (t, J = 2.6 Hz, 3H), 0.91 (s, 9H), 0.86 (d, J = 6.9 Hz, 3H), 0.02 (s, 3H), −0.01 (s, 3H) ppm

^{13}C NMR (101 MHz, Benzene-d_6) δ 147.7, 78.5, 77.6, 77.3, 77.1, 39.2, 26.0, 22.2, 18.4, 15.3, 3.4, −4.2, −4.9 ppm

HRMS (*m/z*): (ESI) calculated for $C_{15}H_{27}INaOSi$ $[M+Na]^+$: 401.0768, found: 401.0777

IR (thin film) ν_{max}: 2955, 2928, 2856, 1607, 1471, 1462, 1361, 1253, 1166, 1064, 1029, 951, 835 and 775 cm^{-1}

$[\alpha]_D^{18}$**:** +6.0 (c = 0.82, $CHCl_3$)

3.3. Synthesis of Beraprost

(3a*R*,4*R*,5*R*,6a*S*)-4-((3*S*,4*S*,*E*)-3-(*tert*-Butyldimethylsilyloxy)-4-methyloct-1-en-6-ynyl)-5-hydroxyhexahydro-2*H*-cyclopenta[*b*]furan-2-one (49)

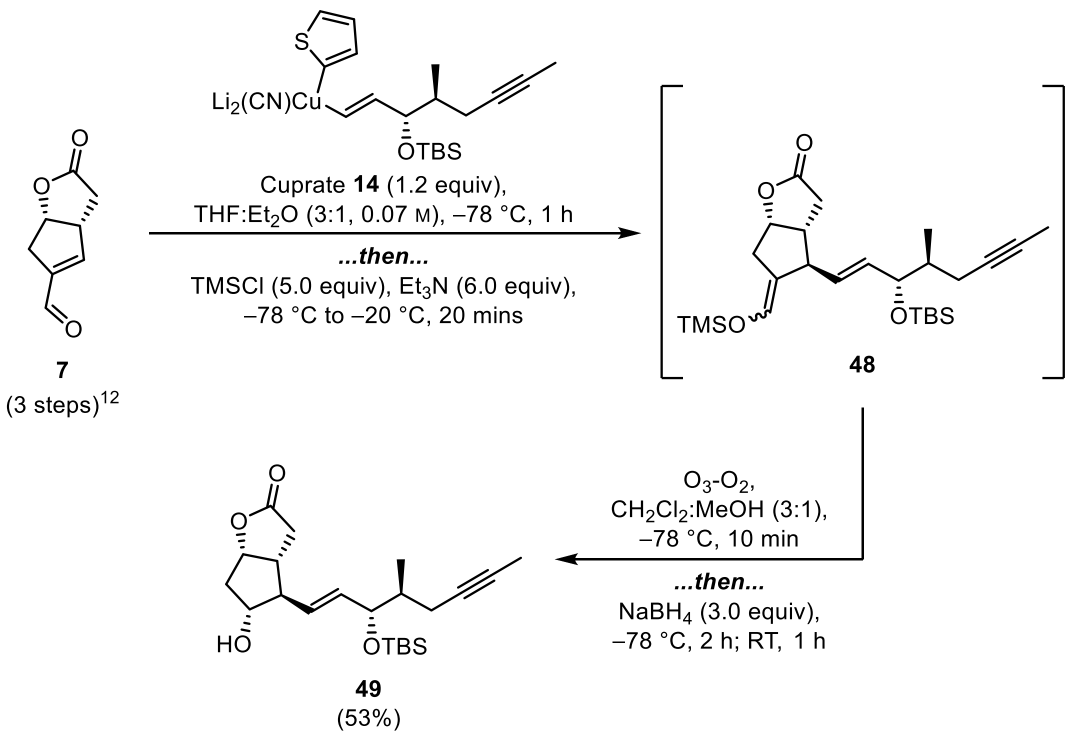

To a solution of vinyl iodide **6** (355 mg, 0.94 mmol, 1.20 equiv) in anhydrous Et_2O (2.80 mL) at –78 °C was added *tert*-butyllithium (1.17 mL, 1.88 mmol, 2.40 equiv, 1.61 M in *n*-pentane) slowly dropwise and the resultant pale-yellow solution was stirred at –78 °C for 2 h before warming to –40 °C for a further 2 h.

During this time, a solution of thiophene (75 μL, 0.94 mmol, 1.20 equiv) in anhydrous THF (2.80 mL) was prepared and cooled to –40 °C before *n*-butyllithium (0.60 mL, 0.94 mmol, 1.20 equiv, 1.56 M in hexanes) was added slowly dropwise. The resultant dark yellow solution was stirred at this temperature for 45 min before cooling to –78 °C and adding copper(I) cyanide (84.1 mg, 0.94 mmol, 1.20 equiv) in a single portion and warming to RT. The thienyl cuprate solution was then added to the above vinyl lithium solution at –78 °C and anhydrous THF (2.80

mL) was added (used for washing the transferring flask). The bright yellow reaction mixture was then warmed to –20 °C for 1 h before re-cooling back to –78 °C once again.

A solution of lactone **7**[12] (119 mg, 0.78 mmol, 1.0 equiv) in anhydrous THF (2.80 mL) was then added dropwise to the pre-formed cuprate **14** at –78 °C to give a bright orange reaction mixture, which was stirred at –78 °C for 1 h before trimethylsilyl chloride (0.50 mL, 3.91 mmol, 5.00 equiv) and triethylamine (0.65 mL, 4.69 mmol, 6.00 equiv) were added sequentially. The reaction was then warmed to –20 °C and stirred for 20 min before quenching with saturated aqueous NH_4Cl solution (20 mL) and warming to RT. The aqueous phase was extracted with EtOAc (3 × 20 mL) and the organic phases combined, washed with saturated aqueous NH_4Cl (20 mL), washed with brine, dried over $MgSO_4$, filtered, and concentrated under reduced pressure to give crude **48** as a brown oil, which was used directly in the next step.

Crude **48** was dissolved in anhydrous CH_2Cl_2/MeOH (6.00 mL, 3:1) and cooled to –78 °C before a stream of O_3–$O_{2(g)}$ was gently bubbled through the solution. The reaction's progress was carefully monitored by TLC (100% CH_2Cl_2) until a small amount of starting **48** remained (~10.0 min). The reaction was then purged by bubbling a gentle stream of $N_{2(g)}$ through the solution for 20 min, which was followed by the portion wise addition of freshly ground $NaBH_4$ (88.8 mg, 2.35 mmol, 3.00 equiv). The mixture was stirred at –78 °C for 2 h followed by 1 h at RT before quenching with brine (15 mL) and extracting with EtOAc (3 × 15 mL). The organic phases were combined, dried over $MgSO_4$, filtered, and concentrated under reduced pressure to give a crude material that was then purified by column chromatography (5–10% EtOAc in CH_2Cl_2) to give alcohol **49** as a colourless oil (164 mg, 53%).

TLC: R_f = 0.28 (10% EtOAc in CH_2Cl_2)

^{1}H NMR (400 MHz, Chloroform-*d*) δ 5.56 (dd, *J* = 15.6, 6.3 Hz, 1H), 5.43 (dd, *J* = 15.6, 7.9 Hz, 1H), 4.93 (td, *J* = 7.0, 2.7 Hz, 1H), 4.04 – 3.97 (m, 2H), 2.75 (dd, *J* = 18.0, 9.8 Hz, 1H), 2.61 (ddd, *J* = 9.7, 8.4, 1.9 Hz, 1H), 2.50 – 2.43 (m, 2H), 2.39 – 2.34 (m, 1H), 2.20 – 2.13 (m, 1H), 2.12 – 2.04 (m, 1H), 1.99 (ddd, *J* = 14.8, 6.8, 2.7 Hz, 1H), 1.78 – 1.77 (m, 4H), 1.72 – 1.63 (m, 1H), 0.92 – 0.87 (m, 12H), 0.05 (s, 3H), –0.01 (s, 3H) ppm

^{13}C NMR (101 MHz, Chloroform-*d*) δ 176.9, 135.2, 129.6, 82.8, 77.9, 77.5, 76.8, 75.8, 56.4, 42.8, 40.2, 39.5, 34.5, 26.0, 21.9, 18.3, 15.7, 3.6, –3.9, –4.7 ppm

Note: The alkyne peak (C≡CMe) and the peak of the carbon attached to the unprotected alcohol in the ^{13}C NMR reside below the chloroform-d peaks and were assigned using 2D NMR.

HRMS (*m/z*): (ESI) calculated for $C_{22}H_{36}NaO_4Si$ $[M+Na]^+$: 415.2275, found: 415.2281

IR (thin film) ν_{max}: 3451, 2955, 2928, 2894, 2856, 1768, 1471, 1360, 1250, 1173, 1079, 1031, 973, 835 and 775 cm^{-1}

$[\alpha]_D^{25}$: +6.2 (c = 0.27, $CHCl_3$)

X-Ray Crystal Structure of Lactone 7: CCDC Number 2172054:

Table 1: Crystal data and structure refinement for **7**.

Empirical formula	$C_8H_8O_3$
Formula weight	152.14
Temperature/K	100(2)
Crystal system	orthorhombic
Space group	$P2_12_12_1$
a/Å	6.9614(2)
b/Å	8.8105(2)
c/Å	11.5184(3)
α/°	90
β/°	90
γ/°	90
Volume/Å^3	706.46(3)
Z	4
ρ_{calc}g/cm^3	1.430
μ/mm^{-1}	0.927
F(000)	320.0
Crystal size/mm^3	0.278 × 0.101 × 0.021
Radiation	CuKα (λ = 1.54178)
2θ range for data collection/°	12.648 to 145.108
Index ranges	$-8 \le h \le 8$, $-10 \le k \le 10$, $-13 \le l \le 14$
Reflections collected	7307
R_{int} / R_{sigma}	1399 [R_{int} = 0.0404, R_{sigma} = 0.0306]
Data/restraints/parameters	1399/0/100
Goodness-of-fit on F^2	1.077
Final R indexes [I>=2σ (I)]	R_1 = 0.0411, wR_2 = 0.1147
Final R indexes [all data]	R_1 = 0.0434, wR_2 = 0.1178

Largest diff. peak/hole / e Å^{-3} 0.20/-0.30

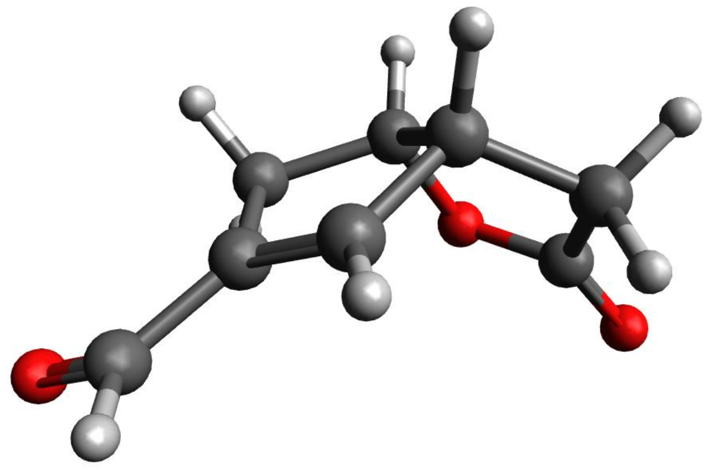

Figure 2: Illustration of structure **7**.

(3a*R*,4*R*,5*R*,6a*S*)-5-(*tert*-Butyldimethylsilyloxy)-4-((3*S*,4*S*,*E*)-3-(*tert*-butyldimethylsilyloxy)-4-methyloct-1-en-6-ynyl)hexahydro-2*H*-cyclopenta[*b*]furan-2-one (15)

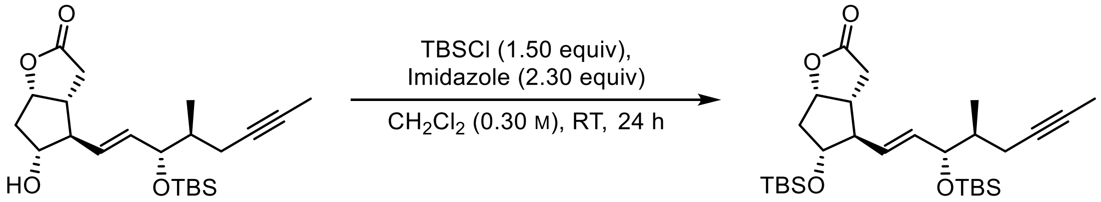

To a solution of alcohol **49** (164 mg, 0.42 mmol, 1.00 equiv) in anhydrous CH_2Cl_2 (1.40 ml, 0.30 M) at 0 °C was added imidazole (65.4 mg, 0.96 mmol, 2.30 equiv) followed by *tert*-butyldimethylsilyl chloride (TBSCl, 94.4 mg, 0.63 mmol, 1.50 equiv). The reaction was stirred at this temperature for 15 mins before warming to room temperature and stirring for 24 h. Following this, the mixture was poured into a separating funnel containing water (15 mL) and the aqueous phase was extracted with CH_2Cl_2 (3 × 15 mL). The organic phases were combined, washed with brine (15 mL), dried over Na_2SO_4, filtered, and concentrated under reduced pressure. The crude residue was purified by column chromatography (5–10% EtOAc in *n*-hexane) to give protected alcohol **15** (165 mg, 78%) as a white, amorphous solid.

MP: 62 – 66 °C

TLC: R_f = 0.22 (10% EtOAc in *n*-hexane)

^{1}H NMR (400 MHz, Chloroform-*d*) δ 5.45 (dd, *J* = 15.5, 5.7 Hz, 1H), 5.38 (dd, *J* = 15.6, 6.7 Hz, 1H), 4.96 (td, *J* = 7.0, 1.9 Hz, 1H), 4.02 – 3.94 (m, 2H), 2.76 (dd, *J* = 17.7, 10.5 Hz, 1H), 2.69 – 2.62 (m, 1H), 2.53 – 2.46 (m, 2H), 2.25 – 2.18 (m, 1H), 2.17 – 2.12 (m, 1H), 2.11 – 2.03 (m, 1H), 1.99 (dd, *J* = 14.7, 2.8 Hz, 1H), 1.77 (t, *J* = 2.5 Hz, 3H), 1.70 – 1.61 (m, 1H), 0.89 – 0.89 (m, 21H), 0.05 (s, 6H), 0.04 (s, 3H), –0.02 (s, 3H) ppm

^{13}C NMR (101 MHz, Chloroform-*d*) δ 177.2, 133.5, 130.5, 83.7, 78.3, 77.8, 76.7, 76.0, 57.1, 42.5, 40.8, 39.7, 35.3, 26.0, 25.9, 22.1, 18.3, 18.1, 15.6, 3.6, –3.9, –4.6, –4.7, –4.8 ppm

HRMS (*m/z*): (ESI) calculated for $C_{28}H_{50}NaO_4Si_2$ $[M+Na]^+$: 529.3140, found: 529.3132

IR (thin film) ν_{max}: 2953, 2929, 2894, 2856, 1750, 1471, 1361, 1249, 1164, 1112, 1030, 969, 913, 834 and 774 cm^{-1}

$[\alpha]_D^{20}$: –16.0 (*c* = 1.00, $CHCl_3$)

***tert*-Butyl(((3*S*,4*S*,*E*)-1-((3a*R*,4*R*,5*R*,6a*S*)-5-((*tert*-butyldimethylsilyl)oxy)-3a,5,6,6a-tetrahydro-4*H*-cyclopenta[*b*]furan-4-yl)-4-methyloct-1-en-6-yn-3-yl)oxy)dimethylsilane (5)**

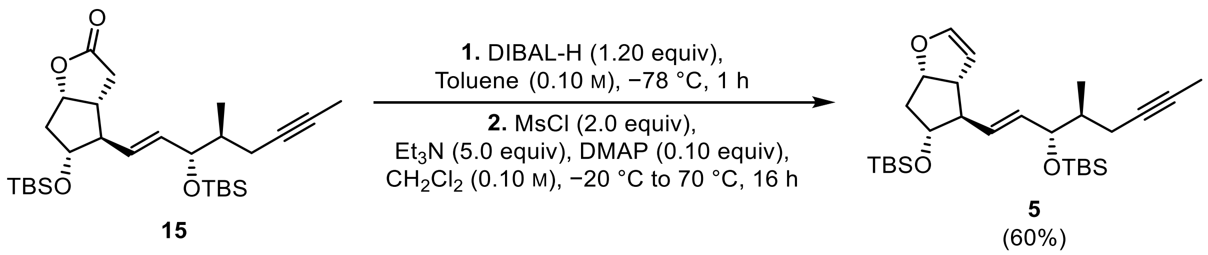

To a solution of lactone **15** (165 mg, 0.33 mmol, 1.00 equiv) in anhydrous toluene (3.26 mL, 0.10 M) at −78 °C was added DIBAL-H (1 M in toluene, 0.39 mL, 0.39 mmol, 1.20 equiv) slowly dropwise and the reaction was stirred at this temperature for 1 h. Following this, the cooling bath was removed, and the reaction was immediately quenched with saturated aqueous Rochelle's salt (7.5 mL). The solution was allowed to warm to room temperature and stirred for 2 h before transferring to a separating funnel, washing the flask with H_2O (7.5 mL), and extracting with CH_2Cl_2 (4 × 15 mL). The organic phases were combined, washed with brine (15 mL), dried with Na_2SO_4, filtered, and concentrated under reduced pressure to afford a crude colourless oil.

This crude material was dissolved in anhydrous CH_2Cl_2 (3.30 mL) and transferred to a flame-dried microwave vial under an atmosphere of nitrogen. The flask was cooled to 0 °C and Et_3N (0.23 mL, 1.63 mmol, 5.0 equiv) was added followed by DMAP (4.00 mg, 0.03 mmol, 0.10 equiv). The solution was cooled to −20 °C and methanesulfonyl chloride (MsCl, 50.0 μL, 0.65 mmol, 2.0 equiv) was added dropwise and the reaction was stirred at this temperature for 20 min. The mixture was then allowed to warm to room temperature before the microwave vial was capped and the reaction placed into a pre-heated oil bath at 70 °C for 16 h. The reaction mixture was then cooled to room temperature and transferred to a separating funnel, washing the transferring flask with CH_2Cl_2 (10 mL). The organic phase was washed with saturated aqueous NH_4Cl (2 × 15 mL), dried with Na_2SO_4, filtered, and concentrated under reduced pressure. The crude residue obtained was purified by flash column chromatography (0–10% Et_2O in *n*-hexane) to afford enol ether **5** (96 mg, 60%) as a colourless oil.

TLC: R_f = 0.51 (5% Et_2O in *n*-hexane)

^{1}H NMR (500 MHz, Benzene-d_6) δ 6.17 (dd, *J* = 2.7, 1.8 Hz, 1H), 5.52 (dd, *J* = 15.6, 6.8 Hz, 1H), 5.47 (dd, *J* = 15.6, 5.8 Hz, 1H), 4.92 (t, *J* = 2.7 Hz, 1H), 4.59 (ddd, *J* = 10.0, 7.4, 5.7 Hz, 1H), 4.08 (t, *J* = 6.0 Hz, 1H), 3.59 (ddd, *J* = 8.8, 8.0, 6.0 Hz, 1H), 2.74 (ddt, *J* = 10.1, 8.1, 2.2 Hz, 1H), 2.42 – 2.21 (m, 4H), 1.92 (ddd, *J* = 13.1, 8.9, 5.7 Hz, 1H), 1.84 (qd, *J* = 7.0, 5.6 Hz, 1H), 1.60 (t, *J* = 2.6 Hz, 3H), 1.08 (d, *J* = 6.8 Hz, 3H), 1.02 (s, 9H), 0.96 (s, 9H), 0.14 (s, 3H), 0.12 (s, 3H), 0.07 (s, 3H), 0.02 (s, 3H) ppm

^{13}C NMR (126 MHz, Benzene-d_6) δ 145.3, 132.6, 132.4, 103.2, 82.9, 78.2, 76.8, 76.6, 76.6, 58.9, 50.3, 43.1, 40.2, 26.2, 26.1, 22.6, 18.5, 18.2, 15.8, 3.4, −3.6, −4.3, −4.5, −4.6 ppm

Note: The alkyne peak (C≡CMe*) overlaps with the carbon attached to the secondary alcohol on the ω-sidechain in the ^{13}C NMR spectrum and has been assigned using 2D data.*

HRMS (*m/z*): (ESI) calculated for $C_{28}H_{51}O_3Si_2$ $[M+H]^+$: 491.3371, found: 491.3353

IR (thin film) ν_{max}: 2956, 2929, 2903, 2857, 1611, 1472, 1364, 1254, 1128, 1061, 1006, 866, 836, 775, 713 and 672 cm^{-1}

$[\alpha]_D^{23}$**:** +14.0 (*c* = 1.00, CH_2Cl_2)

4-((1*R*,2*R*,3a*S*,4a*R*,8a*S*,8b*S*)-2-((*tert*-butyldimethylsilyl)oxy)-1-((3*S*,4*S*,*E*)-3-((*tert*-butyldimethylsilyl)oxy)-4-methyloct-1-en-6-yn-1-yl)-8-chloro-2,3,3a,4a,8a,8b-hexahydro-1*H*-cyclopenta[*b*]benzofuran-5-yl)butanoic acid (3)

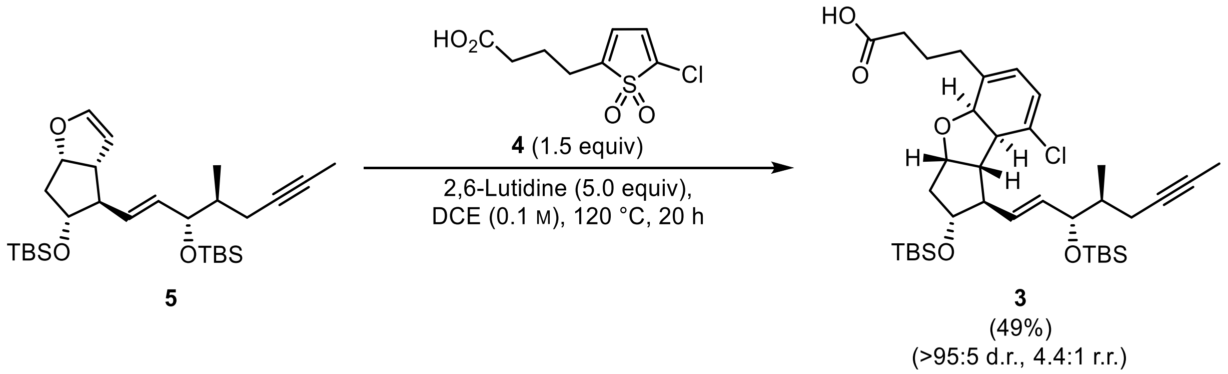

To a flame-dried microwave vial containing a solution of thiophene 1,1-dioxide **4** (60.0 mg, 0.25 mmol, 1.5 equiv) in anhydrous 1,2-dichloroethane (DCE, 0.85 mL) was added 2,6-lutidine (0.10 mL, 0.85 mmol, 5.0 equiv) and the solution was stirred for 5 minutes. A solution of enol ether **5** (83.0 mg, 0.17 mmol, 1.00 equiv) in anhydrous DCE (0.85 mL) was then added to the mixture. The microwave vial was capped and placed into a pre-heated oil bath at 120 °C and stirred for 16 h. Following this, the reaction was allowed to cool to room temperature before it was diluted with CH_2Cl_2 (5 mL) followed by addition of 1 M hydrochloric acid (5 mL). The aqueous phase was extracted with CH_2Cl_2 (3 × 5 mL) and the organic phases were combined, washed with brine (5 mL), dried with Na_2SO_4, filtered, and concentrated under reduced pressure. The crude residue obtained was purified by flash column chromatography (20–50% EtOAc in *n*-hexane) to afford chloro-diene **3** (55 mg, 49%, >95:5 d.r., 4.4:1 r.r.) as a yellow oil. Further iterations of flash column chromatography using the above conditions was used to fully separate the regioisomers for characterisation.

TLC: R_f = 0.40 (50% EtOAc in *n*-hexane)

^{1}H NMR (500 MHz, Chloroform-*d*) δ 5.99 (dd, *J* = 6.3, 2.0 Hz, 1H), 5.76 (d, *J* = 6.3 Hz, 1H), 5.58 – 5.53 (m, 2H), 4.53 (d, *J* = 7.7 Hz, 1H), 4.31 (td, *J* = 7.3, 4.4 Hz, 1H), 4.06 – 4.00 (m, 1H), 3.85 (td, *J* = 7.7, 6.5 Hz, 1H), 2.93 (dt, *J* = 7.7, 2.0 Hz, 1H), 2.83 (ddd, *J* = 9.1, 7.3, 2.0 Hz, 1H), 2.43 – 2.36 (m, 2H), 2.37 – 2.23 (m, 4H), 2.24 – 2.17 (m, 1H), 2.12 – 2.02 (m, 1H), 1.87 (dq, *J* = 14.2, 7.3 Hz, 2H), 1.78 (t, *J* = 2.5 Hz, 3H), 1.73 – 1.63 (m, 2H), 0.92 (d, *J* = 6.8 Hz, 3H), 0.89 (s, 9H), 0.89 (s, 9H), 0.06 – 0.05 (m, 9H), 0.02 (s, 3H) ppm

^{13}C NMR (126 MHz, Chloroform-*d*) δ 178.3, 135.2, 134.1, 133.1, 131.1, 121.2, 121.1, 78.3, 78.2, 77.4, 77.0, 76.5, 76.1, 55.9, 53.0, 50.3, 41.9, 39.9, 34.1, 33.4, 26.1, 26.0, 22.8, 22.2, 18.3, 18.2, 15.6, 3.6, −3.8, −4.4, −4.6, −4.7 ppm

Note: The carboxylic acid peak (178.3 ppm) as well as the peaks *alpha* (33.4 ppm) and *beta* (22.8 ppm) to the carboxylic acid appear broadened and weak in the ^{13}C NMR and as such, these weaker peaks have been further confirmed using 2D NMR data. These weak and broadened signals nearby the carboxylic acid could be suggestive that **3** exists as a dimer that is hydrogen bonded together through the carboxylic acid. Similar issues with the ^{13}C NMR spectra of the carboxylic acid containing natural products strasseriolide A and B have also been reported by Rychnovsky and co-workers.[13] In their case, they are unable to observe the natural products' carboxylic acid peak or the peak of the carbon *alpha* to the acid by ^{13}C NMR. They propose this is due to the formation of a carboxylic acid hydrogen-bonded dimer. We observe these issues for this compound (**3**) as well as the carboxylic acid containing compounds **16** and **21b**.

HRMS (*m/z*): (ESI) calculated for $C_{36}H_{58}ClO_5Si_2$ $[M{-}H]^-$: 661.3517, found: 661.3499

IR (thin film) ν_{max}: 2955, 2928, 2856, 1709, 1462, 1251, 1118, 1059, 836 and 775 cm^{-1}

$[\alpha]_D^{22}$**:** −12.0 (c = 3.00, $CHCl_3$)

***tert*-Butyl 4-((1*R*,2*R*,3a*S*,4a*R*,8a*S*,8b*S*)-2-((*tert*-butyldimethylsilyl)oxy)-1-((3*S*,4*S*,*E*)-3-((*tert*-butyldimethylsilyl)oxy)-4-methyloct-1-en-6-yn-1-yl)-8-chloro-2,3,3a,4a,8a,8b-hexahydro-1*H*-cyclopenta[*b*]benzofuran-5-yl)butanoate (3a)**

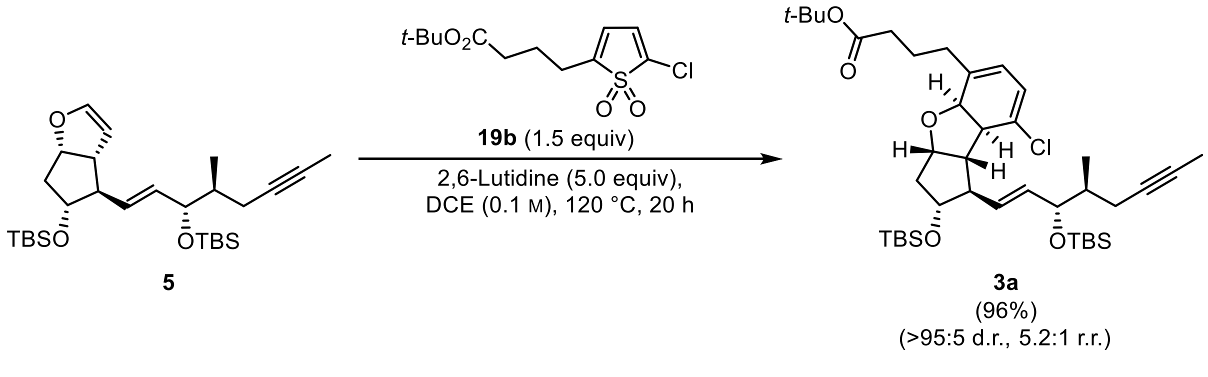

To a flame-dried microwave vial containing a solution of enol ether **5** (30.0 mg, 0.06 mmol, 1.00 equiv) in anhydrous DCE (0.61 mL, 0.10 M) was added thiophene 1,1-dioxide **19b** (26.8 mg, 0.09 mmol, 1.5 equiv) and 2,6-lutidine (36.0 μL, 0.31 mmol, 5.0 equiv). The microwave vial was then capped and placed into a pre-heated oil bath at 120 °C and stirred for 16 h.

Following this, the reaction was allowed to cool to room temperature before it was diluted with CH_2Cl_2 (5 mL) and transferred to a separating funnel containing saturated aqueous NH_4Cl (5 mL). The aqueous phase was extracted with CH_2Cl_2 (3 × 5 mL) and the organic phases were combined, washed with brine (5 mL), dried with Na_2SO_4, filtered, and concentrated under reduced pressure. The crude residue obtained was purified by flash column chromatography (0–5% Et_2O in *n*-hexane) to afford chloro-diene **3a** (42 mg, 96%, >95:5 d.r., 5.2:1 r.r.) as a colourless oil.

TLC: R_f = 0.30 (10% Et_2O in *n*-hexane)

^{1}H NMR (500 MHz, Benzene-d_6) δ 5.94 (d, *J* = 6.3 Hz, 0.16H),* 5.86 (dd, *J* = 6.3, 2.0 Hz, 0.84H), 5.67 – 5.54 (m, 1.70H),* 5.50 – 5.35 (m, 1.20H),* 5.24 (dd, *J* = 6.1, 1.3 Hz, 0.17H),* 4.80 (d, *J* = 9.0 Hz, 0.17H),* 4.37 (d, *J* = 7.9 Hz, 0.83H), 4.21 (m, 1H),* 4.17 – 4.08 (m, 1H),* 3.79 (q, *J* = 6.4 Hz, 0.16H),* 3.62 (td, *J* = 8.1, 6.8 Hz, 0.84H), 2.82 (dd, *J* = 7.9, 2.0 Hz, 0.83H), 2.73 (ddd, *J* = 9.1, 7.0, 1.9 Hz, 1H),* 2.42 (m, 1H),* 2.32 (m, 2H),* 2.26 – 2.05 (m, 5H),* 2.04 – 1.84 (m, 1.77H),* 1.81 (m, 1.68H),* 1.78 – 1.67 (m, 1.35H),* 1.61 (m, 3H),* 1.40 (m, 9H),* 1.12 (m, 3H),* 1.04 (m, 9H),* 0.98 (m, 9H),* 0.20 (s, 2.62H), 0.18 (s, 2.45H), 0.15 (s, 0.48H),* 0.12 (s, 0.45H),* 0.09 (s, 3H),* 0.05 (s, 0.49H),* 0.04 (s, 2.45H) ppm

*Note: Peaks corresponding to the minor regioisomer have been marked with an asterisk**

^{13}C NMR (126 MHz, Benzene-d_6) δ 172.4, 136.3, 133.9, 133.4, 131.7, 121.7, 120.6, 79.5, 78.4, 78.2, 77.7, 77.4, 76.8, 76.4, 56.0, 53.4, 50.4, 42.2, 40.4, 35.0, 34.5, 28.2, 26.2, 26.1, 23.4, 22.7, 18.5, 18.2, 15.8, 3.5, −3.5, −4.2, −4.5, −4.6 ppm

Note: Only peaks corresponding to the major regioisomer have been reported

HRMS (*m/z*): (ESI) calculated for $C_{40}H_{67}NaO_5ClSi_2$ $[M+Na]^+$: 741.4108, found: 741.4111

IR (thin film) ν_{max}: 2957, 2930, 2857, 2893, 1730, 1647, 1594, 1462, 1366, 1253, 1142, 1060, 836, 776 and 671 cm^{-1}

$[\alpha]_D^{22}$**:** −14.0 (*c* = 1.00, CH_2Cl_2)

4-((1*R*,2*R*,3a*S*,8b*S*)-2-((*tert*-butyldimethylsilyl)oxy)-1-((3*S*,4*S*,*E*)-3-((*tert*-butyldimethylsilyl)oxy)-4-methyloct-1-en-6-yn-1-yl)-2,3,3a,8b-tetrahydro-1*H*-cyclopenta[*b*]benzofuran-5-yl)butanoic acid (16)

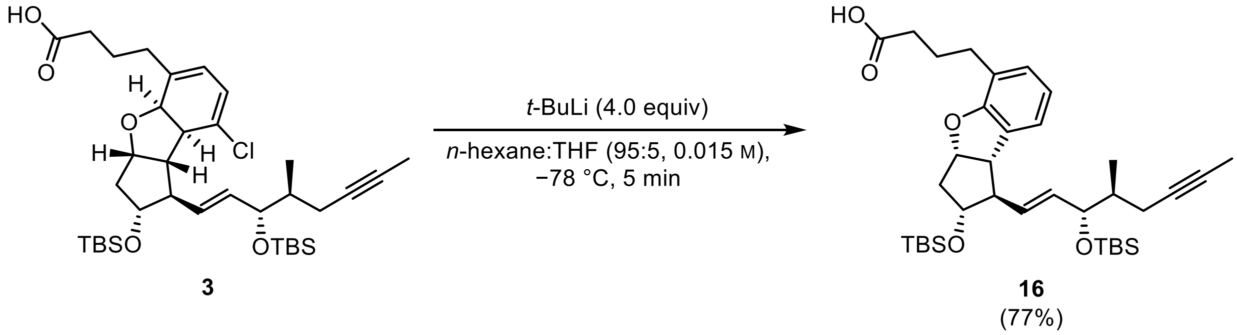

To a flame-dried Schlenk tube containing a solution of chloro-diene **3** (33 mg, 0.05 mmol, 1.0 equiv) in anhydrous *n*-hexane:THF (95:5, 3.30 mL, 0.015 M) was added *tert*-butyllithium (*t*-BuLi, 1.61 M in *n*-pentane, 0.12 mL, 0.20 mmol, 4.0 equiv) at −78 °C slowly dropwise. The reaction was stirred at −78 °C for 5 minutes before quenching with MeOH (0.20 mL) and warming to room temperature. The solvent was removed under reduced pressure and the crude residue was then dissolved in CH_2Cl_2 (5 mL) followed by addition of 1 M hydrochloric acid (5 mL). The aqueous phase was extracted with CH_2Cl_2 (3 × 5 mL) and the organic phases were combined, washed with brine (5 mL), dried with Na_2SO_4, filtered, and concentrated under reduced pressure. The crude residue obtained was purified by flash column chromatography (20–50% EtOAc in *n*-hexane) to afford protected beraprost **16** (24 mg, 77%) as a colourless oil.

TLC: R_f = 0.56 (50% EtOAc in *n*-hexane)

^{1}H NMR (600 MHz, Chloroform-*d*) *δ* 6.98 (d, *J* = 7.3 Hz, 1H), 6.92 (d, *J* = 7.3 Hz, 1H), 6.74 (t, *J* = 7.3 Hz, 1H), 5.60 (dd, *J* = 15.6, 7.4 Hz, 1H), 5.53 (dd, *J* = 15.6, 6.0 Hz, 1H), 5.11 (app. q, *J* = 7.9 Hz, 1H), 4.04 (t, *J* = 6.0 Hz, 1H), 3.94 (td, *J* = 7.2, 5.8 Hz, 1H), 3.47 (app. t, *J* = 7.9 Hz, 1H), 2.69 – 2.32 (m, 6H), 2.23 (ddq, *J* = 16.5, 5.2, 2.5 Hz, 1H), 2.11 (ddt, *J* = 16.5, 7.8, 2.5 Hz, 1H), 2.00 – 1.92 (m, 3H), 1.79 (t, *J* = 2.5 Hz, 3H), 1.72 (app. p, *J* = 6.8 Hz, 1H), 0.96 (d, *J* = 6.8 Hz, 3H), 0.91 (s, 9H), 0.76 (s, 9H), 0.08 (s, 3H), 0.05 (s, 3H), 0.00 (s, 3H), −0.04 (s, 3H) ppm

^{13}C NMR (151 MHz, Chloroform-*d*) *δ* 157.5, 133.0, 131.5, 130.4, 128.6, 123.0, 122.1, 120.3, 85.1, 78.1, 77.3, 76.6, 76.1, 58.3, 50.2, 42.5, 39.9, 29.3, 26.1, 25.8, 24.9, 22.1, 18.3, 18.0, 15.7, 3.6, −3.8, −4.5, −4.7, −4.7 ppm

Note: The carboxylic acid peak and the peaks *alpha* and *beta* to it were not observed in the ^{13}C NMR due to potential dimer formation (as described for compound **3**). However, the carbon *gamma* (29.9 ppm) to the carboxylic acid was observed but appeared weak and as such, HSQC data was used to further confirm this peak. The peak *beta* (24.9 ppm) to the carboxylic acid was also observed by HSQC but not in the ^{13}C NMR (see HSQC data below).

HRMS (*m/z*): (ESI) calculated for $C_{36}H_{58}NaO_5Si_2$ $[M+Na]^+$: 649.3715, found: 649.3735

IR (thin film) ν_{max}: 2926, 2855, 1710, 1459, 1252 and 1104 cm^{-1}

$[\alpha]_D^{24}$**:** +34.7 (*c* = 1.67, $CHCl_3$)

4-((1*R*,2*R*,3a*S*,8b*S*)-2-hydroxy-1-((3*S*,4*S*,*E*)-3-hydroxy-4-methyloct-1-en-6-yn-1-yl)-2,3,3a,8b-tetrahydro-1*H*-cyclopenta[*b*]benzofuran-5-yl)butanoic acid (Beraprost, 1)

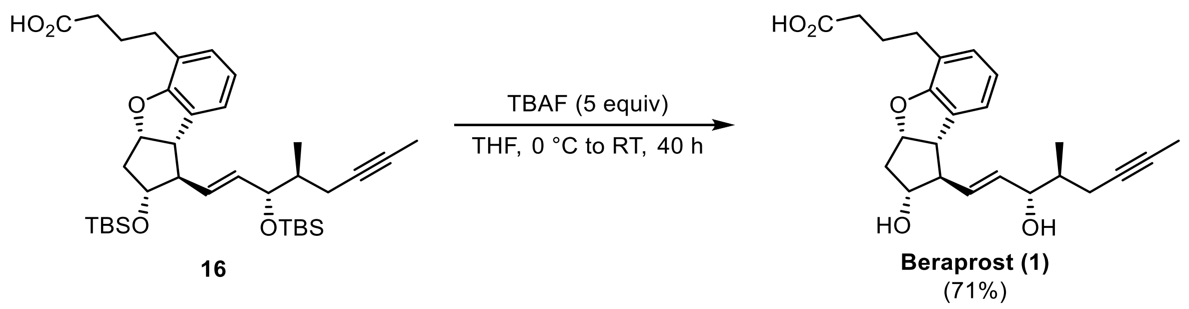

To a solution of protected beraprost **16** (20.0 mg, 0.032 mmol, 1.0 equiv) in anhydrous THF (0.32 mL, 0.10 M) at 0 °C was added TBAF (1.0 M in THF, 0.16 mL, 0.16 mmol, 5.0 equiv) slowly dropwise. The reaction mixture was then warmed to room temperature and stirred for a further 40 h. Following this, the reaction was quenched by addition of H_2O (1.5 mL) and the aqueous phase was extracted with Et_2O (3 × 4 mL). The combined organic phases were washed with brine (4 mL), dried over $MgSO_4$, filtered and concentrated under reduced pressure. The crude residue obtained was purified by preparative TLC (CH_2Cl_2/EtOAc/AcOH = 40:59:1) to afford beraprost **1** (9.0 mg, 71%) as a colourless oil.

TLC: R_f = 0.20 (10% MeOH in DCM)

^{1}H NMR (500 MHz, Chloroform-*d*) δ 6.98 – 6.92 (m, 2H), 6.76 (t, *J* = 7.4 Hz, 1H), 5.67 (dd, *J* = 15.3, 8.5 Hz, 1H), 5.59 (dd, *J* = 15.3, 7.3 Hz, 1H), 5.10 (td, *J* = 8.5, 5.0 Hz, 1H), 4.05 (app. t, *J* = 7.3 Hz, 1H), 3.93 (td, *J* = 8.5, 6.3 Hz, 1H), 3.44 (app. t, *J* = 8.5 Hz, 1H), 2.71 – 2.55 (m, 3H), 2.44 (app. q, *J* = 8.5 Hz, 1H), 2.34 (td, *J* = 7.4, 2.6 Hz, 2H), 2.25 (dt, *J* = 5.4, 2.6 Hz, 2H), 2.03 – 1.88 (m, 3H), 1.80 (t, *J* = 2.6 Hz, 3H), 1.79 – 1.74 (m, 1H), 0.99 (d, *J* = 6.8 Hz, 3H) ppm

^{13}C NMR (126 MHz, Chloroform-*d*) δ 178.0, 157.4, 134.1, 133.0, 129.8, 129.1, 123.3, 122.1, 120.7, 84.7, 77.5, 77.4, 76.6, 76.4, 59.0, 50.6, 41.4, 38.3, 33.1, 29.2, 24.7, 22.4, 16.0, 3.7 ppm

HRMS (*m/z*): (ESI) calculated for $C_{24}H_{29}O_5$ [M−H]$^-$: 397.2020, found: 397.2031

IR (thin film) ν_{max}: 3349, 2958, 2923, 2854, 1706, 1452, 1255, 1193, 1073, 1023, 1002, 972, 763 and 744 cm^{-1}

$[\alpha]_D^{24}$: +44.0 (c = 0.5, EtOH)

Analytical data is consistent with that reported in the literature.[14]

4. Dehalogenation–Aromatization Model Studies

4.1. Synthesis of *tert*-Butyl Protected Brominated Thiophene 1,1-Dioxide

***tert*-Butyl 4-(5-bromothiophen-2-yl)butanoate (50)**

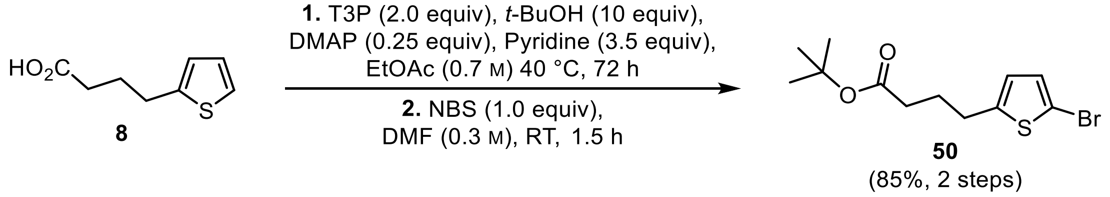

4-(2-Thienyl)butyric acid **8** (2.50 g, 14.7 mmol, 1.00 equiv) was dissolved in EtOAc (12.5 mL) and to this was added pyridine (4.15 mL, 51.5 mmol, 3.50 equiv), DMAP (448 mg, 3.67 mmol, 0.25 equiv) and *t*-BuOH (14.0 mL, 147 mmol, 10.0 equiv). The solution was cooled to 0 °C and a solution of *n*-propylphosphonic anhydride (T3P, ≥50 wt. % in EtOAc, 17.5 mL, 29.4 mmol, 2.00 equiv) was added slowly dropwise (rate: 1 mL/min). The reaction mixture was then placed into a pre-heated oil bath at 40 °C and stirred at this temperature for 72 h. Following this, the reaction was cooled to room temperature, diluted with EtOAc (25 mL) and transferred to a separating funnel containing saturated aqueous $NaHCO_3$ (50 mL). The organic phase was extracted, washed with saturated aqueous NH_4Cl (50 mL), dried with $MgSO_4$, filtered, and concentrated under reduced pressure to afford a crude residue.

The crude material was dissolved in DMF (40 mL, 0.3 M) and *N*-bromosuccinimide (NBS, 2.61 g, 7.34 mmol, 0.5 equiv) was added in one portion and the resulting mixture was stirred for 45 minutes. Following this, a further portion of NBS (2.61 g, 7.34 mmol, 0.5 equiv) was added and the solution was stirred for an additional 45 minutes. The reaction mixture was then diluted with *n*-pentane (40 mL) and water (40 mL) and stirred for 2 hours. The solution was transferred to a separating funnel and the aqueous phase was extracted with *n*-pentane (2 × 40 mL). The

organic phases were combined, dried with $MgSO_4$, filtered, and concentrate under reduced pressure. The crude residue obtained was purified by flash column chromatography (0–50% Et_2O in *n*-pentane) to afford bromo-thiophene **50** (3.83 g, 85% over two steps) as a colourless oil.

TLC: R_f = 0.40 (100% Et_2O in *n*-pentane)

^{1}H NMR (400 MHz, Chloroform-*d*) δ 6.85 (d, *J* = 3.7 Hz, 1H), 6.55 (td, *J* = 3.7, 1.0 Hz, 1H), 2.78 (td, *J* = 7.5, 1.0 Hz, 2H), 2.26 (t, *J* = 7.5 Hz, 2H), 1.91 (p, *J* = 7.5 Hz, 2H), 1.45 (s, 9H) ppm

^{13}C NMR (101 MHz, Chloroform-*d*) δ 172.5, 146.2, 129.6, 125.0, 109.1, 80.5, 34.5, 29.6, 28.2, 26.8 ppm

HRMS (*m/z*): (ESI) calculated for $C_{12}H_{17}BrNaO_2S$ $[M+Na]^+$: 327.005, found: 327.0034

IR (thin film) ν_{max}: 2977, 1728, 1446, 1392, 1367, 1151 and 961 cm^{-1}

***tert*-Butyl 4-(5-bromo-1,1-dioxidothiophen-2-yl)butanoate (19a)**

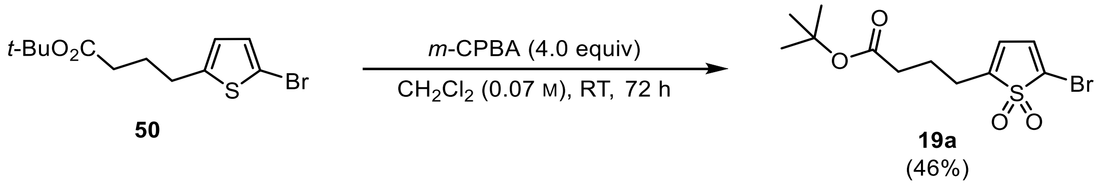

To a solution of bromo-thiophene **50** (801 mg, 2.62 mmol, 1.00 equiv) in CH_2Cl_2 (12 mL) was added a solution of *m*-CPBA (≥70% purity, 2.58 g, 10.5 mmol, 4.00 equiv) in CH_2Cl_2 (28 mL) and the resulting reaction mixture was stirred at room temperature for 72 h. The reaction was then cooled to 0 °C and the emerging precipitate was filtered and washed with cold CH_2Cl_2 (10 mL). The collected filtrate was washed with a 10% aqueous solution of Na_2SO_4 (50 mL) followed by saturated aqueous $NaHCO_3$ (2 × 50 mL). The organic phase was then dried over $MgSO_4$ and concentrated under reduced pressure. The crude residue obtained was purified by flash column chromatography (0–60% Et_2O in *n*-pentane + 1% Et_3N) to afford thiophene 1,1-dioxide **19a** (409 mg, 46%) as a white solid.

MP: 65–66 °C (*n*-pentane)

TLC: R_f = 0.27 (50% Et_2O in *n*-pentane)

^{1}H NMR (400 MHz, Chloroform-*d*) δ 6.77 (d, J = 4.8 Hz, 1H), 6.42 (dt, J = 4.8, 1.9 Hz, 1H), 2.57 (td, J = 7.4, 1.8 Hz, 2H), 2.32 (t, J = 7.4 Hz, 2H), 1.97 (p, J = 7.4 Hz, 2H), 1.45 (s, 9H) ppm

^{13}C NMR (101 MHz, Chloroform-*d*) δ 172.1, 144.6, 127.5, 123.4, 119.8, 80.9, 34.4, 28.3, 24.8, 22.2 ppm

HRMS (*m/z*): (ESI) calculated for $C_{12}H_{17}BrNaO_4S$ $[M+Na]^+$: 358.9923, found: 358.9935

IR (thin film) ν_{max}: 2978, 2936, 1723, 1456, 1393, 1367, 1313 and 1145 cm^{-1}

4.2. Synthesis of *tert*-Butyl Protected Chlorinated Thiophene 1,1-Dioxide

***tert*-Butyl 4-(5-chloro-1,1-dioxidothiophen-2-yl)butanoate (19b)**

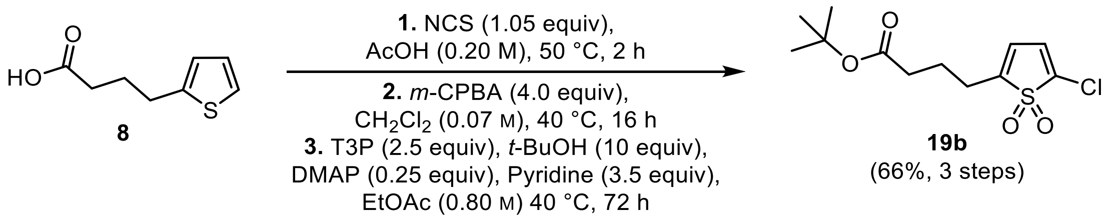

4-(2-Thienyl)butyric acid **8** (1.71 mL, 11.7 mmol, 1.00 equiv) was added to acetic acid (58.7 mL, 0.20 M) at room temperature and the mixture was stirred (ca. 30 s) to provide a homogenous solution. To this was added *N*-chlorosuccinimide (NCS, 1.65 g, 12.3 mmol, 1.05 equiv)[A] and following its complete dissolution, the reaction was placed into a pre-heated oil bath at 50 °C and stirred at this temperature for 2 h. The reaction was then cooled to room temperature, toluene (30 mL) was added, and the solution was concentrated under reduced pressure. The azeotropic removal of acetic acid with toluene was repeated twice more (2 × 30 mL) to afford a light red semi-solid. Cold Et_2O (20 mL) was then added, and the white solid (succinimide) was filtered, washing with further portions of cold Et_2O (2 × 20 mL). The filtrate was concentrated under reduced pressure and the precipitation/filtration/concentration procedure was repeated a further 2× to give a red oil.

The crude material was dissolved in CH_2Cl_2 (65 mL) and cooled to 0 °C. To this was added a solution of *m*-CPBA (≥70% purity, 11.6 g, 47.0 mmol, 4.00 equiv) in CH_2Cl_2 (100 mL) dropwise (syringe pump: 1 mL/min). Following this, the reaction was warmed to room temperature, a reflux condenser was attached, and the reaction mixture placed into a pre-heated oil bath at 40 °C and stirred at this temperature for 24 h. The reaction was cooled to room temperature and then to −78 °C (dry ice/acetone) for 15 min to precipitate out both *m*-CPBA

and *m*-chlorobenzoic acid.[B] A Teflon cannula for filtration under a positive pressure of nitrogen was then prepared by inserting a Teflon cannula through a Suba-Seal® and wrapping a small piece of filter paper around this end using PTFE tape.[C] The cannula was placed into the flask containing the crude product at −78 °C and a nitrogen balloon was inserted to facilitate cannula transfer into a 500 mL receiving flask. Once completed, further portions of CH_2Cl_2 at −78 °C (2 × 50 mL) were added to the transferring flask and the cannula transfer repeated. The filtrate was concentrated under reduced pressure to afford a crude light-yellow solid.

The crude material was dissolved in EtOAc (6.00 mL) and cooled to 0 °C. To this was added pyridine (3.33 mL, 41.1 mmol, 3.50 equiv) dropwise, followed by DMAP (359 mg, 2.94 mmol, 0.25 equiv) and *t*-BuOH (8.71 mL, 117 mmol, 10.0 equiv). Finally, a solution of *n*-propylphosphonic anhydride (T3P, ≥50 wt. % in EtOAc, 17.5 mL, 29.4 mmol, 2.50 equiv) was added slowly dropwise (syringe pump: 1 mL/min). The reaction was then warmed to room temperature and placed into a pre-heated oil bath at 40 °C and stirred at this temperature for 72 h. Following this, the reaction was cooled to room temperature and transferred into a separating funnel containing saturated aqueous $Na_2S_2O_3$ (50 mL) and the reaction flask was washed with EtOAc (25 mL). The aqueous phase was removed, and the organic phase was further washed with saturated aqueous $NaHCO_3$ (50 mL), saturated aqueous NH_4Cl (50 mL), and finally, brine (50 mL) before drying with Na_2SO_4, filtering, and concentrating under reduced pressure to give a crude off-white solid. This was purified by flash column chromatography (0–20% EtOAc in *n*-hexane) to afford thiophene 1,1-dioxide **19b** (2.28 g, 66% over three steps) as a white solid.

Notes: (**A**) *N*-Chlorosuccinimide was recrystallised from chloroform prior to use.[2] (**B**) Other temperatures (e.g., 0 °C, −20 °C) were also attempted for the precipitation of *m*-CPBA and *m*-chlorobenzoic acid but proved more ineffective compared to −78 °C. (**C**) A Teflon cannula for filtration under an inert atmosphere is prepared by cutting a piece of HPLC tubing to the required length and threading this through two appropriately sized Suba-Seals®. In our case, BOLA PTFE tubing (ID 1.5 mm, OD 2.5 mm) from Sigma-Aldrich was used (**Figure 1**; reproduced from above).

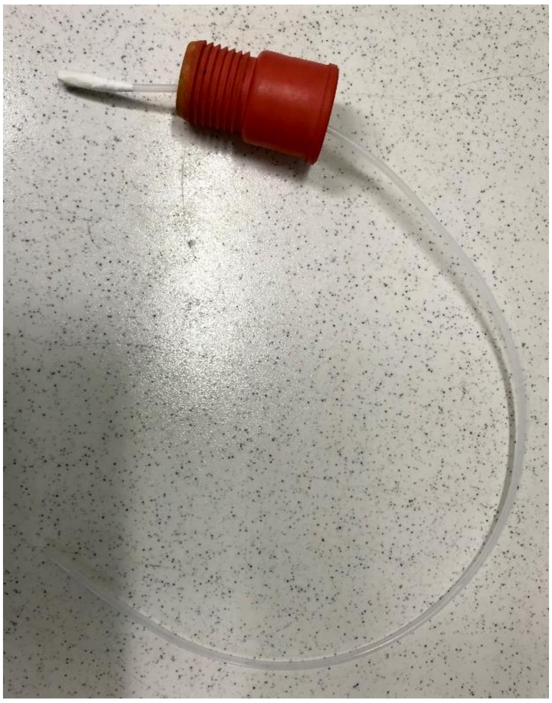

Figure 1 (reproduced from above). Image of the teflon cannula used for inert atmosphere filtration of *m*-CPBA and *m*-chlorobenzoic acid from the crude reaction mixture.

MP: 53–55 °C (*n*-hexane)

TLC: R_f = 0.36 (20% EtOAc in *n*-hexane)

^{1}H NMR (500 MHz, Chloroform-*d*) δ 6.58 (d, J = 4.9 Hz, 1H), 6.42 (dt, J = 4.9, 1.9 Hz, 1H), 2.51 (td, J = 7.4, 1.9 Hz, 2H), 2.28 (t, J = 7.4 Hz, 2H), 1.91 (p, J = 7.4 Hz, 2H), 1.40 (s, 9H) ppm

^{13}C NMR (126 MHz, Chloroform-*d*) δ 171.9, 142.8, 131.9, 123.1, 122.3, 80.7, 34.3, 28.1, 24.4, 22.0 ppm

HRMS (*m/z*): (ESI) calculated for $C_{12}H_{17}ClNaO_4S$ $[M+Na]^+$: 315.0428, found: 315.0438

IR (thin film) ν_{max}: 3078, 2978, 2935, 1721, 1634, 1563, 1457, 1431, 1393, 1367, 1312, 1275, 1255, 1143, 1047, 1001, 941, 908, 839, 753, 705, 660 and 629 cm^{-1}

4.3. Synthesis of Corey Lactone Dienes

(3a*R*,4*S*,5*R*,6a*S*)-5-((*tert*-Butyldimethylsilyl)oxy)-4-(((*tert*-butyldimethylsilyl)oxy)methyl)hexahydro-2*H*-cyclopenta[*b*]furan-2-one (17)

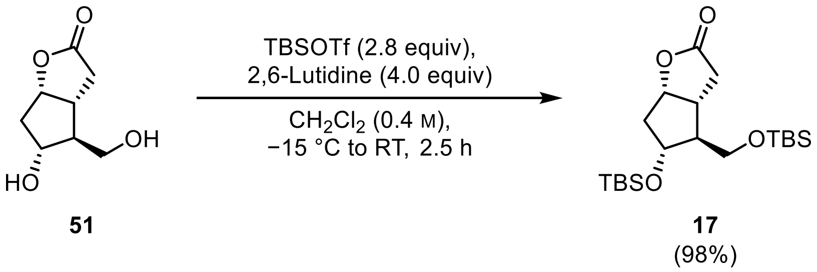

To a solution of Corey lactone **51** (1.00 g, 5.81 mmol, 1.00 equiv) in anhydrous CH_2Cl_2 (10 mL) at −15 °C was added 2,6-lutidine (2.71 mL, 23.2 mmol, 4.00 equiv) followed by a solution of *tert*-butyldimethylsilyl trifluoromethanesulfonate (TBSOTf, 3.74 mL, 16.3 mmol, 2.80 equiv) in anhydrous CH_2Cl_2 (5 mL) slowly dropwise (rate: 1 mL/min). The reaction mixture was then warmed to room temperature and stirred for 2.5 hours before diluting with CH_2Cl_2 (15 mL) and transferring to a separating funnel containing H_2O (30 mL). The organic phase was extracted and washed with saturated aqueous $NaHCO_3$ (30 mL), dried with $MgSO_4$, filtered, and concentrated under reduced pressure. The crude residue was purified by flash column chromatography (0–20% Et_2O in *n*-pentane) to afford di-silylated Corey lactone **17** (2.27 g, 5.66 mmol, 98%) as a white solid.

TLC: R_f = 0.38 (20% Et_2O in *n*-pentane)

^{1}H NMR (400 MHz, Chloroform-*d*) δ 4.92 (td, *J* = 7.3, 2.6 Hz, 1H), 4.12 (q, *J* = 5.5 Hz, 1H), 3.54 (dd, *J* = 10.3, 5.5 Hz, 1H), 3.49 (dd, *J* = 10.3, 5.5 Hz, 1H), 2.77 (dd, *J* = 17.8, 10.5 Hz, 1H), 2.66 (dddd, *J* = 10.5, 7.3, 5.5, 2.7 Hz, 1H), 2.54 (dd, *J* = 17.8, 2.7 Hz, 1H), 2.23 (ddd, *J* = 14.8, 7.0, 5.5 Hz, 1H), 2.01 – 1.94 (m, 2H), 0.89 (s, 9H), 0.87 (s, 9H), 0.05 (s, 6H), 0.04 (s, 6H) ppm

^{13}C NMR (101 MHz, Chloroform-*d*) δ 177.4, 84.3, 74.8, 62.7, 57.1, 41.2, 39.3, 35.8, 26.0, 25.9, 18.4, 18.1, −4.6, −4.9, −5.3, −5.4 ppm

Analytical data is consistent with that reported in the literature.[15]

***tert*-Butyl(((3a*R*,4*S*,5*R*,6a*S*)-5-((*tert*-butyldimethylsilyl)oxy)-3a,5,6,6a-tetrahydro-4*H*-cyclopenta[*b*]furan-4-yl)methoxy)dimethylsilane (18)**

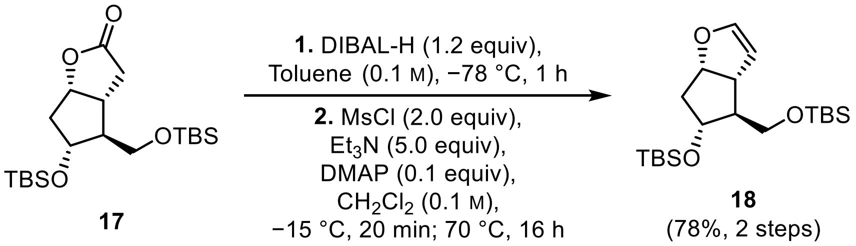

To a solution of di-silylated Corey lactone **17** (2.00 g, 5.00 mmol, 1.00 equiv) in anhydrous toluene (50.0 mL, 0.10 M) at −78 °C was added DIBAL-H (1.00 M in toluene, 6.00 mL, 6.00 mmol, 1.20 equiv) slowly dropwise (rate: 1 mL/min). The reaction mixture was then stirred at −78 °C for 1 hour before the cooling bath was removed and the reaction was immediately quenched with a saturated aqueous solution of Rochelle's salt (25 mL). The mixture was subsequently allowed to warm to room temperature and stirred for 16 h before transferring to a separating funnel and extracting with CH_2Cl_2 (2 × 50 mL). The organic phases were combined, washed with brine (50 mL), dried with $MgSO_4$, filtered, and concentrated under reduced pressure to afford a crude colourless oil.

The crude residue was then dissolved in anhydrous CH_2Cl_2 (50 mL, 0.10 M) and transferred to a flame-dried pressure flask under an atmosphere of nitrogen. To this was added Et_3N (3.50 mL, 25.0 mmol, 5.00 equiv) followed by DMAP (61 mg, 0.50 mmol, 0.10 equiv) and the flask was cooled to −15 °C. Methanesulfonyl chloride (MsCl, 0.77 mL, 10.0 mmol, 2.00 equiv) was added slowly dropwise and the reaction was allowed to stir at this temperature for 20 min before warming to room temperature, sealing the pressure flask, and heating to 70 °C for 16 h. The reaction was then cooled to room temperature and transferred to a separating funnel where the organic phase was washed with saturated aqueous NH_4Cl (2 × 50 mL), dried with $MgSO_4$, filtered, and concentrated under reduced pressure to afford a crude residue. The crude product was purified by flash column chromatography (0–40% Et_2O in *n*-pentane) to afford enol ether **18** (1.50 g, 78%) as a colourless oil.

TLC: R_f = 0.55 (2.5% Et_2O in *n*-pentane)

^{1}H NMR (400 MHz, Chloroform-*d*) δ 6.19 (t, J = 2.3 Hz, 1H), 4.98 (t, J = 2.3 Hz, 1H), 4.77 (ddd, J = 10.0, 7.4, 6.1 Hz, 1H), 3.94 – 3.81 (m, 1H), 3.63 (dd, J = 10.2, 4.1 Hz, 1H), 3.57 (dd, J = 10.2, 5.3 Hz, 1H), 3.01 (ddt, J = 9.8, 7.4, 2.3 Hz, 1H), 2.39 (ddd, J = 13.2, 7.3, 6.1 Hz, 1H), 1.86 – 1.72 (m, 2H), 0.89 (s, 9H), 0.87 (s, 9H), 0.05 (s, 6H), 0.04 (s, 6H) ppm

^{13}C NMR (101 MHz, Chloroform-d_6) δ 144.1, 104.4, 83.1, 72.2, 62.5, 57.7, 46.7, 42.8, 26.1, 25.9, 18.4, 18.1, −4.4, −4.7, −5.3, −5.4 ppm

HRMS (*m/z*): (ESI) calculated for $C_{20}H_{40}NaO_3Si_2$ $[M+Na]^+$: 407.2408, found: 407.2403

IR (thin film) ν_{max}: 2930, 2857, 1613, 1472, 1389, 1255, 1107, 906, 834, 775 and 729 cm^{-1}

***tert*-Butyl 4-((1*S*,2*R*,3a*S*,4a*R*,8a*S*,8b*S*)-8-bromo-2-((*tert*-butyldimethylsilyl)oxy)-1-(((*tert*-butyldimethylsilyl)oxy)methyl)-2,3,3a,4a,8a,8b-hexahydro-1*H*-cyclopenta[*b*]benzofuran-5-yl)butanoate (20a)**

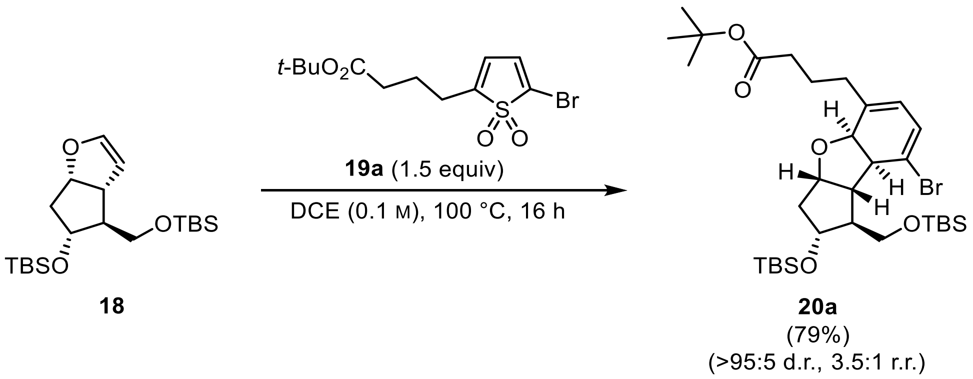

To a flame-dried microwave vial containing a solution of enol ether **18** (235 mg, 0.61 mmol, 1.0 equiv) in DCE (6.1 mL, 0.1 M) was added thiophene 1,1-dioxide **19a** (327 mg, 0.92 mmol, 1.5 equiv) followed by 2,6-lutidine (0.34 mL, 3.05 mmol, 5.0 equiv). The vial was subsequently sealed, and the reaction was placed into a pre-heated oil bath at 100 °C and stirred for 16 h. The reaction was cooled to room temperature, diluted with CH_2Cl_2 (5 mL) and the solution transferred to a separating funnel containing saturated aqueous NH_4Cl (10 mL). The organic phase was collected, and the aqueous phase was further extracted with CH_2Cl_2 (2 × 10 mL). The organic phases were combined, washed with brine (10 mL), dried with $MgSO_4$, filtered, and concentrated under reduced pressure. The crude residue obtained was purified by flash column chromatography (0–10% Et_2O in *n*-hexane) to afford bromo-diene **20a** (318 mg, 79%, >95:5 d.r., 3.5:1 r.r.) as a colourless oil. Further iterations of flash column chromatography using the above conditions was used to fully separate the regioisomers for characterisation.[A,B]

Notes: (**A**) NMR analysis was performed in benzene-d_6 due to partial instability of bromo-diene **20a** in chloroform-*d*, which previously showed decomposition when left overnight in an NMR tube containing chloroform-*d*. (**B**) Flash column chromatography was performed using 0–10% Et_2O in *n*-hexane to separate the major regioisomer for characterisation with 10% acetone in *n*-hexane being used as the TLC eluent. This was due to better separation on TLC

using the acetone conditions; however, these conditions did not transfer well to flash column chromatography as all regioisomers eluted at once.

TLC: R_f = 0.58 (10% acetone in *n*-hexane)

^{1}H NMR (500 MHz, Benzene-d_6) δ 6.06 (dd, J = 6.3, 1.6 Hz, 1H), 5.32 (d, J = 6.3 Hz, 1H), 4.47 (d, J = 8.1 Hz, 1H), 4.19 (td, J = 7.1, 4.3 Hz, 1H), 4.06 (q, J = 7.3 Hz, 1H), 3.70 (dd, J = 10.0, 4.2 Hz, 1H), 3.60 (dd, J = 10.0, 4.2 Hz, 1H), 2.96 – 2.89 (m, 2H), 2.25 – 2.18 (m, 1H), 2.18 – 2.14 (m, 2H), 2.14 – 2.08 (m, 1H), 2.08 – 2.02 (m, 1H), 1.89 (tt, J = 7.3, 4.2 Hz, 1H), 1.84 – 1.75 (m, 3H), 1.40 (s, 9H), 1.01 (s, 9H), 0.99 (s, 9H), 0.15 (s, 3H), 0.14 (s, 3H), 0.11 (s, 3H), 0.06 (s, 3H) ppm

^{13}C NMR (126 MHz, Benzene-d_6) δ 172.4, 137.2, 126.6, 125.3, 120.9, 79.5, 79.0, 78.1, 73.6, 61.6, 56.2, 53.3, 51.7, 41.9, 35.1, 34.0, 28.2, 26.3, 26.1, 23.3, 18.6, 18.2, −4.2, −4.7, −5.1, −5.2 ppm

HRMS (*m/z*): (ESI) calculated for $C_{32}H_{57}BrNaO_5Si_2$ $[M+Na]^+$: 657.3001, found: 657.3004

IR (thin film) ν_{max}: 2954, 2929, 2887, 2856, 1730, 1682, 1472, 1463, 1390, 1367, 1252, 1147, 1110, 833 and 775 cm^{-1}

$[\alpha]_D^{24}$: –43.3 (c = 0.10, CH_2Cl_2)

***tert*-Butyl 4-((1*S*,2*R*,3a*S*,4a*R*,8a*S*,8b*S*)-2-((*tert*-butyldimethylsilyl)oxy)-1-(((*tert*-butyldimethylsilyl)oxy)methyl)-8-chloro-2,3,3a,4a,8a,8b-hexahydro-1*H*-cyclopenta[*b*]benzofuran-5-yl)butanoate (20b)**

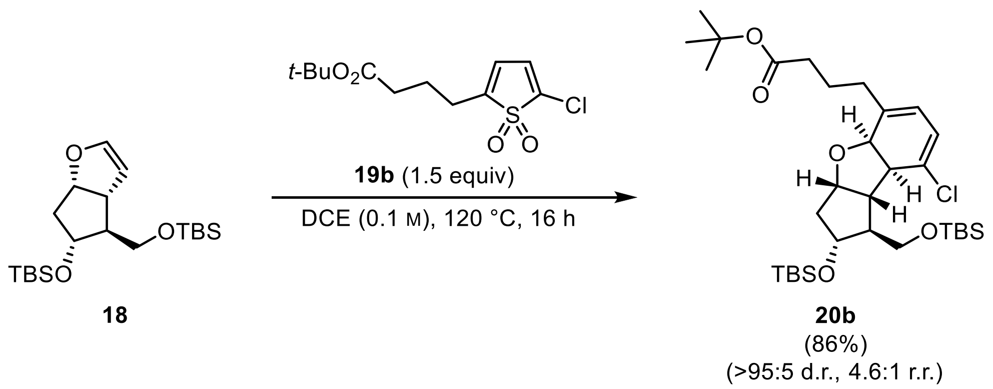

To a flame-dried microwave vial containing a solution of enol ether **18** (200 mg, 0.52 mmol, 1.0 equiv) in DCE (5.2 mL, 0.1 M) was added thiophene 1,1-dioxide **19b** (228 mg, 0.78 mmol, 1.5 equiv) followed by 2,6-lutidine (0.30 mL, 2.59 mmol, 5.0 equiv). The vial was subsequently sealed, and the reaction was placed into a pre-heated oil bath at 120 °C and stirred for 16 h.

The reaction was cooled to room temperature, diluted with CH_2Cl_2 (5 mL) and the solution transferred to a separating funnel containing saturated aqueous NH_4Cl (10 mL). The organic phase was collected, and the aqueous phase was further extracted with CH_2Cl_2 (2 × 10 mL). The organic phases were combined, washed with brine (10 mL), dried with $MgSO_4$, filtered, and concentrated under reduced pressure. The crude residue obtained was purified by flash column chromatography (0–5% Et_2O in *n*-hexane) to afford chloro-diene **20b** (255 mg, 86%, >95:5 d.r., 4.6:1 r.r.) as a colourless oil. Further iterations of flash column chromatography using the above conditions was used to fully separate the regioisomers for characterisation.

TLC: R_f = 0.38 (10% Et_2O in *n*-hexane)

^{1}H NMR (500 MHz, Benzene-d_6) δ 5.84 (dd, *J* = 6.3, 1.7 Hz, 1H), 5.41 (dd, *J* = 6.3, 1.4 Hz, 1H), 4.50 (d, *J* = 8.4 Hz, 1H), 4.18 (td, *J* = 7.6, 4.4 Hz, 1H), 4.04 (app. q, *J* = 7.3 Hz, 1H), 3.71 (dd, *J* = 10.0, 4.0 Hz, 1H), 3.60 (dd, *J* = 10.0, 4.0 Hz, 1H), 2.95 (td, *J* = 7.6, 2.6 Hz, 1H), 2.82 (dt, *J* = 8.4, 2.6 Hz, 1H), 2.29 – 2.05 (m, 5H), 1.91 – 1.73 (m, 4H), 1.40 (s, 9H), 1.01 (s, 9H), 0.99 (s, 9H), 0.14 (s, 3H), 0.13 (s, 3H), 0.10 (s, 3H), 0.06 (s, 3H) ppm

^{13}C NMR (126 MHz, Benzene-d_6) δ 172.4, 136.5, 134.5, 121.1, 120.4, 79.5, 79.0, 77.9, 73.4, 61.3, 56.1, 51.7, 50.2, 41.9, 35.1, 34.0, 28.2, 26.2, 26.1, 23.4, 18.5, 18.2, −4.3, −4.7, −5.2, −5.3 ppm

HRMS (*m/z*): (ESI) calculated for $C_{32}H_{57}ClNaO_5Si_2$ $[M+Na]^+$: 635.3325, found: 635.3320

IR (thin film) ν_{max}: 2954, 2930, 2888, 2857, 1730, 1664, 1596, 1471, 1390, 1366, 1254, 1141, 1111, 1005, 940, 880, 835, 776 and 670 cm^{-1}

$[\alpha]_D^{23}$**:** –42.0 (*c* = 0.14, CH_2Cl_2)

4-((1*S*,2*R*,3a*S*,8a*S*,8b*S*)-2-((*tert*-Butyldimethylsilyl)oxy)-1-(((*tert*-butyldimethylsilyl)oxy)methyl)-8-chloro-2,3,3a,4a,8a,8b-hexahydro-1*H*-cyclopenta[*b*]benzofuran-5-yl)butanoic acid (20c)

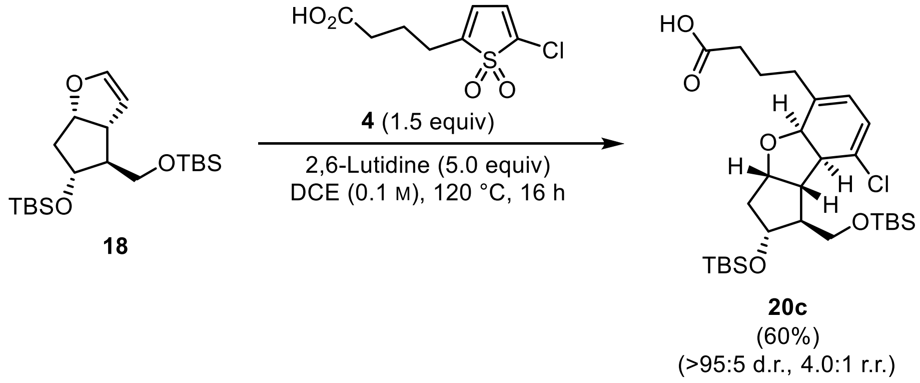

To a flame-dried microwave vial containing a solution of thiophene 1,1-dioxide **4** (71.0 mg, 0.30 mmol, 1.50 equiv) in anhydrous DCE (1.0 mL) was added 2,6-lutidine (0.12 mL, 1.0 mmol, 5.0 equiv) and the solution was stirred for 5 mins. A solution of enol ether **18** (76.9 mg, 0.20 mmol, 1.0 equiv) in DCE (1 mL) was then added to the mixture. The vial was subsequently sealed and placed into a pre-heated oil bath at 120 °C and stirred for 16 h. Following this, the reaction was allowed to cool to room temperature before it was diluted with CH_2Cl_2 (5 mL) followed by addition of 1 M hydrochloric acid (5 mL). The aqueous phase was extracted with CH_2Cl_2 (3 × 5 mL) and the organic phases were combined, washed with brine (5 mL), dried with Na_2SO_4, filtered, and concentrated under reduced pressure. The crude residue obtained was purified by flash column chromatography (20%–40% EtOAc in *n*-hexane) to afford chloro-diene **20c** (67 mg, 60%, >95:5 d.r., 4.0:1 r.r.) as a colourless oil. Further iterations of flash column chromatography using the above conditions were used to fully separate the regioisomers for characterisation.

TLC: R_f = 0.40 (50% EtOAc in *n*-hexane)

^{1}H NMR (500 MHz, Chloroform-*d*) δ 5.96 (dd, *J* = 6.3, 1.8 Hz, 1H), 5.77 – 5.71 (m, 1H), 4.60 (d, *J* = 8.1 Hz, 1H), 4.29 (td, *J* = 7.2, 4.4 Hz, 1H), 4.04 (app. q, *J* = 7.2 Hz, 1H), 3.68 (dd, *J* = 10.0, 4.4 Hz, 1H), 3.63 (dd, *J* = 10.0, 4.4 Hz, 1H), 2.96 (dt, *J* = 8.1, 2.5 Hz, 1H), 2.91 (td, *J* = 7.2, 2.5 Hz, 1H), 2.38 (t, *J* = 7.4 Hz, 2H), 2.32 – 2.20 (m, 3H), 1.93 – 1.76 (m, 3H), 1.67 (ddd, *J* = 13.7, 7.2, 4.4 Hz, 1H), 0.90 (s, 9H), 0.88 (s, 9H), 0.06 (s, 12H) ppm

^{13}C NMR (126 MHz, Chloroform-*d*) δ 179.3, 135.4, 134.6, 120.9, 120.5, 79.1,* 79.0,* 77.3, 73.2,* 73.2,* 61.4, 56.0, 51.6,* 51.5,* 49.8, 41.7, 33.8, 33.5, 26.1, 26.0, 22.7, 18.4, 18.2, −4.4,* −4.4,* −4.7,* −4.8,* −5.3,* −5.3,* −5.4,* −5.5* ppm

Note: Certain peaks, in particular those nearby the TBS groups, appear doubled in the ^{13}C NMR. This is proposed to be due to the restricted rotation imposed upon these groups by the chlorine of the nearby chloro-diene system, causing rotameric forms. These double peaks have been assigned an asterisk (*).

HRMS (*m/z*): (ESI) calculated for $C_{28}H_{48}ClO_5Si_2$ $[M–H]^-$: 555.2734, found: 555.2731

IR (thin film) ν_{max}: 2954, 2928, 2856, 1709, 1472, 1252, 1111, 880, 834, 775 and 669 cm^{-1}

$[\alpha]_D^{23}$**:** –24.0 (*c* = 2.00, $CHCl_3$)

4.4. Optimization of Corey Lactone Diene Dehalogenation–Aromatization

4.4.1. Palladium and Platinum Catalysis

Table 2. Screening of palladium and platinum catalysts to enable the dehalogenation–aromatization of bromo-diene 20a in xylene at 130 °C.

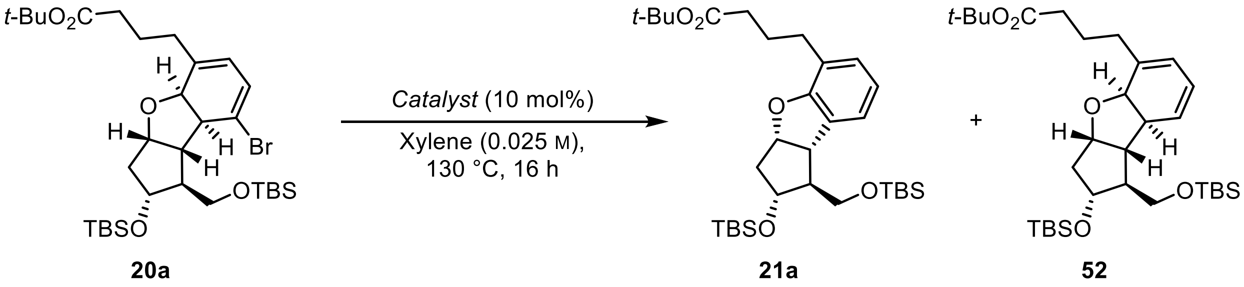

Entry	Catalyst	Atmosphere	21a	52	Notes
Nitrogen atmosphere					
1	Palladium black (surface area: 40-60 m^2/g)	Nitrogen	0%	0%	Decomposition
2	Platinum on activated carbon (5 wt.%)	Nitrogen	0%	0%	Decomposition
3	Palladium on activated carbon (10 wt.%)	Nitrogen	0%	*trace*	Decomposition
4	Platinum nanopowder (<50 nm particle size)	Nitrogen	0%	0%	Decomposition
Oxygen atmosphere					
5	Palladium black (surface area: 40-60 m^2/g)	Oxygen	0%	*trace*	Decomposition
6	Platinum on activated carbon (5 wt.%)	Oxygen	0%	*trace*	Decomposition
7	Palladium on activated carbon (10 wt.%)	Oxygen	0%	0%	Decomposition
8	Platinum nanopowder (<50 nm particle size)	Oxygen	0%	0%	Decomposition

Table 3. Additional screening of palladium catalysts[16]

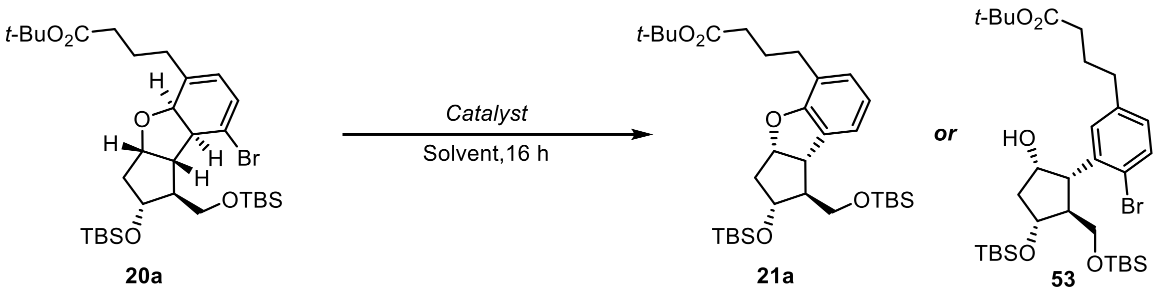

Entry	Catalyst	Atmosphere	Solvent	Temp.	21a	Notes
Catalyst						
1	Pd/C (10 wt.%, 20 mol%)	Nitrogen	PhMe	110 °C	0%	No conversion
2	Pd/C (10 wt.%, 20 mol%)	Nitrogen	Decalin	160 °C	0%	Elimination product (**53**)
3	$Pd(TFA)_2$ (20 mol%)	Oxygen	PhCl	110 °C	0%	Decomposition
4	$Pd(PPh_3)_4$ (10 mol%)	Nitrogen	Dioxane	100 °C	0%	No conversion
5	Pd/C (10 wt.%, 10 mol%), HCO_2NH_4 (1 equiv)	Nitrogen	Dioxane	100 °C	0%	Decomposition
6	$PdCl_2$ (20 mol %), NHC (20 mol%), $HSi(OEt)_2$ (30 mol %), 3,3-dimethylbut-1-ene (2 equiv)	Nitrogen	THF	rt	0%	Elimination product (**53**)

Table 4. Attempted aromatization of diene 52 using palladium catalysis

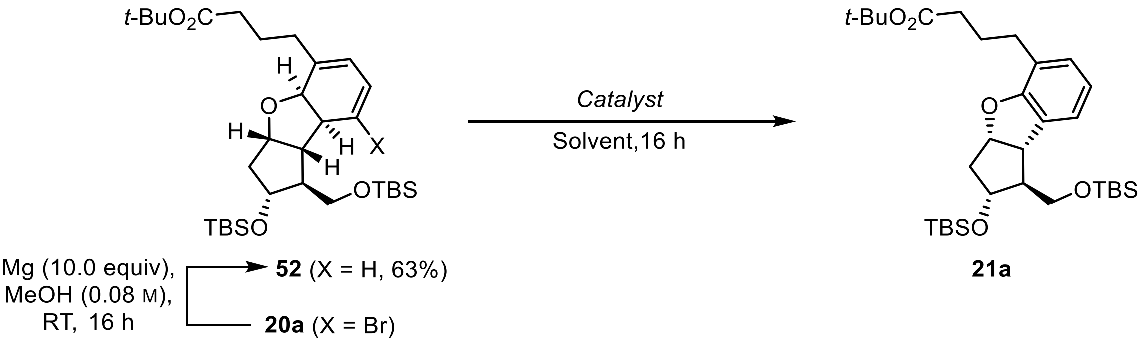

Entry	Catalyst	Atmosphere	Solvent	Temp.	21a	Notes
Catalyst						
1	Pd/C (10 wt.%, 10 mol%)	Nitrogen	PhMe	100 °C	**0%**	No conversion
2	Pd/C (10 wt.%, 20 mol%), hydroquinone (0.5 mol%)	Nitrogen	PhMe	100 °C	**0%**	Decomposition
3	Pd(TFA)$_2$ (20 mol%), sodium anthraquinone-2-sulfonate (20 mol%), MgSO$_4$ (0.8 equiv)	Oxygen	PhCl	110 °C	**0%**	Decomposition

4.4.2. Miscellaneous Oxidant Screening

Table 5. Screening of various oxidants to enable the dehalogenation–aromatization of bromo-diene 20a.

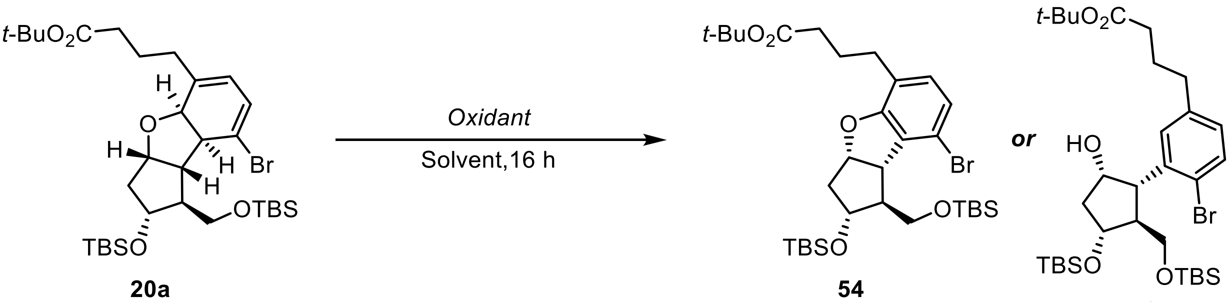

20a **54** **53**

Entry	Oxidant	Atmosphere	Solvent	Temp.	54	Notes
Oxidant						
1	DDQ (1.5 equiv)	Nitrogen	PhMe	110 °C	**0%**	Elimination product (**53**)
2	DBU (1.0 equiv)	Oxygen	MeCN	80 °C	**0%**	Elimination product (**53**)
3	KMnO$_4$•Al$_2$O$_3$	Nitrogen	Acetone	0 °C	**0%**	Decomposition
4	SeO$_2$ (1.5 equiv)	Nitrogen	Dioxane	100 °C	**11%**	-

Table 6. Screening of various oxidants to enable the aromatization of diene 39.

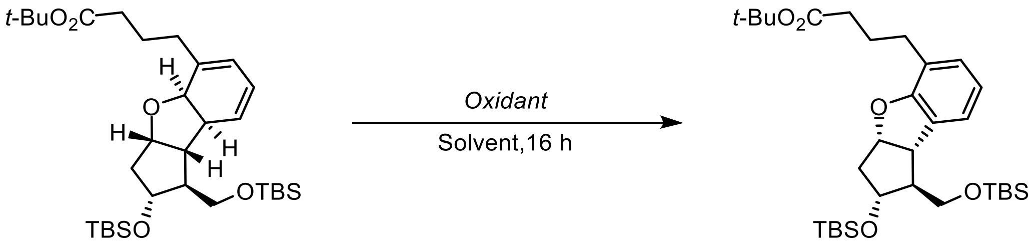

39 **21a**

Entry	Oxidant	Atmosphere	Solvent	Temp.	21a	Notes
Oxidant						
1	DDQ (1.5 equiv)	Nitrogen	PhMe	100 °C	**0%**	Decomposition
2	DDQ (1.5 equiv)	Nitrogen	PhMe	100 °C	**0%**	Cycloaddition adduct (**57**)
3	MnO_2 (10 equiv)	Nitrogen	Dioxane	100 °C	**0%**	No conversion
4	O_2 (1 atm.)	Oxygen	PhMe	100 °C	**0%**	Decomposition
5	**42** (1.5 equiv)	Nitrogen	PhMe	100 °C	**0%**	No conversion
6	**43** (1.5 equiv)	Nitrogen	PhMe	100 °C	**0%**	No conversion

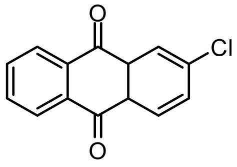

55

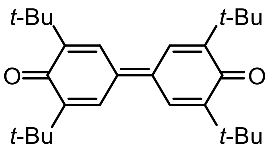

56

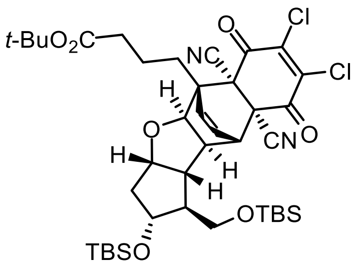

57

4.4.3. Discovery and Optimisation of *t*-BuLi-Mediated Dehalogenation–Aromatization

Table 7. Optimization of the dehalogenation–aromatization reaction of halodienes 20a–c using *t*-BuLi.

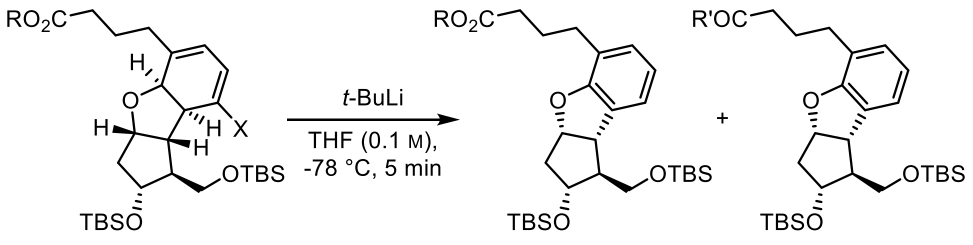

Entry	Substrate	*t*-BuLi (equiv)	Yield (%)[b] 20	21	22
1	**20a** (R = R' = *t*-Bu, X = Br)	2.0	29	7	2
2[c]	**20a** (R = R' = *t*-Bu, X = Br)	2.0	21	21	8
3[c,d]	**20a** (R = R' = *t*-Bu, X = Br)	3.0	23	33	13
4[c,d]	**20b** (R = R' = *t*-Bu, X = Cl)	3.0	0	56	32
5[c,d]	**20c** (R = H, R' = *t*-Bu, X = Cl)	4.0	20	38	N/A
6[c]	**20c (R = H, R' = *t*-Bu, X = Cl)**	**4.0**	***trace***	**66**[e]	N/A
7[c]	**20c** (R = H, R' = *t*-Bu, X = Cl)	3.0	*trace*	49[e]	N/A
8[c]	**20c** (R = H, R' = *t*-Bu, X = Cl)	*n*-BuLi (4.0)	59[e]	0	N/A
9[c]	**20c** (R = H, R' = *t*-Bu, X = Cl)	*s*-BuLi (4.0)	*trace*	60[e]	N/A

[a] Reaction conditions: **20** (0.05 mmol), THF (0.5 mL). [b] Measured by ^{1}H NMR spectroscopy using 1,3,5-trimethoxybenzene as an internal standard. [c] *n*-hexane:THF (95:5) was used instead of THF. [d] Inverse addition of lithium reagent was operated. [e] Isolated yields.

4-((1*S*,2*R*,3a*S*,8b*S*)-2-((*tert*-butyldimethylsilyl)oxy)-1-(((*tert*-butyldimethylsilyl)oxy)methyl)-2,3,3a,8b-tetrahydro-1*H*-cyclopenta[*b*]benzofuran-5-yl)butanoic acid (21b)

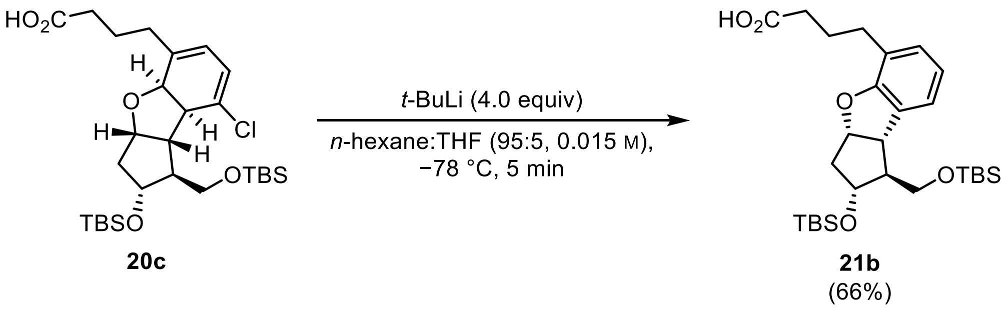

To a flame-dried Schlenk tube containing a solution of chloro-diene **20c** (27.9 mg, 0.05 mmol, 1.0 equiv) in anhydrous *n*-hexane:THF (95:5, 3.30 mL, 0.015 M) was added *tert*-butyllithium (*t*-BuLi, 1.61 M in *n*-pentane, 0.12 mL, 0.20 mmol, 4.0 equiv) at −78 °C slowly dropwise. The reaction was stirred at −78 °C for 5 mins before quenching with MeOH (0.20 mL) and warming to room temperature. The solvent was removed under reduced pressure and the crude residue was then dissolved in CH_2Cl_2 (5 mL) followed by addition of 1 M hydrochloric acid (5 mL). The aqueous phase was extracted with CH_2Cl_2 (3 × 5 mL) and the organic phases were combined, washed with brine (5 mL), dried with Na_2SO_4, filtered, and concentrated under reduced pressure. The crude residue obtained was purified by flash column chromatography (20–50% EtOAc in *n*-hexane) to afford aromatic **21b** (17 mg, 66%) as a colourless oil.

TLC: R_f = 0.56 (50% EtOAc in *n*-hexane)

^{1}H NMR (500 MHz, Chloroform-*d*) *δ* 7.02 (dt, *J* = 7.4, 1.3 Hz, 1H), 6.90 (dd, *J* = 7.4, 1.3 Hz, 1H), 6.75 (t, *J* = 7.4 Hz, 1H), 5.08 (ddd, *J* = 9.2, 7.2, 5.5 Hz, 1H), 4.07 (td, *J* = 7.2, 5.5 Hz, 1H), 3.74 (dd, *J* = 10.2, 4.4 Hz, 1H), 3.69 (dd, *J* = 10.2, 4.4 Hz, 1H), 3.59 (dd, *J* = 9.2, 6.8 Hz, 1H), 2.60 (qt, *J* = 14.0, 7.4 Hz, 2H), 2.47 – 2.33 (m, 3H), 2.05 (tt, *J* = 6.8, 4.4 Hz, 1H), 1.99 – 1.89 (m, 3H), 0.93 (s, 9H), 0.77 (s, 9H), 0.11 (s, 3H), 0.09 (s, 3H), 0.01 (s, 3H), −0.03 (s, 3H) ppm

^{13}C NMR (126 MHz, Chloroform-*d*) *δ* 178.6, 157.5, 131.2, 128.4, 123.0, 122.3, 120.3, 85.3, 72.8, 62.0, 57.8, 46.6, 42.6, 33.5, 29.3, 26.1, 25.8, 24.7, 18.5, 18.0, −4.5, −4.9, −5.2, −5.3 ppm

HRMS (*m/z*): (ESI) calculated for $C_{28}H_{49}O_5Si_2^+$ [M+H]$^+$: 521.3113, found: 521.3105

IR (thin film) ν_{max}: 2953, 2928, 2856, 1709, 1472, 1454, 1252, 1108, 1084, 834 and 775 cm^{-1}

$[\alpha]_D^{24}$**:** +4.0 (*c* = 2.00, $CHCl_3$)

Data for *tert*-Butyl Derivative (21a)

***tert*-Butyl 4-((1*S*,2*R*,3a*S*,8b*S*)-2-((*tert*-butyldimethylsilyl)oxy)-1-(((*tert*-butyldimethylsilyl)oxy)methyl)-2,3,3a,8b-tetrahydro-1*H*-cyclopenta[*b*]benzofuran-5-yl)butanoate (21a)**

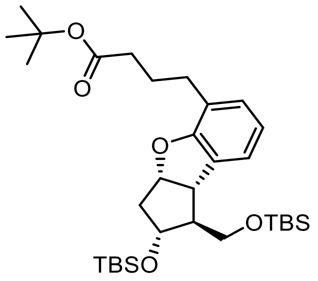

21a

TLC: R_f = 0.20 (5% Et_2O in *n*-hexane)

^{1}H NMR (500 MHz, Chloroform-*d*) δ 7.01 (dt, *J* = 7.4, 1.3 Hz, 1H), 6.91 (ddd, *J* = 7.5, 1.4, 0.7 Hz, 1H), 6.74 (t, *J* = 7.4 Hz, 1H), 5.07 (ddd, *J* = 9.2, 7.2, 5.3 Hz, 1H), 4.06 (ddd, *J* = 7.9, 7.0, 5.9 Hz, 1H), 3.74 (dd, *J* = 10.2, 4.3 Hz, 1H), 3.70 (dd, *J* = 10.2, 4.6 Hz, 1H), 3.59 (dd, *J* = 8.9, 7.1 Hz, 1H), 2.56 (qt, *J* = 13.7, 7.6 Hz, 2H), 2.44 (ddd, *J* = 13.3, 7.2, 6.0 Hz, 1H), 2.24 (dd, *J* = 8.0, 7.1 Hz, 2H), 2.04 (tt, *J* = 6.9, 4.3 Hz, 1H), 1.96 – 1.83 (m, 3H), 1.44 (s, 9H), 0.93 (s, 9H), 0.77 (s, 9H), 0.11 (s, 3H), 0.09 (s, 3H), 0.01 (s, 3H), −0.02 (s, 3H) ppm

^{13}C NMR (126 MHz, Chloroform-*d*) δ 173.3, 157.5, 131.1, 128.4, 123.4, 122.2, 120.2, 85.2, 80.1, 72.7, 61.9, 57.7, 46.5, 42.6, 35.4, 29.4, 28.3, 26.1, 25.8, 25.2, 18.5, 18.0, −4.5, −4.9, −5.2, −5.3 ppm

HRMS (*m/z*): (ESI) calculated for $C_{32}H_{56}NaO_5Si_2$ $[M+Na]^+$: 599.3558, found: 599.3571

IR (thin film) ν_{max}: 2954, 2930, 2893, 2857, 1730, 1598, 1455, 1366, 1254, 1109, 938, 835, 776 and 609 cm^{-1}

$[\alpha]_D^{24}$**:** +8.00 (*c* = 0.25, CH_2Cl_2)

5. Deuterium-Labelling Studies

(3a*R*,4*S*,5*R*,6a*S*)-5-((*tert*-butyldimethylsilyl)oxy)-4-(((*tert*-butyldimethylsilyl)oxy)methyl)hexahydro-2*H*-cyclopenta[*b*]furan-2-one-3,3-d_2 (58)

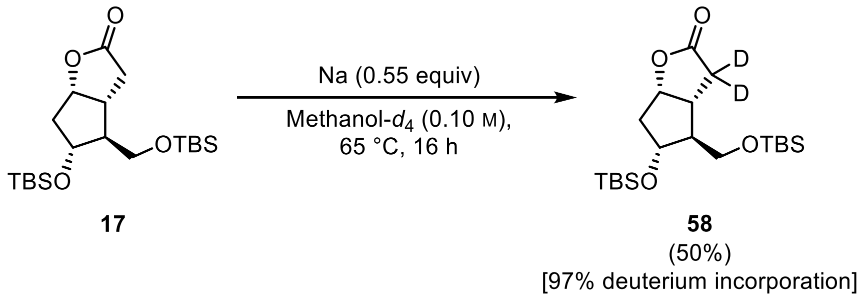

To a flame-dried Schlenk tube containing freshly cut sodium metal (8.90 mg, 0.39 mmol, 0.55 equiv) was added methanol-d_4 (3.50 mL) followed by a solution of silyl-protected Corey lactone **17** (281 mg, 0.71 mmol, 1.00 equiv) in methanol-d_4 (3.5 mL) dropwise at room temperature. The mixture was placed into a pre-heated oil bath at 65 °C and stirred at this temperature for 16 h. The reaction was then removed from the oil bath, cooled to 0 °C and quenched with saturated aqueous NH_4Cl (0.50 mL). The resultant white precipitate was filtered, washed with methanol (5.0 mL) and concentrated under reduced pressure to afford a crude residue that was purified by flash column chromatography (20% Et_2O in *n*-hexane) to give deuterated lactone **58** (142 mg, 50%, 97% deuterium incorporation) as an amorphous, colourless solid.

TLC: R_f = 0.15 (20% Et_2O in *n*-hexane)

^{1}H NMR (500 MHz, Chloroform-*d*) δ 4.90 (td, J = 7.2, 2.2 Hz, 1H), 4.11 (dt, J = 5.7, 4.6 Hz, 1H), 3.53 (dd, J = 10.3, 5.3 Hz, 1H), 3.46 (dd, J = 10.3, 5.7 Hz, 1H), 2.79 – 2.72 (m, 0.03 × 1H),* 2.64 (dd, J = 7.5, 5.5 Hz, 1H), 2.52 – 2.48 (m, 0.03 × 1H),* 2.21 (ddd, J = 14.7, 6.9, 5.7 Hz, 1H), 2.00 – 1.91 (m, 2H), 0.87 (s, 9H), 0.85 (s, 9H), 0.05 – 0.00 (m, 12H) ppm

Note: The multiplet peaks at 2.79 – 2.72 ppm and 2.52 – 2.48 ppm (marked with an asterisk above) correspond to the non-deuterated product (*cf.* compound **17**) and integration of this peak gives a value of 0.03. This integration value corresponds to 97% deuterium incorporation.

^{13}C NMR (126 MHz, Chloroform-*d*) δ 177.4, 84.3, 74.8, 62.7, 57.0, 41.1, 39.1, 35.7 – 34.7 (m),* 26.0, 25.8, 18.3, 18.0, −4.6, −4.9, −5.4, −5.5 ppm

Carbon attached to deuterium marked with an asterisk (*) and appears as a multiplet

HRMS (*m/z*): (ESI) calculated for $C_{20}H_{39}D_2O_4Si_2$ $[M+H]^+$: 403.2663, found: 403.2652

IR (thin film) ν_{max}: 2953, 2929, 2888, 2857, 2099, 1749, 1635, 1471, 1361, 1252, 1193, 1099, 1032, 836 and 776 cm^{-1}

$[\alpha]_D^{23}$**:** −15.0 (c = 1.00, $CHCl_3$)

***tert*-butyl(((3a*R*,4*S*,5*R*,6a*S*)-5-((*tert*-butyldimethylsilyl)oxy)-3a,5,6,6a-tetrahydro-4*H*-cyclopenta[*b*]furan-4-yl-3-*d*)methoxy)dimethylsilane (59)**

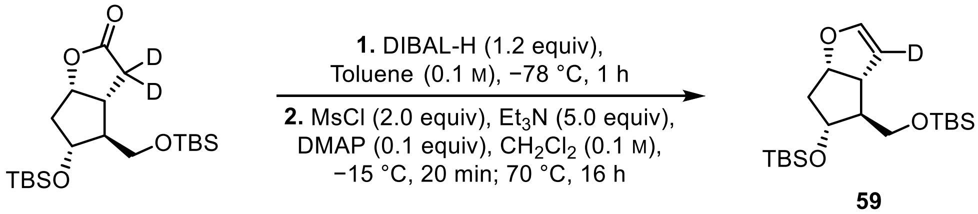

58
[97% deuterium incorporation]

59
(60%, 2 steps)
[97% deuterium incorporation]

To a solution of deuterated Corey lactone **58** (121 mg, 0.30 mmol, 1.00 equiv) in anhydrous toluene (3.00 mL, 0.10 M) at −78 °C was added DIBAL-H (1.00 M in toluene, 0.36 mL, 0.36 mmol, 1.20 equiv) slowly dropwise (rate: 1 mL/min). The reaction mixture was then stirred at −78 °C for 1 hour before the cooling bath was removed and the reaction was immediately quenched with a saturated aqueous solution of Rochelle's salt (3.00 mL). The mixture was subsequently allowed to warm to room temperature and stirred for 16 h before transferring to a separating funnel and extracting with CH_2Cl_2 (2 × 10 mL). The organic phases were combined, washed with brine (10 mL), dried with $MgSO_4$, filtered, and concentrated under reduced pressure to afford a crude colourless oil.

The crude residue was then dissolved in anhydrous CH_2Cl_2 (3.00 mL, 0.10 M) and transferred to a flame-dried pressure flask under an atmosphere of nitrogen. To this was added Et_3N (0.20 mL, 1.50 mmol, 5.00 equiv) followed by DMAP (3.70 mg, 0.03 mmol, 0.10 equiv) and the flask was cooled to −15 °C. Methanesulfonyl chloride (MsCl, 46.0 μL, 0.60 mmol, 2.00 equiv) was added slowly dropwise and the reaction was allowed to stir at this temperature for 20 min before warming to room temperature, sealing the pressure flask, and heating to 70 °C for 16 h. The reaction was then cooled to room temperature and transferred to a separating funnel where the organic phase was washed with saturated aqueous NH_4Cl (2 × 10 mL), dried with $MgSO_4$, filtered, and concentrated under reduced pressure to afford a crude residue. The crude product was purified by flash column chromatography (0–5% Et_2O in n-pentane) to afford deuterated enol ether **59** (78 mg, 60%, 97% deuterium incorporation) as a colourless oil.

TLC: R_f = 0.55 (2.5% Et_2O in *n*-pentane)

^{1}H NMR (500 MHz, Chloroform-*d*) *δ* 6.19 (d, *J* = 2.0 Hz, 1H), 4.98 (t, *J* = 2.7 Hz, 0.03 × 1H),* 4.77 (dddd, *J* = 9.8, 7.4, 6.1, 0.6 Hz, 1H), 3.90 (ddd, *J* = 8.8, 7.8, 6.1 Hz, 1H), 3.64 (dd, *J* = 10.1, 4.0 Hz, 1H), 3.57 (dd, *J* = 10.1, 5.4 Hz, 1H), 3.01 (ddd, *J* = 9.8, 7.2, 2.0 Hz, 1H), 2.39 (ddd, *J* = 13.2, 7.4, 6.1 Hz, 1H), 1.85 – 1.74 (m, 2H), 0.89 (s, 9H), 0.87 (s, 9H), 0.05 (s, 6H), 0.04 (s, 6H) ppm

Note: The peak at 4.98 ppm (marked with an asterisk above) corresponds to the non-deuterated product (*cf.* compound **18**) and integration of this peak gives a value of 0.03. This integration value corresponds to 97% deuterium incorporation (100% retention of deuterium from starting material).

^{13}C NMR (126 MHz, Chloroform-*d*) *δ* 144.0, 104.2 (t, *J* = 26.5 Hz),* 83.1, 72.2, 62.4, 57.7, 46.6, 42.8, 26.1, 25.9, 18.4, 18.1, −4.4, −4.7, −5.3, −5.4 ppm

Carbon attached to deuterium marked with an asterisk (*) and appears as a 1:1:1 triplet

HRMS (*m/z*): (ESI) calculated for $C_{20}H_{40}DO_3Si_2$ $[M+H]^+$: 386.2652, found: 386.2654

IR (thin film) ν_{max}: 2930, 2857, 1613, 1472, 1389, 1255, 1107, 906, 834, 775 and 729 cm^{-1}

$[\alpha]_D^{23}$**:** −17.2 (*c* = 1.63, $CHCl_3$)

4-((1*S*,2*R*,3a*S*,4a*R*,8a*S*,8b*S*)-2-((*tert*-butyldimethylsilyl)oxy)-1-(((*tert*-butyldimethylsilyl)oxy)methyl)-8-chloro-2,3,3a,4a,8a,8b-hexahydro-1*H*-cyclopenta[*b*]benzofuran-5-yl-8a-d)butanoic acid (23a)

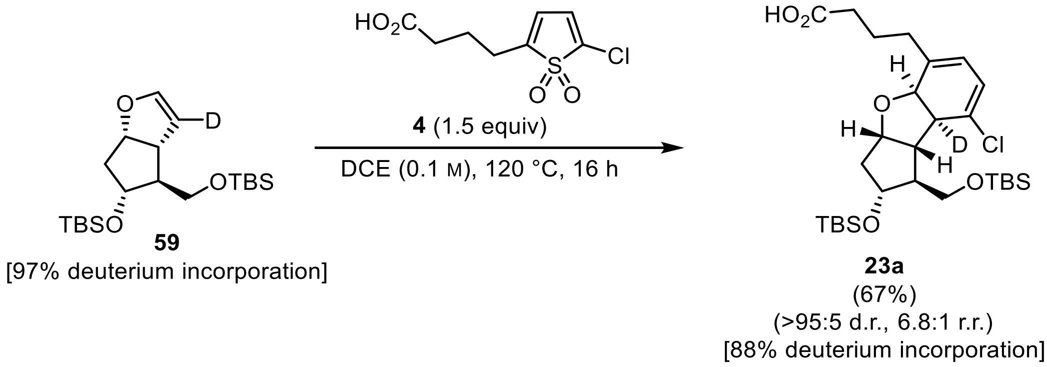

To a flame-dried microwave vial containing a solution of thiophene 1,1-dioxide **4** (71.0 mg, 0.30 mmol, 1.5 equiv) in anhydrous DCE (1.0 mL) was added 2,6-lutidine (0.12 mL, 1.0 mmol, 5.0 equiv) and the solution was stirred for 5 mins. Enol ether **59** (77.1 mg, 0.20 mmol, 1.0 equiv) in anhydrous DCE (1.0 mL) was then added to the mixture. The vial was subsequently

sealed, and the reaction was placed into a pre-heated oil bath at 120 °C and stirred for 16 h. Following this, the reaction was allowed to cool to room temperature before it was diluted with CH_2Cl_2 (5 mL) followed by the addition of 1 M hydrochloric acid (5 mL). The aqueous phase was extracted with CH_2Cl_2 (3 × 5 mL) and the organic phases were combined, washed with brine (5 mL), dried with Na_2SO_4, filtered, and concentrated under reduced pressure. The crude residue obtained was purified by flash column chromatography (20%–40% EtOAc in *n*-hexane) to afford chloro-diene **23a** (67 mg, 67%, >95:5 d.r., 6.8:1 r.r., 88% deuterium incorporation) as a colourless oil. Further iterations of flash column chromatography using the above conditions was used to fully separate the regioisomers for characterisation.

TLC: R_f = 0.40 (50% EtOAc in *n*-hexane)

^{1}H NMR (500 MHz, Chloroform-*d*) *δ* 5.95 (d, *J* = 6.3 Hz, 1H), 5.73 (dt, J = 6.3, 1.5 Hz, 1H), 4.60 (s, 1H), 4.28 (td, *J* = 7.4, 4.5 Hz, 1H), 4.04 (app. q, *J* = 6.8 Hz, 1H), 3.68 (dd, *J* = 10.1, 4.3 Hz, 1H), 3.63 (dd, *J* = 10.1, 4.3 Hz, 1H), 2.96 (d, *J* = 8.4 Hz, 0.13 × 1H),* 2.89 (t, *J* = 7.6 Hz, 1H), 2.38 (t, *J* = 7.4 Hz, 2H), 2.32 – 2.19 (m, 3H), 1.89 – 1.80 (m, 3H), 1.67 (ddd, *J* = 13.8, 7.4, 4.5 Hz, 1H), 0.89 (s, 9H), 0.88 (s, 9H), 0.07 – 0.04 (m, 12H) ppm

Note: The peak at 2.96 ppm (marked with an asterisk above) corresponds to the non-deuterated product (*cf.* compound **20c**) and integration of this peak gives a value of 0.13. This integration value corresponds to 88% deuterium incorporation (91% retention of deuterium from starting material).

^{13}C NMR (126 MHz, Chloroform-*d*) *δ* 179.0, 135.4, 134.6, 120.8, 120.5, 79.1, 77.4, 73.3, 61.5, 56.0, 51.2 (t, *J* = 21.2 Hz),* 49.9, 41.7, 33.8, 33.5, 26.1, 26.0, 22.7, 18.4, 18.1, −4.4, −4.7, −5.3, −5.4 ppm

Carbon attached to deuterium marked with an asterisk (*) and appears as a 1:1:1 triplet

HRMS (*m/z*): (ESI) calculated for $C_{28}H_{47}DClO_5Si_2$ $[M–H]^-$: 556.2797, found: 556.2797

IR (thin film) ν_{max}: 2954, 2928, 2894, 2856, 1710, 1472, 1252, 1102, 835 and 775 cm^{-1}

$[\alpha]_D^{23}$**:** −19.1 (*c* = 1.78, $CHCl_3$)

4-((1*S*,2*R*,3a*S*,8b*S*)-2-((*tert*-butyldimethylsilyl)oxy)-1-(((*tert*-butyldimethylsilyl)oxy)methyl)-2,3,3a,8b-tetrahydro-1*H*-cyclopenta[*b*]benzofuran-5-yl-8-d)butanoic acid (24)

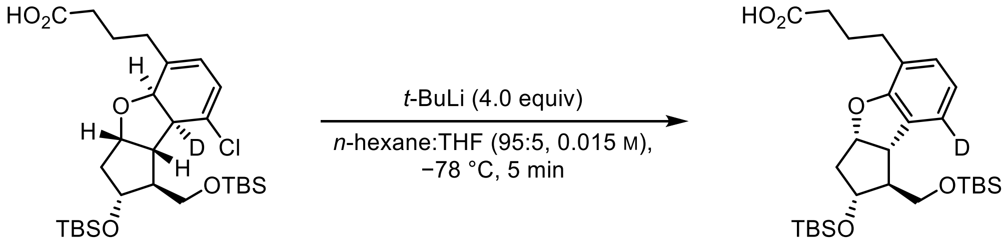

23a
[88% deuterium incorporation]

24 (56%)
[88% deuterium incorporation]

To a flame-dried Schlenk tube containing a solution of chloro-diene **23a** (27.9 mg, 0.05 mmol, 1.0 equiv) in anhydrous *n*-hexane:THF (95:5, 3.3 mL, 0.015 M) was added *t*-BuLi (1.61 M in *n*-pentane, 0.12 mL, 0.20 mmol, 4.0 equiv) at −78 °C slowly dropwise. The reaction was stirred at −78 °C for 5 mins before quenching with MeOH (0.20 mL) and warming to room temperature. The solvent was removed under reduced pressure and the crude residue was then dissolved in CH_2Cl_2 (5 mL) followed by addition of 1 M hydrochloric acid (5 mL). The aqueous phase was extracted with CH_2Cl_2 (3 × 5 mL) and the organic phases were combined, washed with brine (5 mL), dried with Na_2SO_4, filtered, and concentrated under reduced pressure. The crude residue obtained was purified by flash column chromatography (20–50% EtOAc in *n*-hexane) to afford aromatic **24** (14.6 mg, 56%, 88% deuterium incorporation) as a colourless oil.

TLC: R_f = 0.56 (50% EtOAc in *n*-hexane)

^{1}H NMR (500 MHz, Chloroform-*d*) *δ* 7.02 (dt, *J* = 7.3, 1.2 Hz, 0.13 × 1H),* 6.91 (dd, *J* = 7.5, 0.7 Hz, 1H), 6.78 – 6.71 (m, 1H), 5.08 (ddd, *J* = 9.2, 7.2, 5.2 Hz, 1H), 4.07 (ddd, *J* = 7.7, 6.9, 5.9 Hz, 1H), 3.74 (dd, *J* = 10.3, 4.5 Hz, 1H), 3.69 (dd, *J* = 10.3, 4.5 Hz, 1H), 3.59 (dd, *J* = 9.2, 6.9 Hz, 1H), 2.68 – 2.53 (m, 2H), 2.48 – 2.35 (m, 3H), 2.05 (app. tt, *J* = 6.9, 4.5 Hz, 1H), 2.00 – 1.88 (m, 3H), 0.93 (s, 9H), 0.77 (s, 9H), 0.11 (s, 3H), 0.10 (s, 3H), 0.01 (s, 3H), −0.03 (s, 3H) ppm

Note: The peak at 7.02 ppm (marked with an asterisk above) corresponds to the non-deuterated product (*cf.* compound **21b**) and integration of this peak gives a value of 0.13. This integration value corresponds to 88% deuterium incorporation (100% retention of deuterium from starting material).

^{13}C NMR (126 MHz, Chloroform-*d*) δ 179.1, 157.5, 131.1, 128.4, 122.9, 120.2, 85.3, 72.8, 62.0, 57.8, 46.6, 42.6, 33.5, 29.3, 26.1, 25.8, 24.7, 18.5, 18.0, −4.5, −4.9, −5.2, −5.3 ppm

Carbon attached to deuterium not observed due to quadrupolar relaxation

HRMS (*m/z*): (ESI) calculated for $C_{28}H_{46}DO_5Si_2$ $[M–H]^-$: 520.3030, found: 520.3032

IR (thin film) ν_{max}: 2929, 2856, 1710, 1472, 1425, 1275, 1259, 1108, 836, 765 and 750 cm^{-1}

$[\alpha]_D^{24}$**:** +4.0 (*c* = 1.0, $CHCl_3$)

***tert*-Butyl(((3a*R*,4*S*,5*R*,6a*S*)-5-((*tert*-butyldimethylsilyl)oxy)-3a,5,6,6a-tetrahydro-4*H*-cyclopenta[*b*]furan-4-yl-2-*d*)methoxy)dimethylsilane (60)**

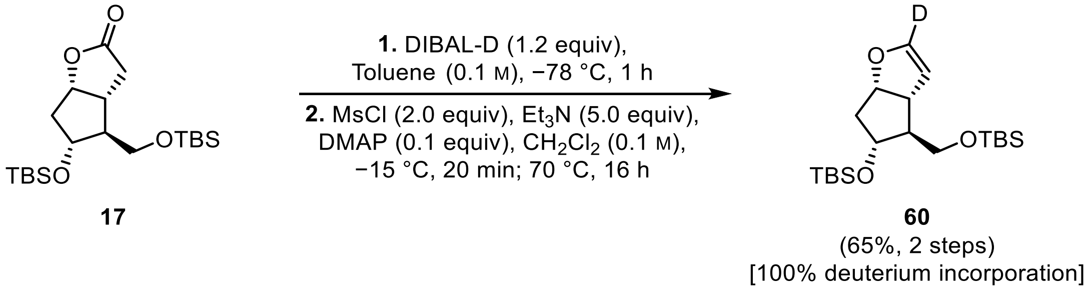

To a solution of silyl-protected Corey lactone **17** (200 mg, 0.5 mmol, 1.00 equiv) in anhydrous toluene (5.0 mL, 0.10 M) at −78 °C was added DIBAL-D (0.70 M in toluene, 0.86 mL, 0.60 mmol, 1.20 equiv) slowly dropwise (rate: 1 mL/min). The reaction mixture was then stirred at −78 °C for 1 hour before the cooling bath was removed and the reaction was immediately quenched with a saturated aqueous solution of Rochelle's salt (5.0 mL). The mixture was subsequently allowed to warm to room temperature and stirred for 16 h before transferring to a separating funnel and extracting with CH_2Cl_2 (2 × 20 mL). The organic phases were combined, washed with brine (40 mL), dried with $MgSO_4$, filtered, and concentrated under reduced pressure to afford a crude colourless oil.

The crude residue was then dissolved in anhydrous CH_2Cl_2 (5.0 mL, 0.10 M) and transferred to a flame-dried pressure flask under an atmosphere of nitrogen. To this was added Et_3N (0.35 mL, 2.50 mmol, 5.00 equiv) followed by DMAP (6.00 mg, 0.05 mmol, 0.10 equiv) and the flask was cooled to −15 °C. Methanesulfonyl chloride (MsCl, 77.0 μL, 1.00 mmol, 2.00 equiv) was added slowly dropwise and the reaction was allowed to stir at this temperature for 20 min before warming to room temperature, sealing the pressure flask, and heating to 70 °C for 16 h. The reaction was then cooled to room temperature and transferred to a separating funnel where the organic phase was washed with saturated aqueous NH_4Cl (2 × 10 mL), dried with $MgSO_4$,

filtered, and concentrated under reduced pressure to afford a crude residue. The crude product was purified by flash column chromatography (0–5% Et_2O in *n*-pentane) to afford deuterated enol ether **60** (125 mg, 65%, 100% deuterium incorporation) as a colourless oil.

TLC: R_f = 0.55 (2.5% Et_2O in *n*-pentane)

^{1}H NMR (400 MHz, Chloroform-*d*) δ 4.98 (d, *J* = 2.6 Hz, 1H), 4.76 (ddd, *J* = 10.0, 7.3, 6.0 Hz, 1H), 3.90 (ddd, *J* = 8.9, 7.7, 6.0 Hz, 1H), 3.64 (dd, *J* = 10.1, 4.0 Hz, 1H), 3.57 (dd, *J* = 10.1, 5.3 Hz, 1H), 3.01 (ddd, *J* = 10.0, 7.7, 2.6 Hz, 1H), 2.39 (ddd, *J* = 13.1, 7.3, 6.0 Hz, 1H), 1.87 – 1.70 (m, 2H), 0.89 (s, 9H), 0.87 (s, 9H), 0.04 (s, 6H), 0.04 (s, 6H) ppm

^{13}C NMR (126 MHz, Chloroform-*d*) δ 143.8 (t, *J* = 28.7 Hz),* 104.2, 83.1, 72.2, 62.4, 57.7, 46.7, 42.8, 26.1, 25.9, 18.4, 18.1, −4.4, −4.7, −5.3, −5.4 ppm

Carbon attached to deuterium marked with an asterisk (*) and appears as a 1:1:1 triplet

HRMS (*m/z*): (ESI) calculated for $C_{20}H_{40}DO_3Si_2$ $[M+H]^+$: 386.2652, found: 386.2655

IR (thin film) ν_{max}: 2955, 2894, 2857, 1586, 1472, 1257, 1111, 1060, 835, 775 and 670 cm^{-1}

$[\alpha]_D^{23}$**:** −15.1 (*c* = 0.93, $CHCl_3$)

4-((1*S*,2*R*,3a*S*,4a*R*,8a*S*,8b*S*)-2-((*tert*-butyldimethylsilyl)oxy)-1-(((*tert*-butyldimethylsilyl)oxy)methyl)-8-chloro-2,3,3a,4a,8a,8b-hexahydro-1*H*-cyclopenta[*b*]benzofuran-5-yl-4a-d)butanoic acid (23b)

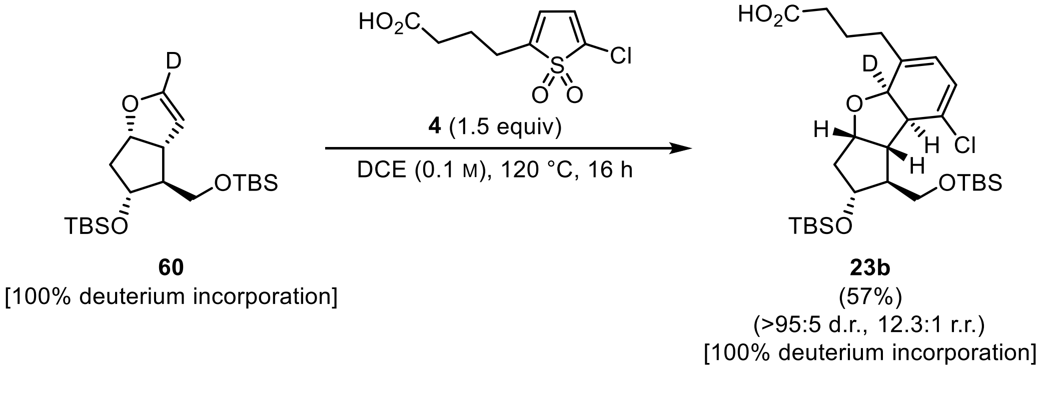

To a flame-dried microwave vial containing a solution of thiophene 1,1-dioxide **4** (83.4 mg, 0.35 mmol, 1.5 equiv) in anhydrous DCE (1.20 mL) was added 2,6-lutidine (0.13 mL, 1.15 mmol, 5.0 equiv) and the solution was stirred for 5 mins. Enol ether **60** (90.6 mg, 0.23 mmol, 1.0 equiv) in anhydrous DCE (1.20 mL) was added to the mixture. The vial was subsequently sealed, and the reaction was placed into a pre-heated oil bath at 120 °C and stirred for 16 h.

Following this, the reaction was allowed to cool to room temperature before it was diluted with CH_2Cl_2 (5 mL) followed by addition of 1 M hydrochloric acid (5 mL). The aqueous phase was extracted with CH_2Cl_2 (3 × 5 mL) and the organic phases were combined, washed with brine (5 mL), dried with Na_2SO_4, filtered, and concentrated under reduced pressure. The crude residue obtained was purified by flash column chromatography (20%–40% EtOAc in *n*-hexane) to afford chloro-diene **23b** (73 mg, 57%, >95:5 d.r., 12.3:1 r.r., 100% deuterium incorporation) as a colourless oil. Further iterations of flash column chromatography using the above conditions was used to fully separate the regioisomers for characterisation.

TLC: R_f = 0.40 (50% EtOAc in *n*-hexane)

^{1}H NMR (500 MHz, Chloroform-*d*) δ 5.96 (dd, *J* = 6.3, 1.7 Hz, 1H), 5.74 (dt, *J* = 6.3, 1.7 Hz, 1H), 4.29 (td, *J* = 7.4, 4.5 Hz, 1H), 4.04 (app. q, *J* = 6.8 Hz, 1H), 3.68 (dd, *J* = 10.1, 4.3 Hz, 1H), 3.63 (dd, *J* = 10.1, 4.3 Hz, 1H), 2.95 (t, *J* = 2.5 Hz, 1H), 2.91 (td, *J* = 7.4, 2.5 Hz, 1H), 2.41 – 2.35 (m, 2H), 2.33 – 2.21 (m, 3H), 1.91 – 1.80 (m, 3H), 1.66 (ddd, *J* = 13.7, 7.4, 4.5 Hz, 1H), 0.90 (s, 9H), 0.88 (s, 9H), 0.07 – 0.05 (m, 12H) ppm

^{13}C NMR (125 MHz, Chloroform-*d*) δ 178.7, 135.3, 134.7, 121.0, 120.5, 79.1, 73.2, 61.5, 56.1, 51.5, 49.9, 41.8, 33.8, 33.4, 26.1, 26.0, 22.7, 18.4, 18.2, −4.4, −4.7, −5.3, −5.4 ppm

Carbon attached to deuterium not observed due to quadrupolar relaxation and it being beneath the chloroform-d peaks

HRMS (*m/z*): (ESI) calculated for $C_{28}H_{47}DClO_5Si_2$ $[M–H]^-$: 556.2797, found: 556.2800

IR (thin film) ν_{max}: 2954, 2929, 2886, 2857, 1710, 1472, 1257, 1109, 882, 835, 775 and 750 cm^{-1}

$[\alpha]_D^{23}$**:** −23.0 (*c* = 2.0, $CHCl_3$)

4-((1*S*,2*R*,3a*S*,8b*S*)-2-((*tert*-Butyldimethylsilyl)oxy)-1-(((*tert*-butyldimethylsilyl)oxy)methyl)-2,3,3a,8b-tetrahydro-1*H*-cyclopenta[*b*]benzofuran-5-yl)butanoic acid (21b)

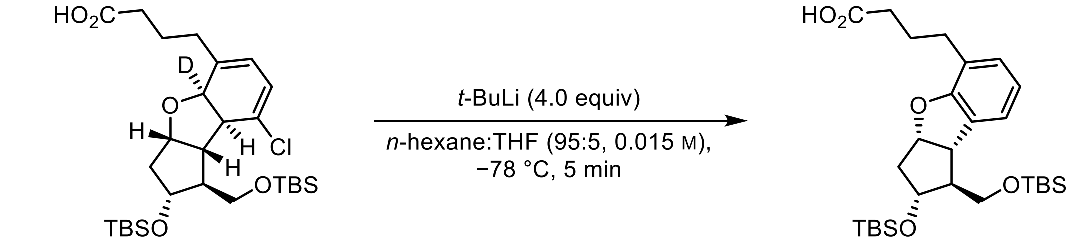

23b
[100% deuterium incorporation]

21b (55%)
[0% deuterium incorporation]

To a flame-dried Schlenk tube containing a solution of chloro-diene **23b** (27.9 mg, 0.05 mmol, 1.0 equiv) in anhydrous *n*-hexane:THF (95:5, 3.3 mL, 0.015 M) was added *t*-BuLi (1.61 M in *n*-pentane, 0.12 mL, 0.20 mmol, 4.0 equiv) at −78 °C slowly dropwise. The reaction was stirred at −78 °C for 5 mins before quenching with MeOH (0.20 mL) and warming to room temperature. The solvent was removed under reduced pressure and the crude residue was then dissolved in CH_2Cl_2 (5 mL) followed by addition of 1 M hydrochloric acid (5 mL). The aqueous phase was extracted with CH_2Cl_2 (3 × 5 mL) and the organic phases were combined, washed with brine (5 mL), dried with Na_2SO_4, filtered, and concentrated under reduced pressure. The crude residue obtained was purified by flash column chromatography (20–50% EtOAc in *n*-hexane) to afford aromatic **21b** (14.3 mg, 55%, 0% deuterium incorporation) as a colourless oil.

6. Substrate Scope

6.1 Synthesis of Halogenated Thiophene 1,1-Dioxides

2-bromo-5-methylthiophene 1,1-dioxide (42a)

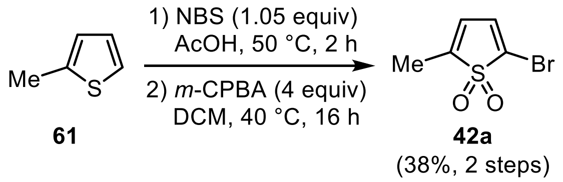

According to **General Procedure A**, 2-methylthiophene **61** (982 mg, 10.0 mmol, 1.00 equiv) was allowed to undergo bromination and oxidation to give a crude residue that was purified by flash column chromatography (20–30% EtOAc in *n*-hexane) to afford bromo-diene **42a** (798 mg, 38%) as a white solid.

MP: 53–54 °C

TLC: R_f = 0.48 (50% EtOAc in *n*-hexane)

^{1}H NMR (400 MHz, Chloroform-*d*) δ 6.77 (d, *J* = 4.7 Hz, 1H), 6.43 – 6.36 (m, 1H), 2.16 (d, *J* = 1.9 Hz, 3H) ppm

^{13}C NMR (101 MHz, Chloroform-*d*) δ 141.1, 127.8, 123.6, 119.4, 10.5 ppm

HRMS (*m/z*): (EI) calculated for $C_5H_6BrO_2S$ $[M]^+$: 207.9188, found: 207.9185

IR (thin film) ν_{max}: 3108, 3064, 1556, 1437, 1298, 1282, 1134, 1032, 841, 735, 600 and 546 cm^{-1}

2-bromo-5-ethylthiophene 1,1-dioxide (42c)

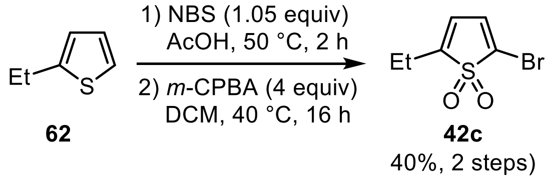

According to **General Procedure A**, 2-ethylthiophene **61** (1.12 g, 10.0 mmol, 1.00 equiv) was allowed to undergo bromination and oxidation to give a crude residue that was purified by flash column chromatography (20–30% EtOAc in *n*-hexane) to afford bromo-diene **42c** (903 mg, 40%) as a brown solid.

MP: 56–57 °C

TLC: R_f = 0.48 (50% EtOAc in *n*-hexane)

^{1}H NMR (400 MHz, Chloroform-*d*) δ 6.78 (dt, *J* = 4.9, 0.6 Hz, 1H), 6.41 – 6.34 (m, 1H), 2.57 – 2.46 (m, 2H), 1.26 (t, *J* = 7.4 Hz, 3H) ppm

^{13}C NMR (101 MHz, Chloroform-*d*) δ 146.7, 127.8, 122.1, 119.3, 18.7, 10.9 ppm

HRMS (*m/z*): (EI) calculated for $C_6H_8BrO_2S$ $[M]^+$: 221.9345, found: 221.9340

IR (thin film) ν_{max}: 3073, 2979, 1727, 1554, 1298, 1135, 1068, 844, 750, 656, 624 and 540 cm^{-1}

6.2. Inverse-Electron Demand Diels–Alder/Cheletropic Extrusion of Dihydropyrroles and Halogenated Thiophene 1,1-Dioxides

***tert*-Butyl 4,7-dibromo-2,3,3a,7a-tetrahydro-1*H*-indole-1-carboxylate (33a)**

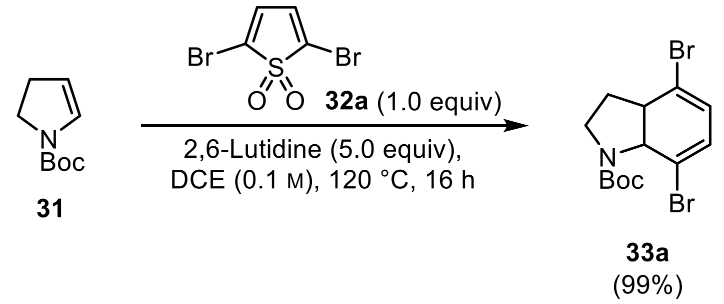

According to **General Procedure B**, *N*-Boc-2,3-dihydro-1*H*-pyrrole **31** (33.8 mg, 0.20 mmol, 1.00 equiv) was allowed to react with 2,5-dibromothiophene 1,1-dioxide **32a** (54.8 mg, 0.20 mmol, 1.00 equiv) to give a crude residue that was purified by flash column chromatography (5–10% EtOAc in *n*-hexane) to afford dibromo-diene **33a** (75 mg, 99%) as a white solid.

MP: 108–109 °C

TLC: R_f = 0.56 (20% EtOAc in *n*-hexane)

^{1}H NMR (500 MHz, Chloroform-*d*) *δ* 6.11 (dd, *J* = 6.5, 2.3 Hz, 1H), 5.97 (d, *J* = 6.5 Hz, 1H), 5.10 (br. s, 1H), 3.45 (br. s, 1H), 3.30 (ddd, *J* = 11.0, 9.5, 2.3 Hz, 1H), 2.99 – 2.89 (m, 1H), 2.40 (dtd, *J* = 12.6, 7.6, 2.3 Hz, 1H), 2.05 – 1.92 (m, 1H), 1.47 (s, 9H) ppm

^{13}C NMR (126 MHz, Chloroform-*d*) δ 154.7, 126.1, 125.5, 124.8, 123.6, 80.5, 61.5, 48.9, 43.0, 29.5, 28.5 ppm

Note: Both the ^{1}H and ^{13}C NMR were complicated by the presence of rotamers, which resulted in certain peaks having smaller shoulder peaks in the ^{13}C NMR. These smaller shoulder peaks have not been given above to simplify analysis but have been presented below in parenthesis.

^{13}C NMR (126 MHz, Chloroform-*d*) δ 154.7, 126.1, 125.5, 124.8, 123.6, 80.5 (80.2), 61.5 (61.2), 48.9 (48.3), (43.4) 43.0, (30.6) 29.5, 28.5 ppm

HRMS (*m/z*): (EI) calculated for $C_9H_9Br_2NO_2$ $[M\text{-}t\text{-}Bu+H]^+$: 320.8995, found: 320.8989

IR (thin film) ν_{max}: 2977, 2938, 2900, 1693, 1634, 1454, 1384, 1334, 1310, 1257, 1157, 1113, 1014, 933, 821 and 773 cm^{-1}

***tert*-Butyl 4,7-dichloro-2,3,3a,7a-tetrahydro-1*H*-indole-1-carboxylate (33b)**

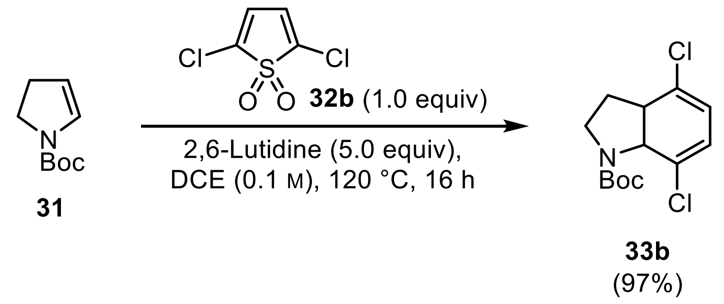

According to **General Procedure B**, *N*-Boc-2,3-dihydro-1*H*-pyrrole **31** (33.8 mg, 0.20 mmol, 1.00 equiv) was allowed to react with 2,5-dichlorothiophene 1,1-dioxide **32b** (37.0 mg, 0.20 mmol, 1.00 equiv) to give a crude residue that was purified by flash column chromatography (5–10% EtOAc in *n*-hexane) to afford dichloro-diene **33b** (56.3 mg, 97%) as a white solid.

MP: 90–91 °C

TLC: R_f = 0.56 (20% EtOAc in *n*-hexane)

^{1}H NMR (500 MHz, Chloroform-*d*) δ 5.93 (dd, J = 6.6, 2.2 Hz, 1H), 5.85 (d, J = 6.6 Hz, 1H), 5.08 (br. s, 1H), 3.43 (br. s, 1H), 3.32 (ddd, J = 10.8, 9.3, 2.4 Hz, 1H), 2.91 – 2.82 (m, 1H), 2.40 (dtd, J = 12.5, 7.5, 2.4 Hz, 1H), 2.05 – 1.93 (m, 1H), 1.47 (s, 9H) ppm

^{13}C NMR (126 MHz, Chloroform-*d*) δ 154.9, 134.2, 133.1, 120.7, 119.1, 80.3, 60.3, 47.0, 43.4, 29.6, 28.5 ppm

Note: Both the ^{1}H and ^{13}C NMR were complicated by the presence of rotamers.

HRMS (*m/z*): (EI) calculated for $C_9H_9Cl_2NO_2$ $[M\text{-}t\text{-}Bu+H]^+$: 233.0005, found: 233.0000

IR (thin film) ν_{max}: 2979, 2934, 2900, 1693, 1634, 1384, 1332, 1310, 1257, 1157, 1113, 1014, 933, 821 and 773 cm^{-1}

4,7-Dibromo-1-tosyl-2,3,3a,7a-tetrahydro-1*H*-indole (36a)

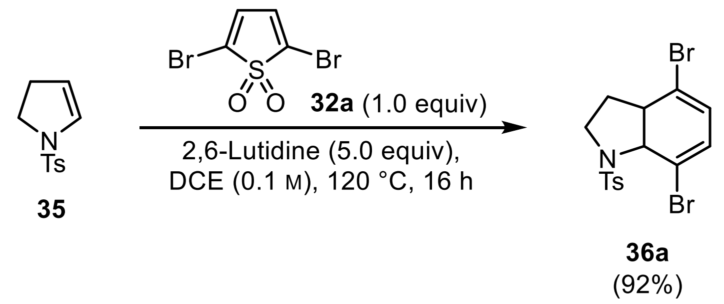

According to **General Procedure B**, *N*-Tosyl-2,3-dihydro-1*H*-pyrrole **35** (44.7 mg, 0.20 mmol, 1.00 equiv) was allowed to react with 2,5- dibromothiophene 1,1-dioxide **32a** (54.8 mg, 0.20 mmol, 1.00 equiv) to give a crude residue that was purified by flash column chromatography (10–20% EtOAc in *n*-hexane) to afford dibromo-diene **36a** (79.4 mg, 92%) as a pale yellow solid.

MP: 114–115 °C

TLC: R_f = 0.33 (20% EtOAc in *n*-hexane)

^{1}H NMR (500 MHz, Chloroform-*d*) δ 7.80 – 7.74 (m, 2H), 7.36 – 7.31 (m, 2H), 6.19 (dd, *J* = 6.6, 2.2 Hz, 1H), 5.97 (d, *J* = 6.6 Hz, 1H), 5.07 (dd, *J* = 10.0, 2.2 Hz, 1H), 3.38 (dt, *J* = 11.2, 8.1 Hz, 1H), 3.26 (ddd, *J* = 11.2, 8.5, 4.5 Hz, 1H), 2.57 (td, *J* = 10.0, 8.1 Hz, 1H), 2.45 (s, 3H), 2.23 (dtd, *J* = 12.6, 8.1, 4.5 Hz, 1H), 1.97 (ddt, *J* = 12.6, 10.0, 8.5 Hz, 1H) ppm

^{13}C NMR (126 MHz, Chloroform-*d*) δ 144.2, 135.2, 130.0, 127.9, 126.5, 124.8, 124.5, 123.7, 64.6, 48.7, 45.3, 31.7, 21.7 ppm

HRMS (*m/z*): (ESI) calculated for $C_{15}H_{15}Br_2NHO_2S$ [M+H]$^+$: 431.9273, found: 431.9263

IR (thin film) ν_{max}: 3005, 2985, 1699, 1635, 1597, 1454, 1351, 1334, 1161, 1091, 1017, 906, 856 and 708cm^{-1}

4,7-Dichloro-1-tosyl-2,3,3a,7a-tetrahydro-1*H*-indole (36b)

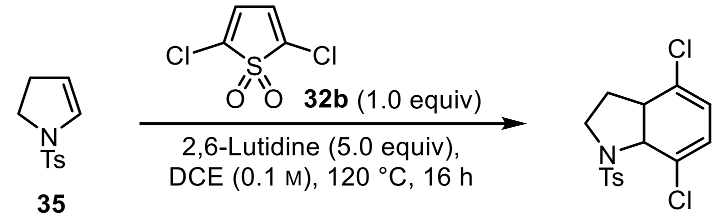

35

36b
(82%)

According to **General Procedure B**, *N*-Tosyl-2,3-dihydro-1*H*-pyrrole **35** (44.7 mg, 0.20 mmol, 1.00 equiv) was allowed to react with 2,5-dichlorothiophene 1,1-dioxide **32b** (37.0 mg, 0.20 mmol, 1.00 equiv) to give a crude residue that was purified by flash column chromatography (10–20% EtOAc in *n*-hexane) to afford dichloro-diene **36b** (56.2 mg, 82%) as a white solid.

MP: 110–111 °C

TLC: R_f = 0.33 (20% EtOAc in *n*-hexane)

^{1}H NMR (500 MHz, Chloroform-*d*) δ 7.80 – 7.71 (m, 2H), 7.37 – 7.29 (m, 2H), 5.99 (dd, *J* = 6.7, 2.0 Hz, 1H), 5.84 (dd, *J* = 6.7, 0.6 Hz, 1H), 5.02 (dd, *J* = 10.2, 2.0 Hz, 1H), 3.38 (dt, *J* = 11.0, 7.8 Hz, 1H), 3.26 (ddd, *J* = 11.0, 8.3, 4.7 Hz, 1H), 2.54 (td, *J* = 10.0, 8.0 Hz, 1H), 2.44 (s, 3H), 2.20 (dtd, *J* = 12.6, 8.0, 4.7 Hz, 1H), 2.04 – 1.94 (m, 1H) ppm

^{13}C NMR (126 MHz, Chloroform-*d*) δ 144.1, 135.2, 133.7, 131.8, 130.0, 127.8, 121.7, 119.3, 63.5, 46.7, 45.7, 31.5, 21.7 ppm

HRMS (*m/z*): (ESI) calculated for $C_{15}H_{15}Cl_2NNaO_2S$ $[M+Na]^+$: 366.0093, found: 366.0101

IR (thin film) ν_{max}: 3005, 2976, 2928, 1699, 1596, 1453, 1381, 1332, 1161, 1142, 1028, 857 and 764 cm^{-1}

***tert*-Butyl 4-bromo-7-(4-(*tert*-butoxy)-4-oxobutyl)-2,3,3a,7a-tetrahydro-1*H*-indole-1-carboxylate (38a)**

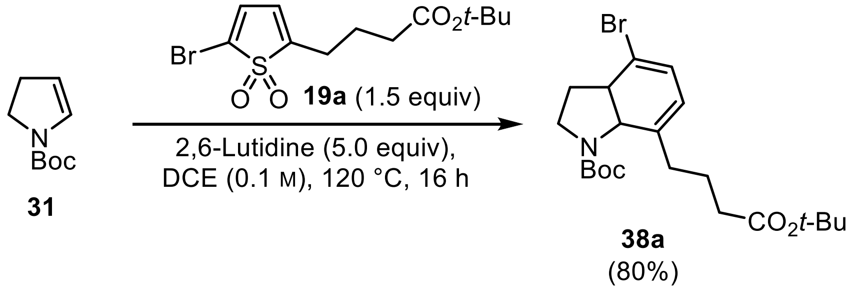

According to **General Procedure B**, *N*-Boc-2,3-dihydro-1*H*-pyrrole **31** (44.7 mg, 0.20 mmol, 1.00 equiv) was allowed to react with thiophene 1,1-dioxide **19a** (101 mg, 0.30 mmol, 1.50 equiv) to give a crude residue (8.4:1 r.r. by ^{1}H NMR) that was purified by flash column chromatography (5–10% EtOAc in *n*-hexane) to remove the minor regioisomer and afford bromo-diene **38a** (70.6 mg, 80%) as a pale yellow solid.

MP: 67–68 °C

TLC: R_f = 0.43 (20% EtOAc in *n*-hexane) for major isomer; R_f = 0.24 (20% EtOAc in *n*-hexane) for minor isomer.

^{1}H NMR (500 MHz, Chloroform-*d*) δ 6.09 (d, *J* = 6.2 Hz, 1H), 5.43 (d, *J* = 4.0 Hz, 1H), 4.95 – 4.83 (m, 1H), 3.50 – 3.26 (m, 1H), 3.18 (t, *J* = 10.1 Hz, 1H), 2.80 (dt, *J* = 17.6, 8.5 Hz, 1H), 2.28 (tt, *J* = 8.5, 4.2 Hz, 1H), 2.18 (t, *J* = 7.7 Hz, 2H), 2.06 (q, *J* = 8.3 Hz, 2H), 1.88 (d, *J* = 15.7 Hz, 1H), 1.78 – 1.61 (m, 2H), 1.44 (s, 9H), 1.41 (s, 9H) ppm

^{13}C NMR (126 MHz, Chloroform-*d*) δ 172.9, 155.3, 137.9, 124.2, 122.8, 118.1, 80.2, 79.6, 60.5, 47.2, 43.5, 35.2, 31.3, 29.9, 28.6, 28.2, 23.2 ppm

Note: Both the ^{1}H and ^{13}C NMR were complicated by the presence of rotamers. In the ^{13}C NMR, this meant that most peaks were split into two. As such, those peaks has been averaged to simplify the data above. However, all peaks without averaging have been reported below.

^{13}C NMR (126 MHz, Chloroform-*d*) δ 173.0 (172.7), 155.5 (155.2), 138.3 (137.5), 124.2, 122.9 (122.7), 118.1, 80.2 (80.1), 79.6, 60.8 (60.2), 47.5 (46.8), 43.7 (43.4), 35.2, 31.4 (31.1), 30.5 (29.3), 28.6, 28.2, 23.4 (23.0) ppm

HRMS (*m/z*): (ESI) calculated for $C_{21}H_{32}BrNNaO_4$ $[M+Na]^+$: 464.1407, found: 464.1426

IR (thin film) ν_{max}: 2975, 2931, 1728, 1693, 1478, 1455, 1389, 1365, 1252, 1159, 1112, 990, 934, 848 and 782 cm^{-1}

tert-Butyl 7-(4-(_tert_-butoxy)-4-oxobutyl)-4-chloro-2,3,3a,7a-tetrahydro-1_H_-indole-1-carboxylate (38b)

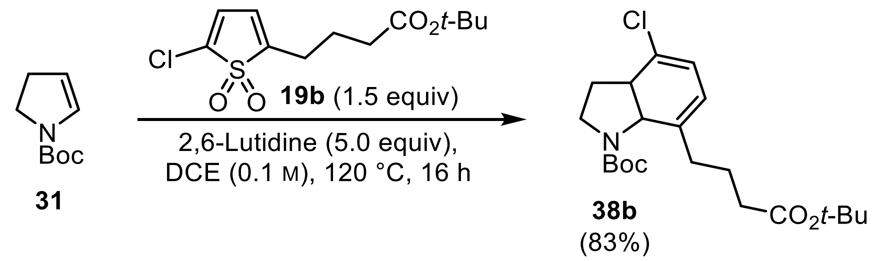

According to **General Procedure B**, *N*-Boc-2,3-dihydro-1*H*-pyrrole **31** (44.7 mg, 0.20 mmol, 1.00 equiv) was allowed to react with thiophene 1,1-dioxide **19b** (87.8 mg, 0.30 mmol, 1.50 equiv) to give a crude residue (10.2:1 r.r. by ^{1}H NMR) that was purified by flash column chromatography (5–10% EtOAc in *n*-hexane) to remove the minor regioisomer and afford chloro-diene **38b** (65.7 mg, 83%) as a pale yellow solid.

MP: 65–66 °C

TLC: R_f = 0.43 (20% EtOAc in *n*-hexane) for major isomer; R_f = 0.24 (20% EtOAc in *n*-hexane) for minor isomer.

^{1}H NMR (500 MHz, Chloroform-*d*) δ 5.88 (d, *J* = 6.3 Hz, 1H), 5.51 (dd, *J* = 6.3, 2.3 Hz, 1H), 4.93 (br. s, 1H), 3.56 – 3.28 (br. m, 1H), 3.23 – 3.15 (m, 1H), 2.69 (td, *J* = 10.6, 7.1 Hz, 1H), 2.34 – 2.25 (m, 1H), 2.24 – 2.16 (m, 2H), 2.14 – 2.03 (m, 2H), 1.92 (br. s, 1H), 1.80 – 1.62 (m, 2H), 1.46 (s, 9H), 1.42 (s, 9H) ppm

^{13}C NMR (126 MHz, Chloroform-*d*) δ 172.9, 155.4, 137.3, 132.9, 120.0, 117.6, 80.2, 79.7, 60.6, 45.4, 43.8, 35.3, 31.3, 29.7, 28.6, 28.2, 23.3 ppm

Note: Both the ^{1}H and ^{13}C NMR were complicated by the presence of rotamers. In the ^{13}C NMR, this meant that most peaks were split into two. As such, those peaks has been averaged to simplify the data above. However, all peaks without averaging have been reported below.

^{13}C NMR (126 MHz, Chloroform-*d*) δ 173.1 (172.8), 155.5 (155.2), 137.7 (137.0), 133.1 (132.8), 120.0, 117.6, 80.2, 79.7, 60.9 (60.3), 45.7 (45.0), 44.0 (43.7), 35.3, 31.4 (31.1), 30.3 (29.1), 28.6, 28.2, 23.5 (23.1) ppm

HRMS (*m/z*): (ESI) calculated for $C_{21}H_{32}ClNNaO_4$ $[M+Na]^+$: 420.1912, found: 420.1925

IR (thin film) ν_{max}: 2975, 2932, 1727, 1692, 1478, 1455, 1389, 1365, 1252, 1156, 1111, 989, 937, 872 and 776 cm^{-1}

tert-Butyl 4-(4-bromo-1-tosyl-2,3,3a,7a-tetrahydro-1*H*-indol-7-yl)butanoate (40)

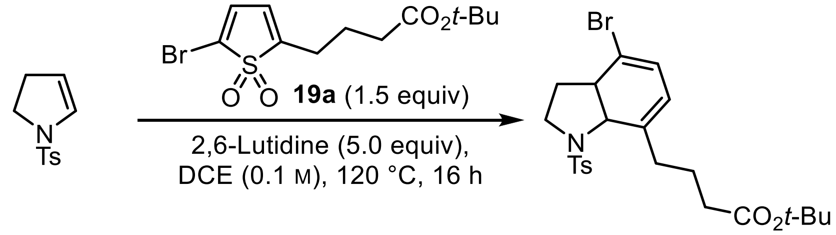

According to **General Procedure B**, *N*-Tosyl-2,3-dihydro-1*H*-pyrrole **35** (33.8 mg, 0.20 mmol, 1.00 equiv) was allowed to react with thiophene 1,1-dioxide **19a** (101 mg, 0.30 mmol, 1.50 equiv) to give a crude residue (11.5:1 r.r. by ^{1}H NMR) that was purified by flash column chromatography (10–20% EtOAc in *n*-hexane) to remove the minor regioisomer and afford diene **40** (59.5 mg, 60%) as a pale yellow solid.

MP: 68–69 °C

TLC: R_f = 0.65 (30% EtOAc in *n*-hexane) for major isomer; R_f = 0.42 (30% EtOAc in *n*-hexane) for minor isomer.

^{1}H NMR (400 MHz, Chloroform-*d*) δ 7.74 – 7.65 (m, 2H), 7.38 – 7.29 (m, 2H), 6.08 (d, *J* = 6.2 Hz, 1H), 5.52 (dq, *J* = 6.3, 1.6 Hz, 1H), 4.82 – 4.73 (m, 1H), 3.32 – 3.17 (m, 2H), 2.45 (s, 3H), 2.34 – 2.23 (m, 4H), 2.18 (td, *J* = 10.4, 7.5 Hz, 1H), 2.04 (dtd, *J* = 11.8, 7.5, 4.1 Hz, 1H), 1.93 – 1.72 (m, 3H), 1.45 (s, 9H) ppm

^{13}C NMR (126 MHz, Chloroform-*d*) δ 173.2, 144.1, 136.9, 134.6, 130.1, 127.7, 124.4, 122.2, 118.9, 80.3, 63.6, 46.9, 45.7, 35.2, 31.4, 31.1, 28.3, 23.3, 21.7 ppm

HRMS (*m/z*): (ESI) calculated for $C_{23}H_{30}BrNNaO_4S$ $[M+Na]^+$: 518.0971, found: 518.0975

IR (thin film) ν_{max}: 2975, 2926, 1724, 1597, 1455, 1348, 1251, 1159, 1092, 1004, 845, 816, 708 and 666 cm^{-1}

tert-Butyl 4-bromo-7-methyl-2,3,3a,7a-tetrahydro-1H-indole-1-carboxylate (43a)

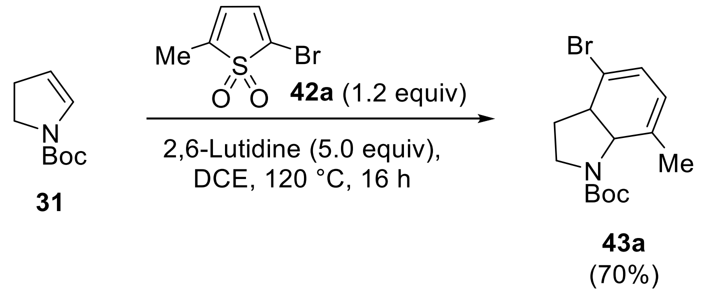

According to **General Procedure B**, *N*-Boc-2,3-dihydro-1*H*-pyrrole **31** (33.8 mg, 0.20 mmol, 1.00 equiv) was allowed to react with thiophene 1,1-dioxide **42a** (50.2 mg, 0.24 mmol, 1.20 equiv) to give a crude residue (3.6:1 r.r. by ^{1}H NMR) that was purified by flash column chromatography (5–10% EtOAc in *n*-hexane) to remove the minor regioisomer and afford diene **43a** (43.9 mg, 70%) as a pale yellow oil.

TLC: R_f = 0.50 (10% EtOAc in *n*-hexane) for major isomer; R_f = 0.30 (10% EtOAc in *n*-hexane) for minor isomer

^{1}H NMR (500 MHz, Chloroform-*d*) δ 6.07 (d, *J* = 6.3 Hz, 1H), 5.45 (d, *J* = 6.3 Hz, 1H), 4.92 – 4.78 (br. m, 1H), 3.46 – 3.28 (br. m, 1H), 3.25 (ddd, *J* = 11.1, 9.4, 1.9 Hz, 1H), 2.83 (br. q, *J* = 9.4 Hz, 1H), 2.36 – 2.27 (br. m, 1H), 1.92 (br. p, *J* = 11.1 Hz, 1H), 1.74 (t, *J* = 1.9 Hz, 3H), 1.47 (s, 9H) ppm

^{13}C NMR (126 MHz, Chloroform-*d*) δ 155.2, 134.9, 124.3, 122.6, 118.8, 80.1, 61.1, 47.4, 43.3, 29.4, 28.6, 19.6 ppm

Note: Both the ^{1}H and ^{13}C NMR were complicated by the presence of rotamers, which resulted in certain peaks having smaller shoulder peaks in the ^{13}C NMR. These smaller shoulder peaks have not been given above to simplify analysis but have been presented below in parenthesis.

^{13}C NMR (126 MHz, Chloroform-*d*) δ (155.5) 155.2, (135.7) 134.9, 124.3, 122.6 (122.3), 118.8 (118.3), 80.1 (79.6), 61.1 (60.7), 47.4 (46.8), (43.7) 43.3, (30.7) 29.4, 28.6, 19.6 ppm

HRMS (*m/z*): (ESI) calculated for $C_{14}H_{20}BrNNaO_2$ $[M+Na]^+$: 336.0570, found: 336.0563

IR (thin film) ν_{max}: 2975, 2936, 2889, 1695, 1448, 1388, 1381, 1255, 1160, 1113, 930, 861 and 767 cm^{-1}

tert-Butyl 4-bromo-7-ethyl-2,3,3a,7a-tetrahydro-1H-indole-1-carboxylate (43b)

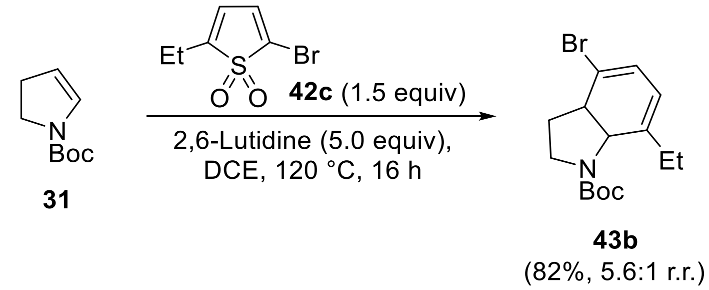

According to **General Procedure B**, *N*-Boc-2,3-dihydro-1*H*-pyrrole **31** (33.8 mg, 0.20 mmol, 1.00 equiv) was allowed to react with thiophene 1,1-dioxide **42c** (67.0 mg, 0.30 mmol, 1.50 equiv) to give a crude residue (5.6:1 r.r. by ^{1}H NMR) that was purified by flash column chromatography (5–10% EtOAc in *n*-hexane) to remove the minor regioisomer and afford diene **43b** (45.4 mg, 69%, 5.6:1 r.r.) as a pale yellow oil.

TLC: R_f = 0.50 (10% EtOAc in *n*-hexane) for major isomer; R_f = 0.30 (10% EtOAc in *n*-hexane) for minor isomer

^{1}H NMR (500 MHz, Chloroform-*d*) δ 6.13 (dd, *J* = 6.3, 0.7 Hz, 1H), 5.46 – 5.40 (m, 1H), 4.99 – 4.88 (br. m, 1H), 3.51 – 3.29 (br. m, 1H), 3.21 (ddd, *J* = 11.1, 9.3, 2.1 Hz, 1H), 2.82 (td, *J* = 10.6, 7.1 Hz, 1H), 2.36 – 2.26 (m, 1H), 2.20 – 2.01 (br. m, 2H), 2.00 – 1.85 (br. m, 1H), 1.47 (s, 9H), 1.02 (t, *J* = 7.4 Hz, 3H) ppm

^{13}C NMR (126 MHz, Chloroform-*d*) δ 155.5, 140.6, 124.4, 122.4, 116.4, 79.9, 60.8, 47.2, 43.6, 29.9, 28.6, 24.6, 12.0 ppm

Note: Both the ^{1}H and ^{13}C NMR were complicated by the presence of rotamers. In the ^{13}C NMR, this meant that most peaks were split into two. As such, those peaks has been averaged to simplify the data above. However, all peaks without averaging have been reported below.

^{13}C NMR (126 MHz, Chloroform-*d*) δ 155.6 (155.3), 141.0 (140.2), 124.4, 122.5 (122.3), 116.7 (116.2), 80.2 (79.6), 61.1 (60.6), 47.6 (46.9), 43.8 (43.4), 30.5 (29.3), 28.6, 24.6, 12.0 ppm

HRMS (*m/z*): (ESI) calculated for $C_{15}H_{22}BrNNaO_2$ $[M+Na]^+$: 350.0726, found: 350.0720

IR (thin film) ν_{max}: 2973, 2929, 2889, 1696, 1454, 1389, 1366, 1281, 1255, 1164, 1113, 932, 869, 836 and 768 cm^{-1}

6.3. Selective Dehalogenation–Aromatization using Potassium *tert*-Butoxide

tert-Butyl 4-bromoindoline-1-carboxylate (34a)

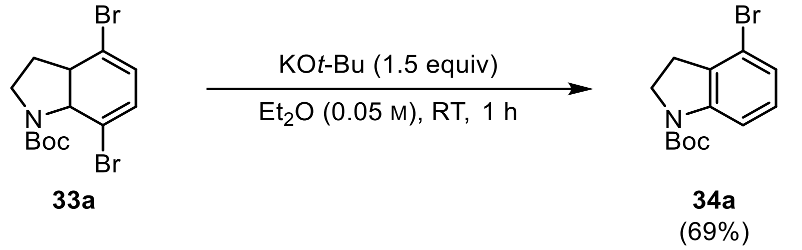

According to **General Procedure C**, dibromo-diene **33a** (37.9 mg, 0.1 mmol, 1.0 equiv) was allowed to react with KO*t*-Bu (16.8 mg, 0.15 mmol, 1.5 equiv) to give a crude residue that was purified by flash column chromatography (5–10% EtOAc in *n*-hexane) to afford 4-bromoindoline **34a** (20.5 mg, 69%) as pale yellow oil.

TLC: R_f = 0.58 (20% EtOAc in *n*-hexane)

^{1}H NMR (500 MHz, Chloroform-*d*) δ 7.90 – 7.30 (br. m, 1H), 7.08 – 7.00 (m, 2H), 3.99 (t, J = 8.8 Hz, 2H), 3.07 (t, J = 8.8 Hz, 2H), 1.56 (s, 9H) ppm

^{13}C NMR (126 MHz, Chloroform-*d*) δ 152.6, 144.2 (br.), 131.6 (br.), 129.2, 125.0, 119.6 (br.), 113.5, 81.0 (br.), 47.2, 28.9 (br.), 28.6 ppm

Note: Due to the presence of rotamers, N-Boc indolines often show a broad C-H peak at C_7 in the ^{1}H NMR and carbons near the Boc group can appear as broad peaks with low intensity or broad peaks with shoulders in the ^{13}C NMR.[17]

HRMS (*m/z*): (EI) calculated for $C_{13}H_{16}BrNO_2$ $[M]^+$: 297.0364, found: 297.0359

IR (thin film) ν_{max}: 3005, 2977, 2929, 1699, 1596, 1578, 1453, 1381, 1332, 1163, 1142, 1028, 857 and 695 cm^{-1}

Analytical data is consistent with that reported in the literature.[18]

tert-Butyl 4-chloroindoline-1-carboxylate (34b)

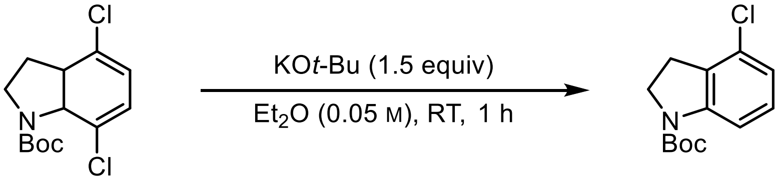

33b **34b** (65%)

According to **General Procedure C**, dichloro-diene **33b** (29.0 mg, 0.10 mmol, 1.00 equiv) was allowed to react with KO*t*-Bu (16.8 mg, 0.15 mmol, 1.50 equiv) to give a crude residue that was purified by flash column chromatography (5–10% EtOAc in *n*-hexane) to afford 4-chloroindoline **34b** (16.4 mg, 65%) as white solid.

MP: 35–36 °C

TLC: R_f = 0.58 (20% EtOAc in *n*-hexane)

^{1}H NMR (500 MHz, Chloroform-*d*) δ 7.93 – 7.28 (br. m, 1H), 7.10 (t, J = 8.1 Hz, 1H), 6.91 (dd, J = 8.1, 0.8 Hz, 1H), 4.00 (t, J = 8.8 Hz, 2H), 3.10 (t, J = 8.8 Hz, 2H), 1.56 (s, 9H) ppm

^{13}C NMR (126 MHz, Chloroform-*d*) δ 152.5, 144.4 (br.), 130.6 (br.), 129.4 (br.), 129.0, 122.2, 113.0, 81.0 (br.), 47.5, 28.6, 26.9 (br.) ppm

Note: Due to the presence of rotamers, N-Boc indolines often show a broad C-H peak at C_7 in the ^{1}H NMR and carbons near the Boc group can appear as broad peaks with low intensity or broad peaks with shoulders in the ^{13}C NMR.[17]

HRMS (*m/z*): (EI) calculated for $C_{13}H_{16}ClNO_2$ $[M]^+$: 253.0870, found: 253.0857

IR (thin film) ν_{max}: 2974, 2930, 1699, 1598, 1473, 1458, 1384, 1332, 1316, 1261, 1163, 1146, 1036, 869 and 765 cm^{-1}

6.4. Selective Detosylation–Aromatization using Potassium *tert*-Butoxide

4,7-Dibromoindoline (37a)

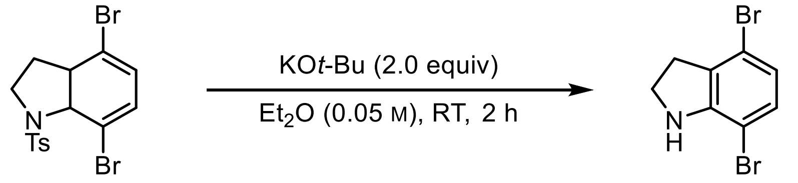

36a **37a** (61%)

According to **General Procedure C**, dibromo-diene **36a** (43.3 mg, 0.10 mmol, 1.00 equiv) was allowed to react with KO*t*-Bu (22.4 mg, 0.20 mmol, 2.00 equiv) to give a crude residue that was purified by flash column chromatography (10–20% EtOAc in *n*-hexane) to afford 4,7-dibromoindoline **37a** (16.9 mg, 61%) as a pale yellow solid.

MP: 45–46 °C

TLC: R_f = 0.40 (20% EtOAc in *n*-hexane)

^{1}H NMR (400 MHz, Chloroform-*d*) δ 7.01 (dt, *J* = 8.5, 0.8 Hz, 1H), 6.67 (d, *J* = 8.5 Hz, 1H), 4.09 (br. s, 1H), 3.67 (td, *J* = 8.6, 2.0 Hz, 2H), 3.16 (td, *J* = 8.6, 0.8 Hz, 2H) ppm

^{13}C NMR (101 MHz, Chloroform-*d*) δ 151.1, 131.4, 130.7, 122.2, 118.5, 101.5, 46.0, 32.4 ppm

HRMS (*m/z*): (ESI) calculated for $C_8H_8Br_2N$ $[M+H]^+$: 275.9018, found: 275.9012

IR (thin film) ν_{max}: 3397, 3005, 2978, 2923, 1699, 1599, 1456, 1385, 1325, 1276, 1027, 904, 882 and 706 cm^{-1}

4,7-Dichloroindoline (37b)

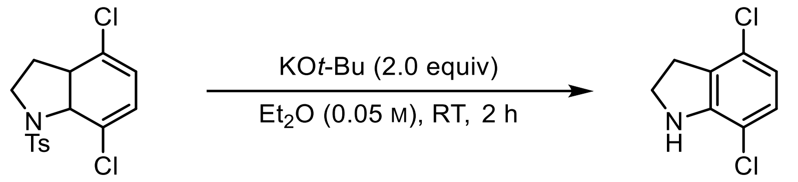

36b **37b** (59%)

According to **General Procedure C**, dichloro-diene **36b** (34.4 mg, 0.10 mmol, 1.00 equiv) was allowed to react with KO*t*-Bu (22.4 mg, 0.20 mmol, 2.00 equiv) to give a crude residue that was purified by flash column chromatography (10–20% EtOAc in *n*-hexane) to afford 4,7-dichloroindoline **37b** (11.1 mg, 59%) as a pale yellow oil.

TLC: R_f = 0.40 (20% EtOAc in *n*-hexane)

^{1}H NMR (500 MHz, Chloroform-*d*) *δ* 6.94 (dt, *J* = 8.6, 0.8 Hz, 1H), 6.58 (d, *J* = 8.6 Hz, 1H), 4.07 (br. s, 1H), 3.68 (t, *J* = 8.6 Hz, 2H), 3.15 (td, *J* = 8.6, 0.8 Hz, 2H) ppm

^{13}C NMR (126 MHz, Chloroform-*d*) *δ* 149.7, 129.0, 128.6, 128.5, 119.1, 112.9, 46.8, 30.2 ppm

HRMS (*m/z*): (ESI) calculated for $C_8H_8Cl_2N$ [M+H]$^+$: 188.0028, found: 188.0023

IR (thin film) ν_{max}: 3404, 2924, 2853, 1606, 1462, 1432, 1276, 1230, 1142, 1053, 1025, 922, 901 and 705 cm^{-1}

6.5. Dehalogenation–Aromatization of Mono-Halodienes using Potassium *tert*-Butoxide

tert-Butyl 7-(4-(*tert*-butoxy)-4-oxobutyl)indoline-1-carboxylate (39)

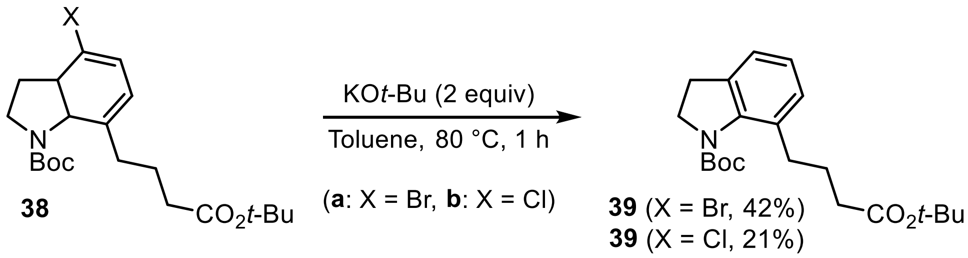

According to **General Procedure C**, halodiene **38** (**a**: X = Br, 44.2 mg, 0.10 mmol, 1.00 equiv) or (**b**: X = Cl, 39.8 mg, 0.10 mmol, 1.00 equiv) was allowed to react with sublimed grade KO*t*-Bu (99.99%, 22.4 mg, 0.20 mmol, 2.00 equiv) in toluene at 80 °C to give a crude residue that was purified by flash column chromatography (5–10% EtOAc in *n*-hexane) to afford indoline **39** (**a**: X = Br, 15.2 mg, 42%) or (**b**: X = Cl, 7.70 mg, 21%) as pale yellow oil.

TLC: R_f = 0.22 (10% EtOAc in *n*-hexane)

^{1}H NMR (400 MHz, Chloroform-*d*) δ 7.08 – 6.95 (m, 3H), 4.05 (t, *J* = 7.6 Hz, 2H), 2.95 (t, *J* = 7.6 Hz, 2H), 2.82 – 2.74 (m, 2H), 2.17 (dd, *J* = 8.0, 7.1 Hz, 2H), 1.90 – 1.78 (m, 2H), 1.51 (s, 9H), 1.43 (s, 9H) ppm

^{13}C NMR (101 MHz, Chloroform-*d*) δ 173.2, 154.0, 141.6, 134.8, 131.8, 128.6, 124.8, 122.2, 80.8, 80.1, 51.2, 35.5, 32.7, 29.8, 28.5, 28.3, 24.9 ppm

HRMS (*m/z*): (ESI) calculated for $C_{21}H_{31}NNaO_4$ [M+Na]$^+$: 384.2145, found: 384.2175

IR (thin film) ν_{max}: 2976, 2930, 1707, 1451, 1365, 1335, 1247, 1156, 1053, 1005, 911, 844, 768 and 731 cm^{-1}

6.6. Detosylation–Aromatization of Mono-Halodienes using Potassium *tert*-Butoxide

8-Bromo-2,3,6,7-tetrahydroazepino[3,2,1-*hi*]indol-4(1*H*)-one (41)

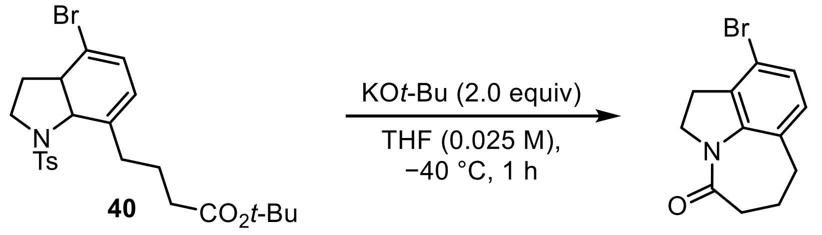

41
(44%)

According to **General Procedure C** with slight modification. To a flame-dried Schlenk tube containing bromo-diene **40** (49.6 mg, 0.10 mmol, 1.00 equiv) in THF (4.00 mL, 0.025 M) at −40 °C was added a solution of KO*t*-Bu (1 M in THF, 0.20 mL, 0.20 mmol, 2.00 equiv) slowly dropwise. The reaction stirred at this temperature for 1 h before it was warmed to room temperature and diluted with EtOAc (5 mL) followed by the 1 M hydrochloric acid (5 mL). The aqueous phase was extracted with EtOAc (3 × 5 mL) and the organic phases were combined, washed with brine (5 mL), dried with Na_2SO_4, filtered, and concentrated under reduced pressure. The crude residue obtained was purified by flash column chromatography (10–40% EtOAc in *n*-hexane) to afford aza-tricycle **41** (11.8 mg, 44%) as a white solid.

MP: 110–111 °C

TLC: R_f = 0.21 (30% EtOAc in *n*-hexane)

^{1}H NMR (500 MHz, Chloroform-*d*) δ 7.06 (d, *J* = 8.2 Hz, 1H), 6.84 (d, *J* = 8.1 Hz, 1H), 4.14 (t, *J* = 8.7 Hz, 2H), 3.04 (t, *J* = 8.7 Hz, 2H), 2.96 – 2.88 (m, 2H), 2.81 – 2.75 (m, 2H), 2.02 – 1.94 (m, 2H) ppm

^{13}C NMR (126 MHz, Chloroform-*d*) δ 172.0, 140.2, 134.2, 131.0, 125.9, 125.5, 117.7, 47.3, 38.3, 33.8, 28.2, 20.4 ppm

HRMS (*m/z*): (ESI) calculated for $C_{12}H_{13}BrNO$ $[M+H]^+$: 266.0175, found: 266.0168

IR (thin film) ν_{max}: 2926, 2874, 1623, 1586, 1447, 1422, 1372, 1344, 1187, 1144, 999 and 801cm^{-1}

7. DFT Calculations

7.1. Methods

Calculations were performed using the Gaussian09 [19] and Gaussian16 [20] programmes. Geometries were optimized with the M06-2X functional[21] and 6-311G(d,p) basis set[22] unless otherwise stated. Frontier molecular orbital calculations to obtain orbital coefficients were run at HF/6-31G.[23] Single point calculations on the cycloaddition reaction were carried out using B3LYP[24], M06-2X[21], wB97XD[25], and wB97X[25] functionals, with no, D3[26], or D3BJ[27] dispersion corrections. All calculations were carried out using the PCM model of solvation[28], with dichloroethane used as the solvent for the cycloaddition calculations and n-hexane as the solvent for the dehalogenation-aromatization calculations.[29] All structures were confirmed as stationary points with frequency calculations, and IRC calculations were run for each transition state structure to confirm that it connected the expected minima.[30] Conformational searches for all flexible structures were performed in Spartan 14.[31] Non Covalent Interaction analysis was done in MultiVFN Version 3.8,[32] according to Yang and Co-workers method.[33] Orbitals and NCI surfaces were visualised in VMD Version 1.9.4.[34] All structures are available in the .xyz file available in the SI of this paper.

7.2. Cycloaddition Selectivity Calculations

The cycloaddition reported in this work is consistently highly regioselective. This selectivity was studied computationally, in attempt to explain the chemical reasoning for the selectivity. Initial studies used a truncated diene, as shown in **Figure 3**, to decrease computational cost and conformational complexity.

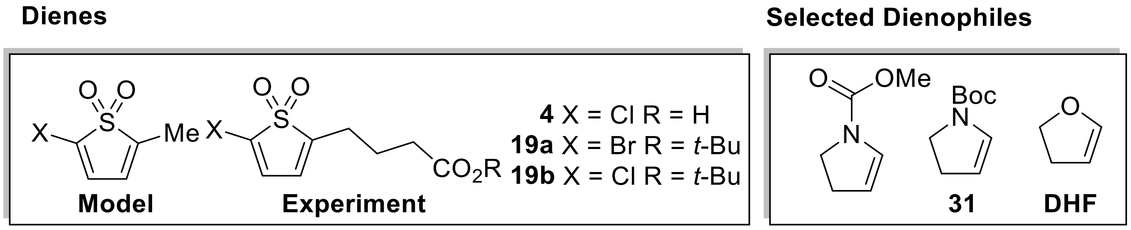

Figure 3 Model systems used in the calculations for the cycloaddition.

The energies and orbital coefficient of the frontier molecular orbitals of the model diene and N-CO_2Me-2-pyrroline (**Figure 3**) were first examined in an attempt to explain the stereochemistry of the cycloaddition seen experimentally. Frontier Molecular Orbital (FMO) analysis suggests that if orbitals from reacting compounds match in energy, and the orbital coefficients on the reacting atoms match in phase and magnitude, that reactivity can be

explained.[21] The HOMO of the dienophile and LUMO of the diene were found to be closer in energy than the dienophile LUMO/diene HOMO combinations, suggesting that these would react. While the dienophile HOMO had two lobes with distinct magnitudes, those of the diene LUMO orbital were very similar, such that a selectivity argument could not be made on this basis alone.

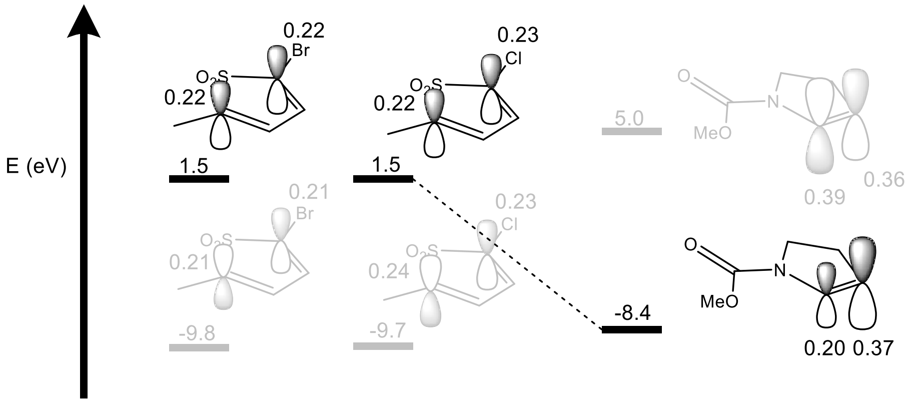

Figure 4 FMO analysis of the model cycloaddition reaction calculated at HF/6-31G. The orbital coefficients of the dienophile are not sufficiently different to indicate any regioselectivity in the reaction.

The selectivity of the cycloaddition was therefore further explored mechanistically, using the model diene **42b** shown in **Figure 3** and DHF (as an approximation of intermediate **5**) and **31** as the dienophiles. The results for DHF can be seen in **Figure 5** and **Table 8**. The *endo* TSs were found to be lower in energy than the *exo* TSs. The two *endo* transition states were found to be quite close in energy – within 0.1 kcal/mol. The experimentally observed regioselectivity is 4.4:1 **A** to **B** for the reaction of **4** and **5**. At the reaction temperature of 120 °C, this translates to roughly 1.1 kcal mol^{-1} energy difference. While the product with the observed regioselectivity (the intermediate leading to **A** after loss of SO_2) is slightly lower in energy, this again is not a large enough difference to account for the experimentally observed regioselectivities.

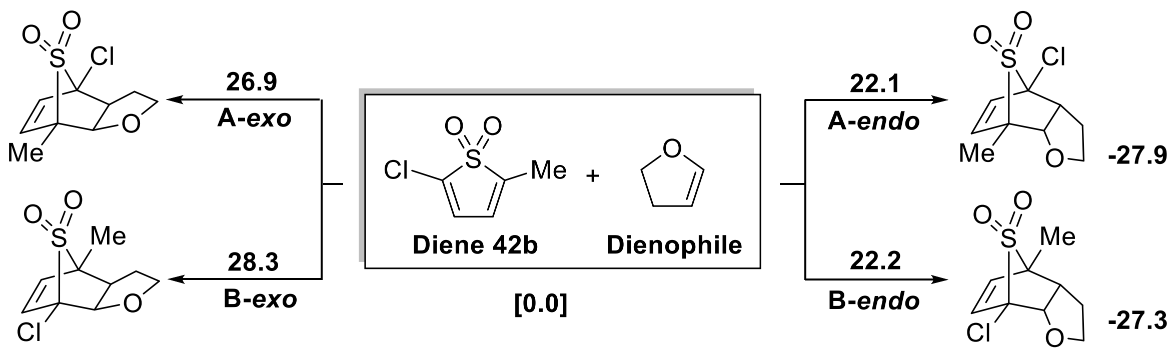

Figure 5 Energies (kcal mol^{-1}) for cycloaddition of the model diene with DHF. Energies calculated at M06-2X/6-311+G** with PCM solvation for dichloroethane.

Table 8 Energies (kcal mol^{-1}) calculated at M06-2X/6-311+G** with PCM solvation for dichloroethane for DHF dienophile.

	Electronic Energy (E)	Thermal Energy (U)	Enthalpy (H)	Free Energy (G)
SM	0.0	0.0	0.0	0.0
TS **A** *endo*	7.0	8.8	8.2	22.1
TS **B** *endo*	7.1	9.0	8.4	22.2
TS **A** *exo*	11.4	13.1	12.5	26.9
TS **B** *exo*	12.6	14.5	13.9	28.3
Int **A** *endo*	-46.9	-42.8	-43.4	-27.9
Int **B** *endo*	-46.3	-42.1	-42.7	-27.3

The calculated energies for the cycloaddition of the model diene with **31** can be seen in **Figure 6** and **Table 9**. In this case, only the *endo* pathways were calculated, as the *exo* pathways are expected to be significantly higher in energy, as observed in the DHF system. In this case, the TS leading to the experimentally observed major regioisomer **A** was 0.9 kcal mol^{-1} lower in energy than that for the minor regioisomer **B**. The intermediate leading to **A** was also 0.8 kcal mol^{-1} lower in energy than the intermediate leading to **B**. The 0.9 kcal mol^{-1} energy difference is consistent with some regioselectivity, but is below the calculated 1.8 kcal mol^{-1} energy difference which would account for the 10:1 selectivity observed experimentally, and does not fully explain the selectivity.

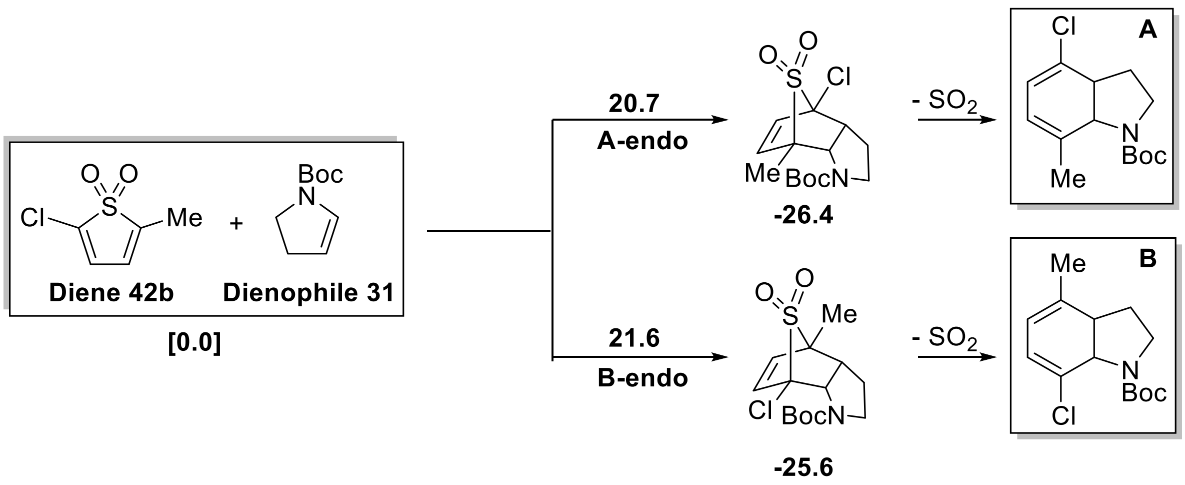

Figure 6 Energies for the cycloaddition of the model diene with **31** calculated at M06-2X/6-311+G** with PCM solvation for dichloroethane.

Table 9 Energies calculated at M06-2X/6-311+G** with PCM solvation for dichloroethane for **31** dienophile.

	E	U	H	G
SM	0.0	0.0	0.0	0.0
TS **A** *endo*	4.2	5.3	4.7	20.7
TS **B** *endo*	5.0	6.1	5.5	21.6
Int **A**	-46.8	-43.2	-43.8	-26.4
Int **B**	-45.9	-42.4	-43.0	-25.6

We theorised that London Dispersion force (LDF) in the full system may be affecting the selectivity. The *endo* TSs of the full system, using diene **19b** and dienophile **31**, were optimised with B3LYP/6-31G* with D3 dispersion correction and PCM solvation for dichloroethane, and single points with and without dispersion were calculated with several functionals to see the effect of dispersion on the transition states, and the robustness of this effect across different levels of theory, as can be seen in **Table 10**. The functional B3LYP was chosen, as it is known to be inaccurate where significant dispersion effects are at play unless corrected, as with the D3 dispersion correction. In single point calculation without dispersion (on the B3LYP-D3 geometry), the electronic energy difference between the TS **A** *endo* and TS **B** *endo* were found to be between 1.4 and 2.8 kcal mol^{-1}. Including dispersion corrections increased this difference in every case, adding between 0.6 and 3.9 kcal mol^{-1}. While this analysis cannot give a quantitative value for the impact of LDF on the selectivity, it is clearly consistent with LDF contributing to the observed selectivity.

Table 10 Single point electronic energies (kcal mol^{-1}) with a variety of functionals, comparing selectivity with and without dispersion corrections in the calculations for the lowest energy conformer of the TSS leading to each product.

	B3LYP D3	B3LYP D3BJ	B3LYP	M06-2X D3	M06-2X	wB97XD	wB97X
TS **A** *endo* Cl	0.0	0.0	0.0	0.0	0.0	0.0	0.0
TS **B** *endo* Cl	5.3	4.9	1.4	3.4	2.8	3.8	2.4

These calculations were repeated with **19a**, the X=Br diene, at B3LYP/6-31G* with GD3 dispersion correction and with PCM solvation for dichloroethane. The computed selectivity for both the X=Cl and X=Br can be seen in **Table 11**. Both systems have a free energy difference between TS **A** *endo* and TS **B** *endo* of roughly 3.5 kcal mol^{-1}, consistent with high selectivity for **A** in both cases, although suggesting even greater selectivity than seen experimentally.

Table 11 Electronic energy, internal energy, enthalpy, and free energy for each TS, relative to the lowest energy conformer for the TSS from **19b** and **19a**, respectively. Calculated at B3LYP/6-31G* with GD3 dispersion correction and with PCM solvation for dichloroethane.

	E	U	H	G
TS **A** *endo* Cl	0.0	0.0	0.0	0.0
TS **B** *endo* Cl	5.3	5.1	5.1	3.8
TS **A** *endo* Br	0.0	0.0	0.0	0.0
TS **B** *endo* Br	5.2	5.1	5.1	3.5

This is consistent with dispersion playing a role in the strong selectivity of the experimental system, despite the selectivity being overestimated by the dispersion corrected calculations, and this was confirmed experimentally. In order to visualise the attractive dispersion force, noncovalent interactions (NCI) were also calculated for TS **A** *endo* Cl.[31] In the NCI plot for the region of the -$CH_2CH_2CH_2CO_2$tBu chain on the diene interacting with the Boc group in the dienophile, the green surface indicates weakly attractive Van der Waals forces between the *tert*-butyl ester and alkyl chain of the diene and the *tert*-butyl ester of the dienophile.

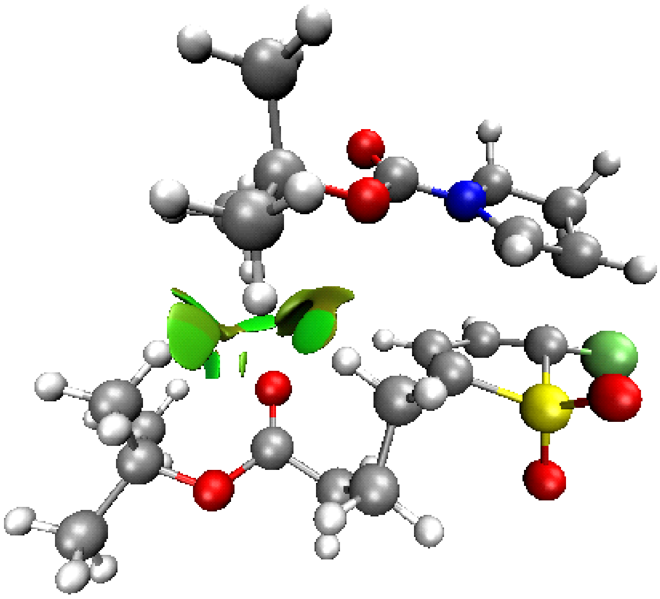

Figure 7 NCI for the TS leading to product **A**, for the non-reactive region. Green coloured surfaces indicate weakly attractive Van der Waals interactions, such as London Dispersion.

5.3 Dehalogenation–Aromatization Calculations

The Dehalogenation-Aromatization step was also studied with DFT. Based on deuterium-labelling studies and DFT calculations using M06-2X/6-311G(d,p) with PCM [*n*-hexane], the mechanism of dehalogenation–aromatization has been proposed to occur through a complex-induced proximity effect (CIPE) (directed deprotonation of **25** through **26** to give **27**/**28**) followed by α-elimination (**28** to **29**) and a 1,2-hydrogen shift (**29** to **30**) (**Scheme 1**). Using DFT calculations, the energy barrier for the resultant 1,2-hydrogen shift of carbene **29** to aromatized product **30** was found to be almost barrierless at 1 kcal mol^{-1}.

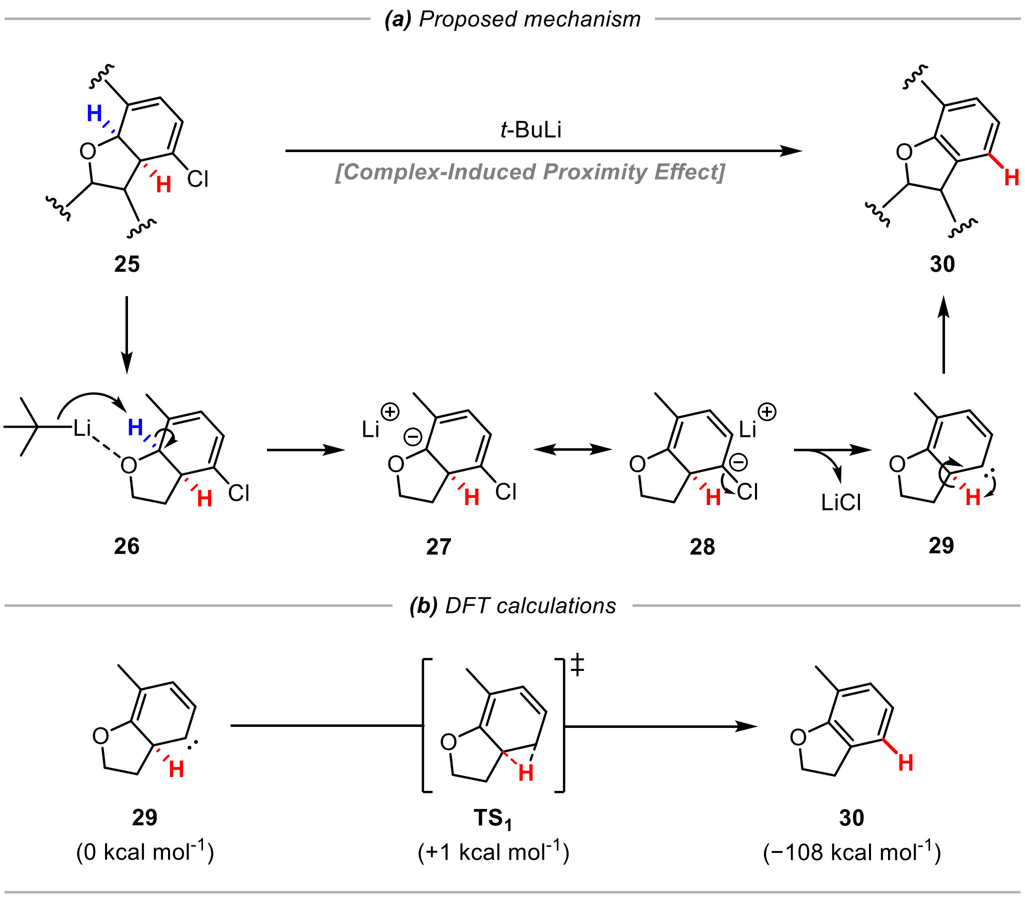

Scheme 1. (a) Proposed mechanism of the dehalogenation–aromatization reaction with *t*-BuLi. **(b)** DFT calculated energy levels using M06-2X/6-311G(d,p) with PCM [*n*-hexane].

8. NMR Spectra (with Table of Contents)

8. NMR Spectra (with Table of Contents) .... - 81 -

8.1. The Total Synthesis of Beraprost .... - 82 -

8.2. Dehalogenation–Aromatization Model Studies .... - 100 -

8.3. Deuterium-Labelling Studies .... - 111 -

8.4. Substrate Scope .... - 117 -

9. References .... - 135 -

8.1. The Total Synthesis of Beraprost

Compound 4:

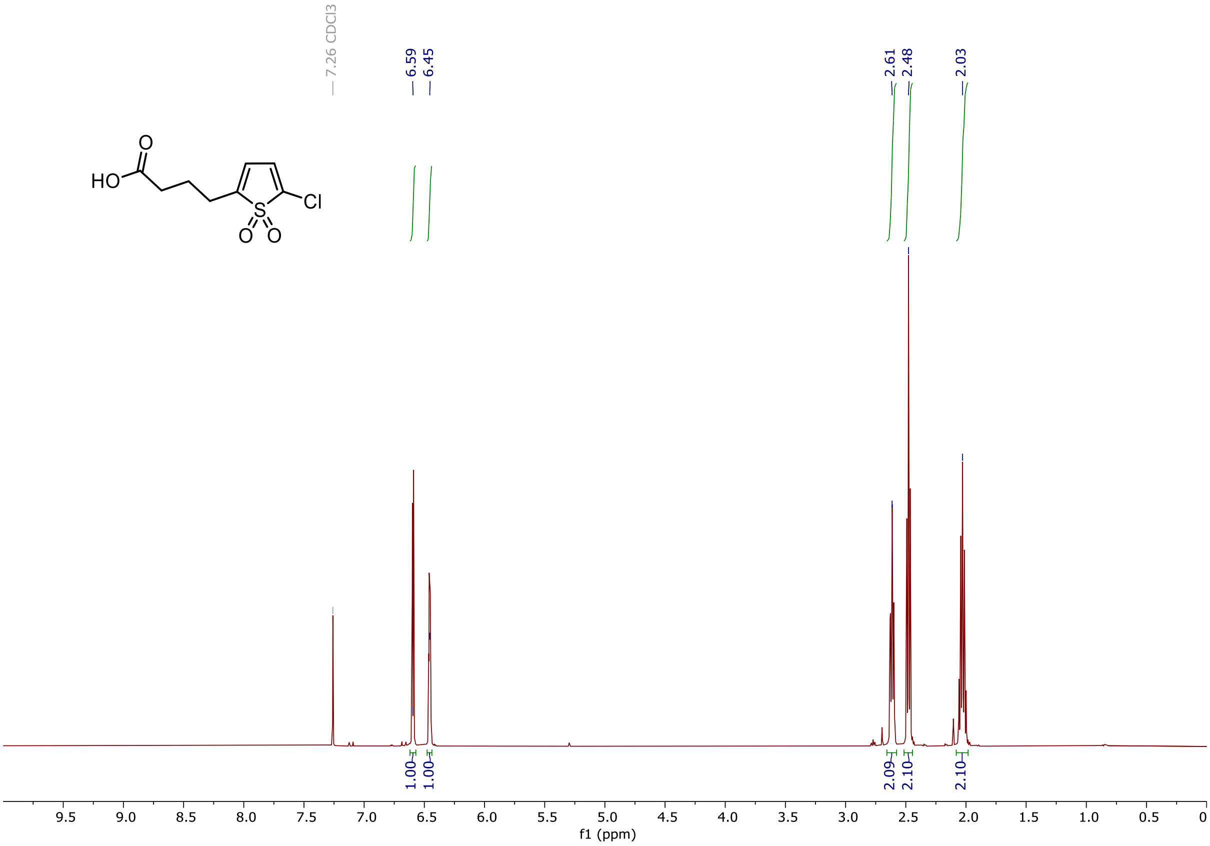

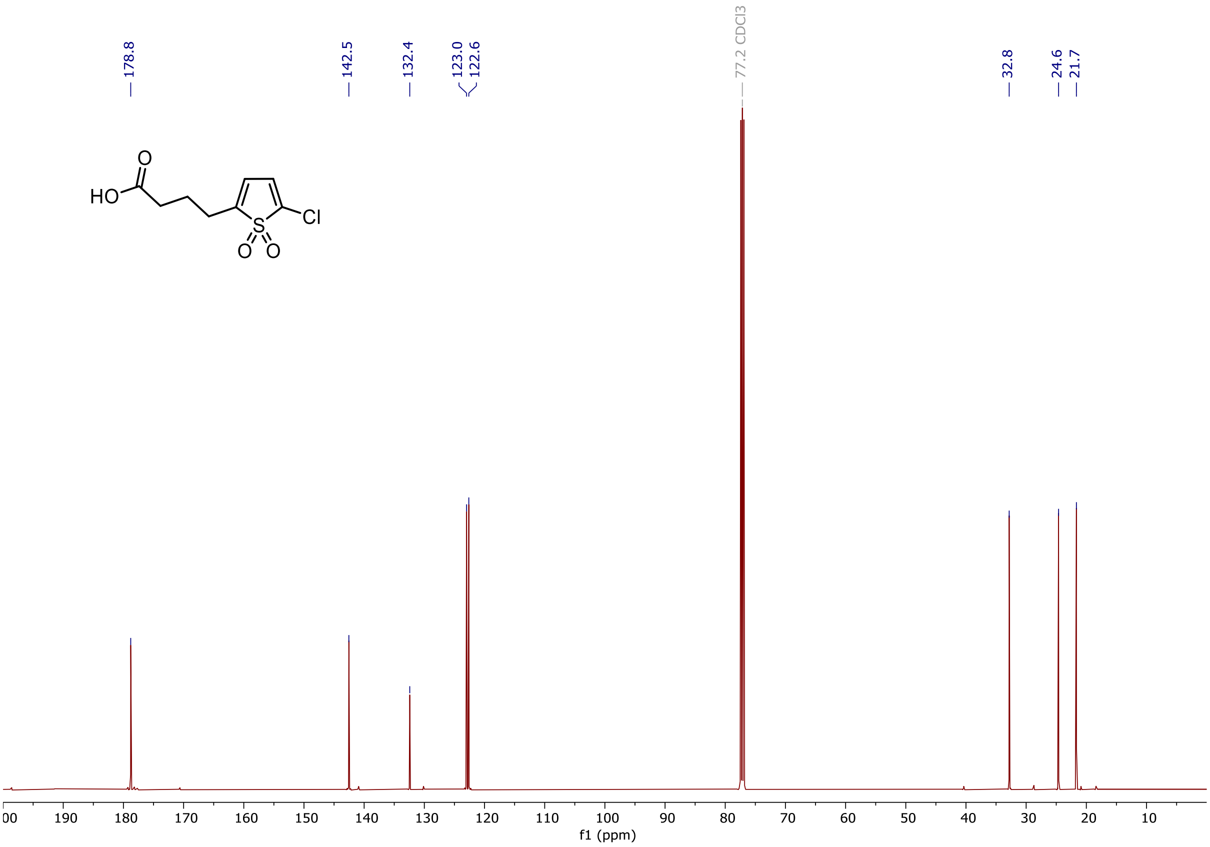

Compound 10:

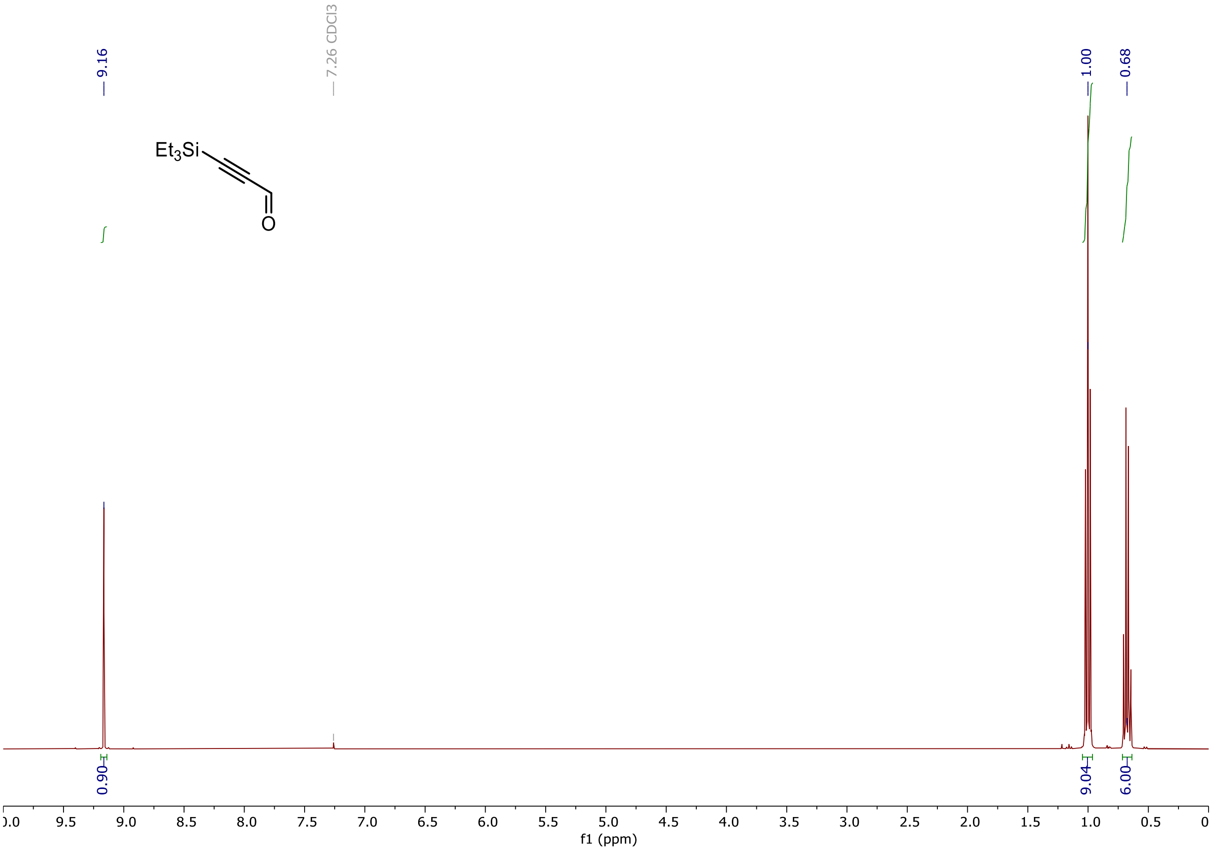
9.16
7.26 CDCl3
1.00
0.68
Et3Si
O
0.90
9.04
6.00
f1 (ppm)

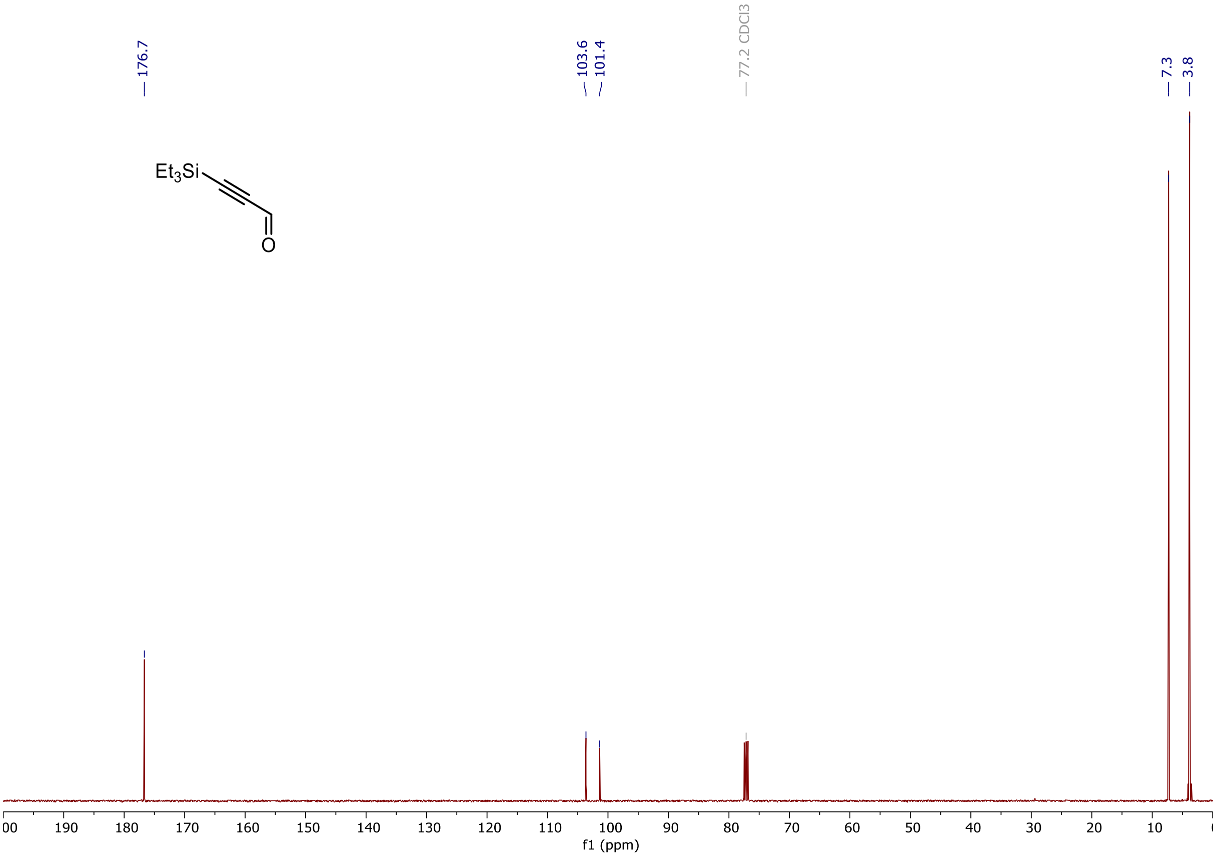
176.7
103.6
101.4
77.2 CDCl3
7.3
3.8
Et3Si
O
f1 (ppm)

Compound 12:

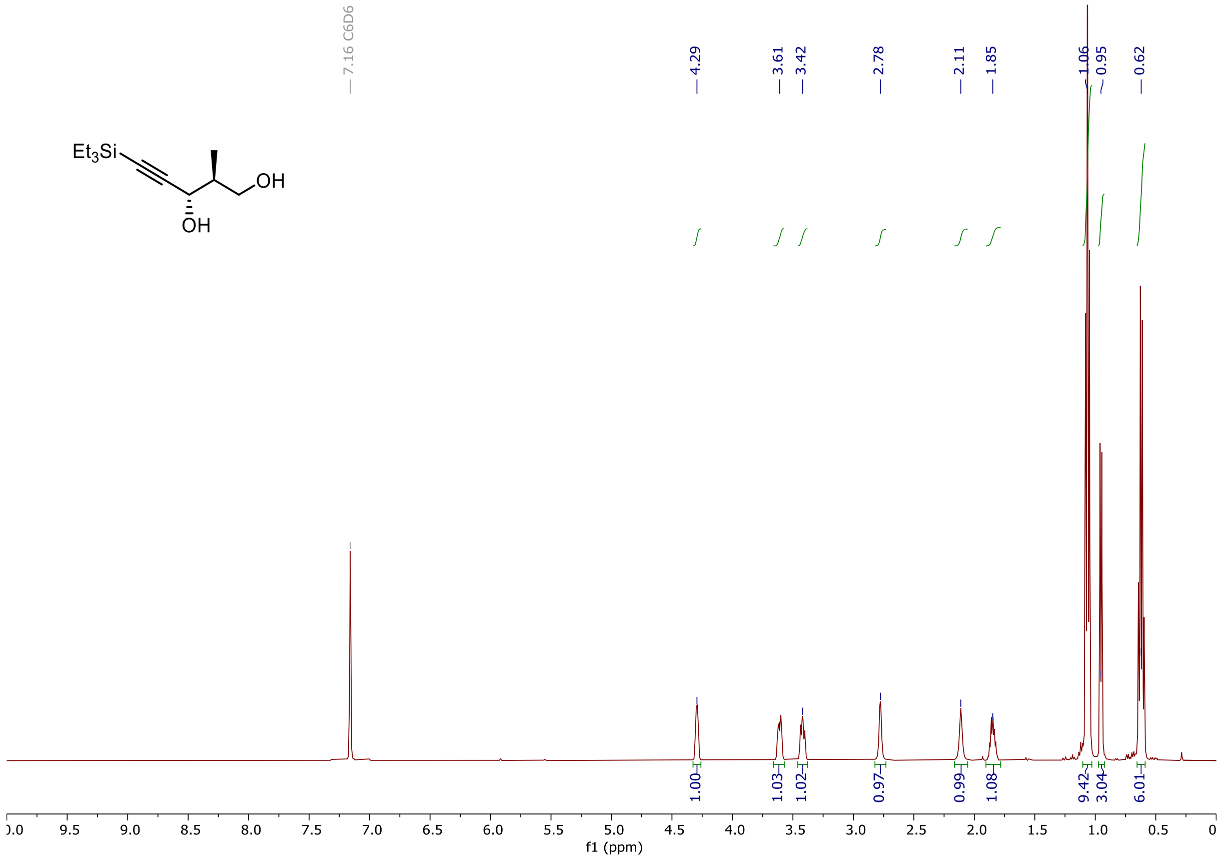

7.16 C6D6
4.29
3.61
3.42
2.78
2.11
1.85
1.06
0.95
0.62
Et3Si
OH
OH
1.00
1.03
1.02
0.97
0.99
1.08
9.42
3.04
6.01
f1 (ppm)

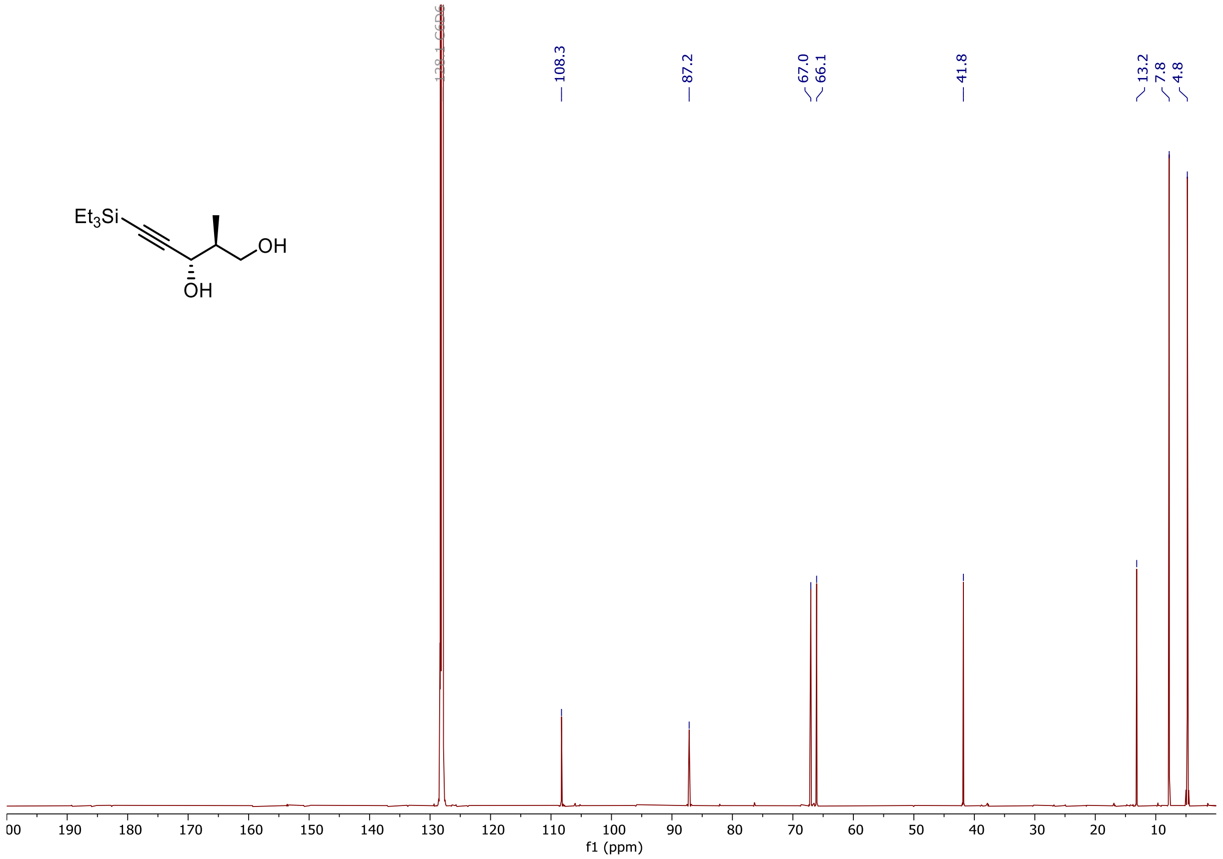

108.3
87.2
67.0
66.1
41.8
13.2
7.8
4.8
Et3Si
OH
OH
f1 (ppm)

Compound 45:

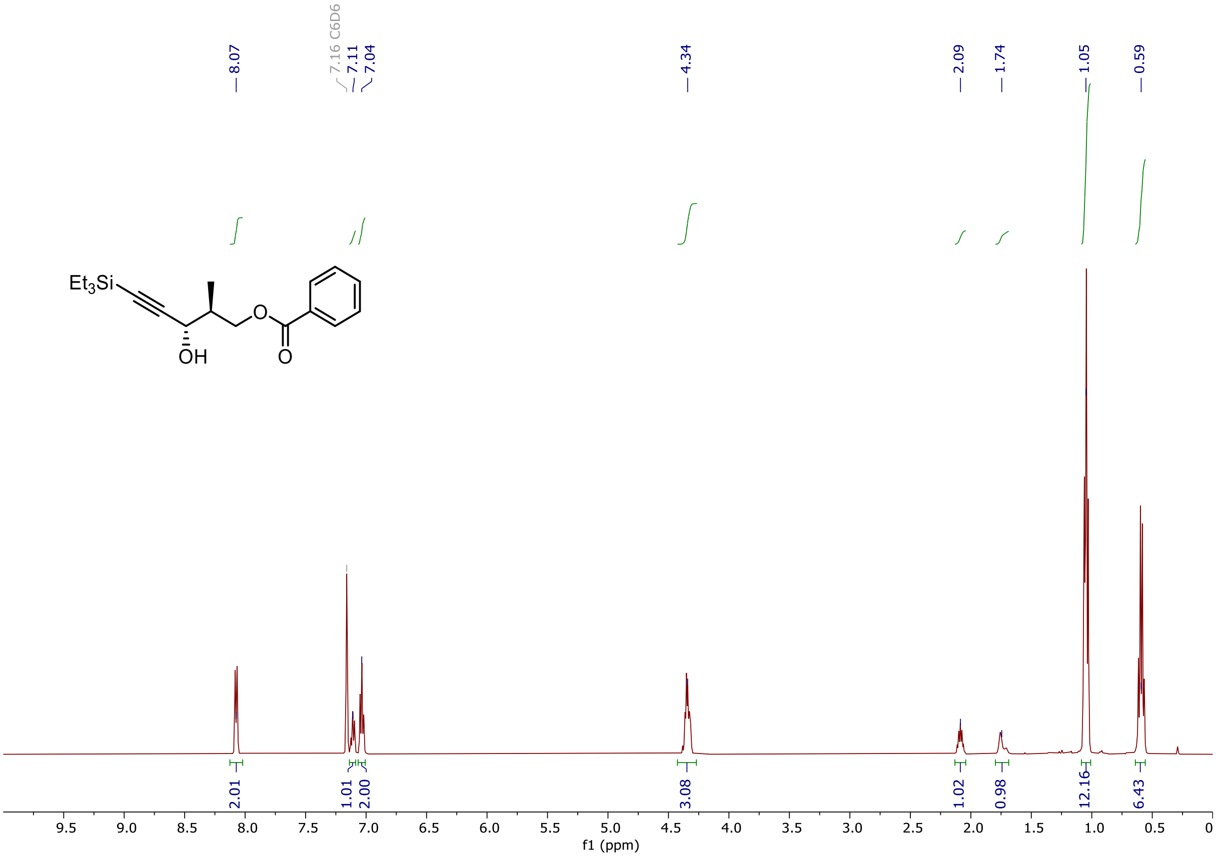
7.16 C6D6
8.07
7.11
7.04
4.34
2.09
1.74
1.05
0.59
Et3Si
OH
O
O
2.01
1.01
2.00
3.08
1.02
0.98
12.16
6.43
f1 (ppm)

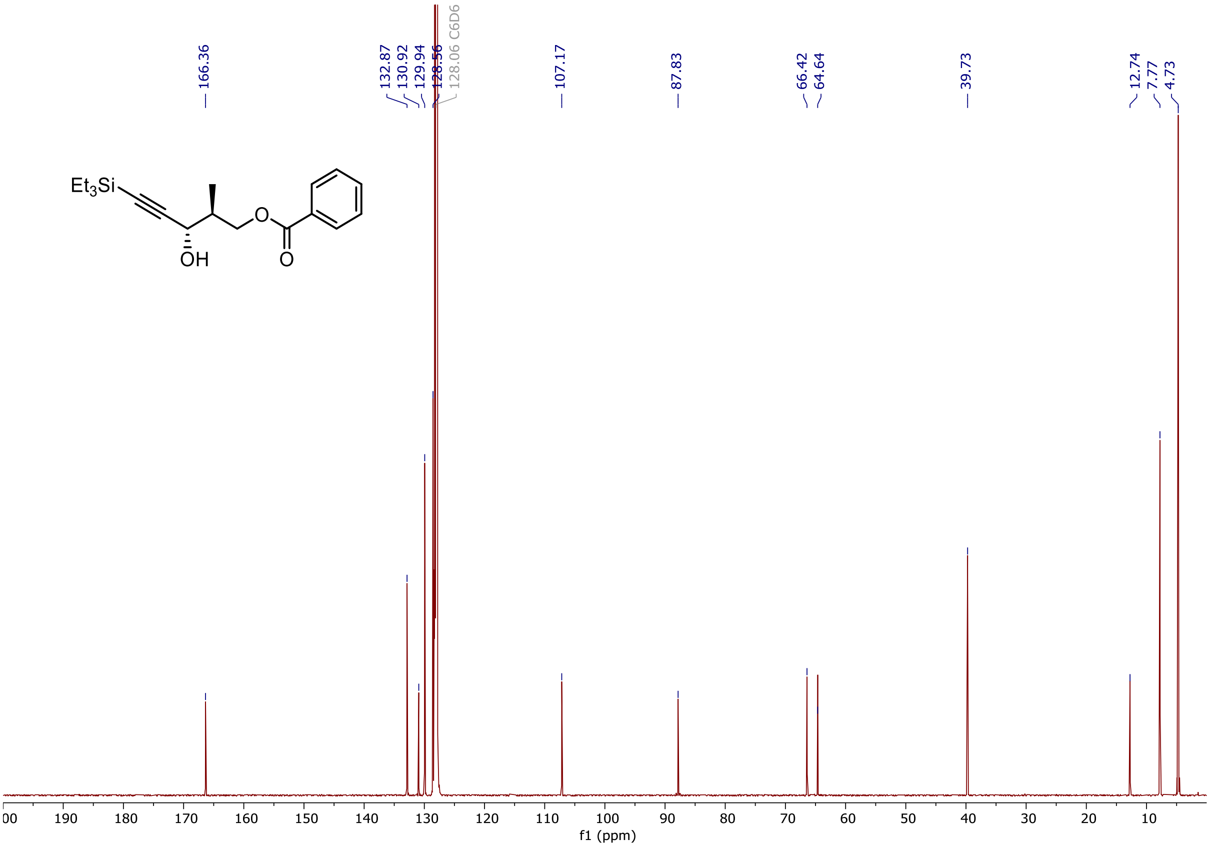
166.36
132.87
130.92
129.94
128.56
128.06 C6D6
107.17
87.83
66.42
64.64
39.73
12.74
7.77
4.73
Et3Si
OH
O
O
f1 (ppm)

Compound 46:

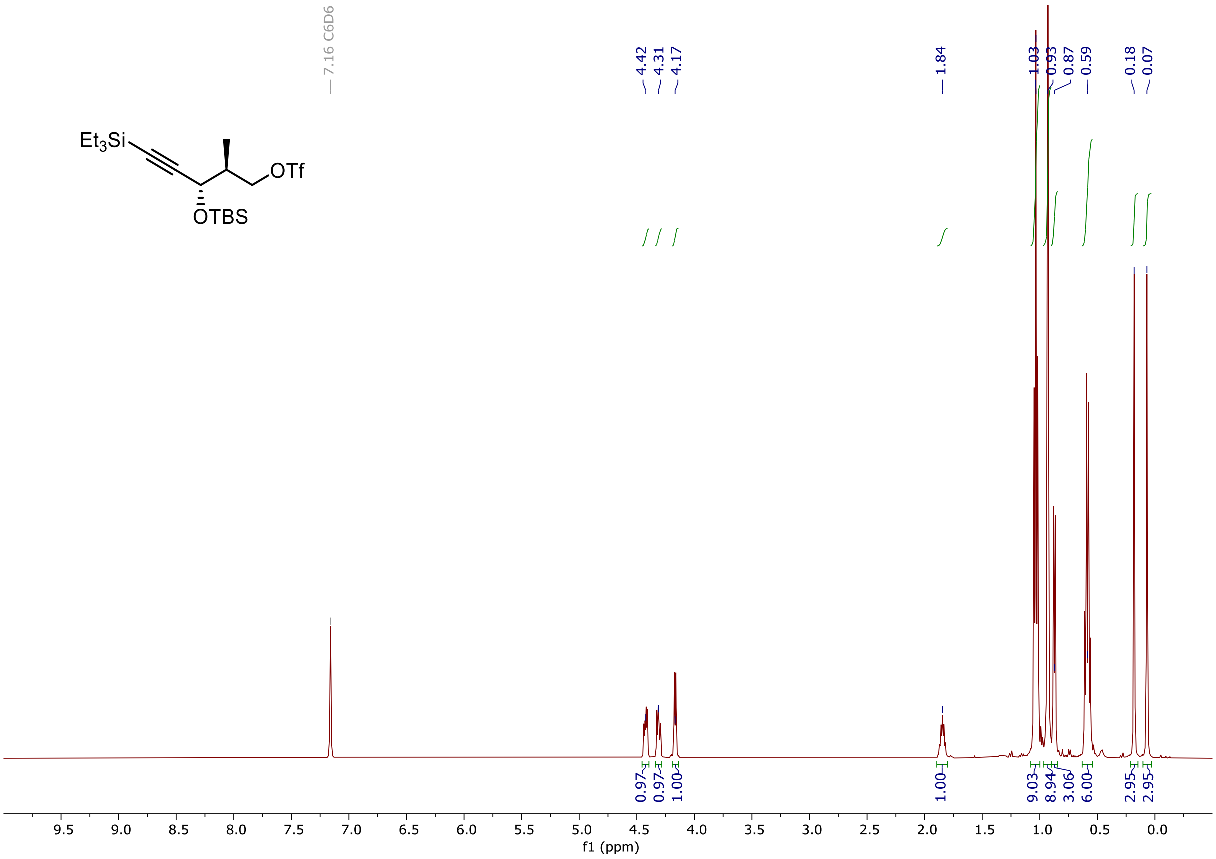

7.16 C6D6
4.42
4.31
4.17
1.84
1.03
0.93
0.87
0.59
0.18
0.07
Et3Si
OTf
OTBS
0.97
0.97
1.00
1.00
9.03
8.94
3.06
6.00
2.95
2.95
f1 (ppm)

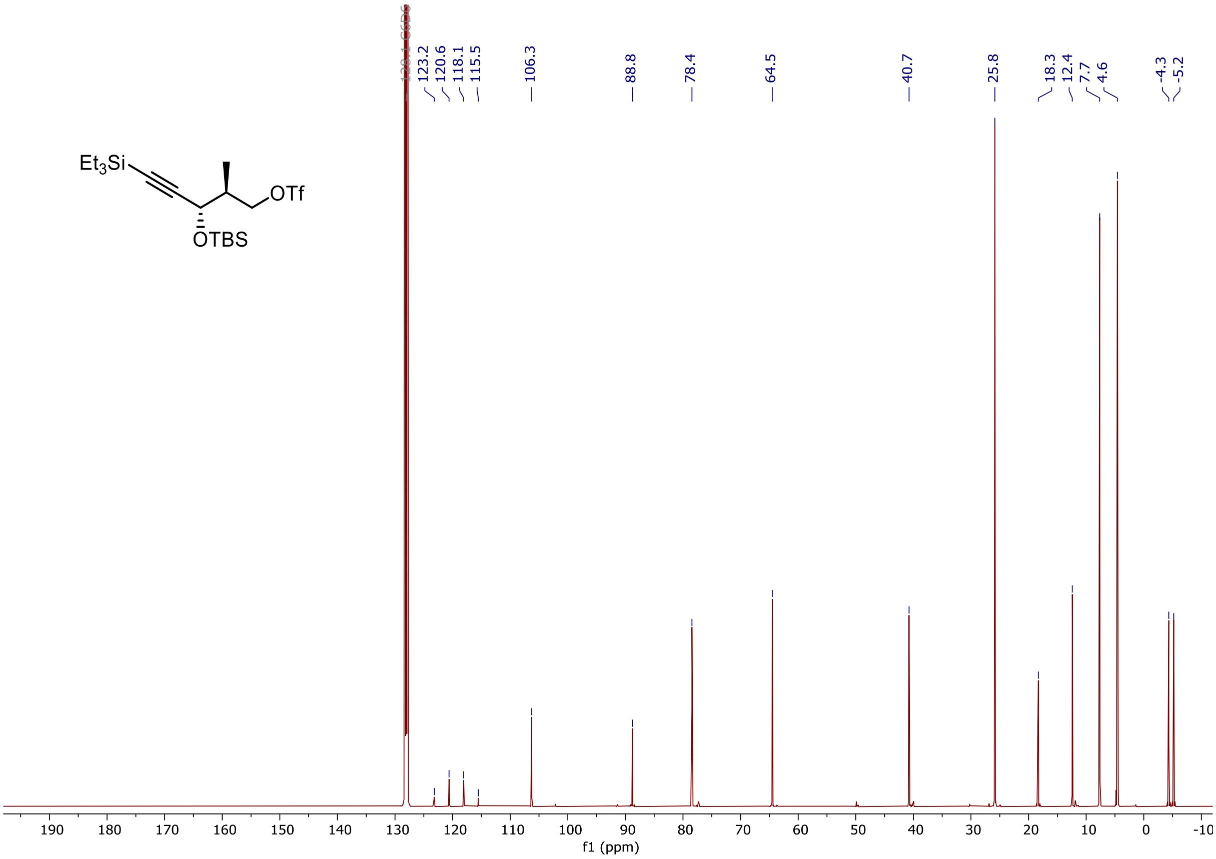

123.2
120.6
118.1
115.5
106.3
88.8
78.4
64.5
40.7
25.8
18.3
12.4
7.7
4.6
-4.3
-5.2
Et3Si
OTf
OTBS
f1 (ppm)

Compound 13:

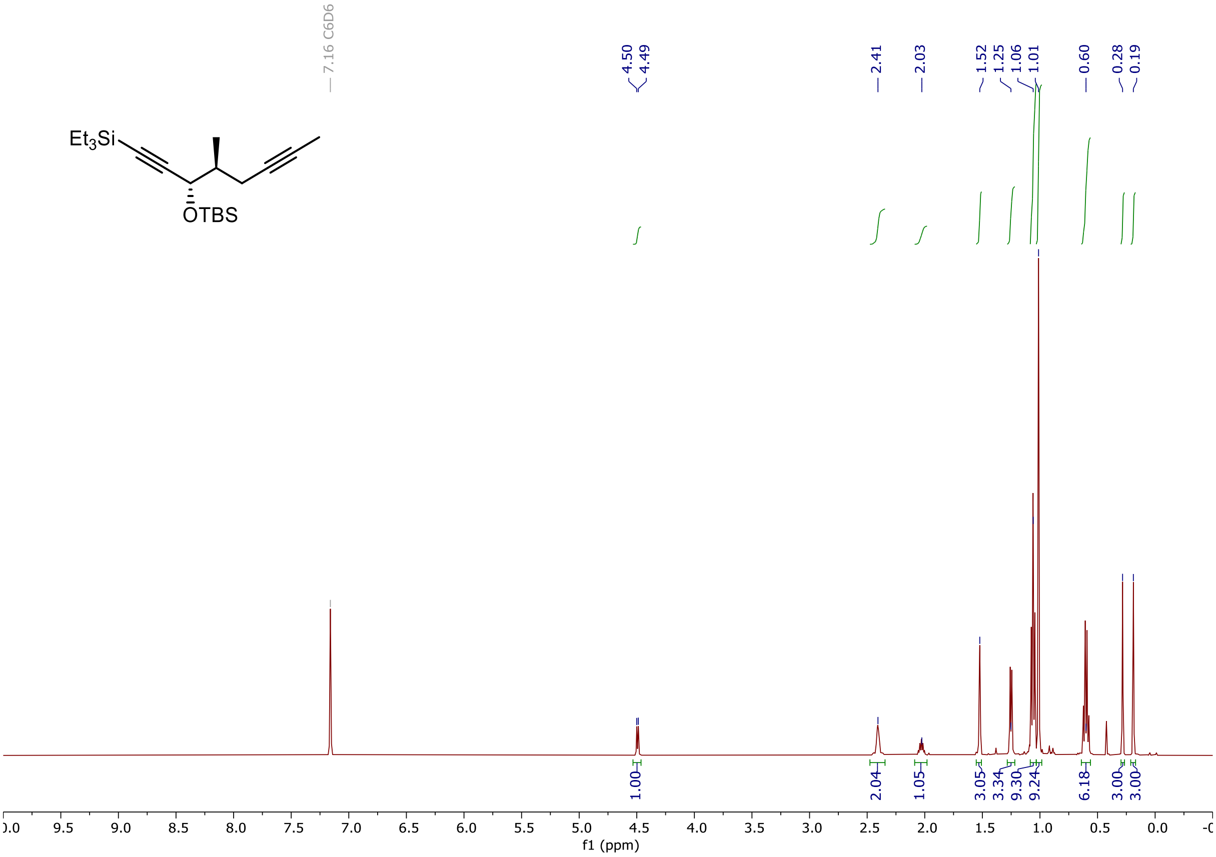
7.16 C6D6
4.50
4.49
2.41
2.03
1.52
1.25
1.06
1.01
0.60
0.28
0.19
Et3Si
OTBS
1.00
2.04
1.05
3.05
3.34
9.30
9.24
6.18
3.00
3.00
f1 (ppm)

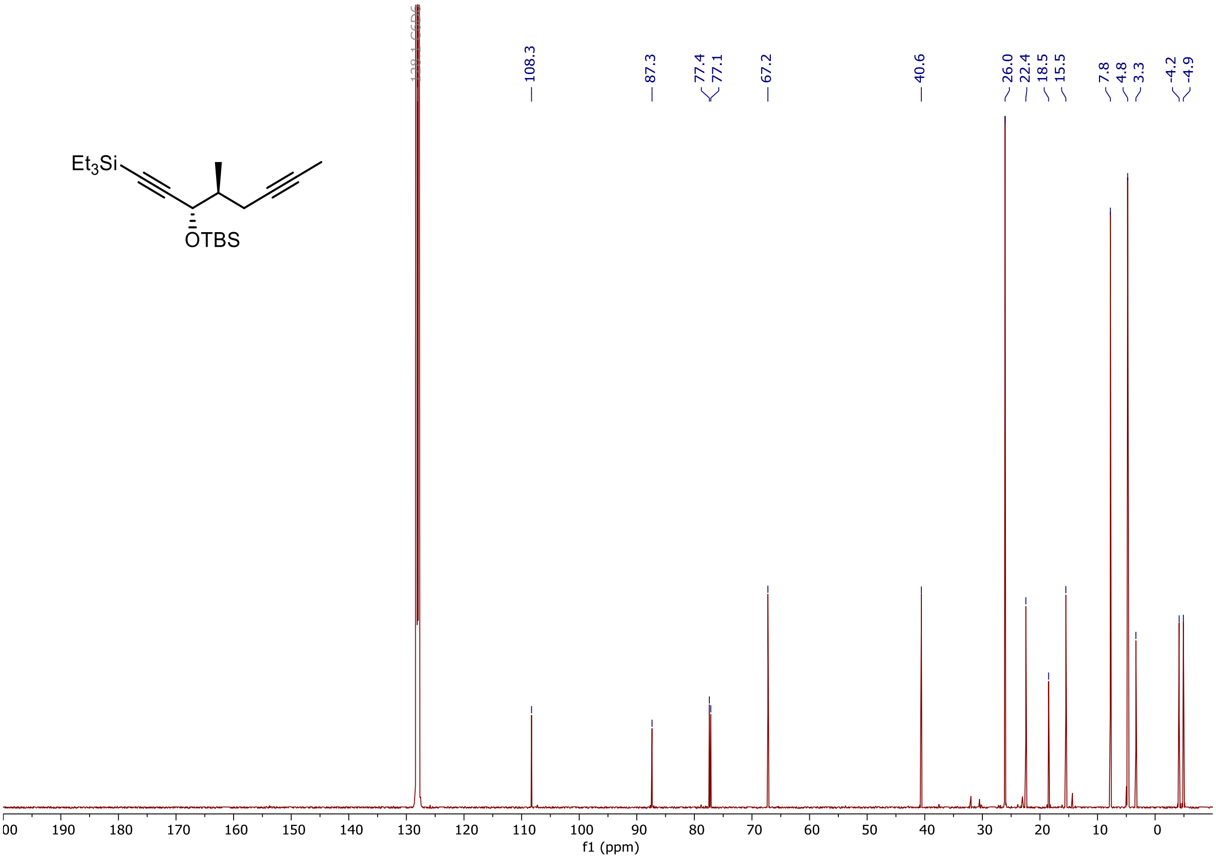
108.3
87.3
77.4
77.1
67.2
40.6
26.0
22.4
18.5
15.5
7.8
4.8
3.3
-4.2
-4.9
Et3Si
OTBS
f1 (ppm)

Compound 47:

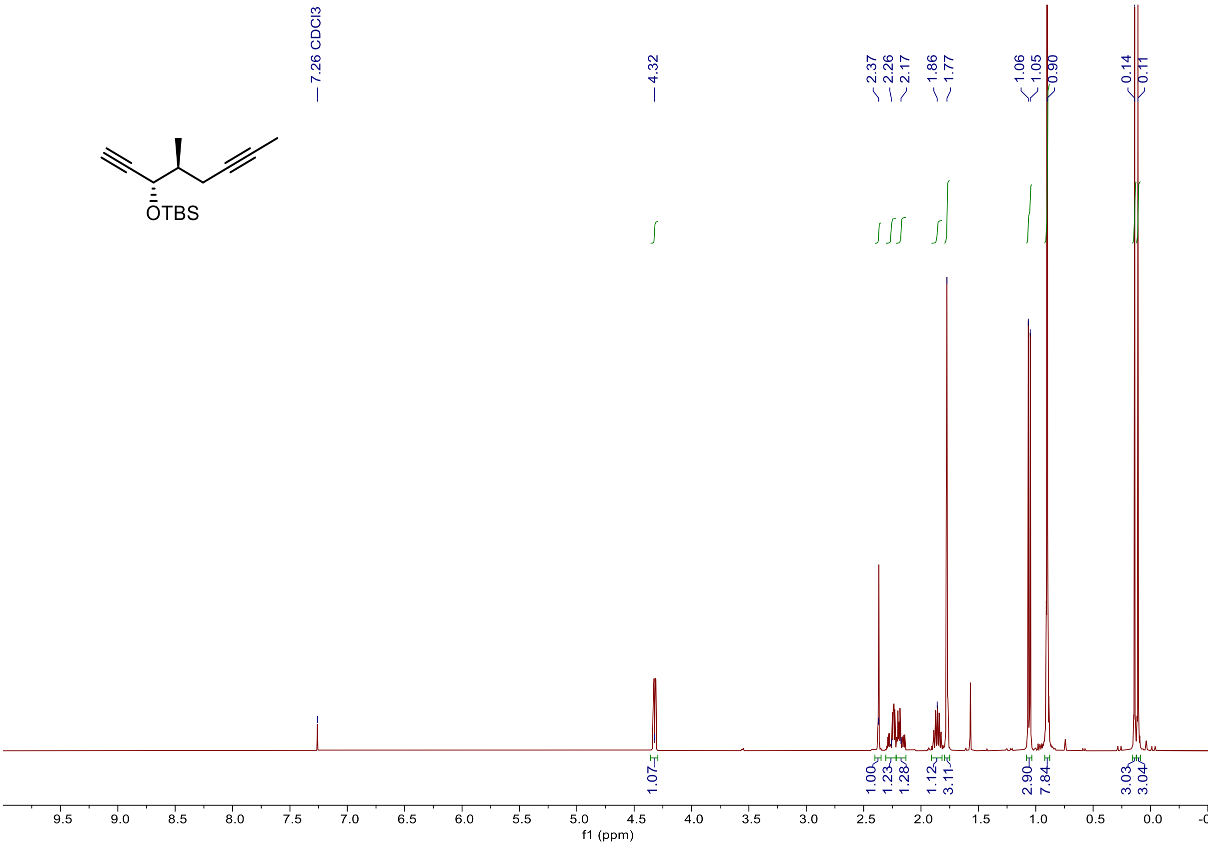

OTBS
7.26 CDCl3
4.32
2.37
2.26
2.17
1.86
1.77
1.06
1.05
0.90
0.14
0.11
1.07
1.00
1.23
1.28
1.12
3.11
2.90
7.84
3.03
3.04
f1 (ppm)

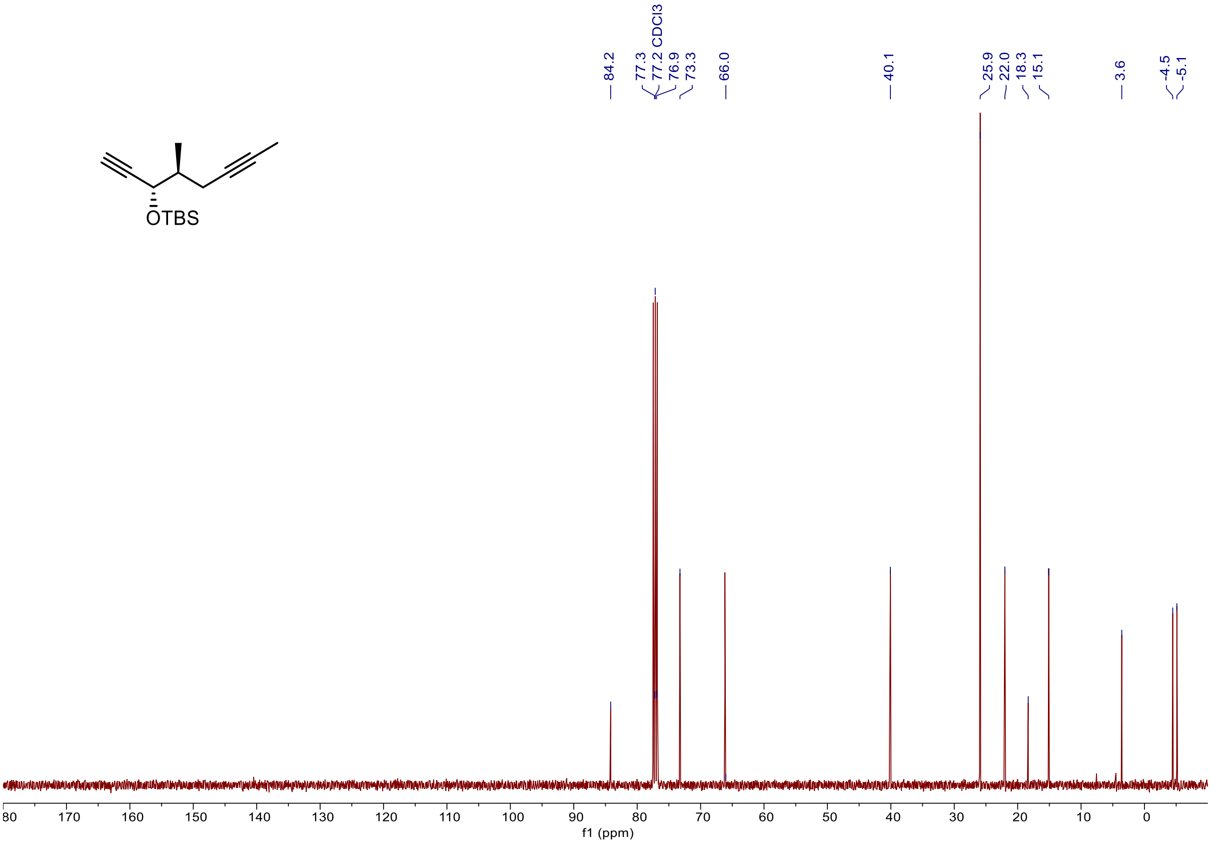

OTBS
84.2
77.3
77.2 CDCl3
76.9
73.3
66.0
40.1
25.9
22.0
18.3
15.1
3.6
-4.5
-5.1
f1 (ppm)

Compound 6:

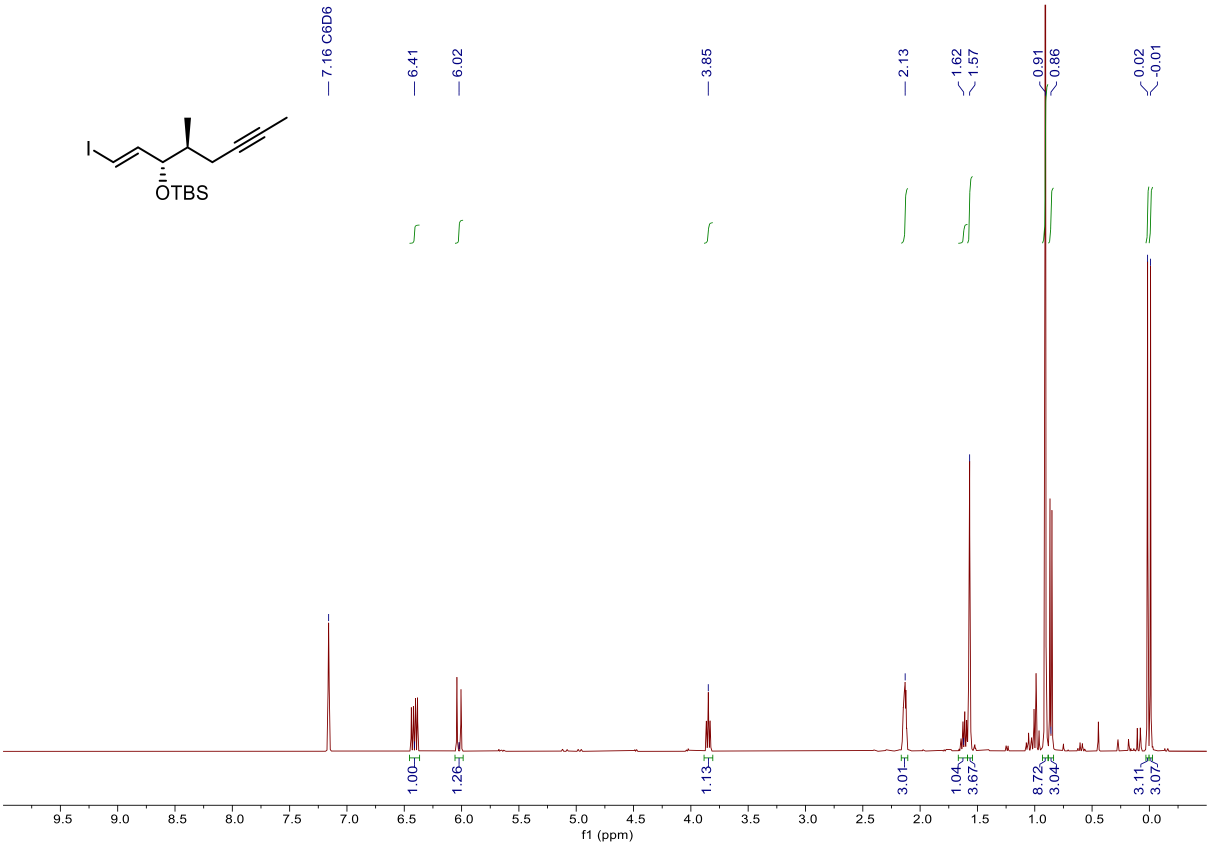

7.16 C6D6
6.41
6.02
3.85
2.13
1.62
1.57
0.91
0.86
0.02
-0.01
OTBS
1.00
1.26
1.13
3.01
1.04
3.67
8.72
3.04
3.11
3.07
9.5 9.0 8.5 8.0 7.5 7.0 6.5 6.0 5.5 5.0 4.5 4.0 3.5 3.0 2.5 2.0 1.5 1.0 0.5 0.0
f1 (ppm)

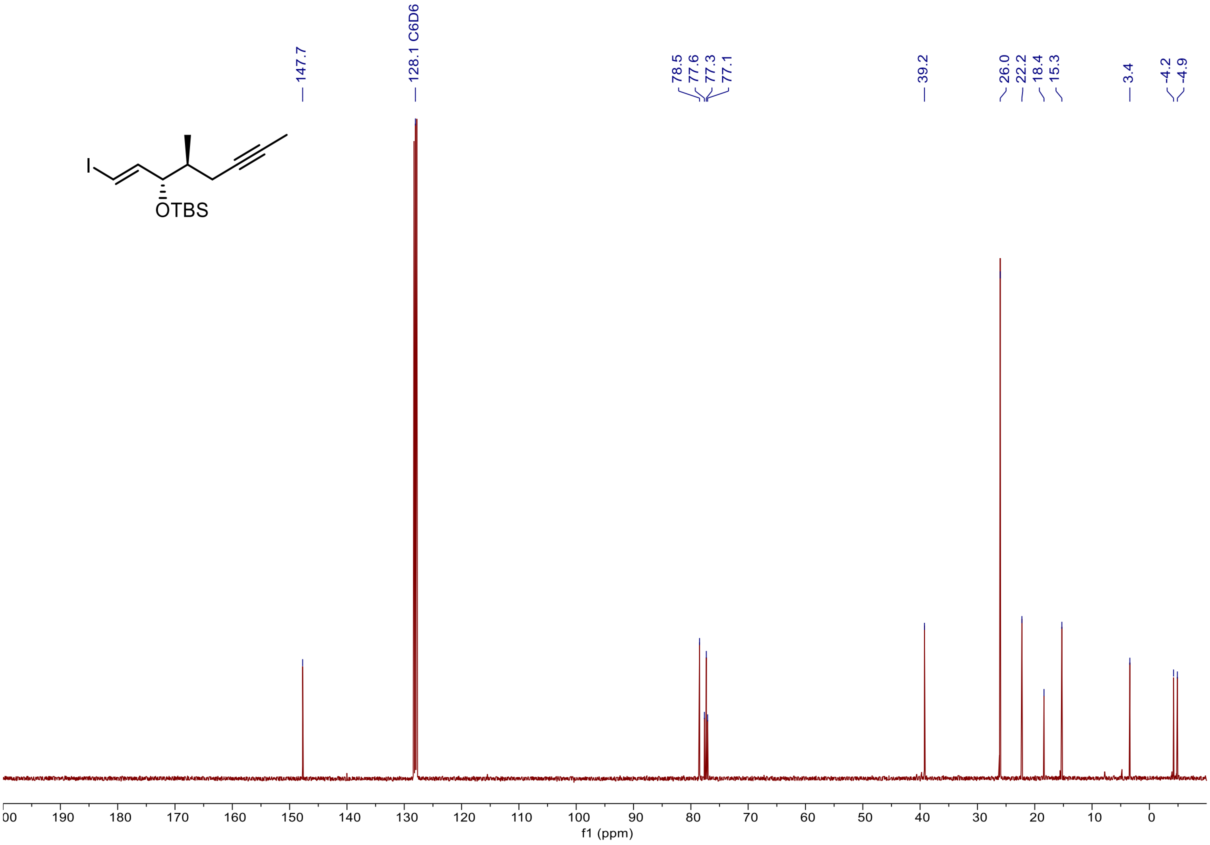

147.7
128.1 C6D6
78.5
77.6
77.3
77.1
39.2
26.0
22.2
18.4
15.3
3.4
-4.2
-4.9
OTBS
00 190 180 170 160 150 140 130 120 110 100 90 80 70 60 50 40 30 20 10 0
f1 (ppm)

Compound 49:

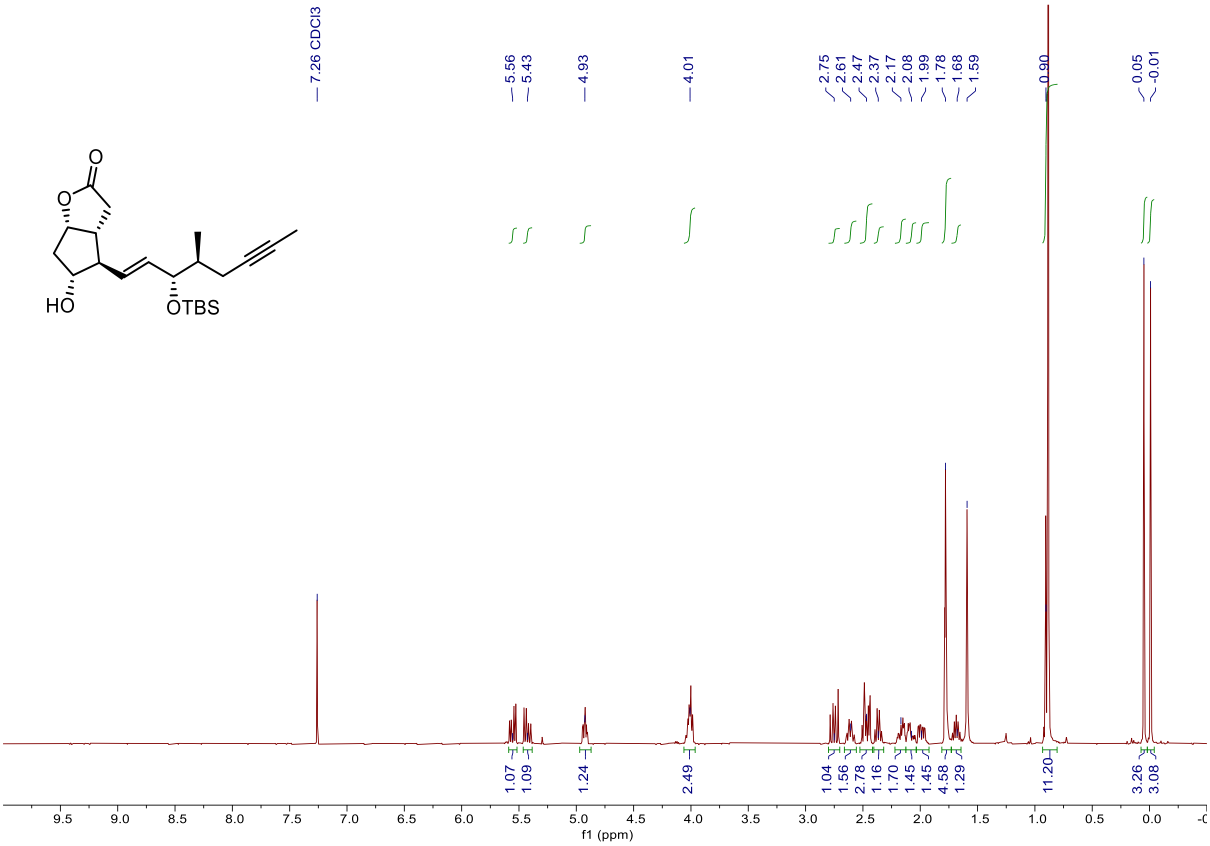

7.26 CDCl3
5.56
5.43
4.93
4.01
2.75
2.61
2.47
2.37
2.17
2.08
1.99
1.78
1.68
1.59
0.90
0.05
-0.01
HO
OTBS
O
1.07
1.09
1.24
2.49
1.04
1.56
2.78
1.16
1.70
1.45
1.45
4.58
1.29
11.20
3.26
3.08
f1 (ppm)

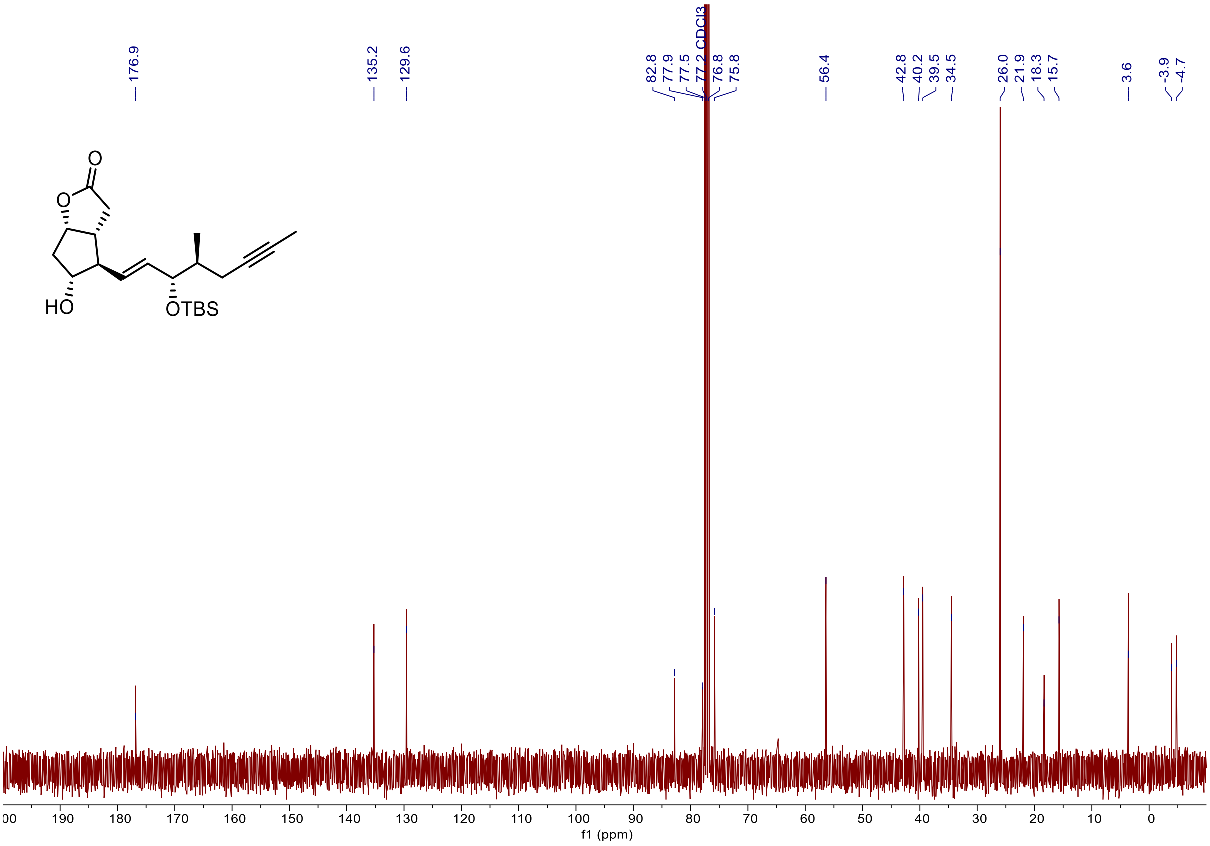

176.9
135.2
129.6
82.8
77.9
77.5
77.2 CDCl3
76.8
75.8
56.4
42.8
40.2
39.5
34.5
26.0
21.9
18.3
15.7
3.6
-3.9
-4.7
HO
OTBS
O
f1 (ppm)

Compound 15:

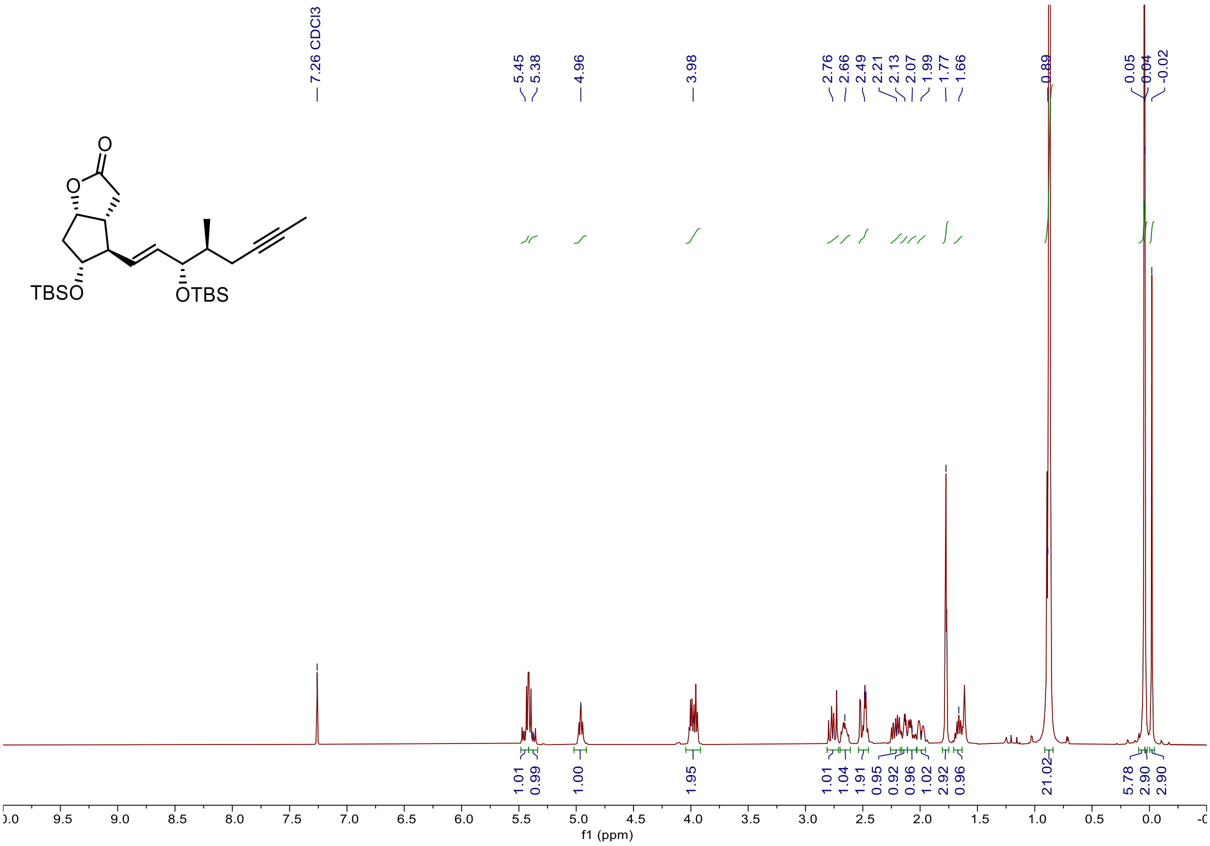
7.26 CDCl3
5.45
5.38
4.96
3.98
2.76
2.66
2.49
2.21
2.13
2.07
1.99
1.77
1.66
0.89
0.05
0.04
-0.02
O
TBSO
OTBS
1.01
0.99
1.00
1.95
1.01
1.04
1.91
0.95
0.92
0.96
1.02
2.92
0.96
21.02
5.78
2.90
2.90
f1 (ppm)

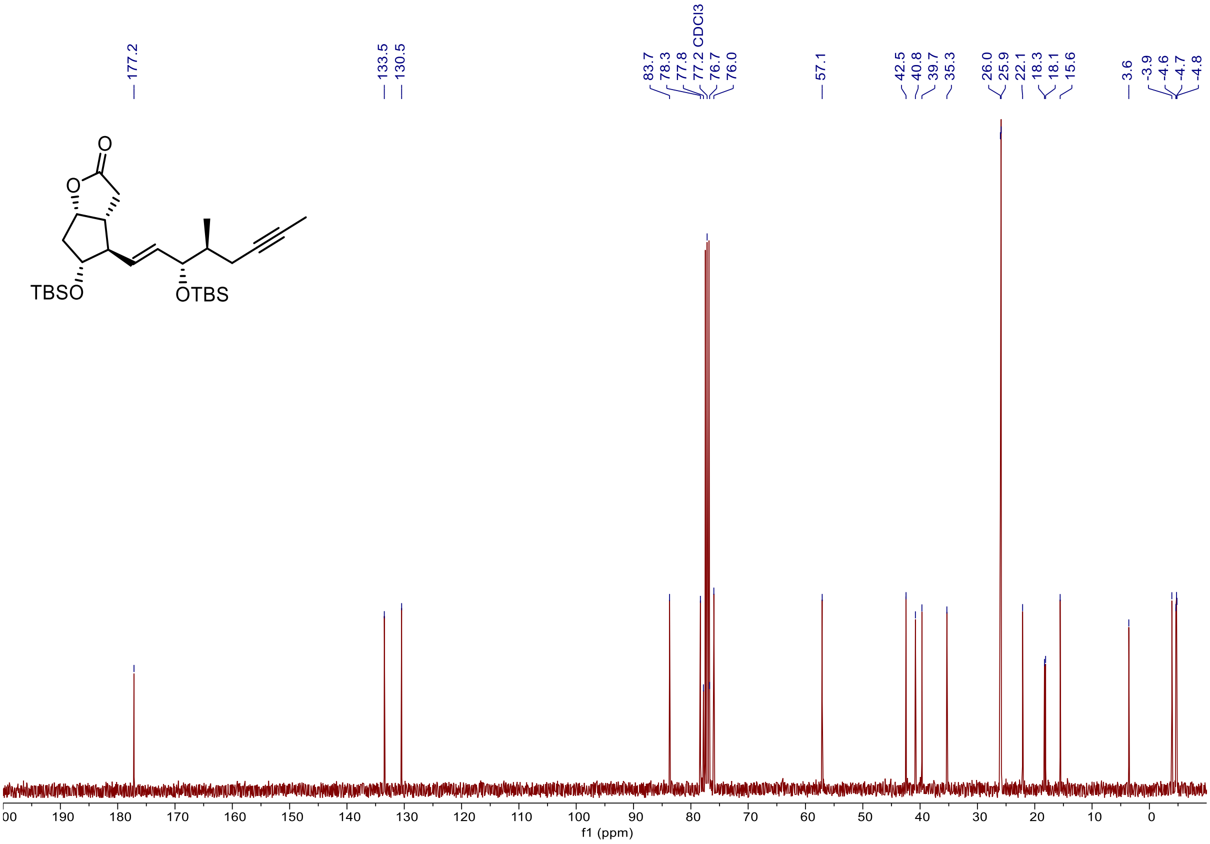
177.2
133.5
130.5
83.7
78.3
77.8
77.2 CDCl3
76.7
76.0
57.1
42.5
40.8
39.7
35.3
26.0
25.9
22.1
18.3
18.1
15.6
3.6
-3.9
-4.6
-4.7
-4.8
O
TBSO
OTBS
f1 (ppm)

Compound 5:

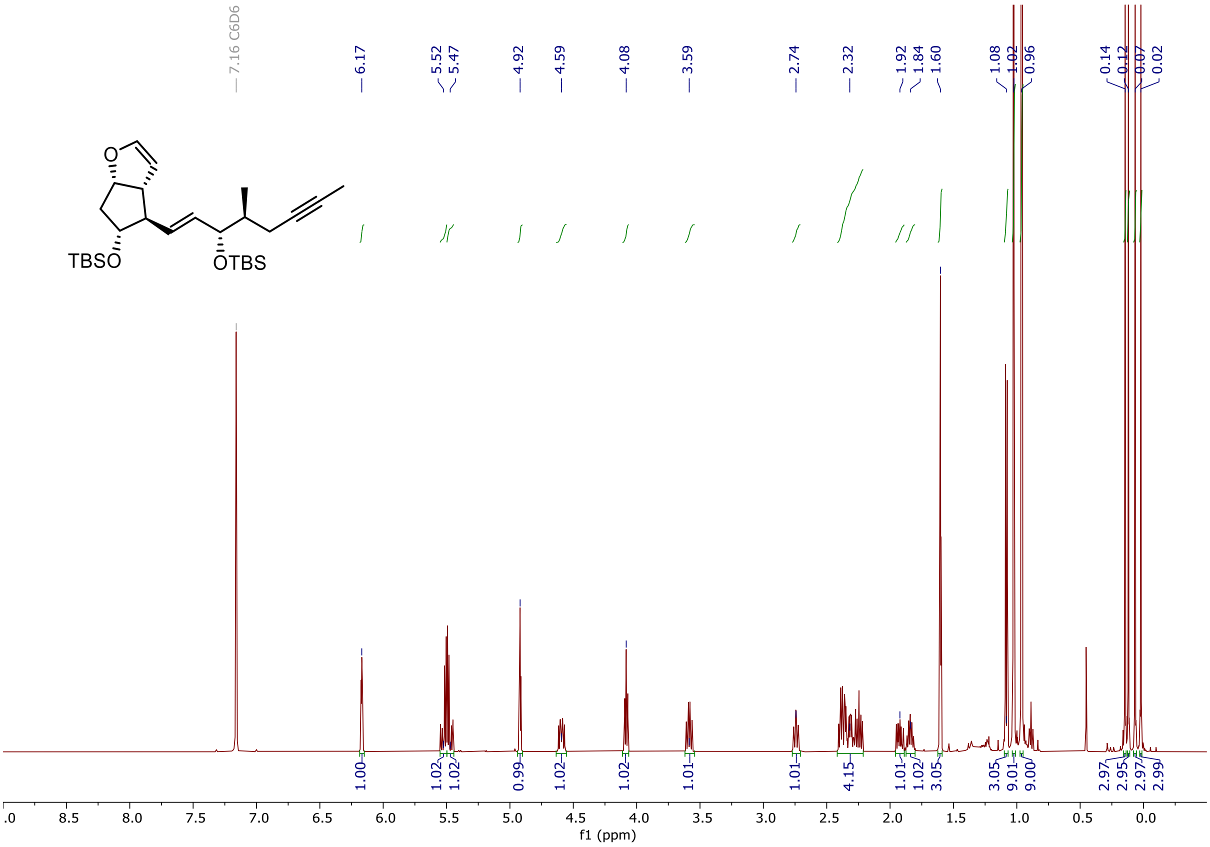
7.16 C6D6
6.17
5.52
5.47
4.92
4.59
4.08
3.59
2.74
2.32
1.92
1.84
1.60
1.08
1.02
0.96
0.14
0.12
0.07
0.02
TBSO
OTBS
O
1.00
1.02
1.02
0.99
1.02
1.02
1.01
1.01
4.15
1.01
1.02
3.05
3.05
9.01
9.00
2.97
2.95
2.97
2.99
f1 (ppm)

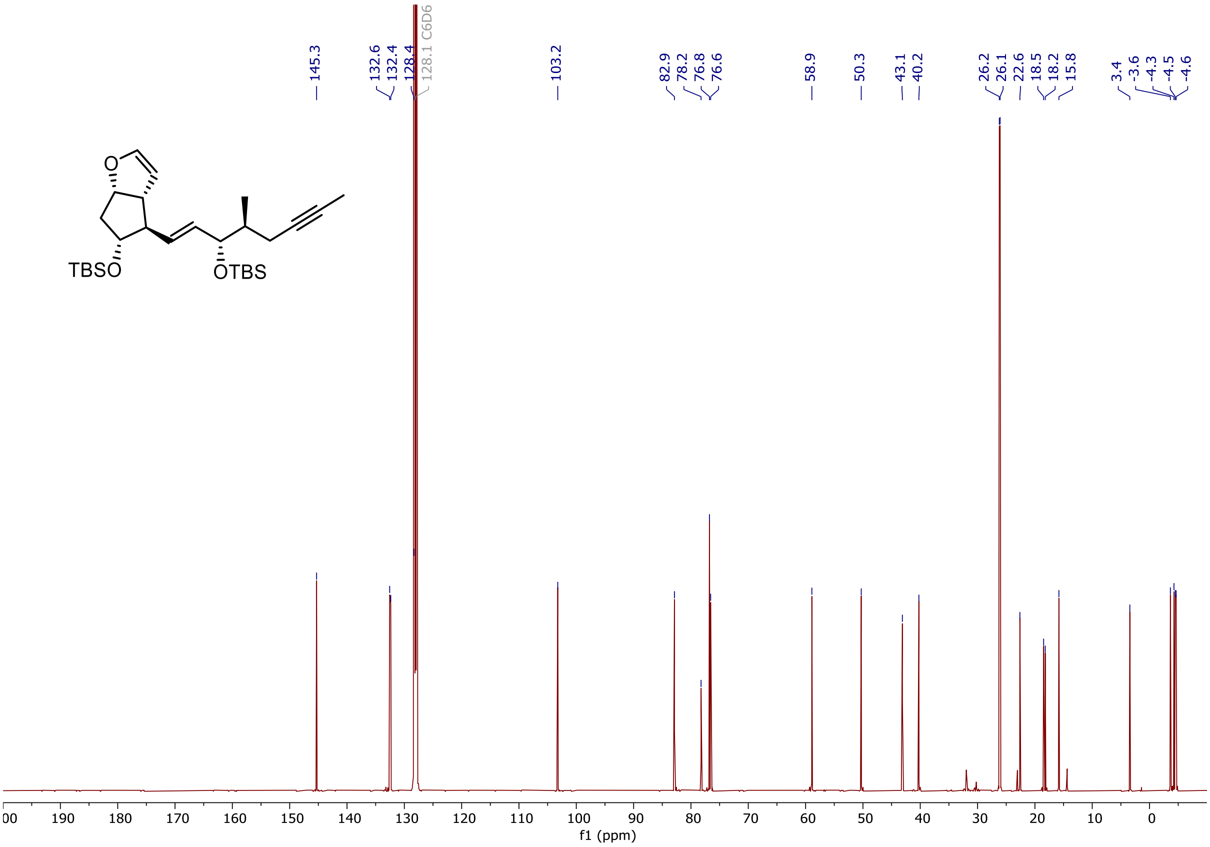
145.3
132.6
132.4
128.4
128.1 C6D6
103.2
82.9
78.2
76.8
76.6
58.9
50.3
43.1
40.2
26.2
26.1
22.6
18.5
18.2
15.8
3.4
-3.6
-4.3
-4.5
-4.6
TBSO
OTBS
O
f1 (ppm)

Compound 3:

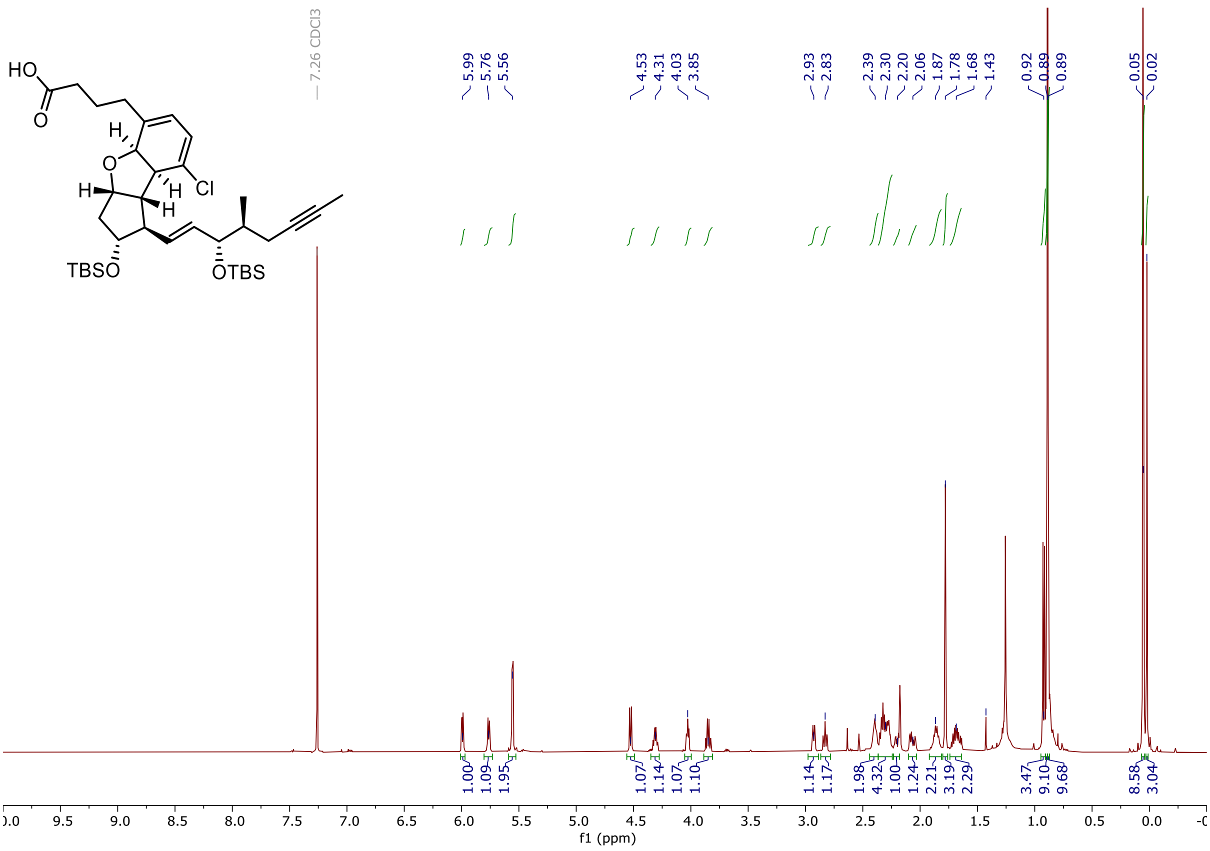
7.26 CDCl3
5.99 5.76 5.56
4.53 4.31 4.03 3.85
2.93 2.83
2.39 2.30 2.20 2.06 1.87 1.78 1.68 1.43
0.92 0.89 0.89
0.05 0.02
HO
O
H
Cl
TBSO
OTBS
1.00 1.09 1.95 1.07 1.14 1.07 1.10 1.14 1.17 1.98 4.32 1.00 1.24 2.21 3.19 2.29 3.47 9.10 9.68 8.58 3.04
f1 (ppm)

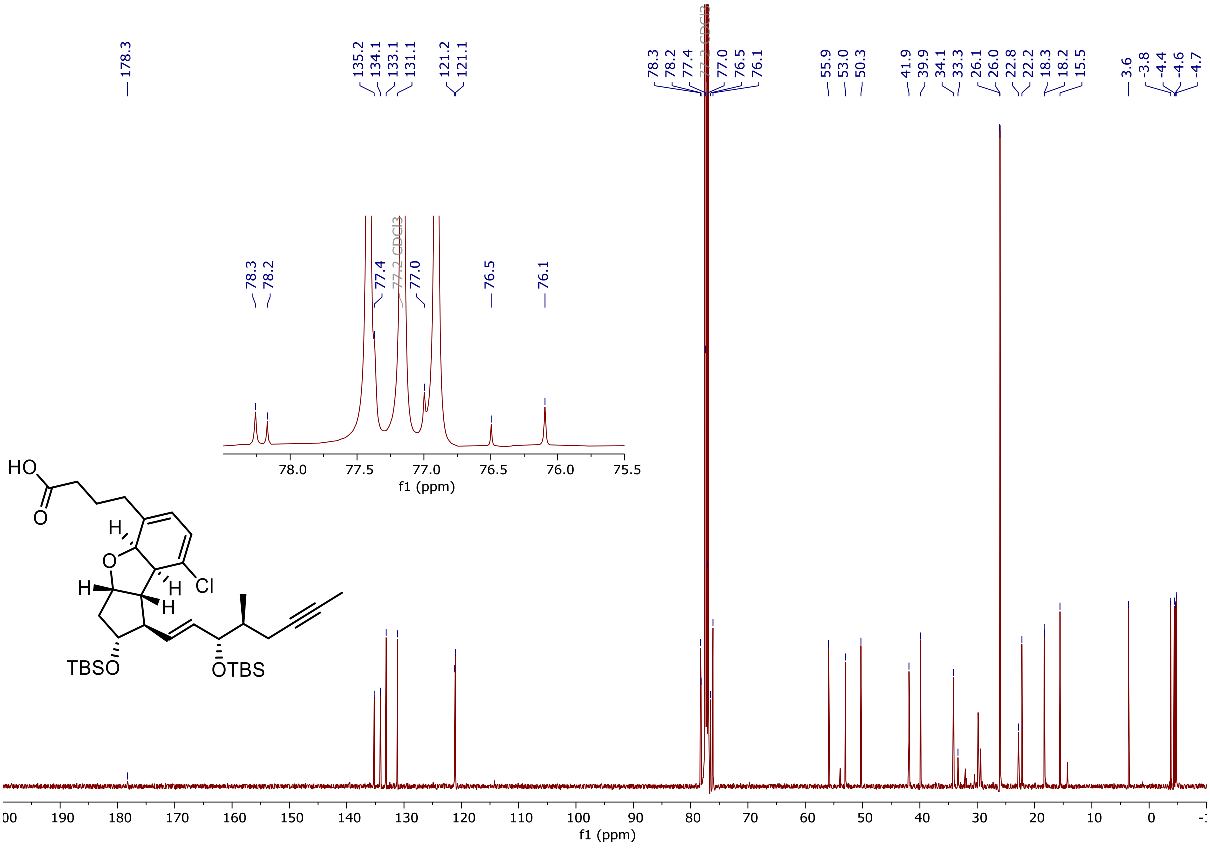
178.3
135.2 134.1 133.1 131.1
121.2 121.1
78.3 78.2 77.4 77.0 76.5 76.1
55.9 53.0 50.3
41.9 39.9 34.1 33.3 26.1 26.0 22.8 22.2 18.3 18.2 15.5
3.6 -3.8 -4.4 -4.6 -4.7
77.2 CDCl3
HO
O
H
Cl
TBSO
OTBS
f1 (ppm)

2D NMR (HSQC and HMBC): Peak-picking of 2D data for upper sidechain correlations.

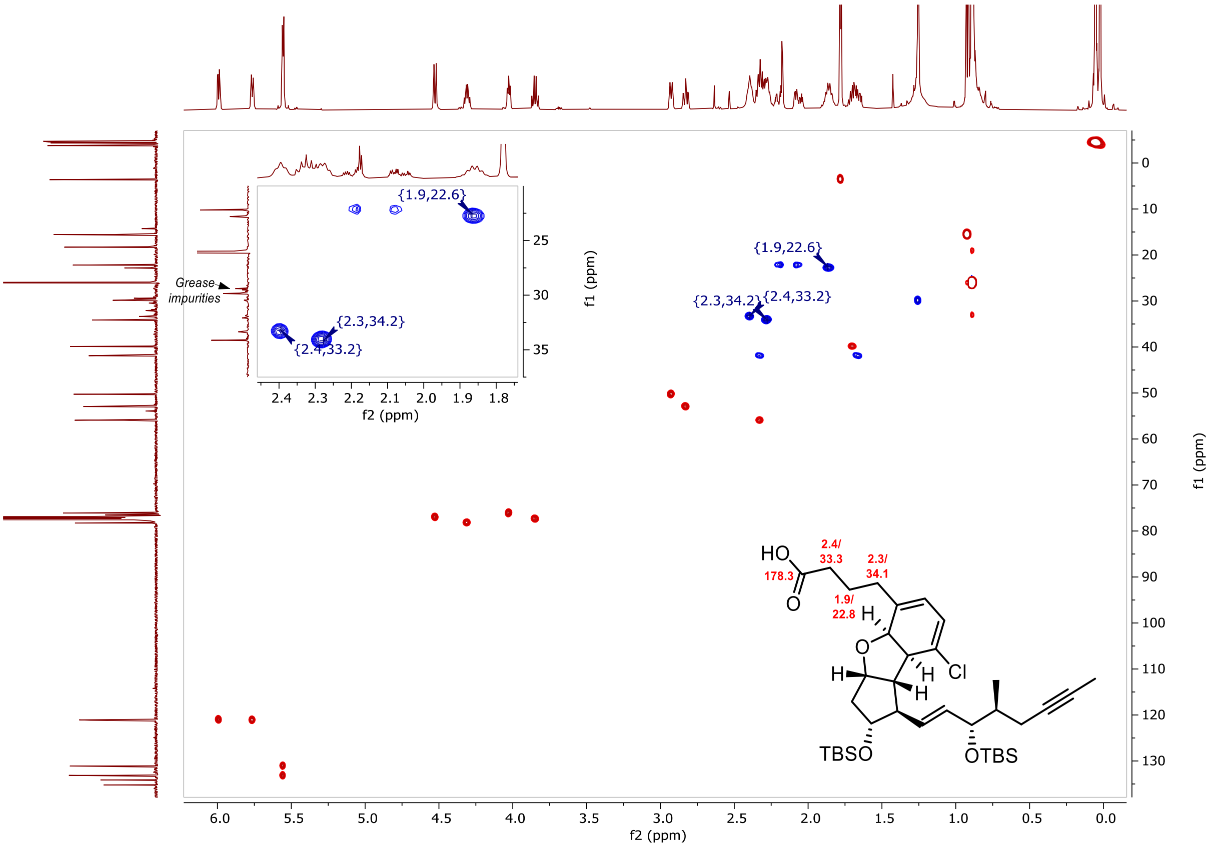

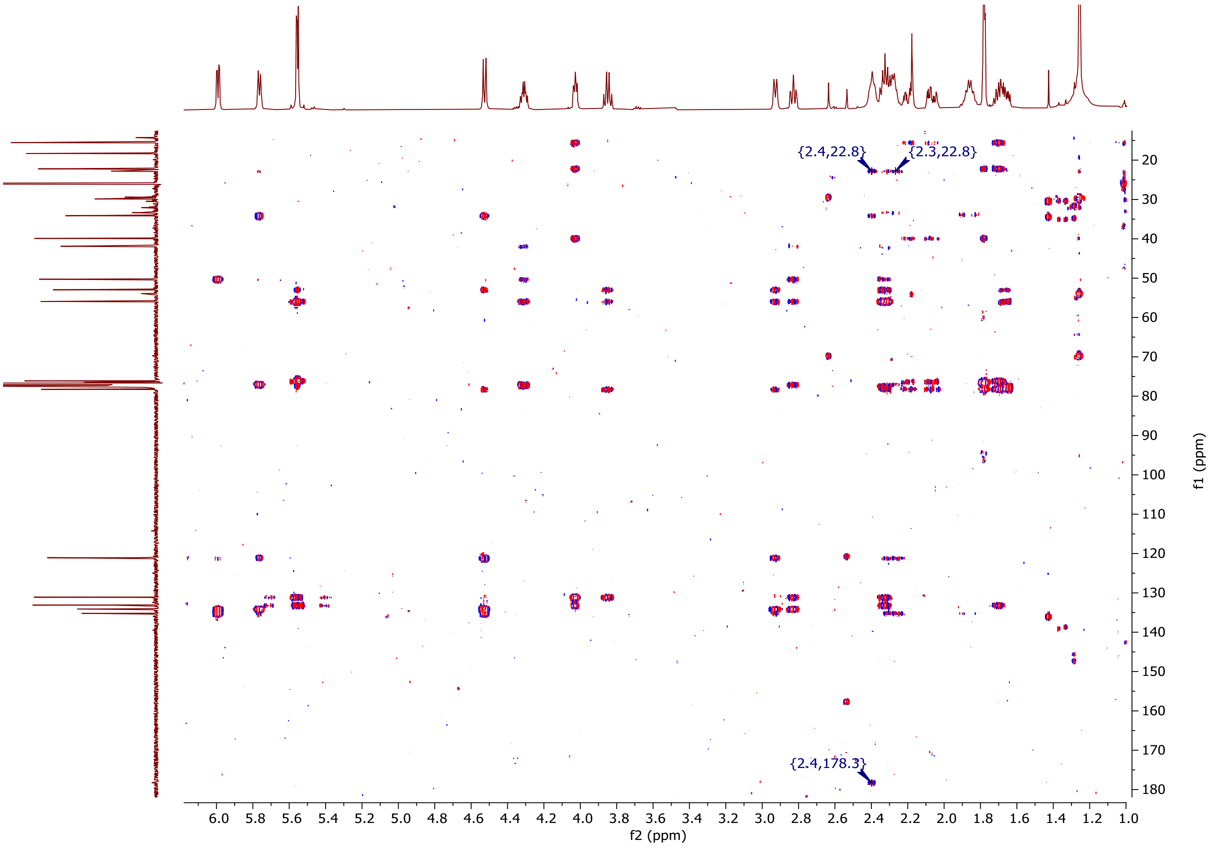

Compound 3a:

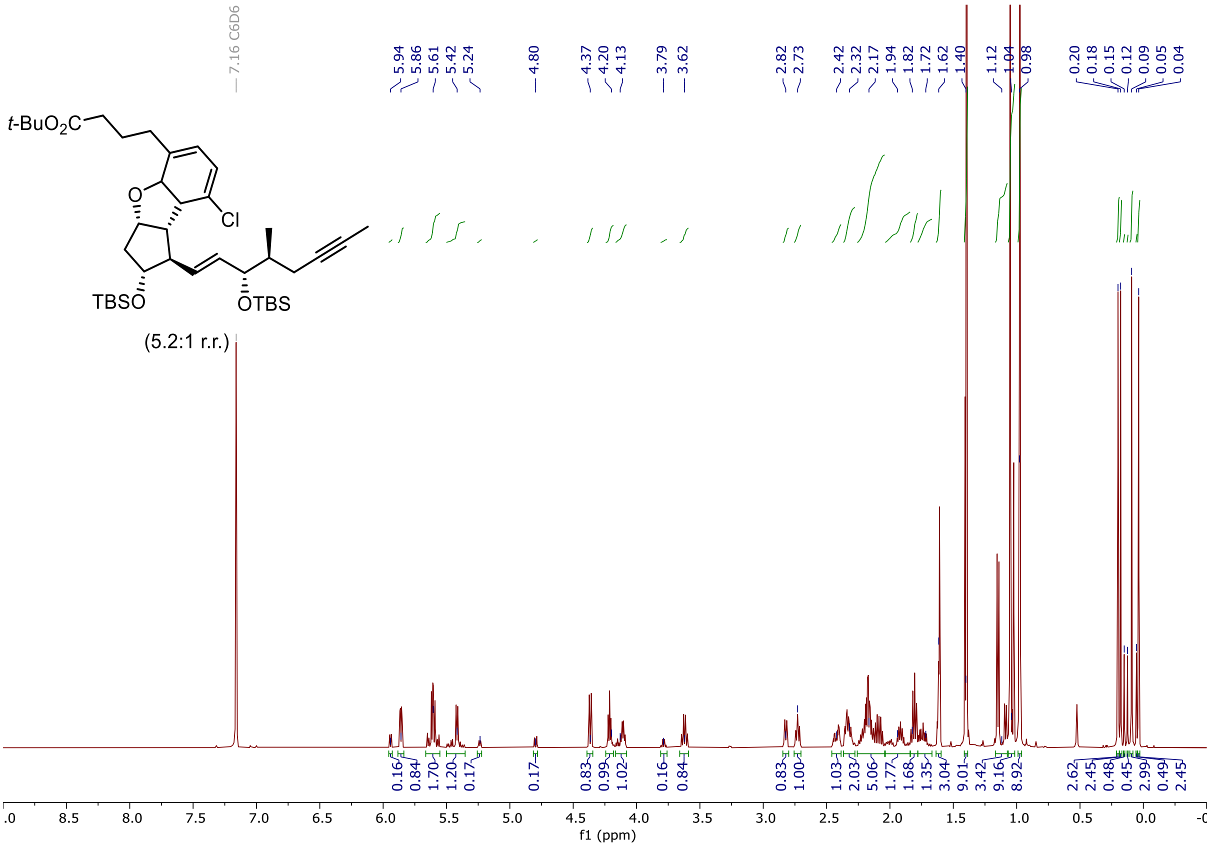

7.16 C6D6
5.94
5.86
5.61
5.42
5.24
4.80
4.37
4.20
4.13
3.79
3.62
2.82
2.73
2.42
2.32
2.17
1.94
1.82
1.72
1.62
1.40
1.12
1.04
0.98
0.20
0.18
0.15
0.12
0.09
0.05
0.04
t-BuO2C
O
Cl
TBSO
OTBS
(5.2:1 r.r.)
0.16
0.84
1.70
1.20
0.17
0.17
0.83
0.99
1.02
0.16
0.84
0.83
1.00
1.03
2.03
5.06
1.77
1.68
1.35
3.04
9.01
3.42
9.16
8.92
2.62
2.45
0.48
0.45
2.99
0.49
2.45
f1 (ppm)

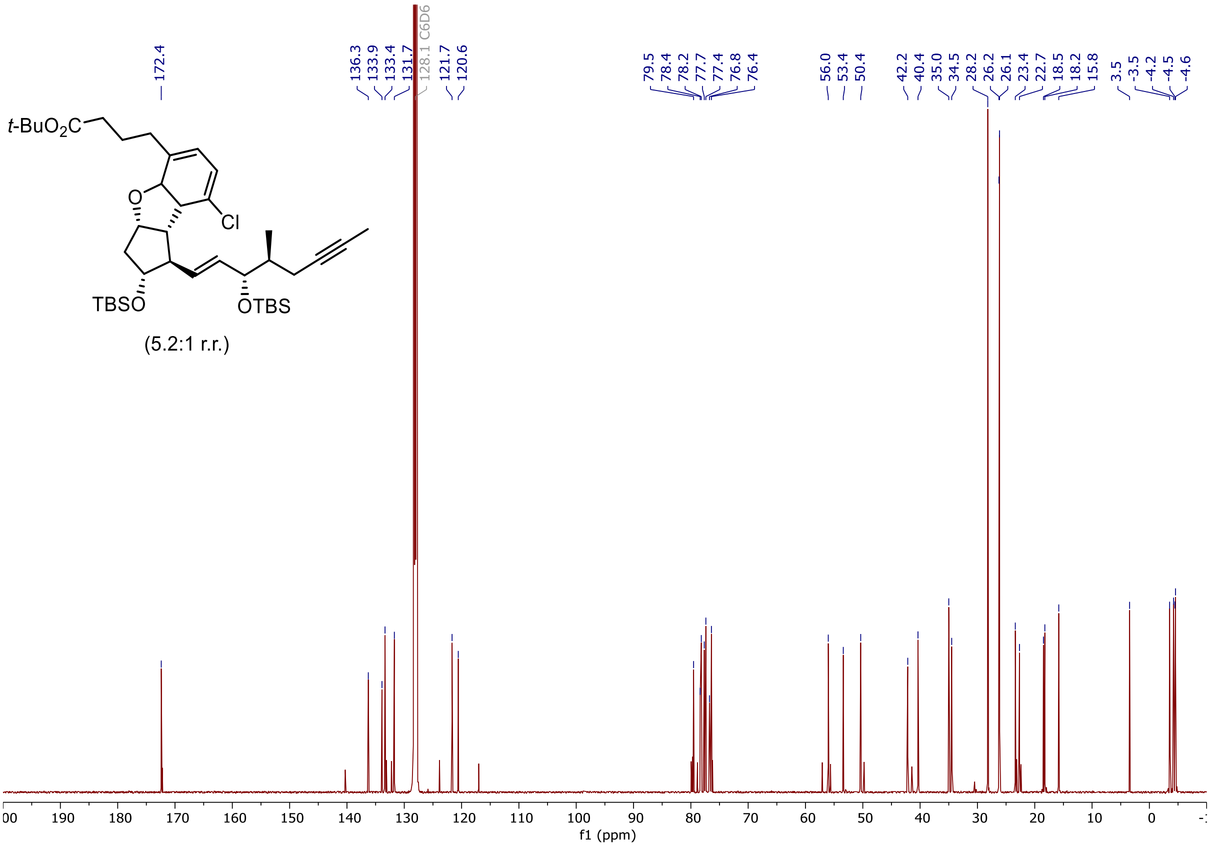

172.4
136.3
133.9
133.4
131.7
128.1 C6D6
121.7
120.6
79.5
78.4
78.2
77.7
77.4
76.8
76.4
56.0
53.4
50.4
42.2
40.4
35.0
34.5
28.2
26.2
26.1
23.4
22.7
18.5
18.2
15.8
3.5
-3.5
-4.2
-4.5
-4.6
t-BuO2C
O
Cl
TBSO
OTBS
(5.2:1 r.r.)
f1 (ppm)

Compound 16:

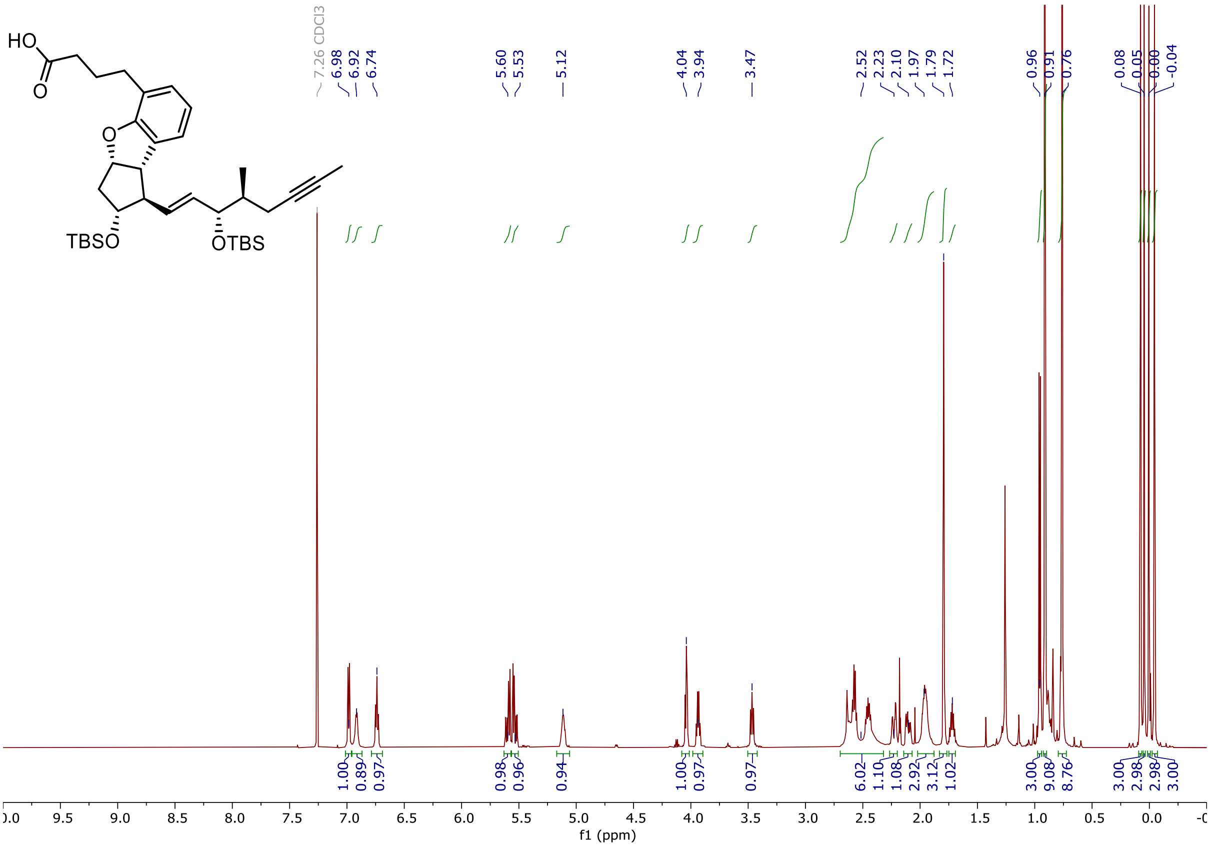
HO
O
O
TBSO
OTBS
7.26 CDCl3
6.98
6.92
6.74
5.60
5.53
5.12
4.04
3.94
3.47
2.52
2.23
2.10
1.97
1.79
1.72
0.96
0.91
0.76
0.08
0.05
0.00
-0.04
1.00
0.89
0.97
0.98
0.96
0.94
1.00
0.97
0.97
6.02
1.10
1.08
2.92
3.12
1.02
3.00
9.08
8.76
3.00
2.98
2.98
3.00
f1 (ppm)

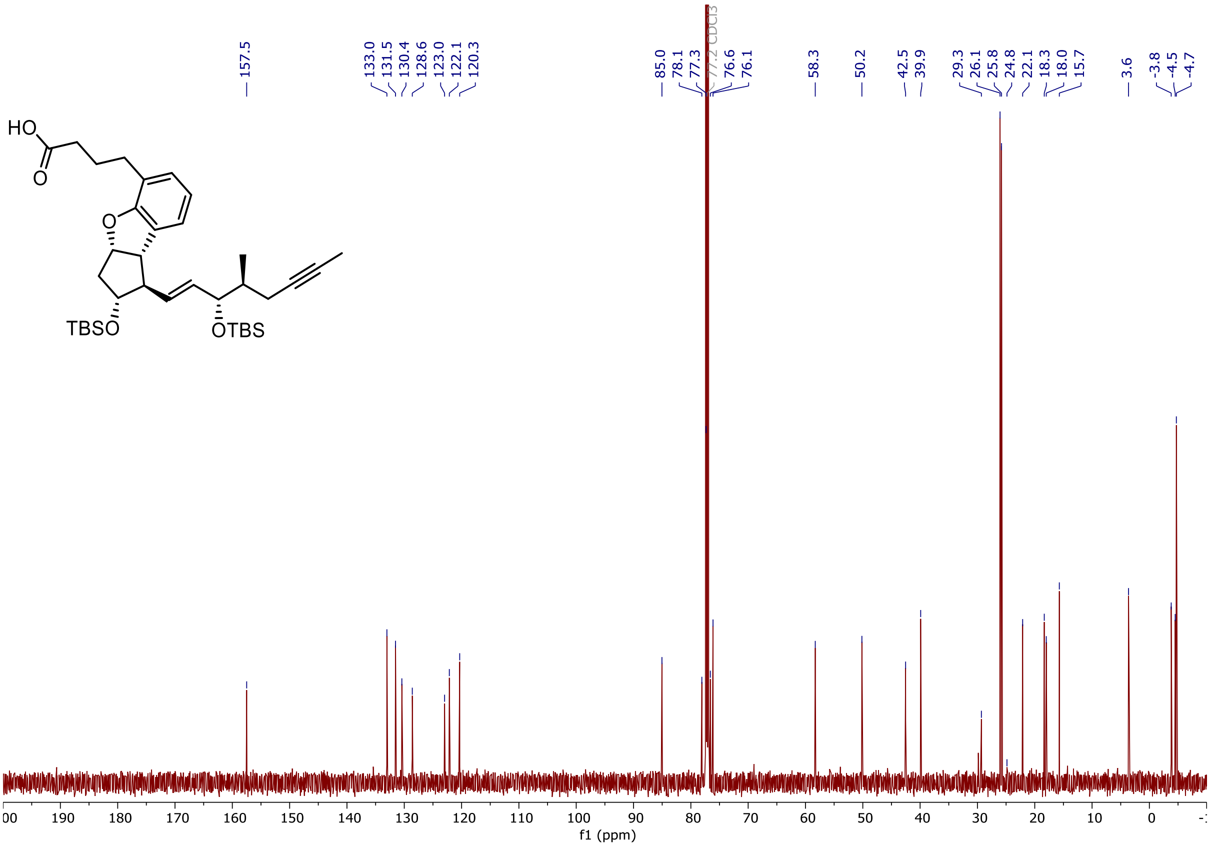
HO
O
O
TBSO
OTBS
157.5
133.0
131.5
130.4
128.6
123.0
122.1
120.3
85.0
78.1
77.3
77.2 CDCl3
76.6
76.1
58.3
50.2
42.5
39.9
29.3
26.1
25.8
24.8
22.1
18.3
18.0
15.7
3.6
-3.8
-4.5
-4.7
f1 (ppm)

2D NMR (HSQC): Carboxylic acid peak and the peak *alpha* to it not observed due to proposed dimer formation.[13]

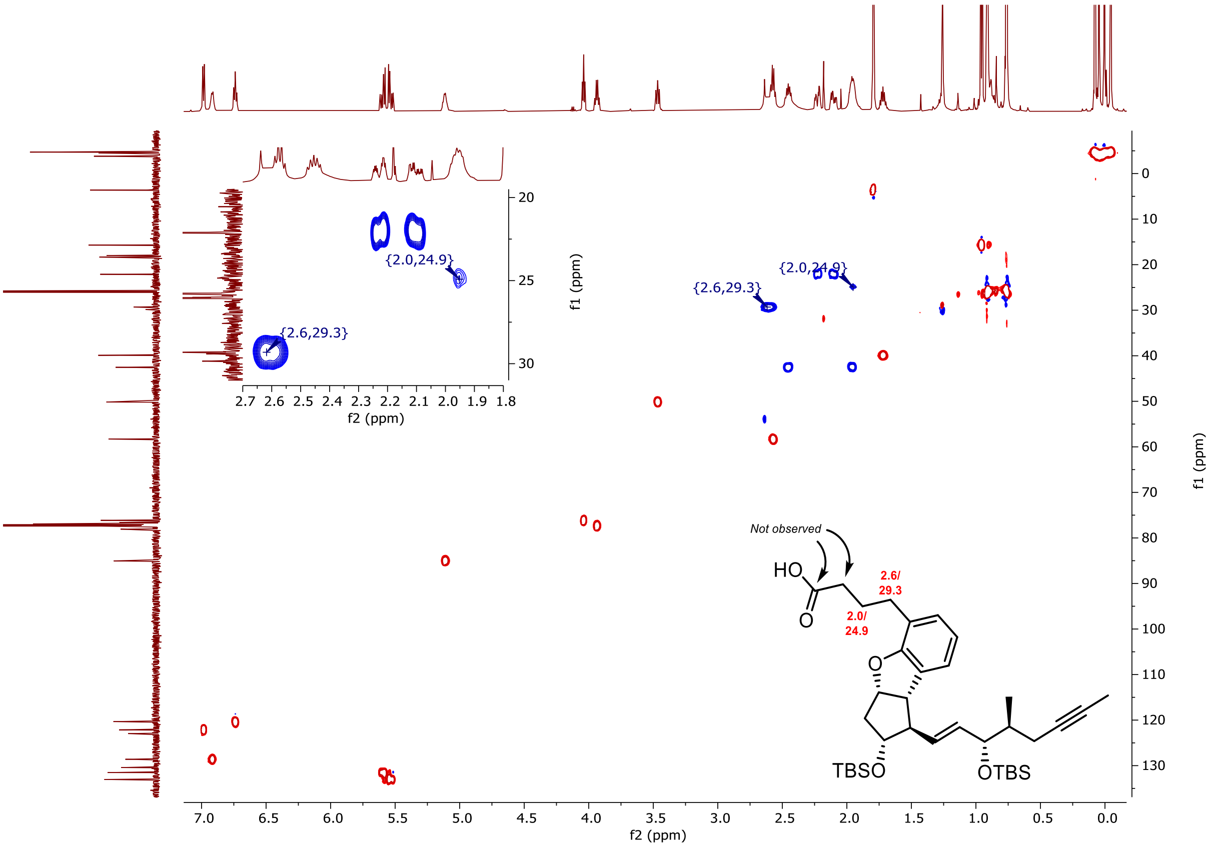

Compound 1 (Beraprost):

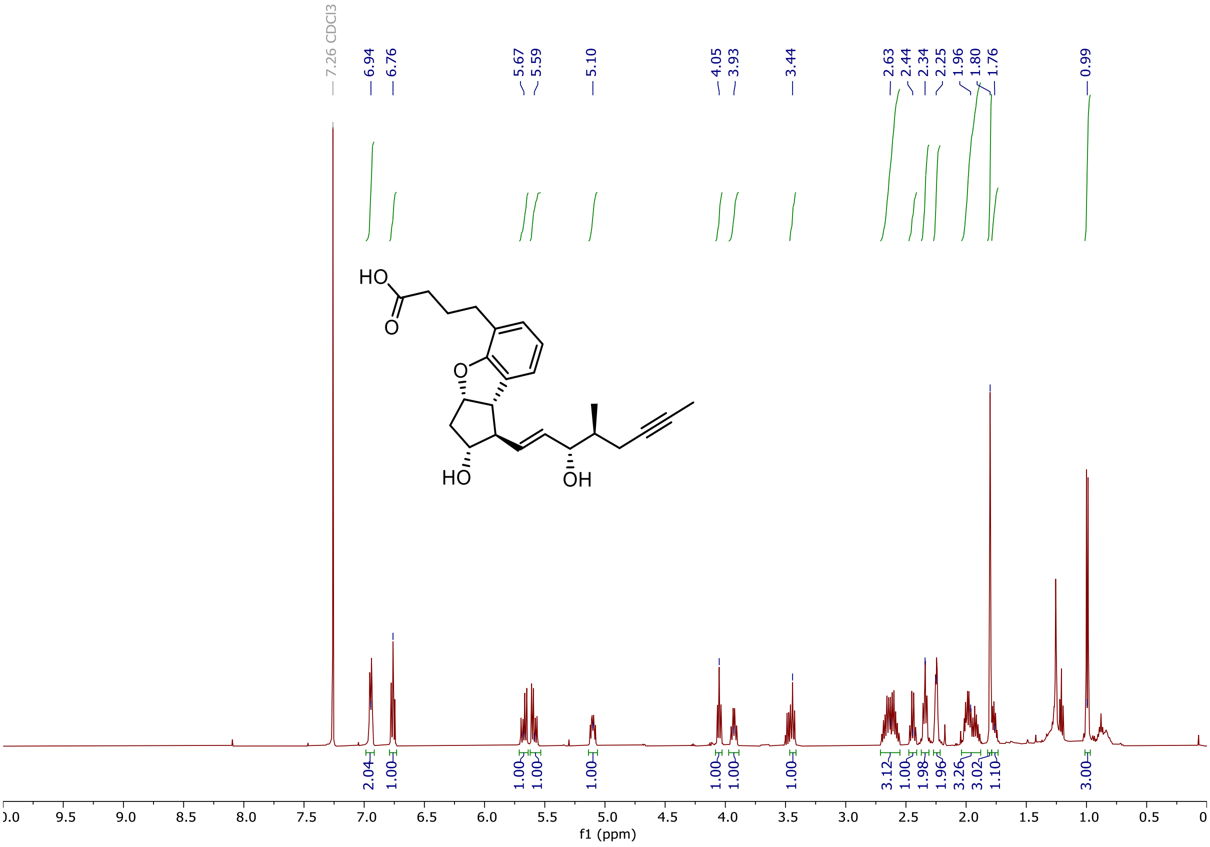

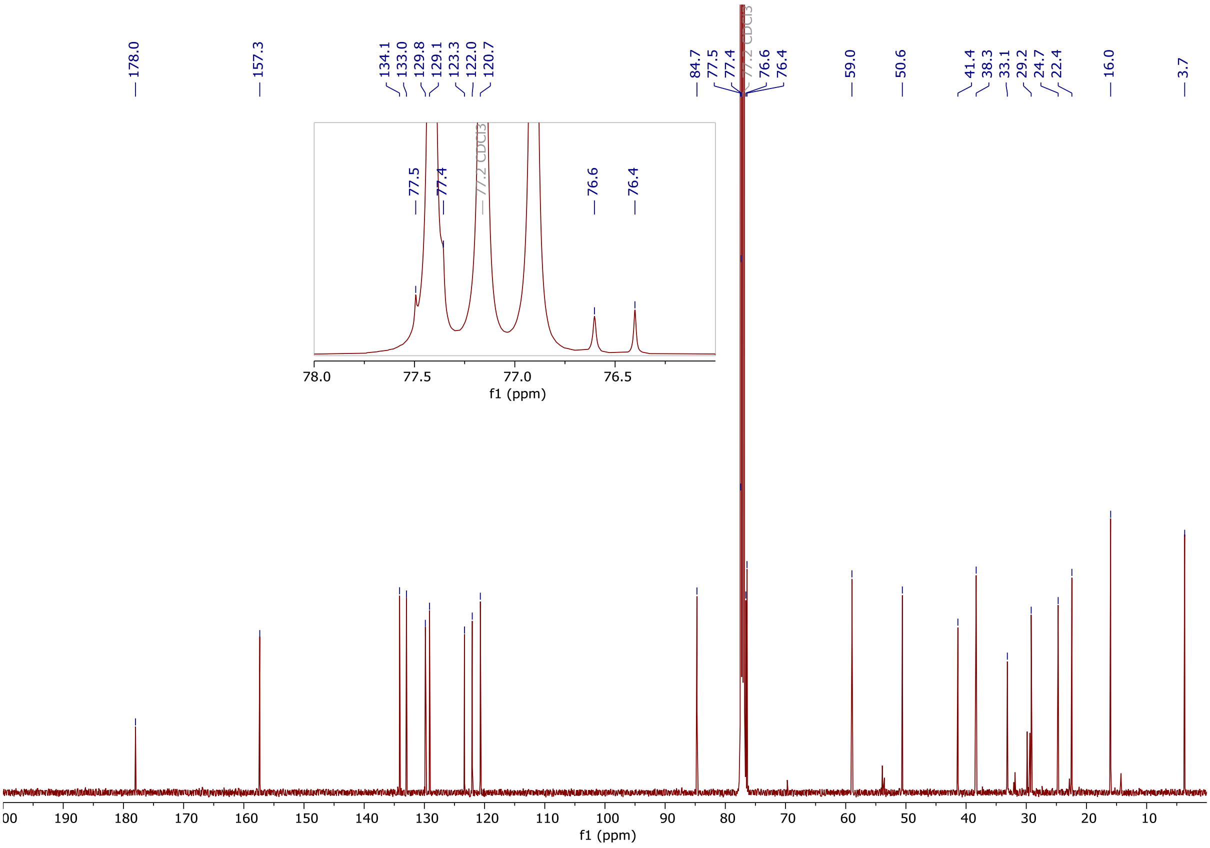

2D NMR (HSQC and HMBC):

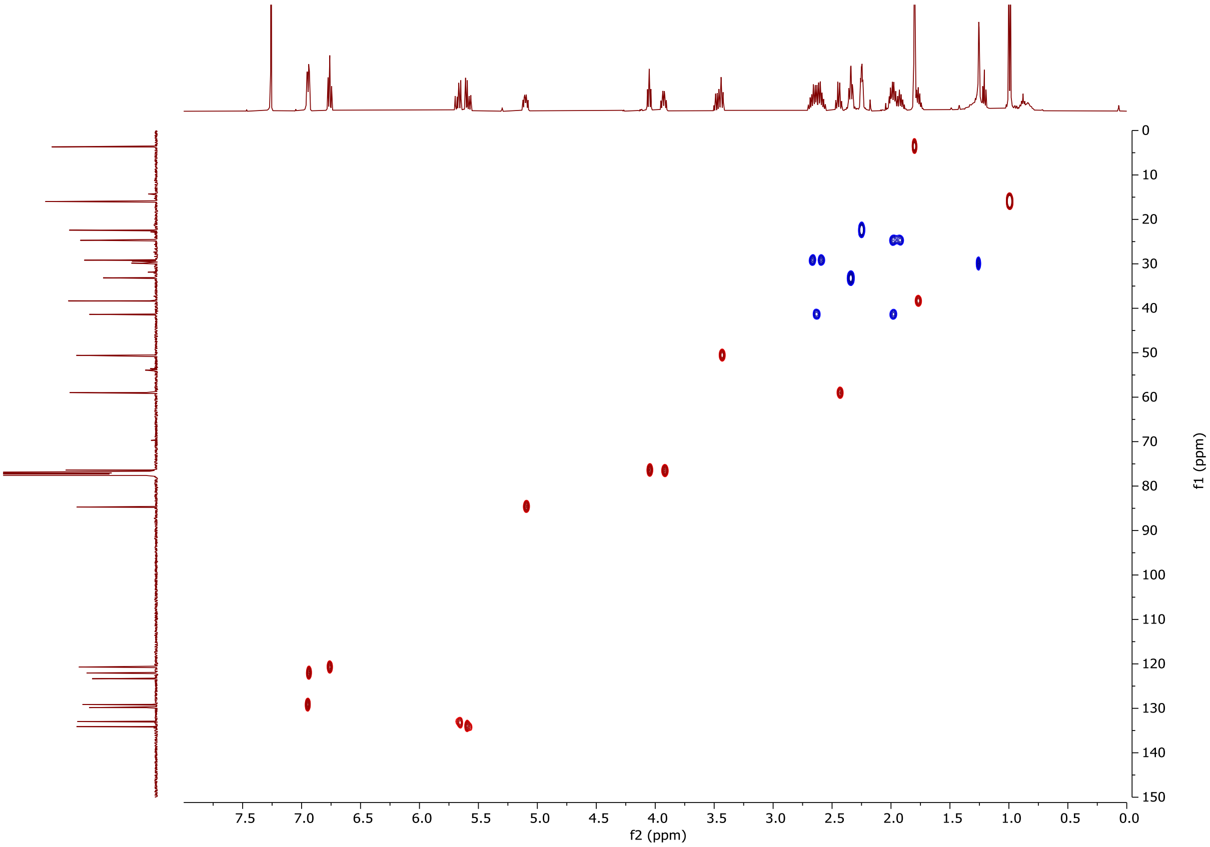

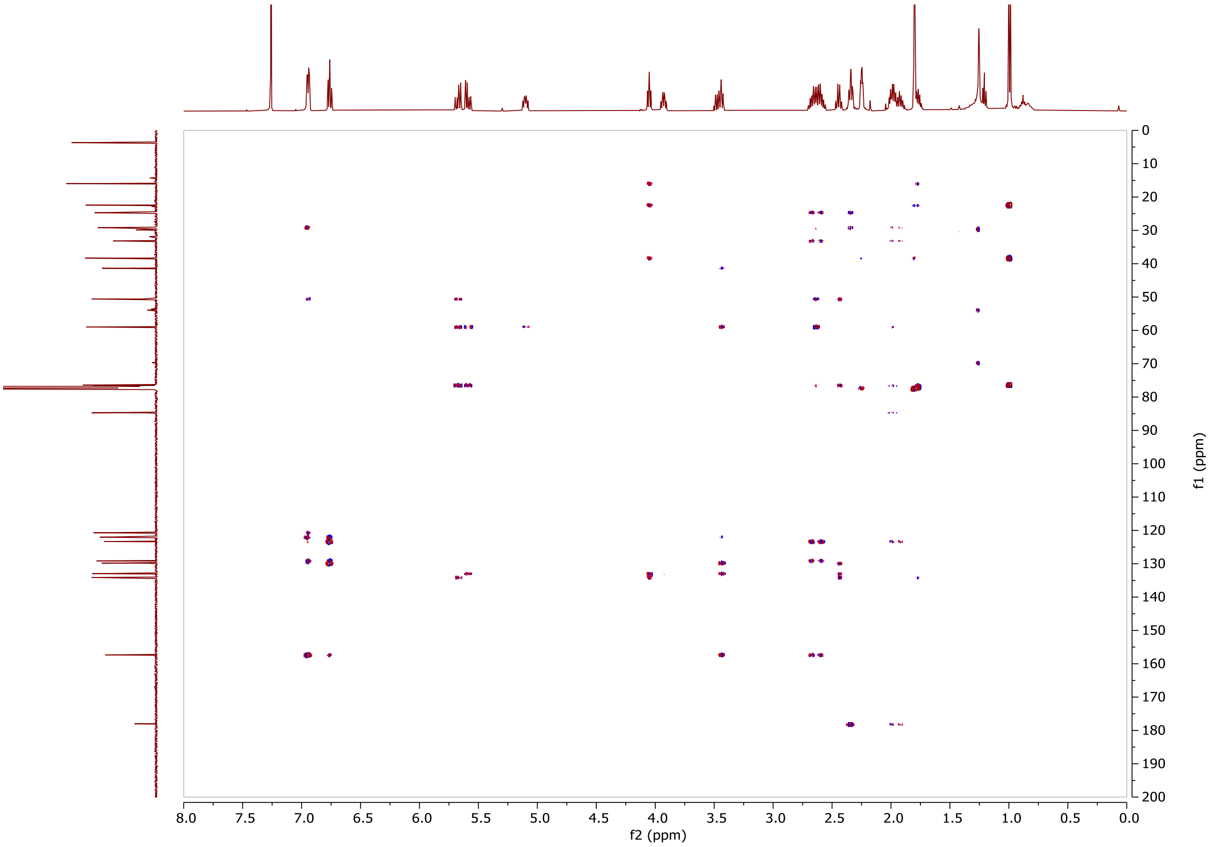

8.2. Dehalogenation–Aromatization Model Studies

Compound 50:

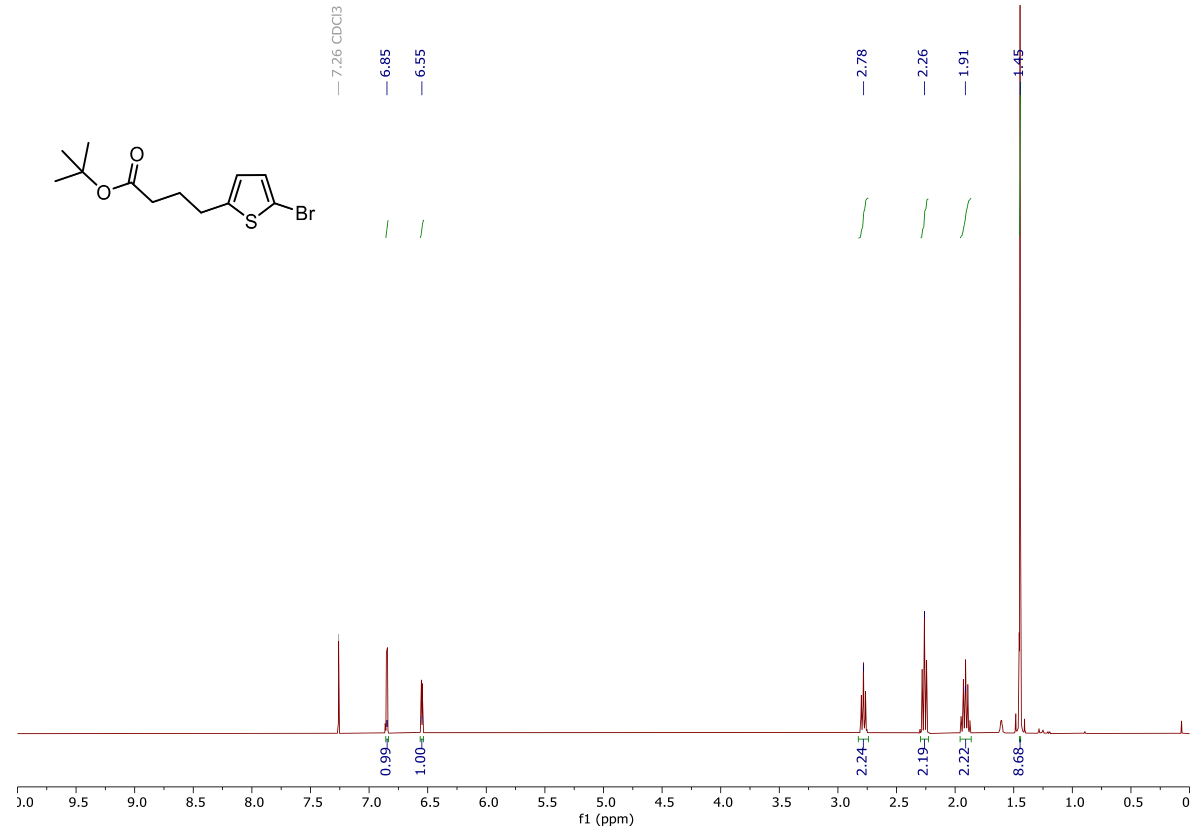

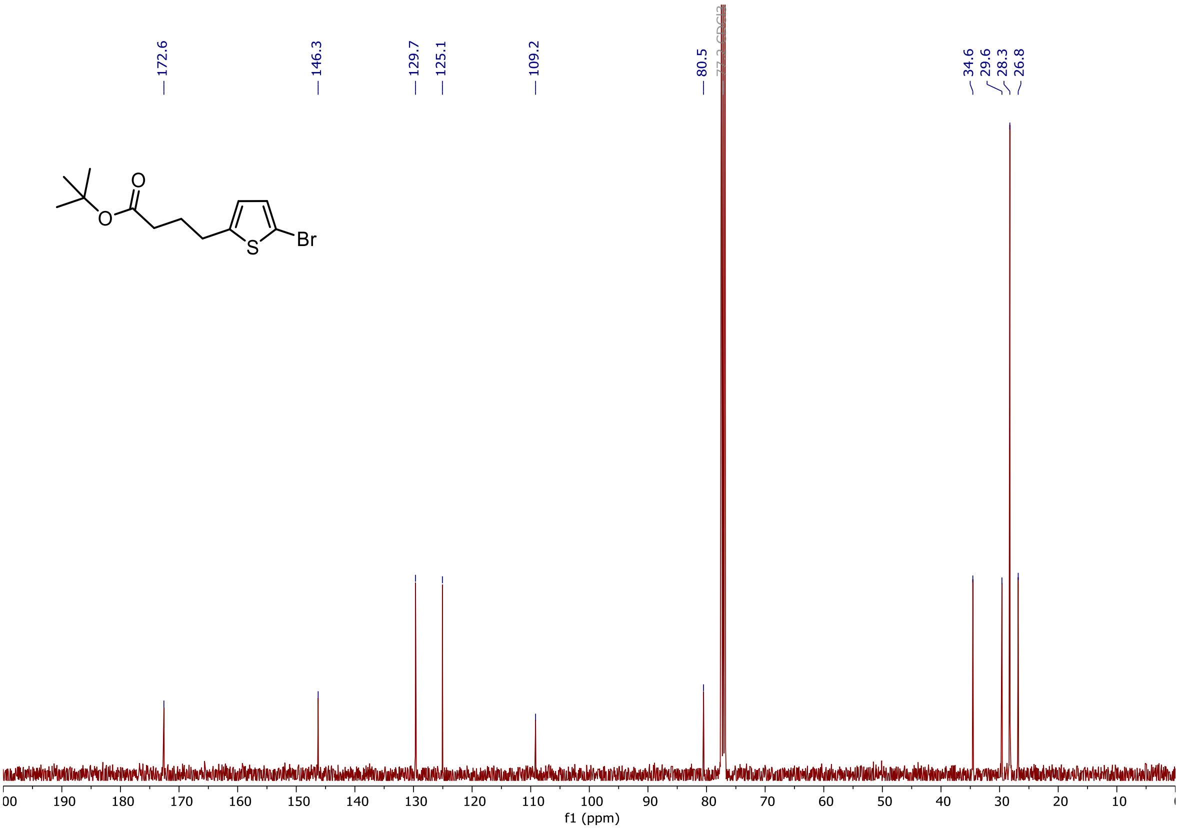

Compound 19a:

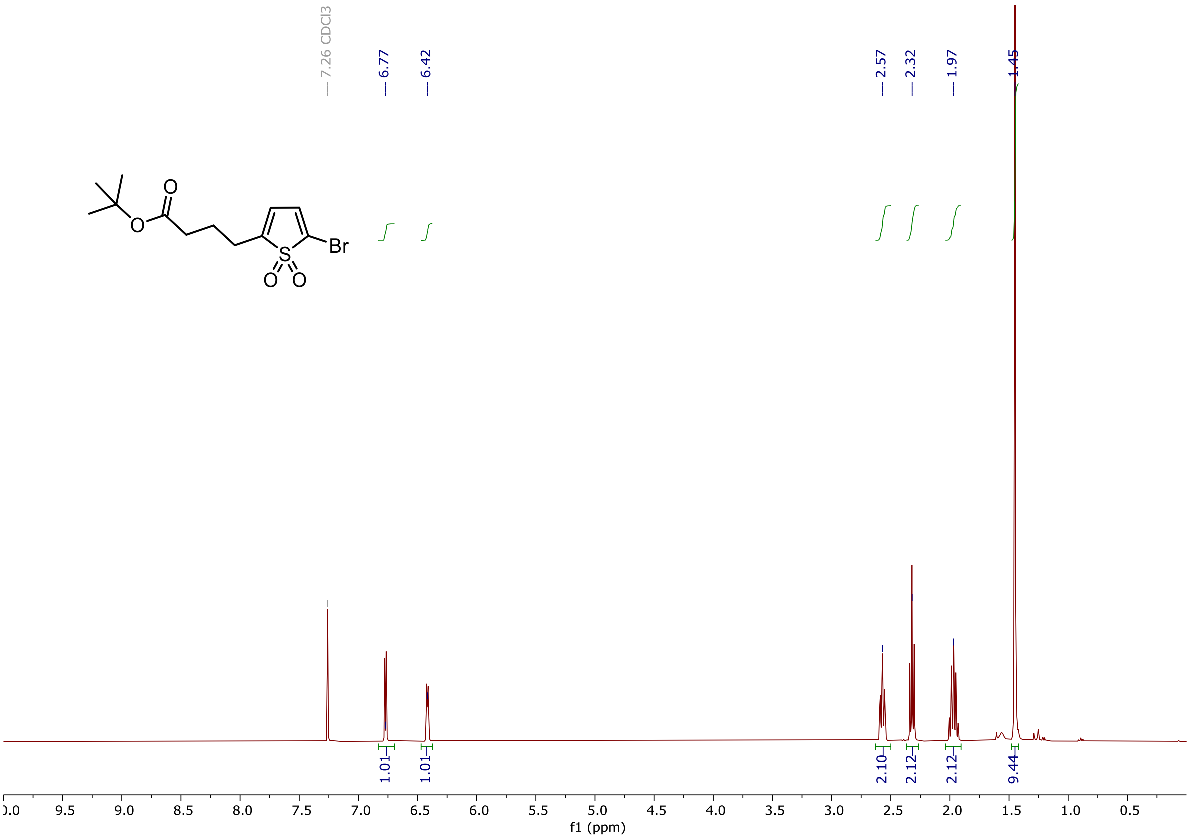

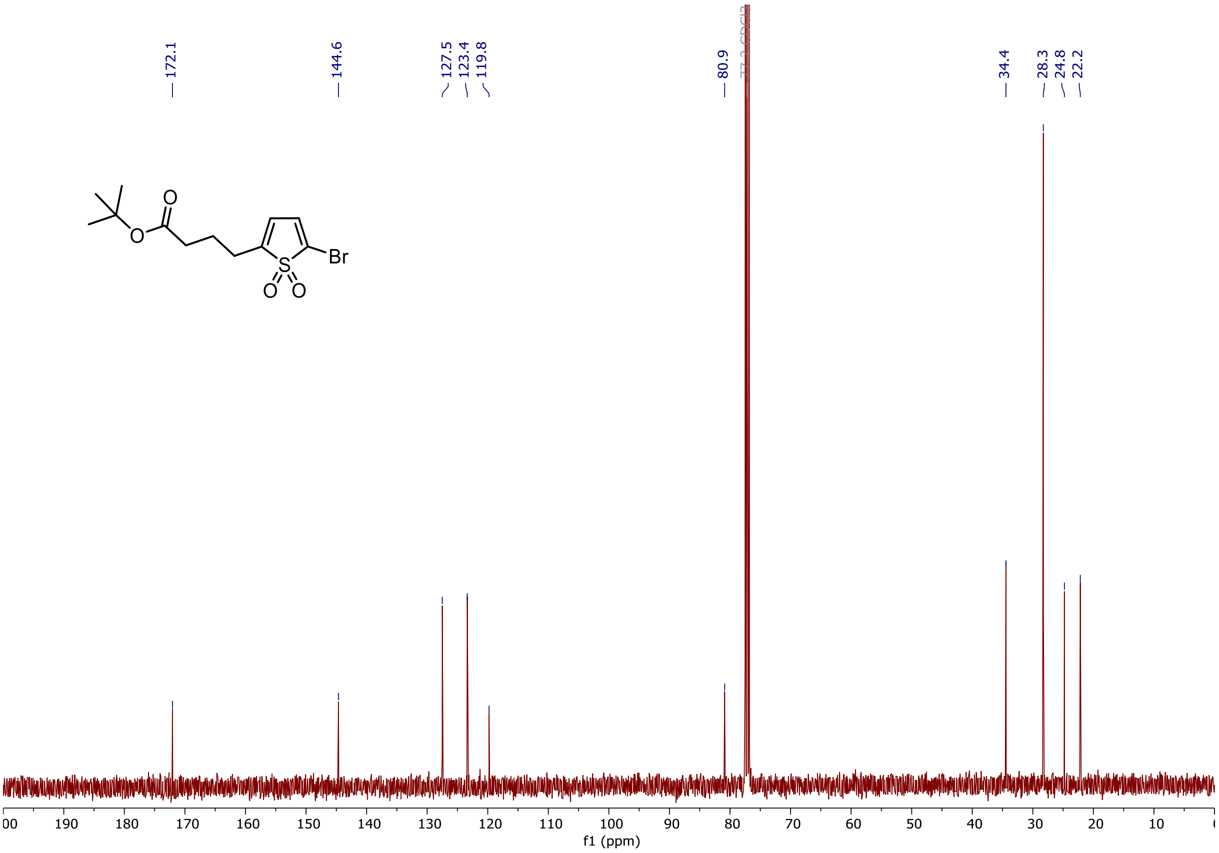

Compound 19b:

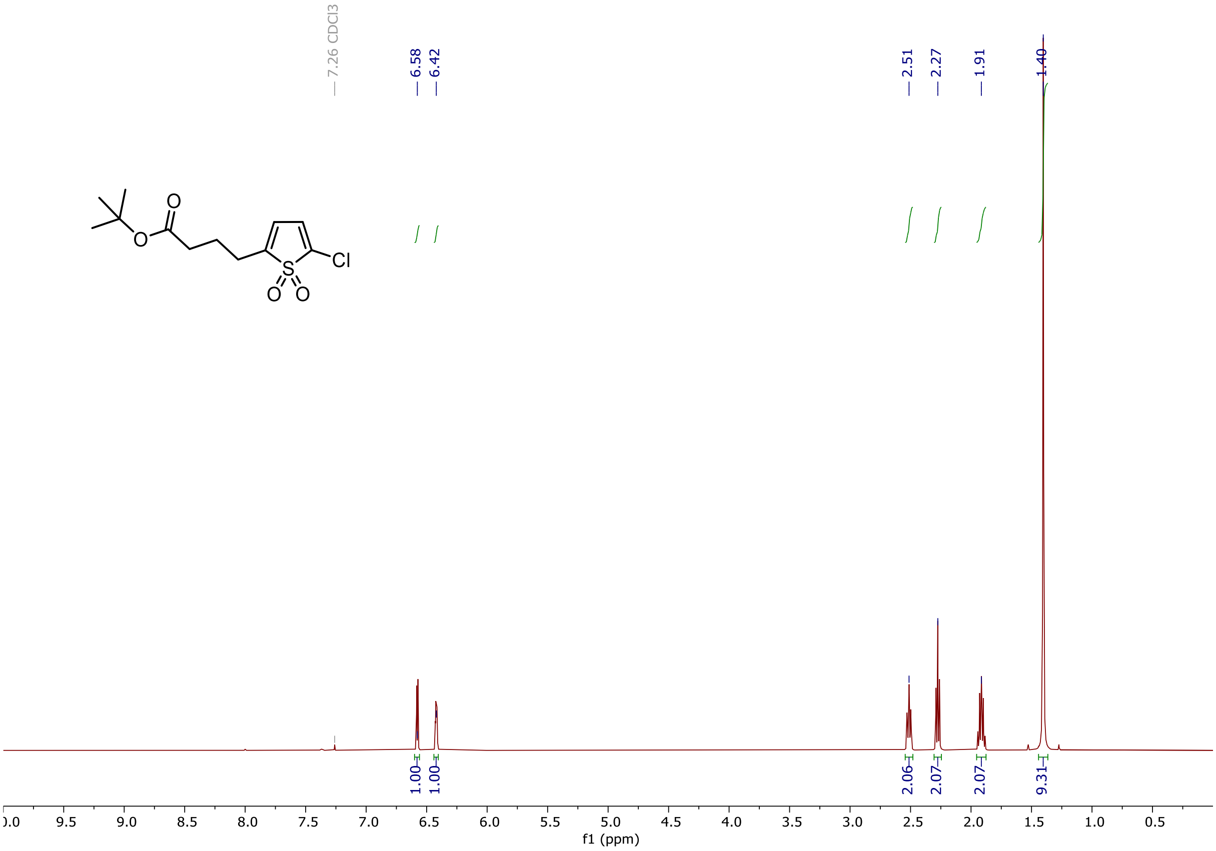

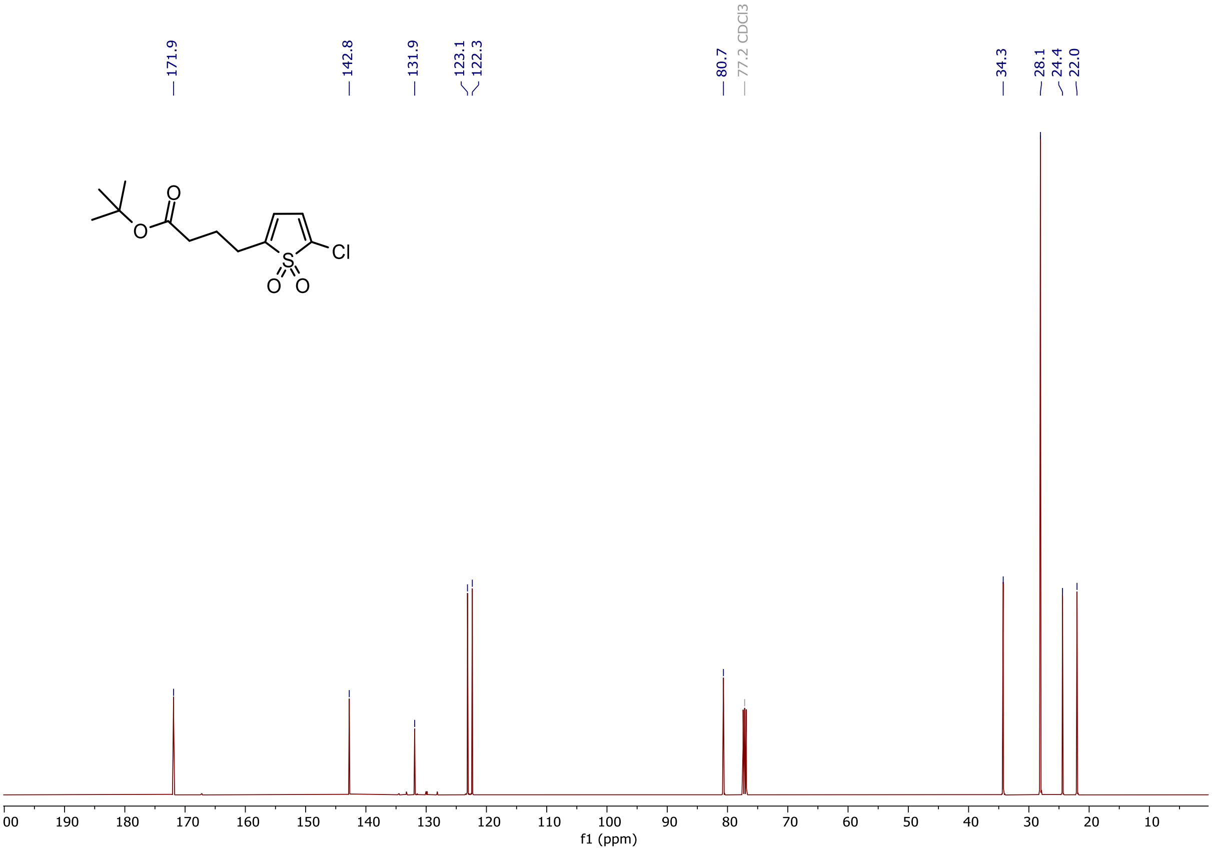

Compound 17:

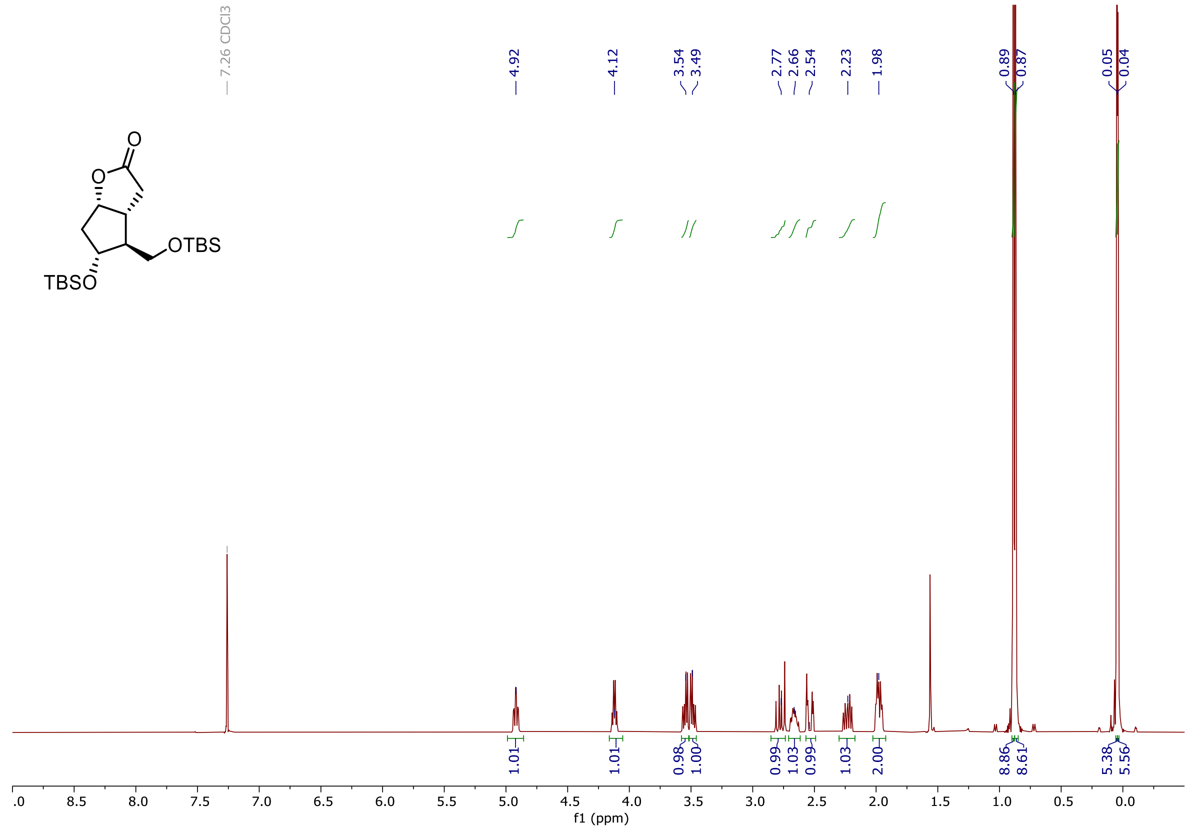
7.26 CDCl3
4.92
4.12
3.54
3.49
2.77
2.66
2.54
2.23
1.98
0.89
0.87
0.05
0.04
O
O
OTBS
TBSO
1.01
1.01
0.98
1.00
0.99
1.03
0.99
1.03
2.00
8.86
8.61
5.38
5.56
f1 (ppm)

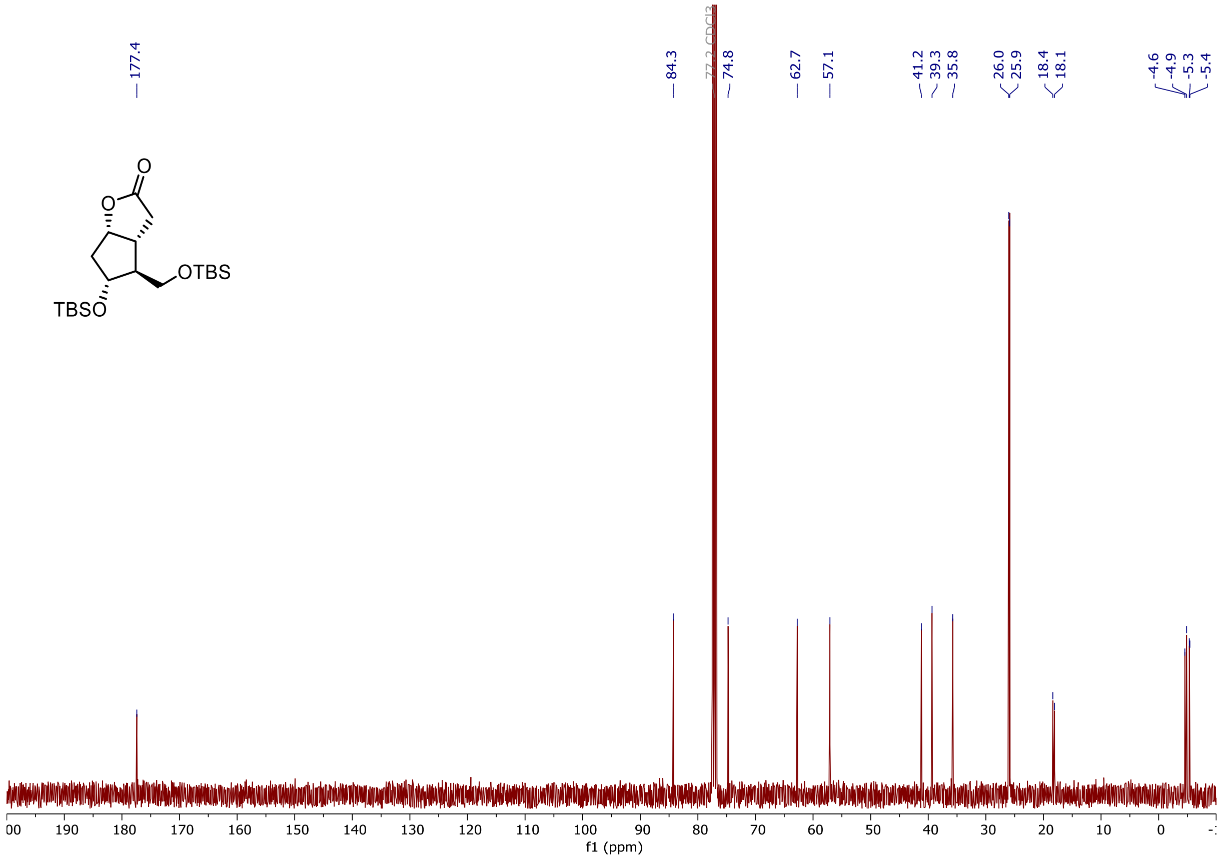
177.4
84.3
74.8
62.7
57.1
41.2
39.3
35.8
26.0
25.9
18.4
18.1
-4.6
-4.9
-5.3
-5.4
O
O
OTBS
TBSO
f1 (ppm)

Compound 18:

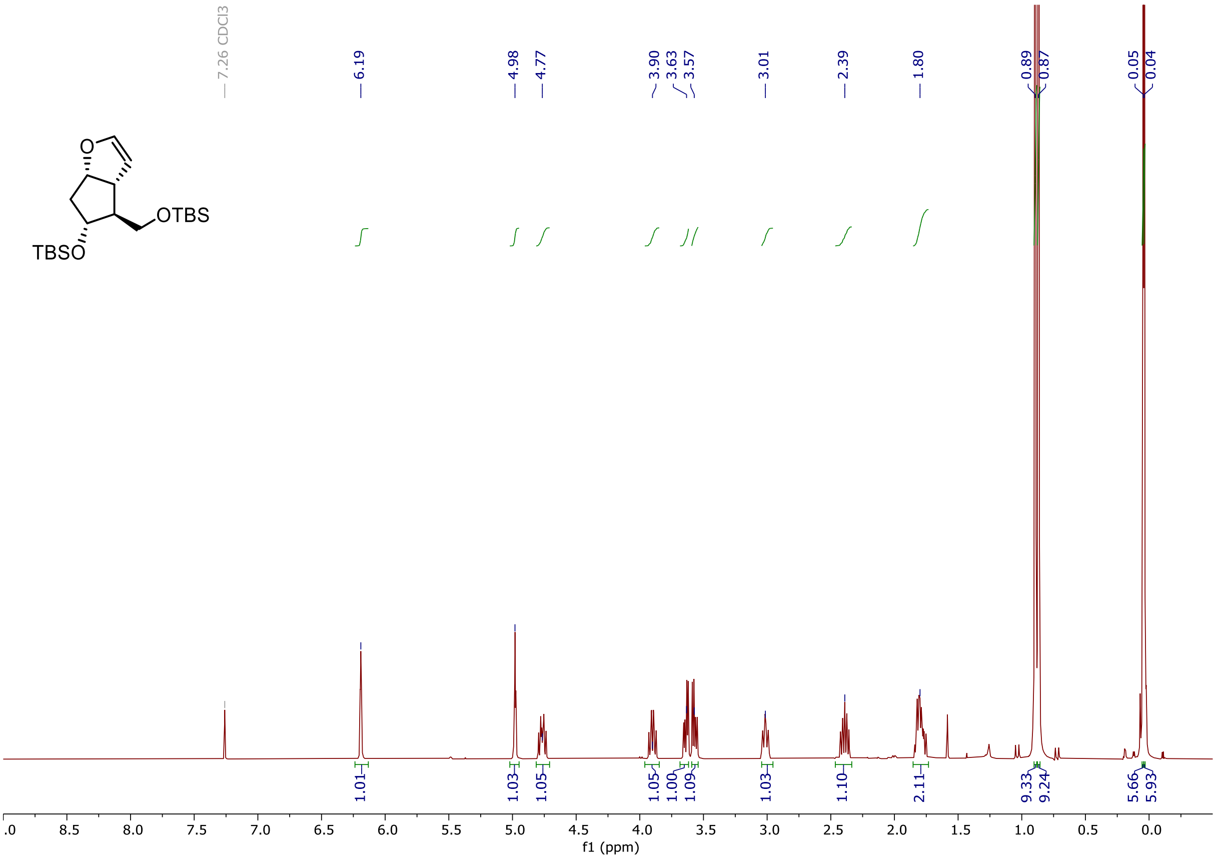

7.26 CDCl3
6.19
4.98
4.77
3.90
3.63
3.57
3.01
2.39
1.80
0.89
0.87
0.05
0.04
OTBS
TBSO
1.01
1.03
1.05
1.05
1.00
1.09
1.03
1.10
2.11
9.33
9.24
5.66
5.93
f1 (ppm)

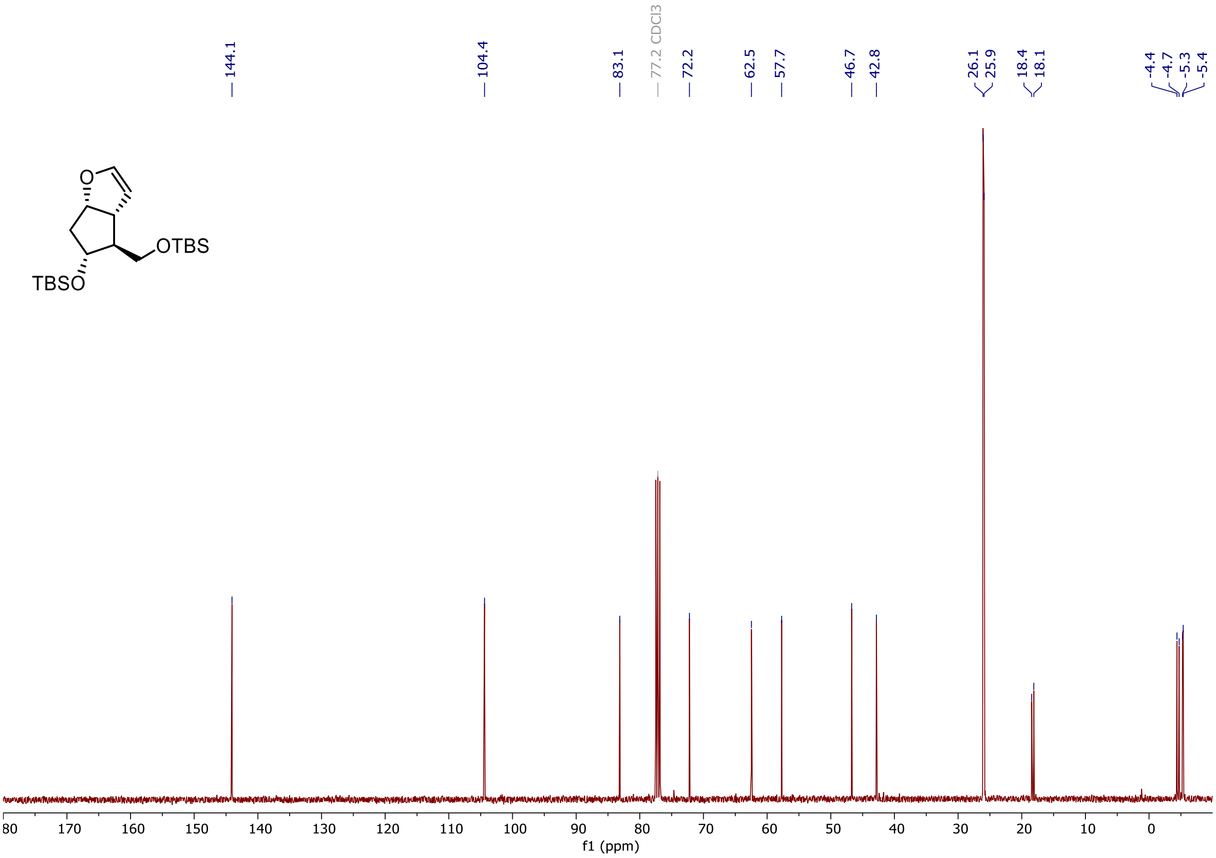

144.1
104.4
83.1
77.2 CDCl3
72.2
62.5
57.7
46.7
42.8
26.1
25.9
18.4
18.1
-4.4
-4.7
-5.3
-5.4
OTBS
TBSO
f1 (ppm)

Compound 20a:

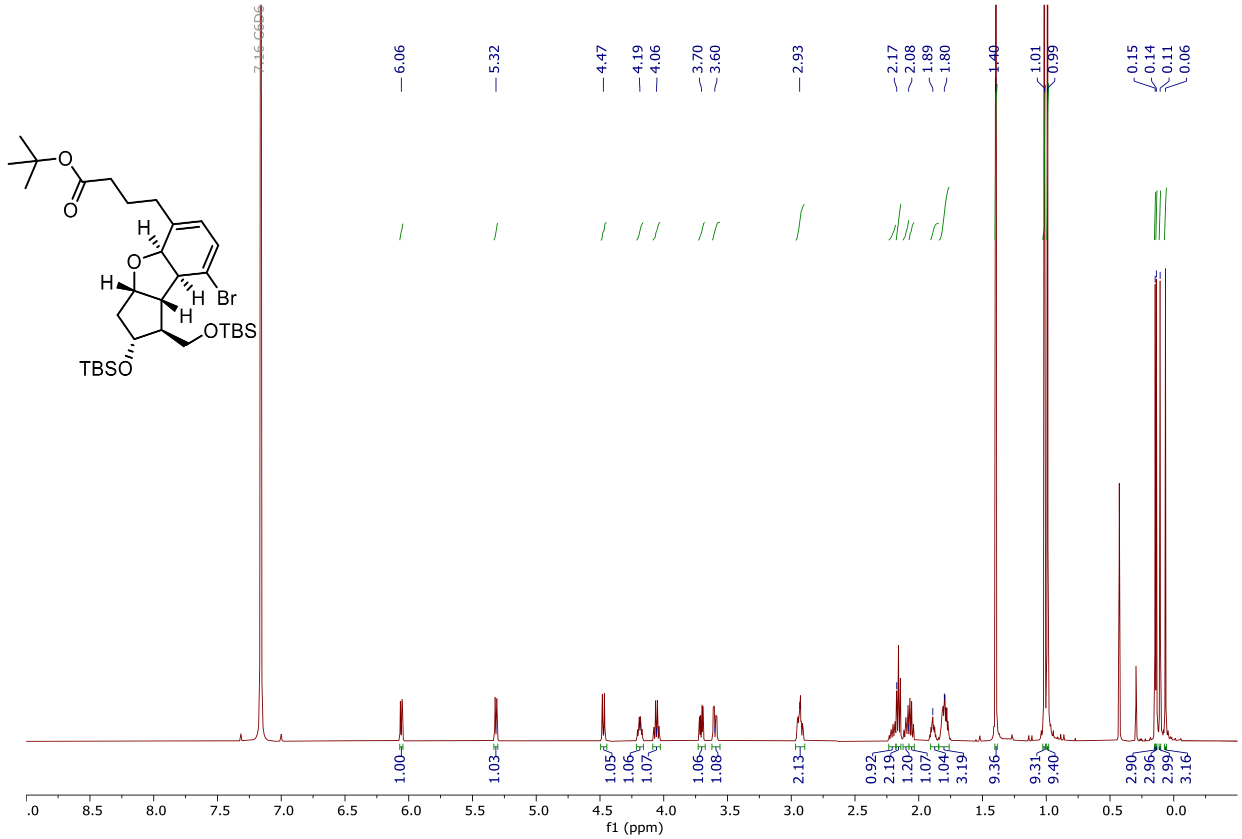

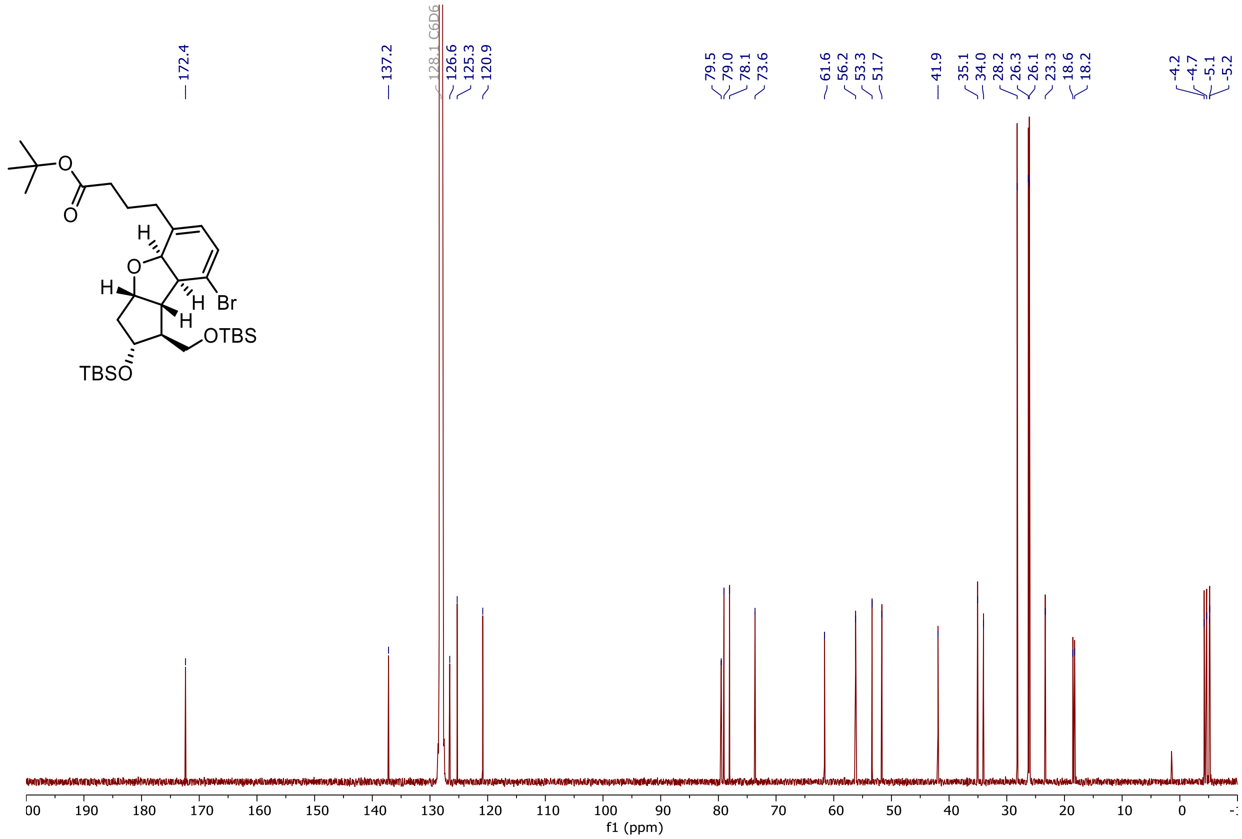

HMBC: Major regioisomer HMBC correlations

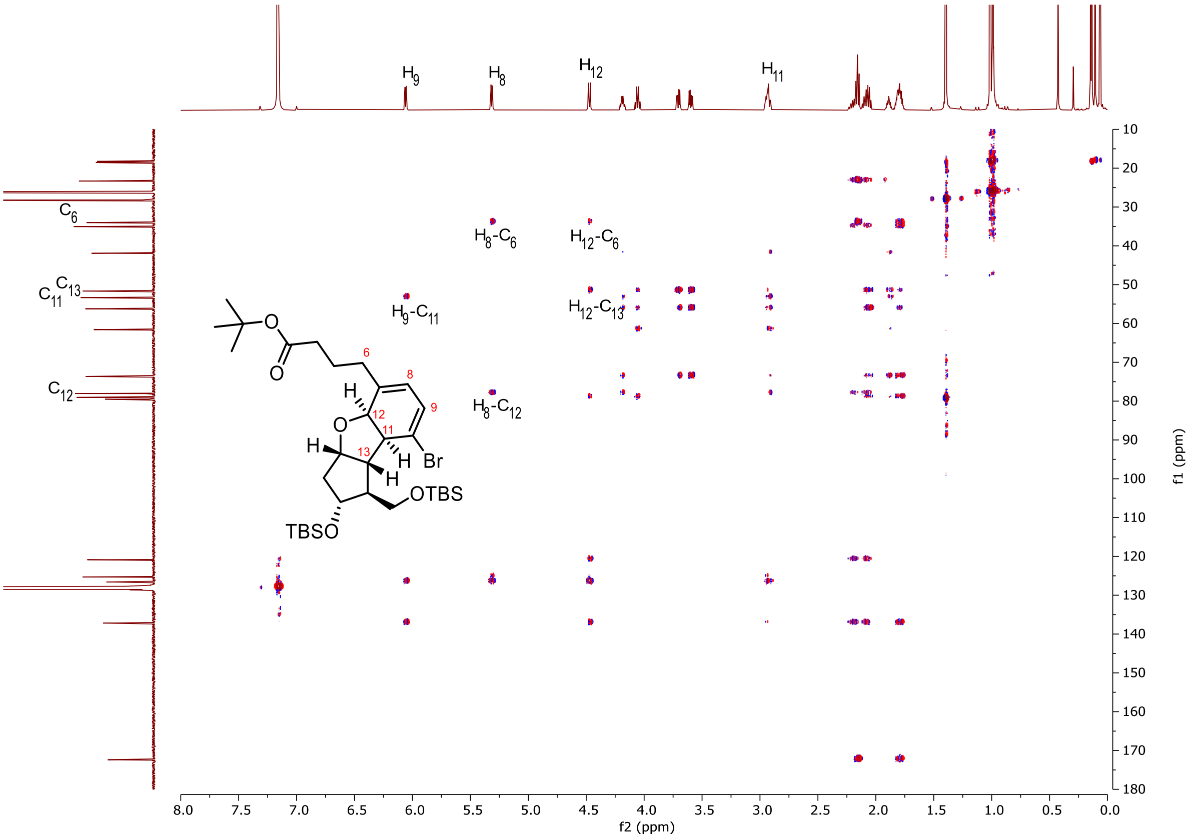

Compound 20b:

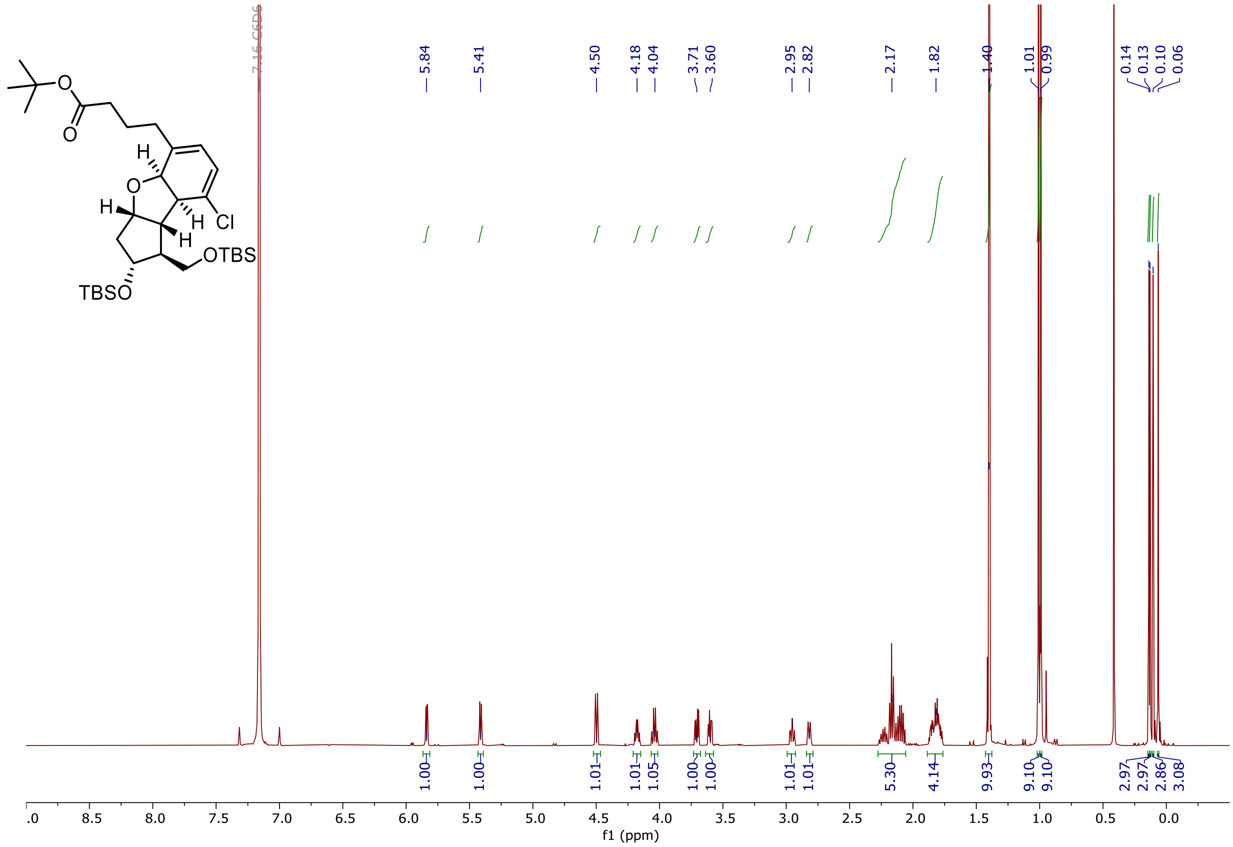

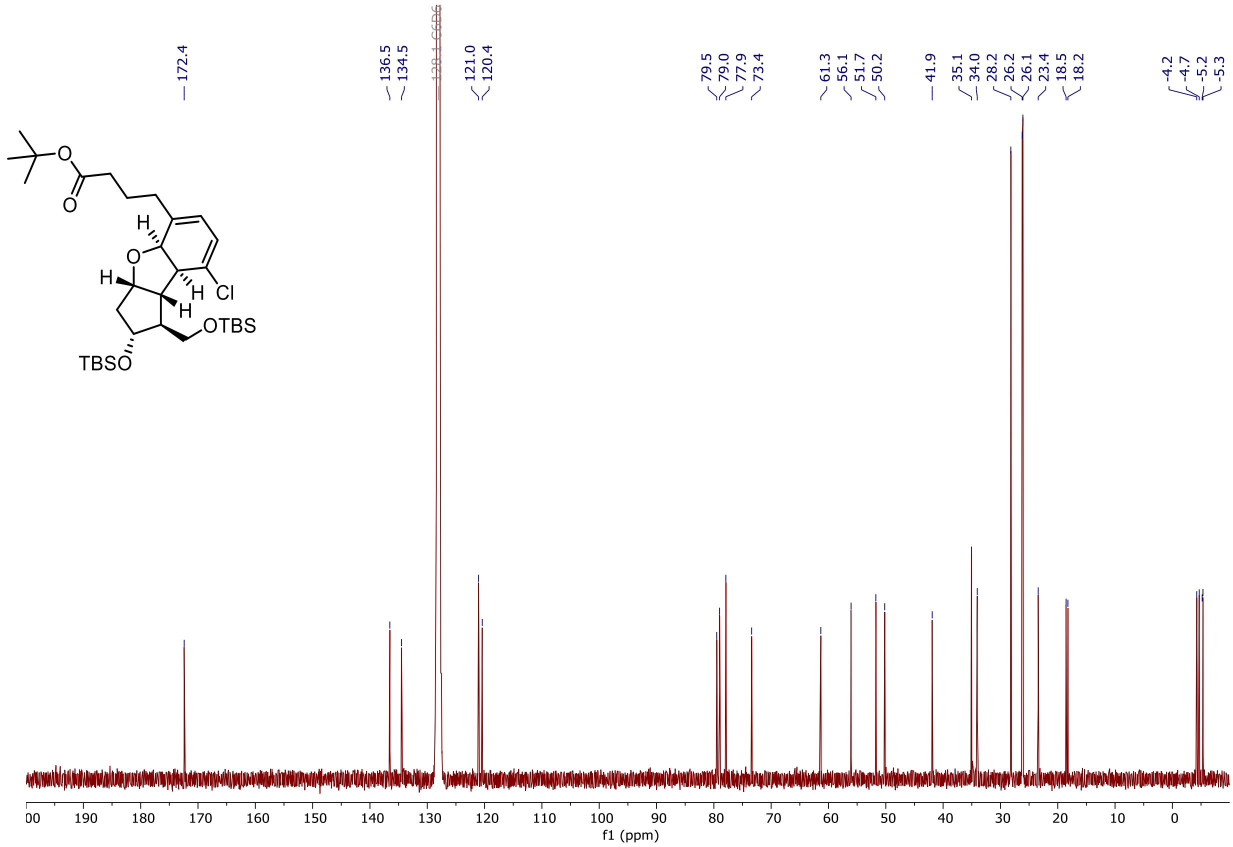

Compound 20c:

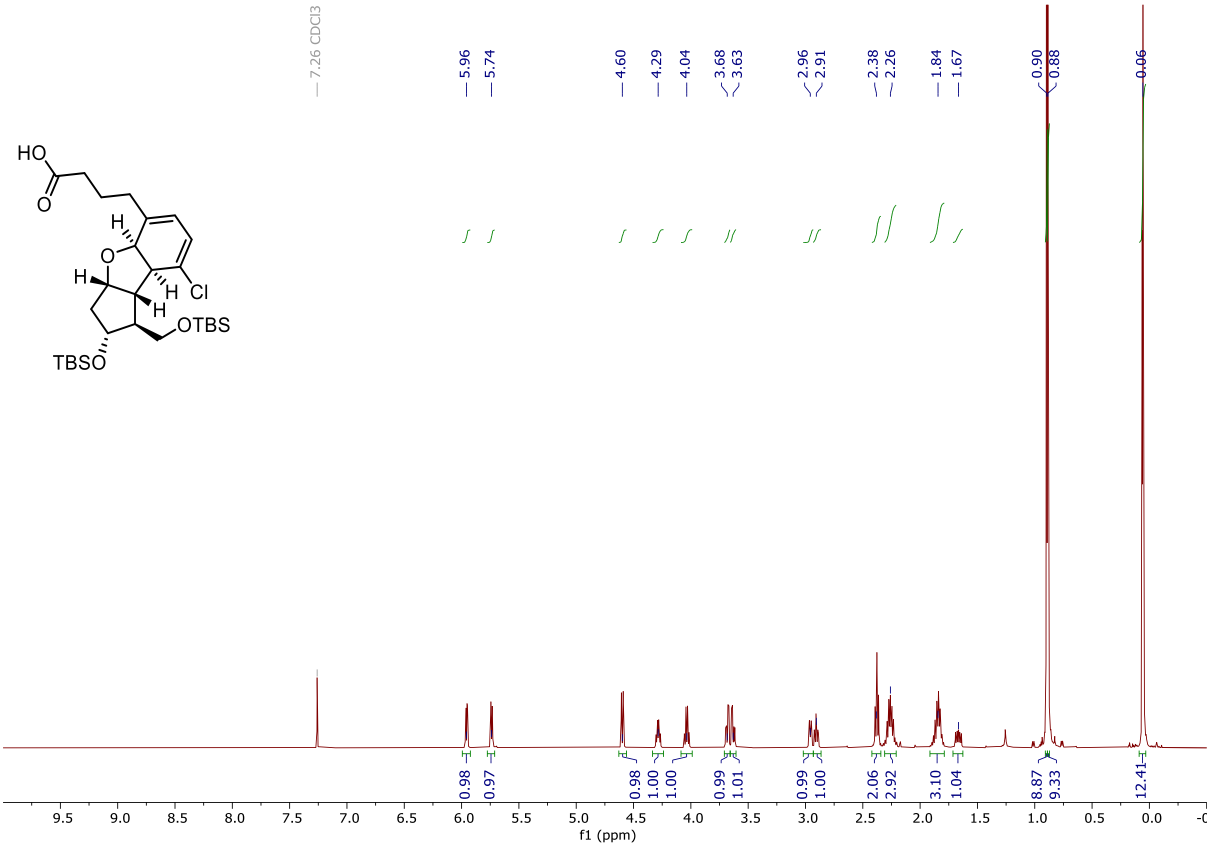

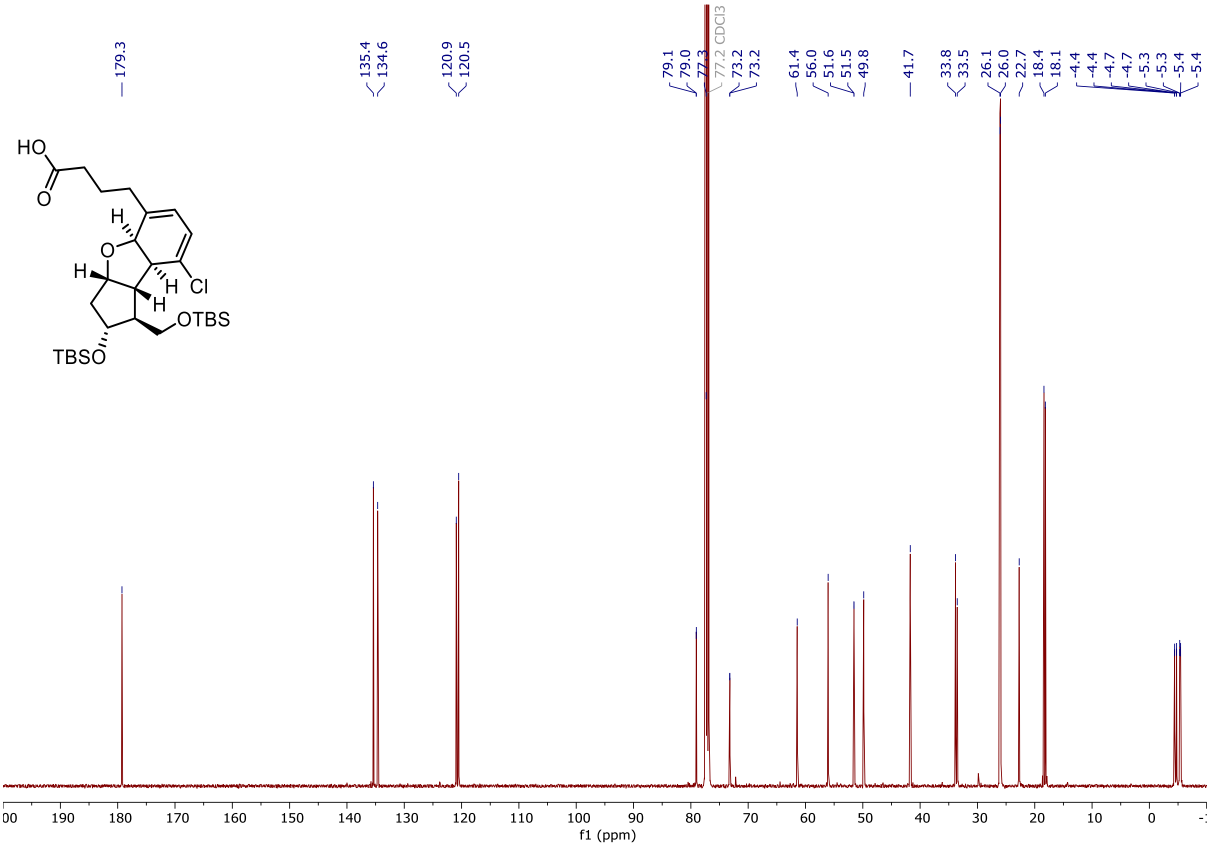

Compound 21a:

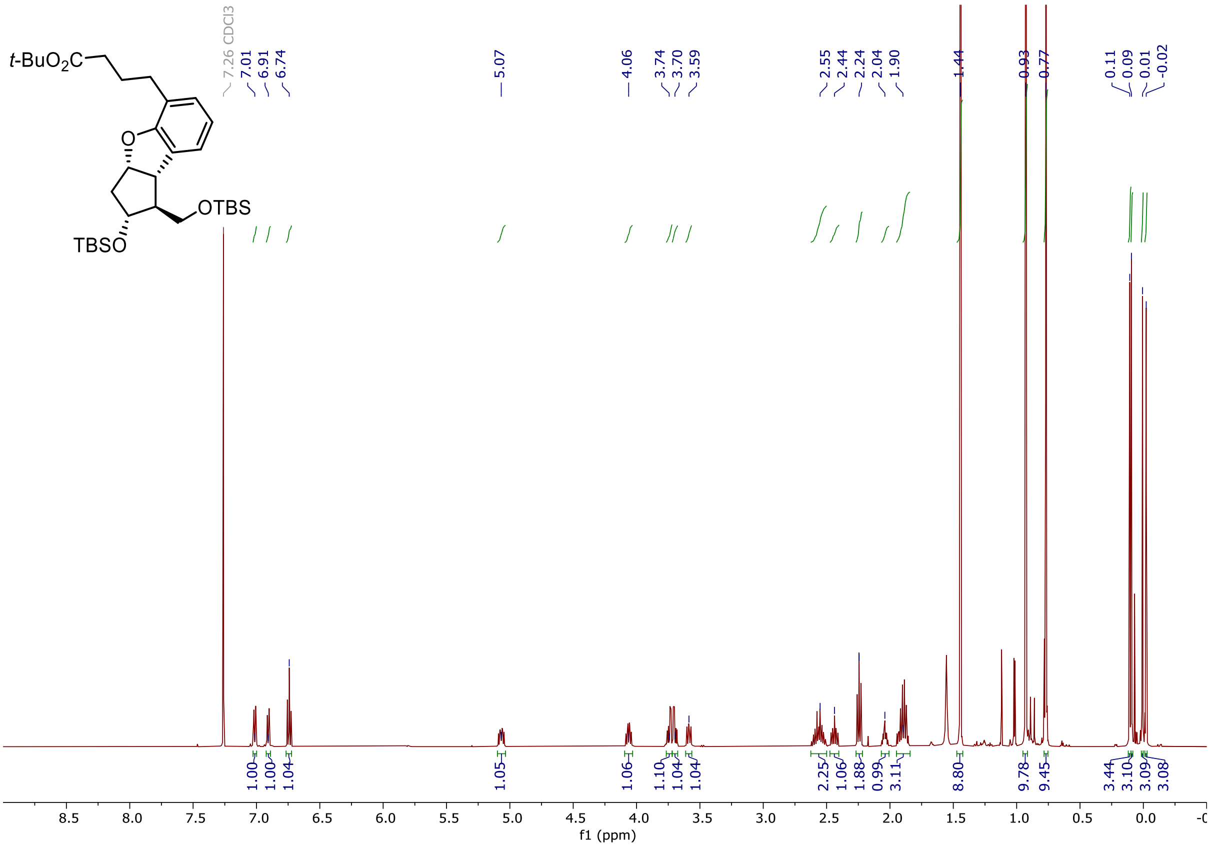

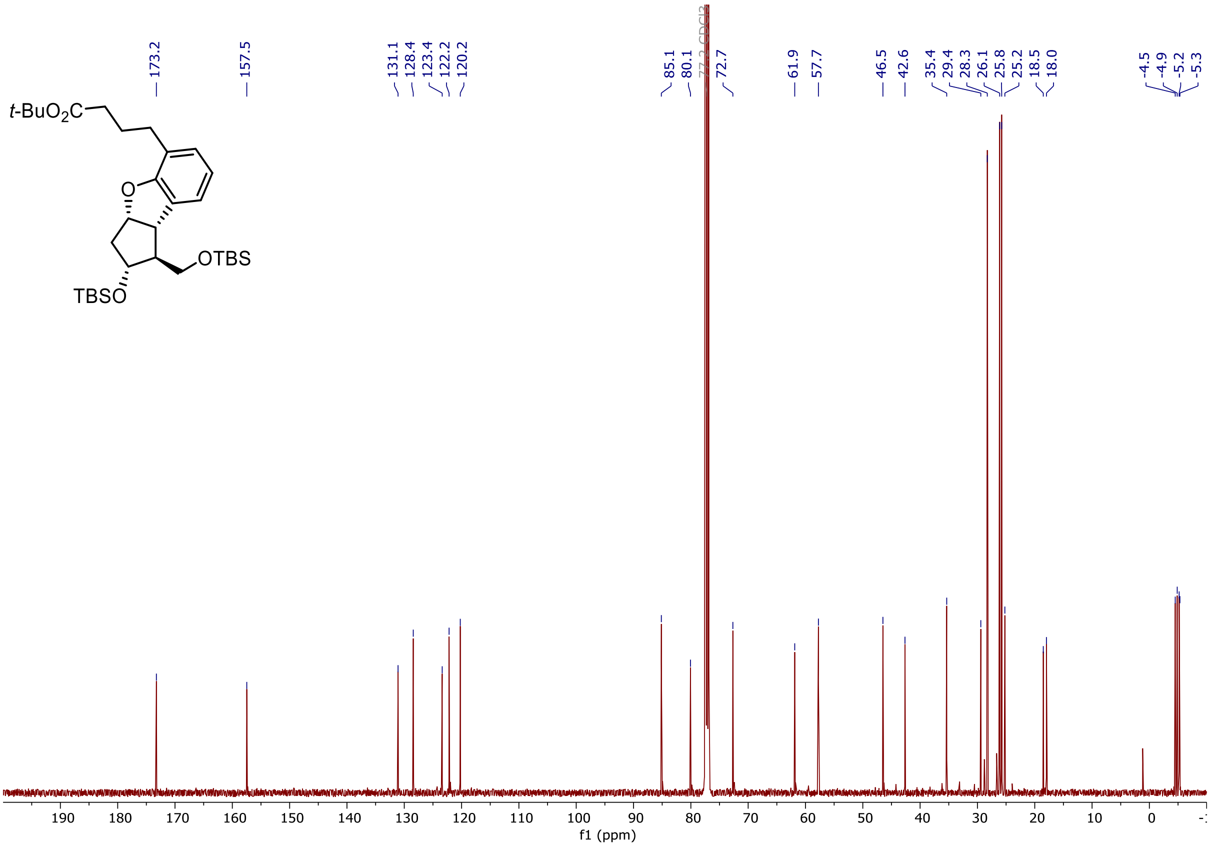

Compound 21b:

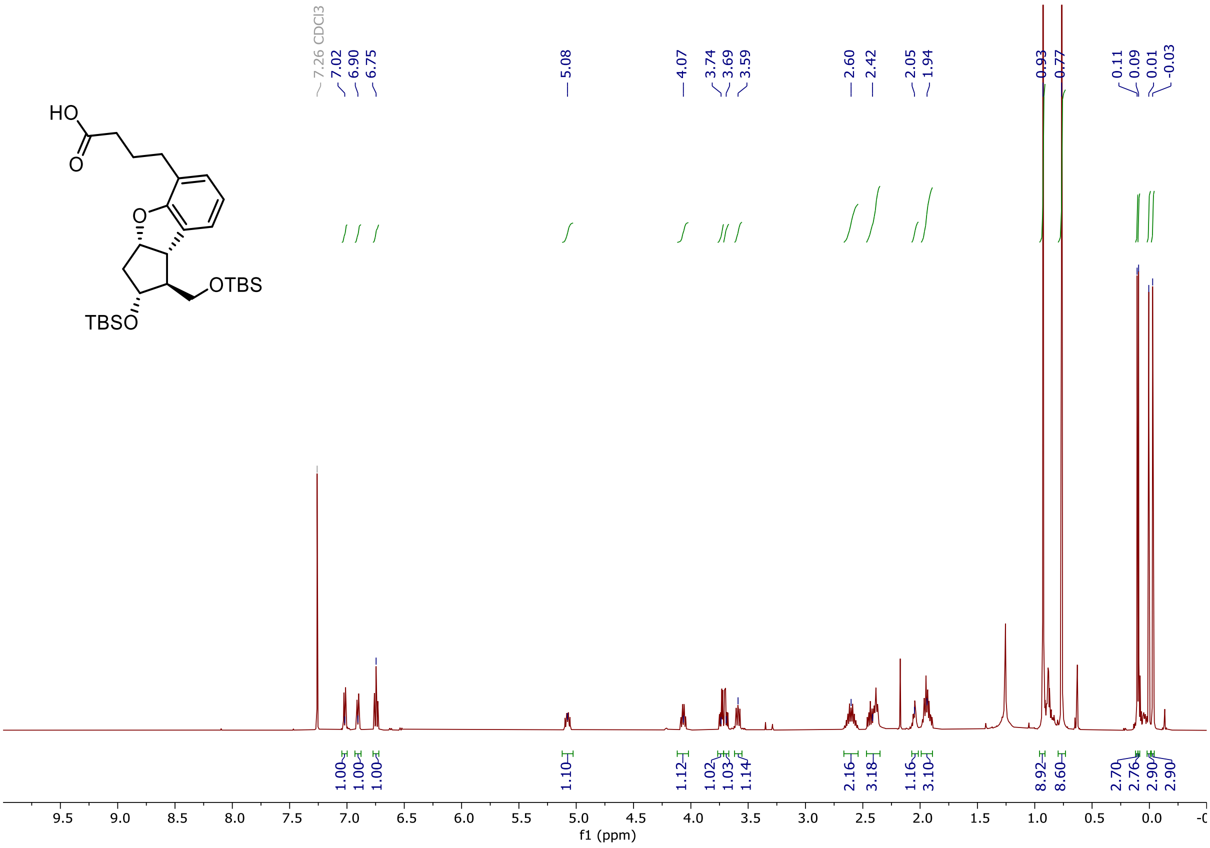

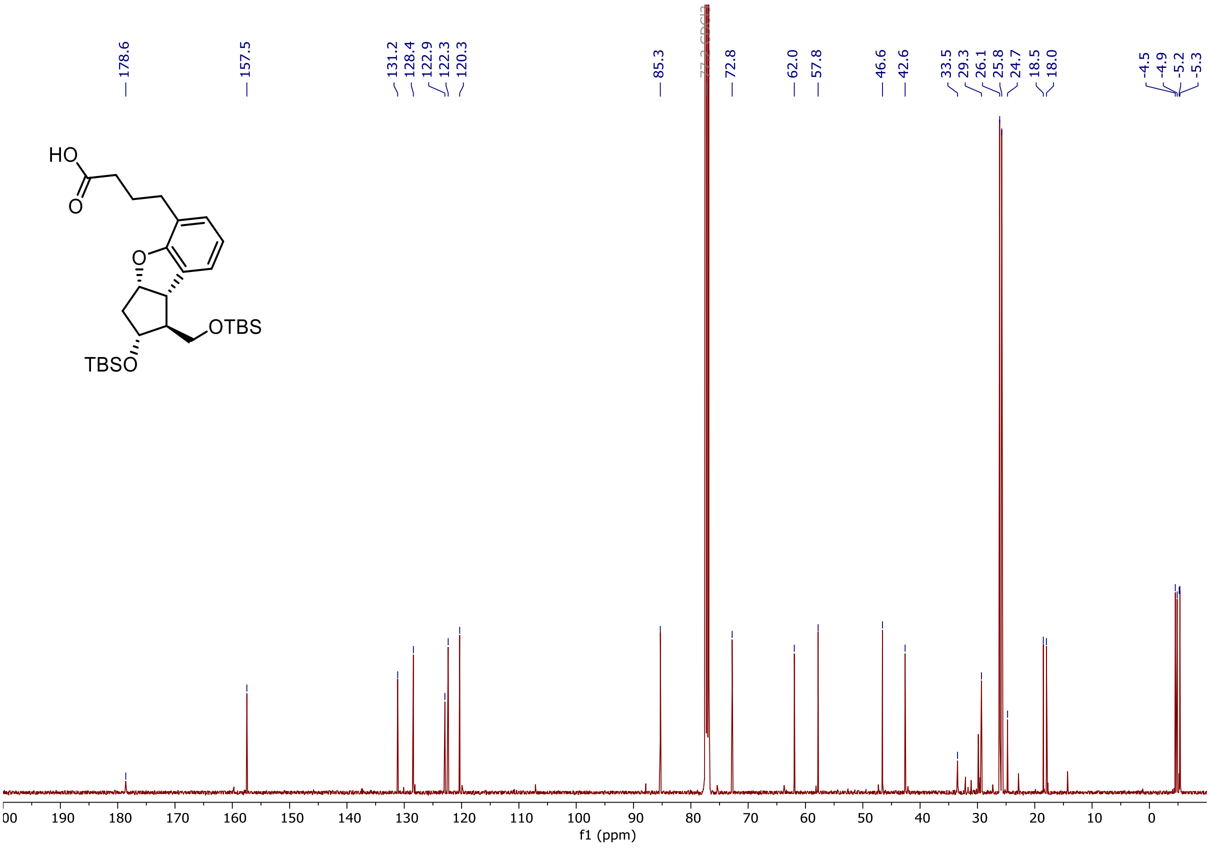

8.3. Deuterium-Labelling Studies

Compound 58:

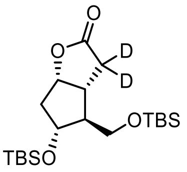

[97% deuterium incorporation]

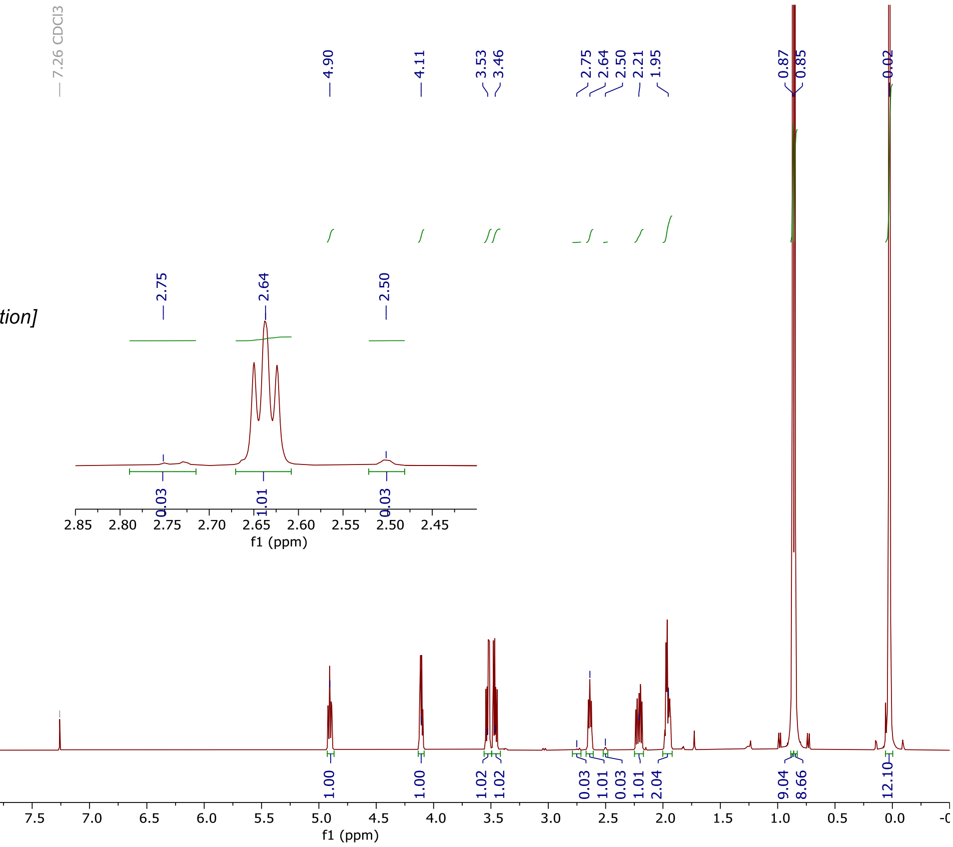

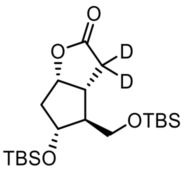

[97% deuterium incorporation]

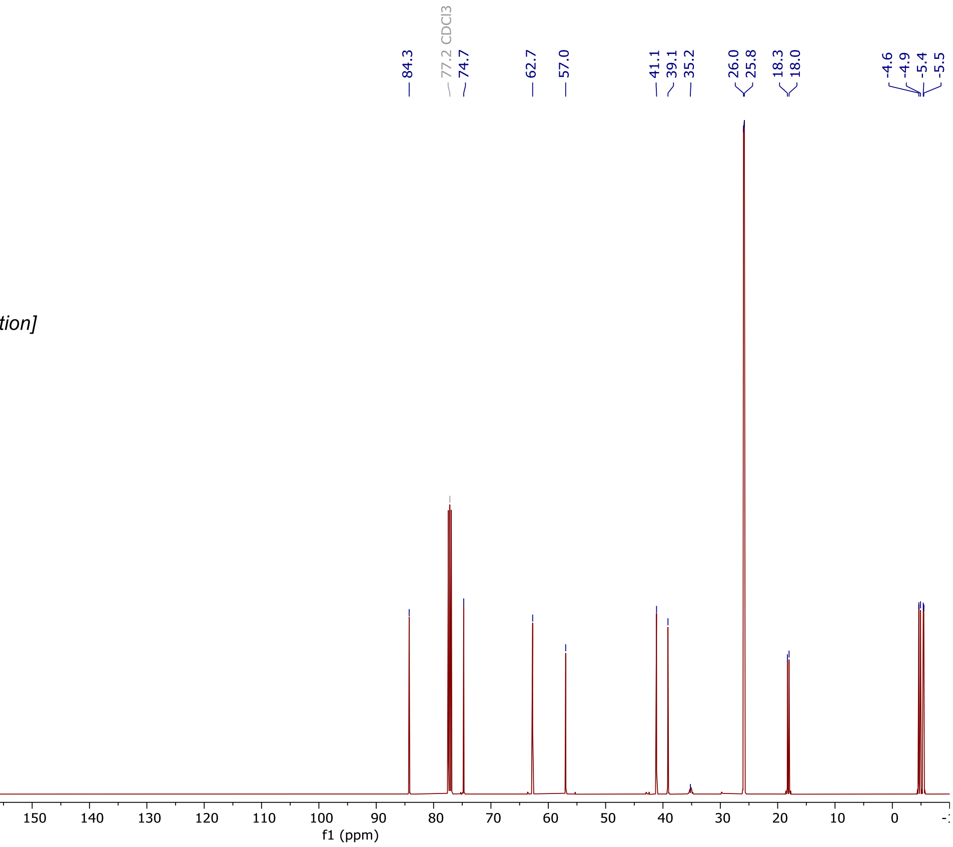

Compound 59:

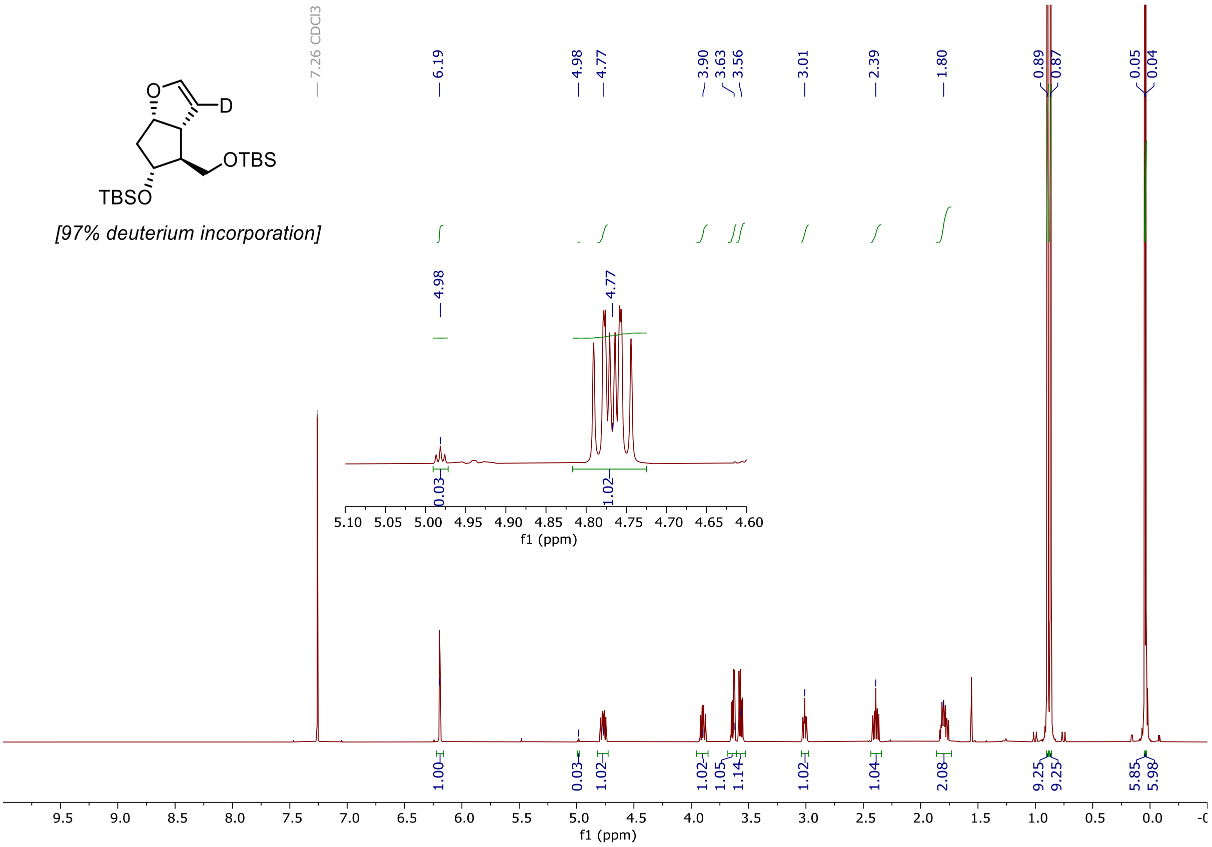
O
D
OTBS
TBSO
[97% deuterium incorporation]
7.26 CDCl3
6.19
4.98
4.77
3.90
3.63
3.56
3.01
2.39
1.80
0.89
0.87
0.05
0.04
1.00
0.03
1.02
1.02
1.05
1.14
1.02
1.04
2.08
9.25
9.25
5.85
5.98
f1 (ppm)

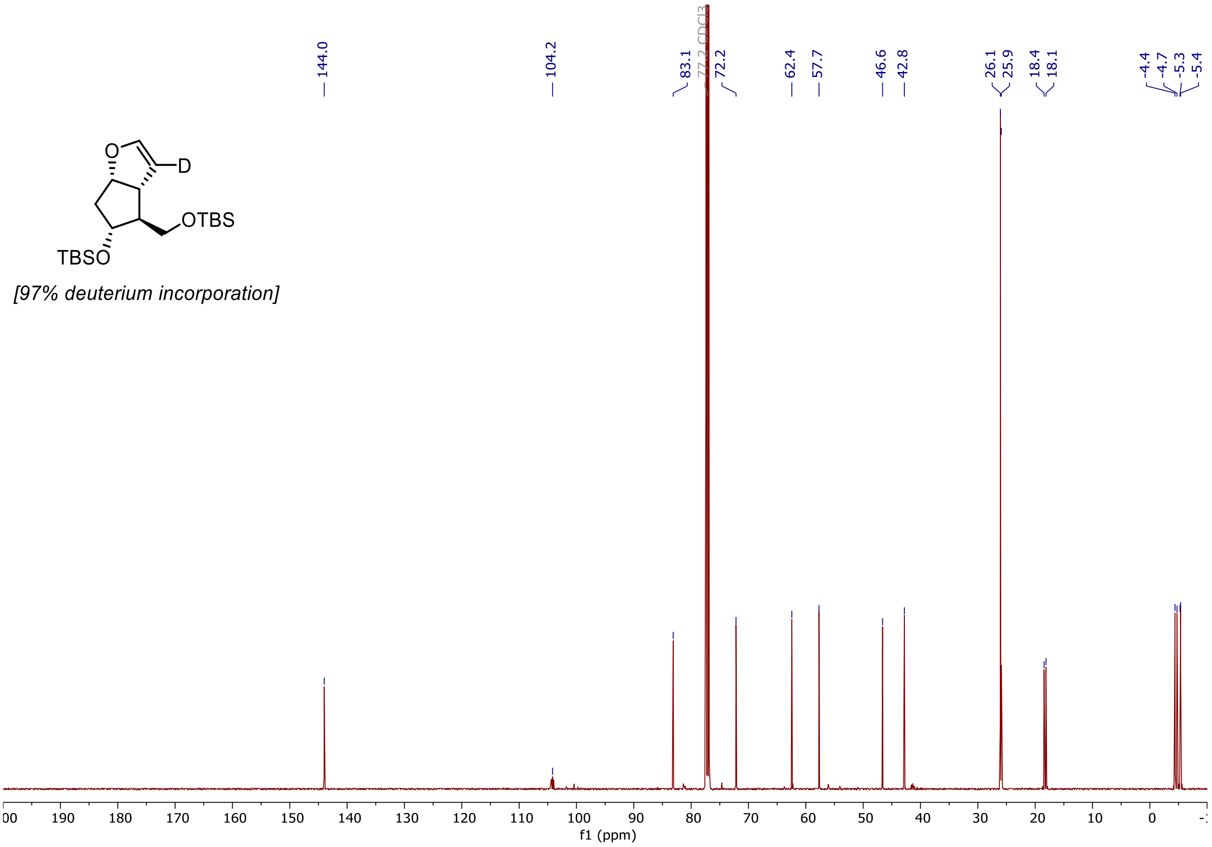
O
D
OTBS
TBSO
[97% deuterium incorporation]
144.0
104.2
83.1
77.2 CDCl3
72.2
62.4
57.7
46.6
42.8
26.1
25.9
18.4
18.1
-4.4
-4.7
-5.3
-5.4
f1 (ppm)

Compound 23a:

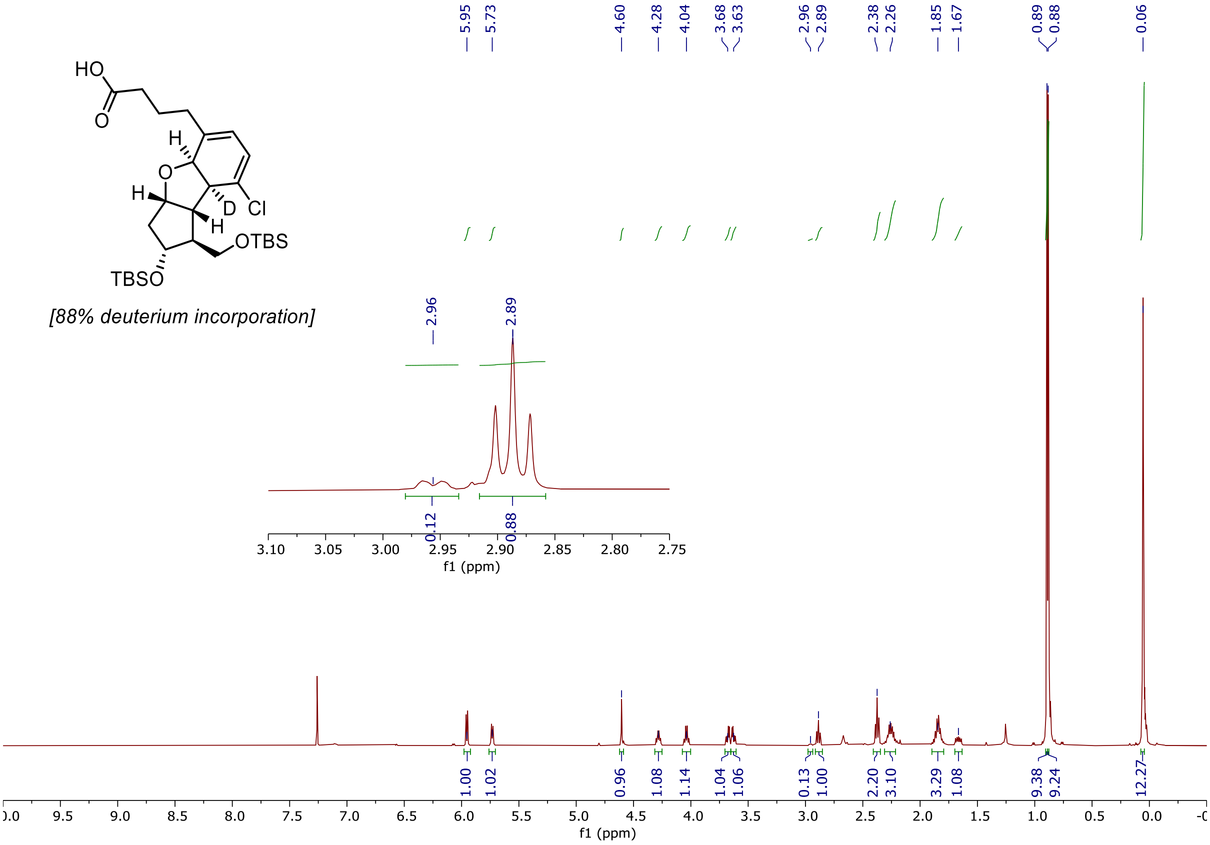

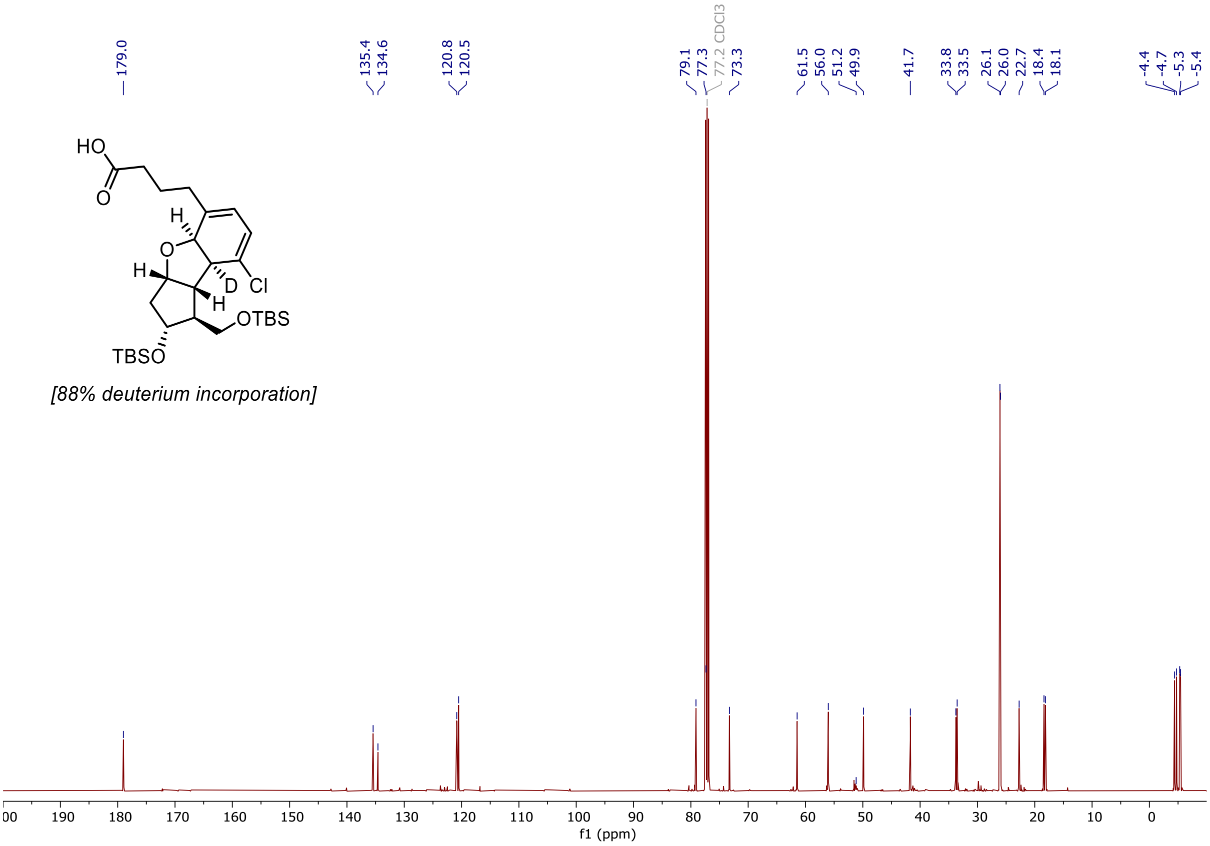

Compound 24:

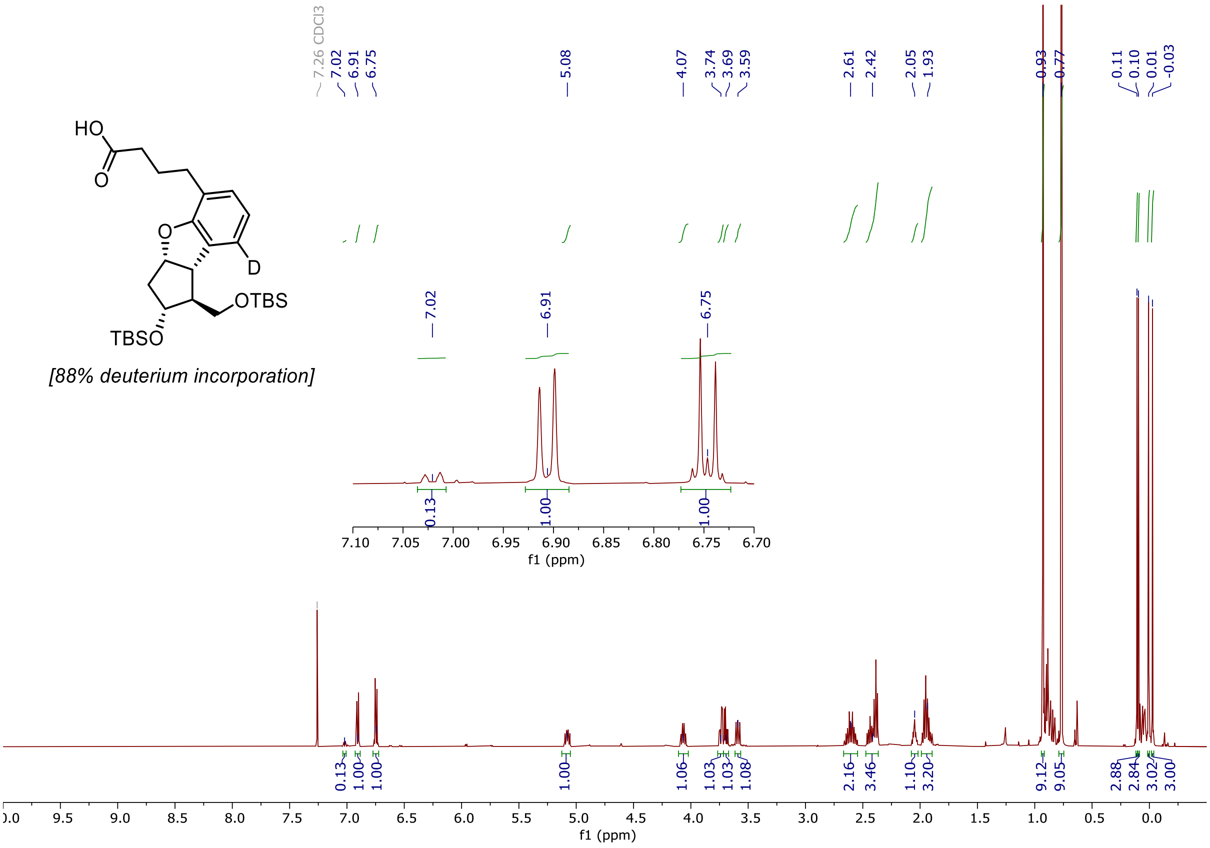

HO
O
O
D
OTBS
TBSO
[88% deuterium incorporation]
7.26 CDCl3
7.02
6.91
6.75
5.08
4.07
3.74
3.69
3.59
2.61
2.42
2.05
1.93
0.93
0.77
0.11
0.10
0.01
-0.03
0.13
1.00
1.00
7.10 7.05 7.00 6.95 6.90 6.85 6.80 6.75 6.70
f1 (ppm)
0.13 1.00 1.00 1.00 1.06 1.03 1.03 1.08 2.16 3.46 1.10 3.20 9.12 9.05 2.88 2.84 3.02 3.00
9.5 9.0 8.5 8.0 7.5 7.0 6.5 6.0 5.5 5.0 4.5 4.0 3.5 3.0 2.5 2.0 1.5 1.0 0.5 0.0
f1 (ppm)

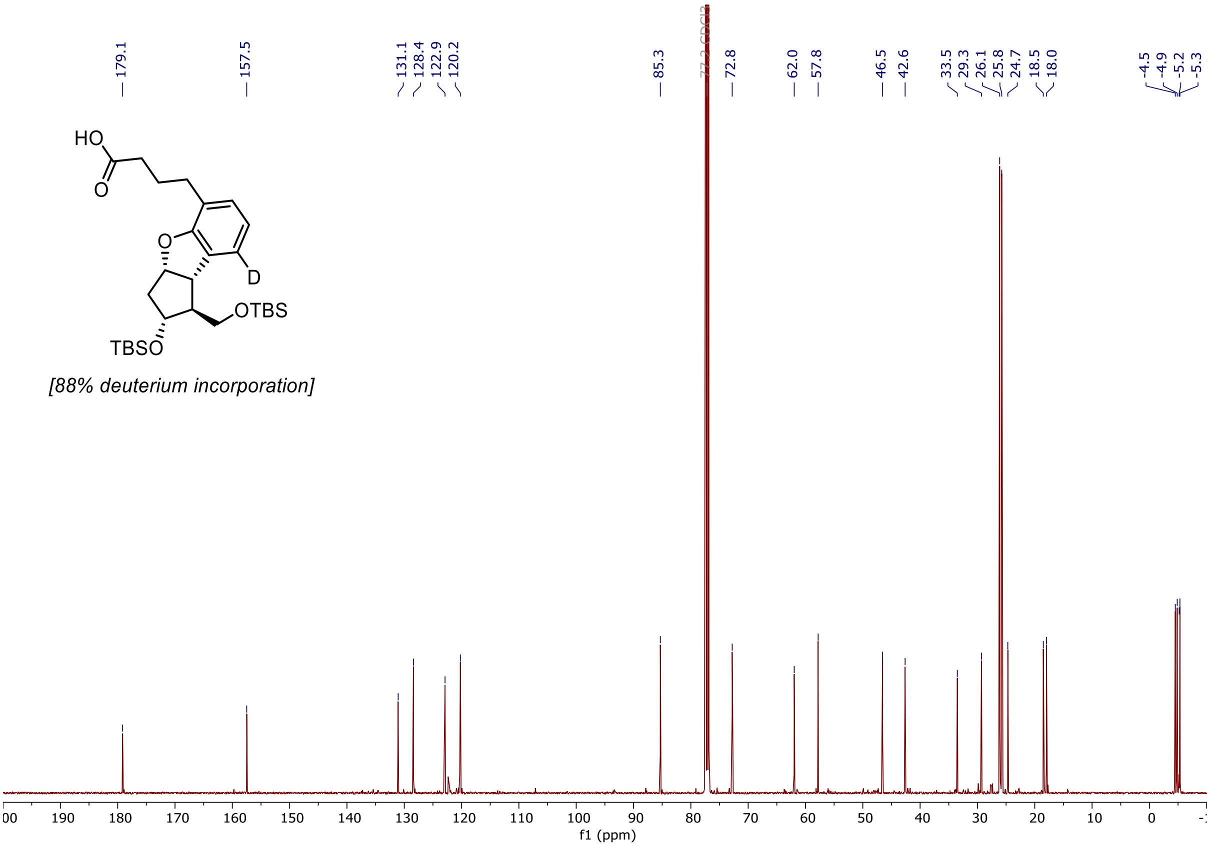

HO
O
O
D
OTBS
TBSO
[88% deuterium incorporation]
179.1
157.5
131.1
128.4
122.9
120.2
85.3
72.8
62.0
57.8
46.5
42.6
33.5
29.3
26.1
25.8
24.7
18.5
18.0
-4.5
-4.9
-5.2
-5.3
190 180 170 160 150 140 130 120 110 100 90 80 70 60 50 40 30 20 10 0
f1 (ppm)

Compound 60:

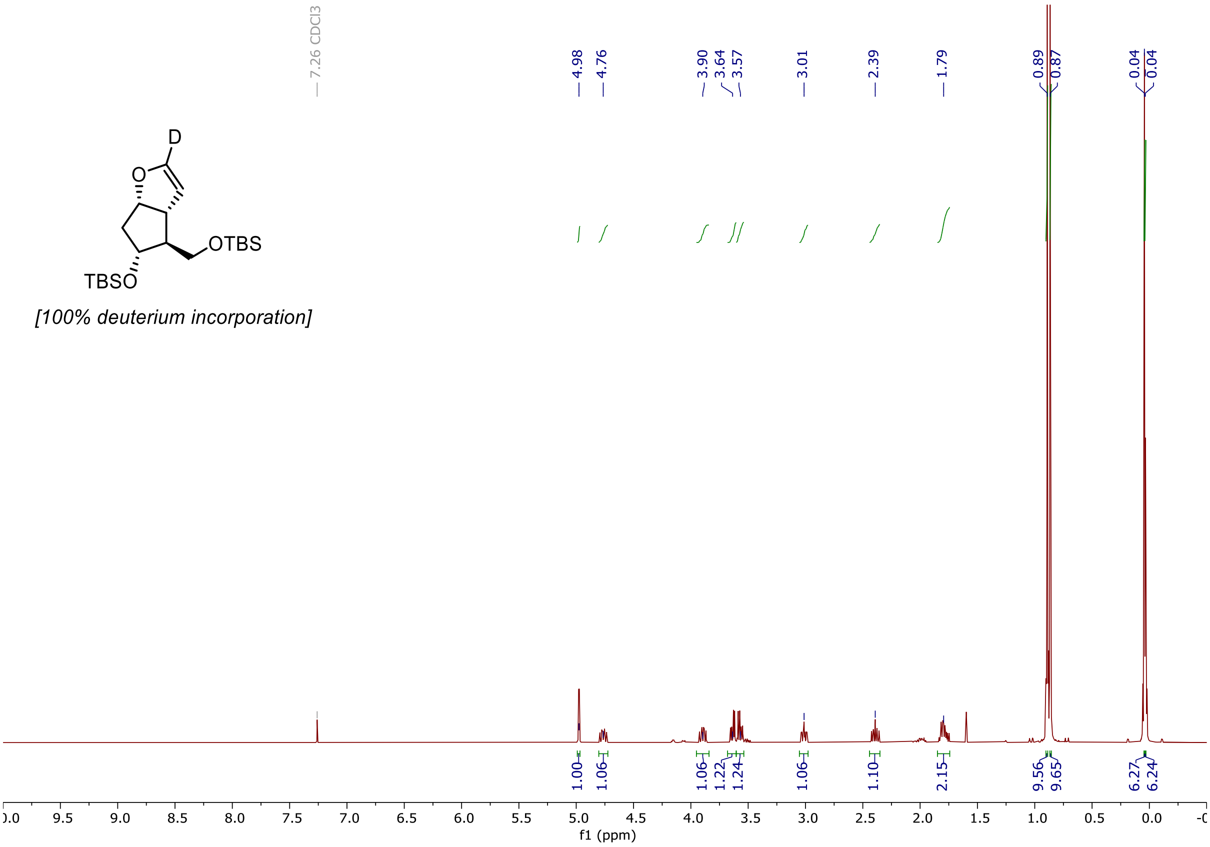

[100% deuterium incorporation]

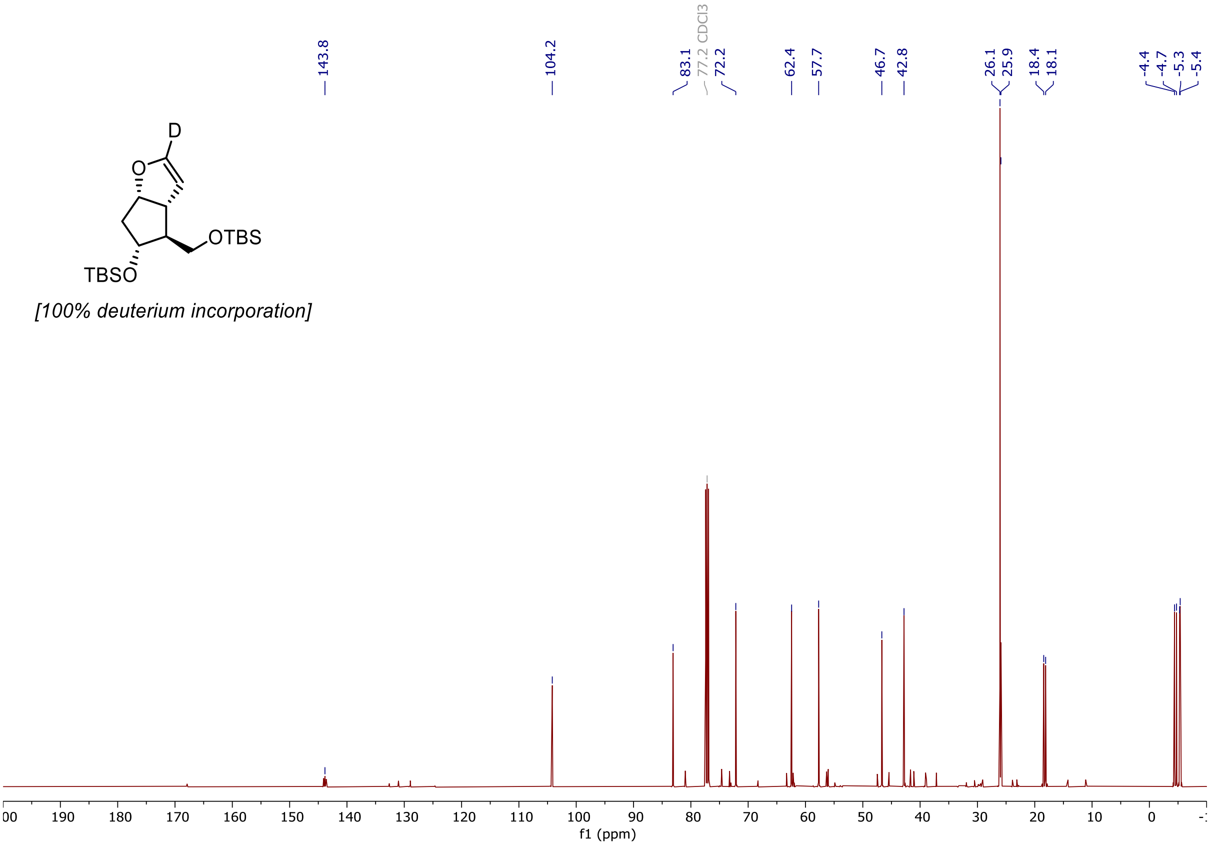

[100% deuterium incorporation]

Compound 23b:

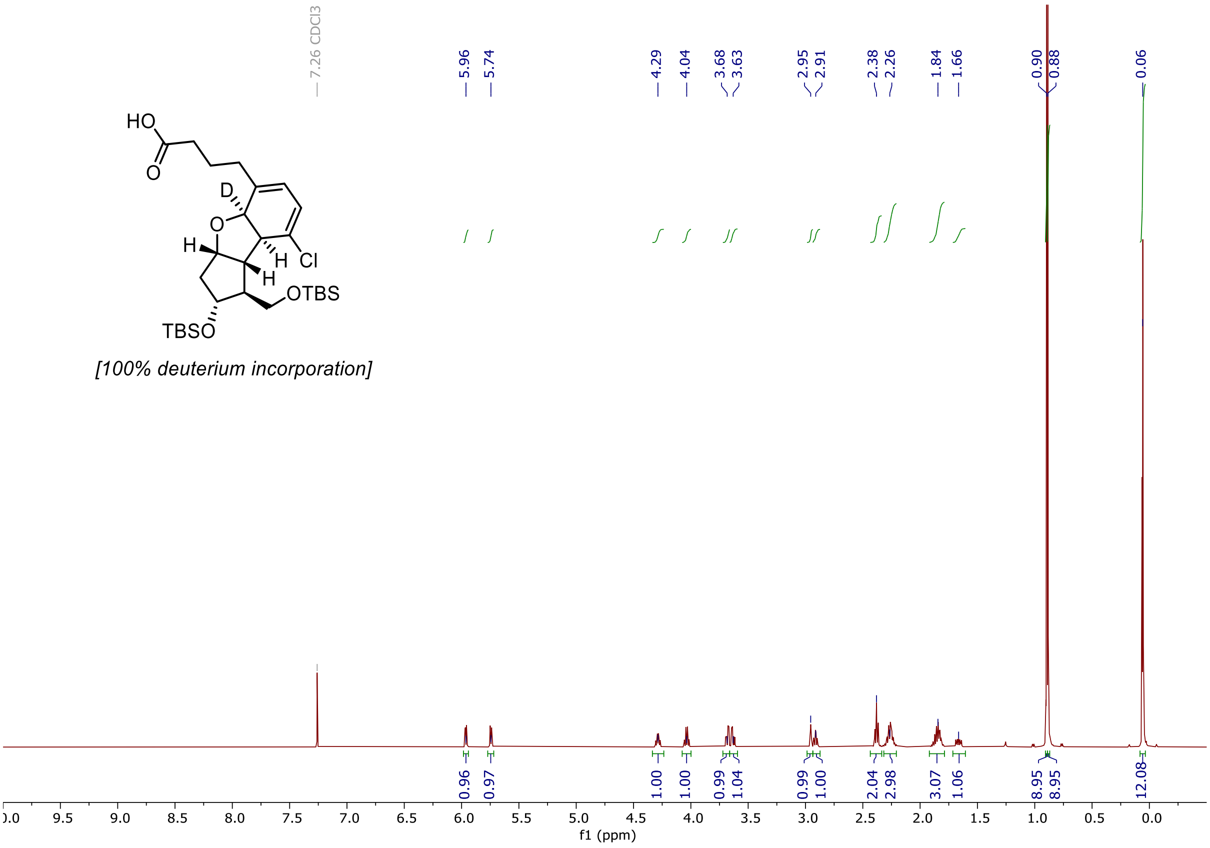

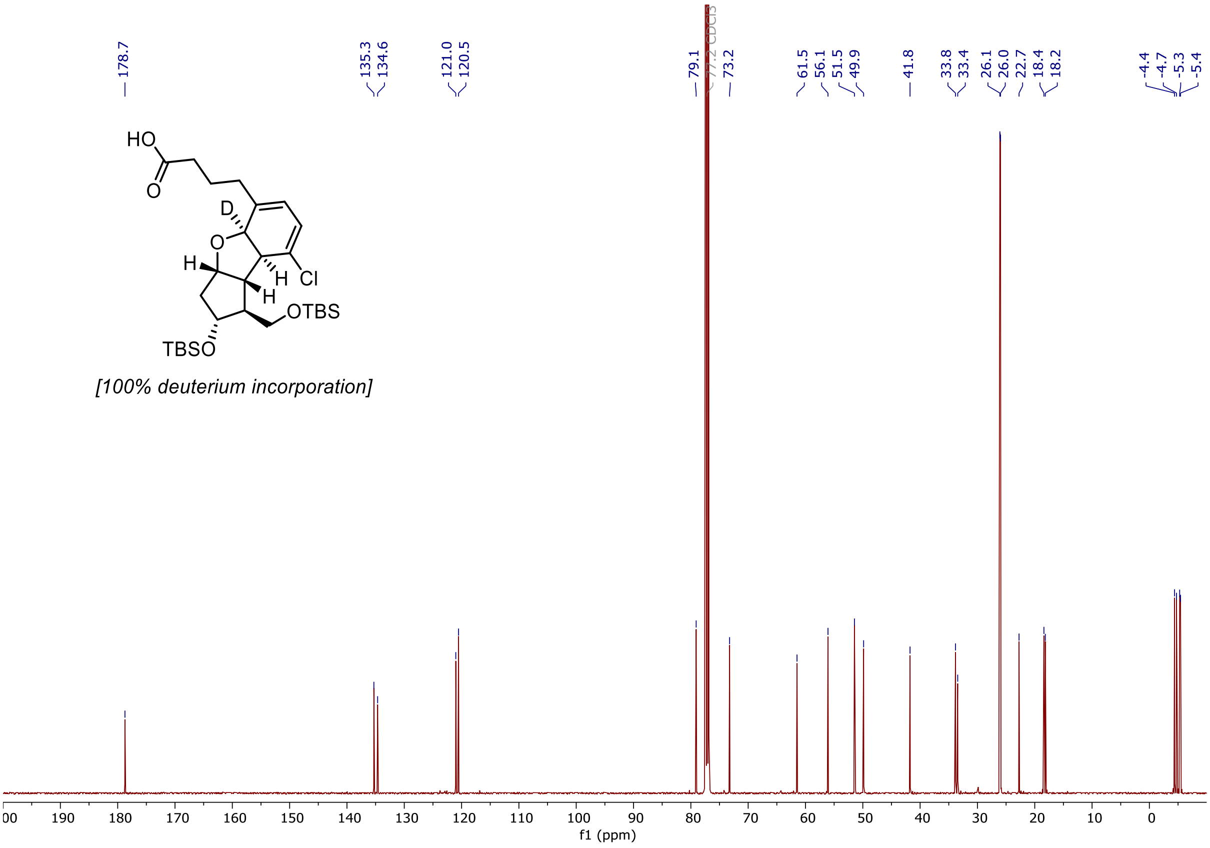

8.4. Substrate Scope

Compound 42a:

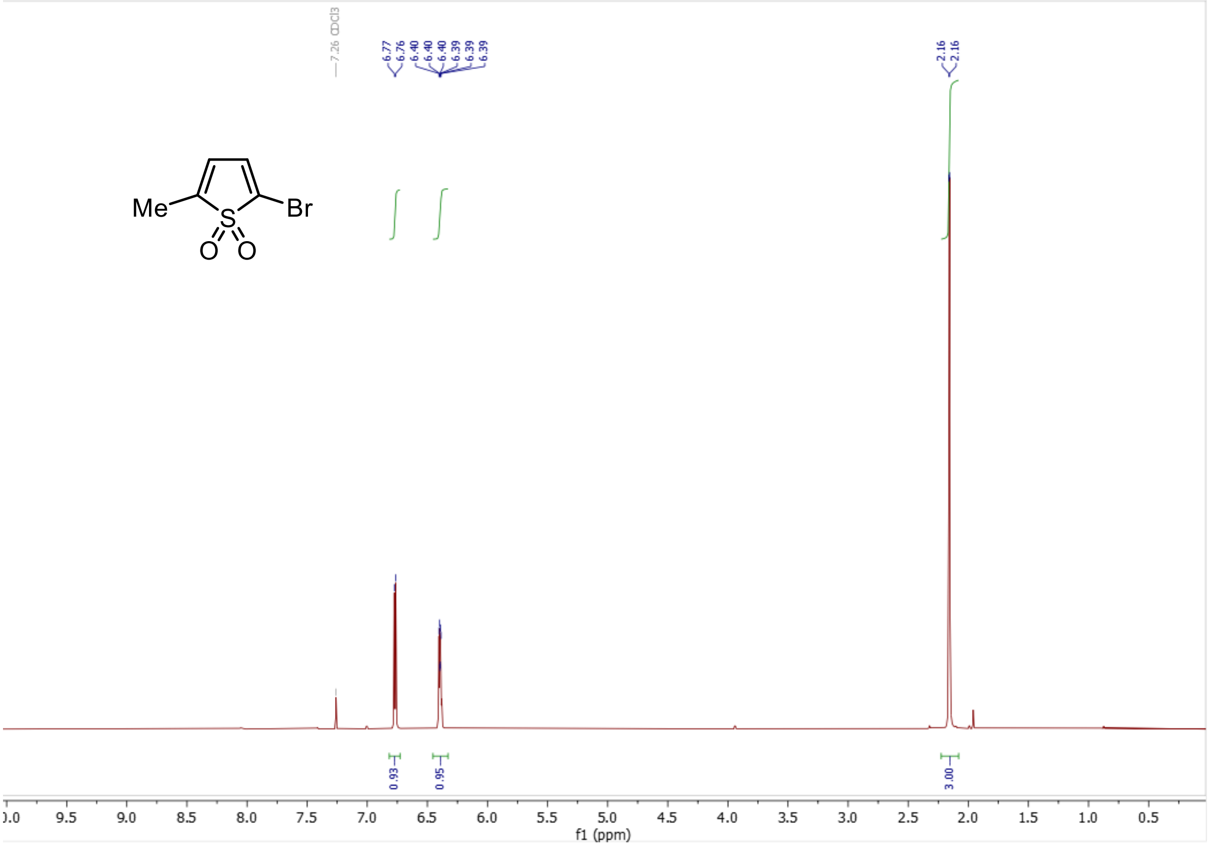

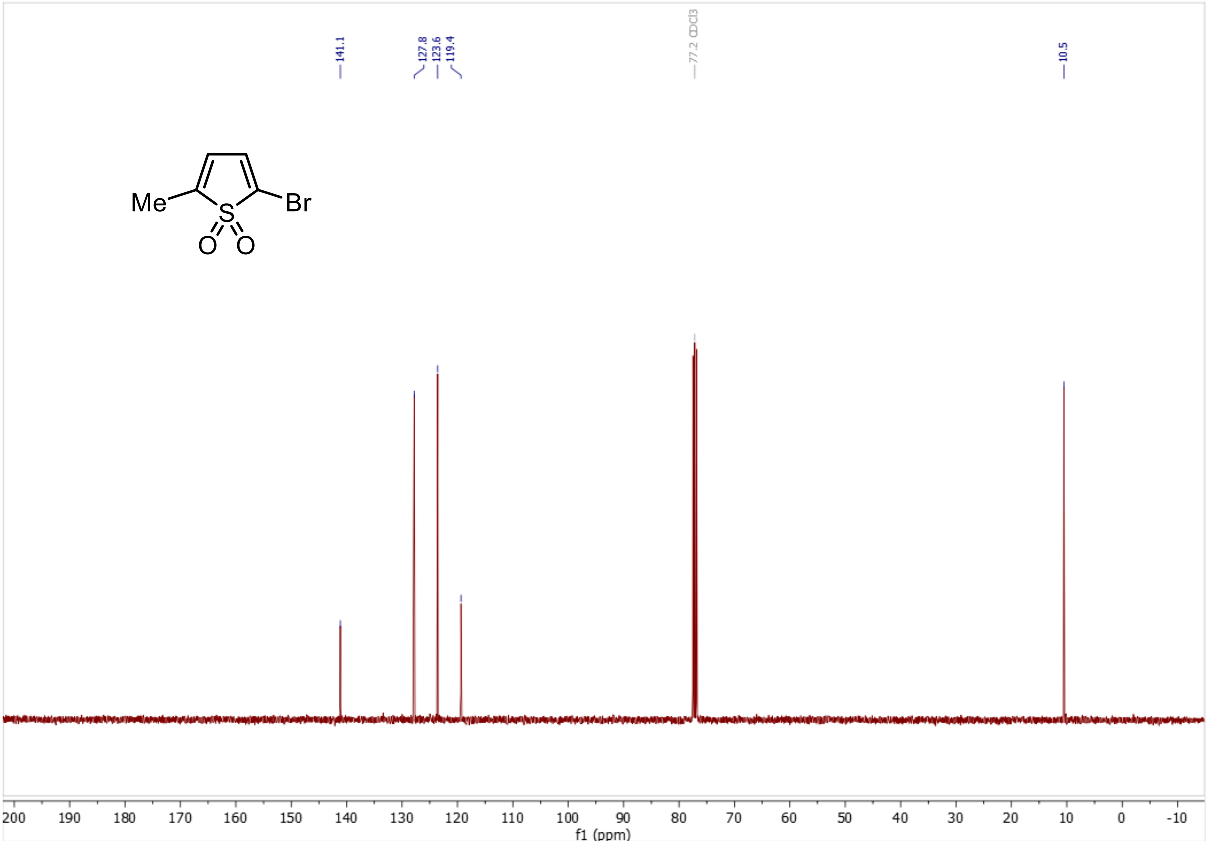

Compound 42c:

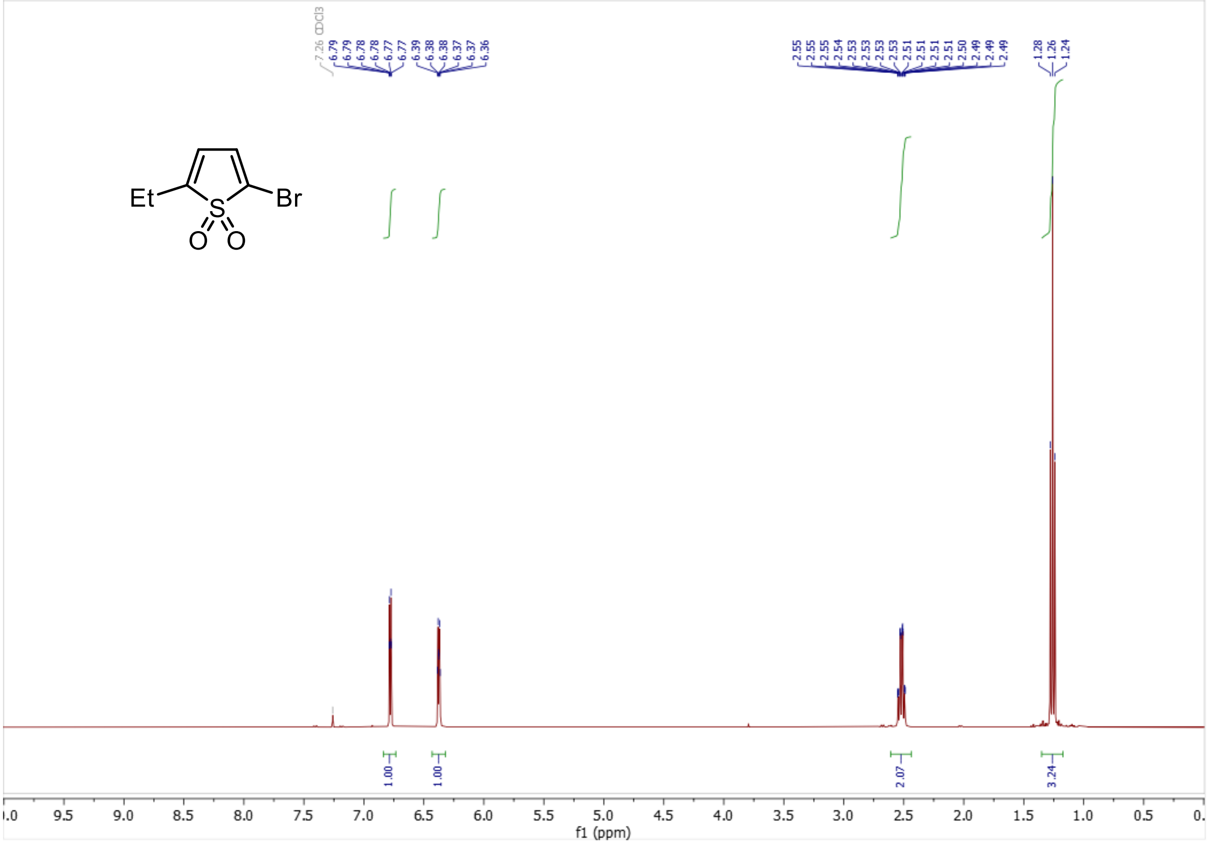

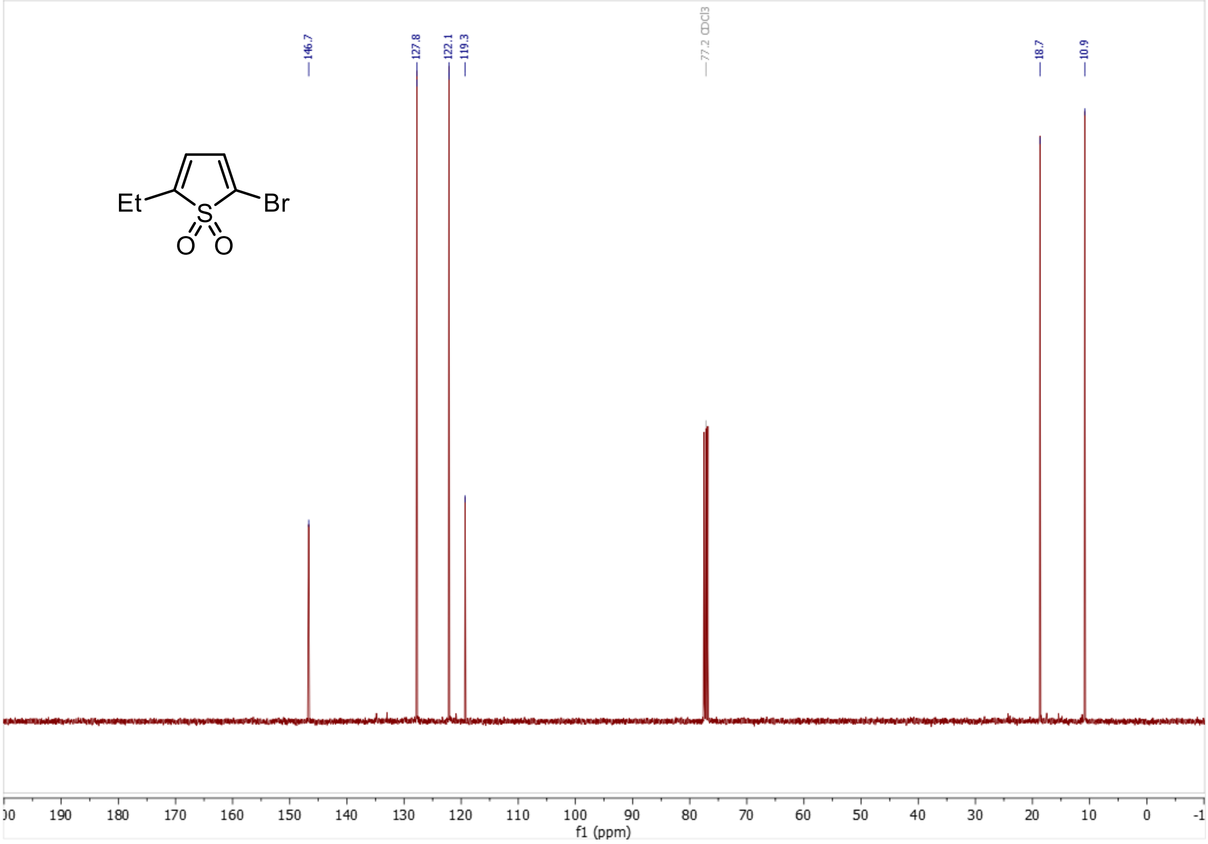

Compound 33a:

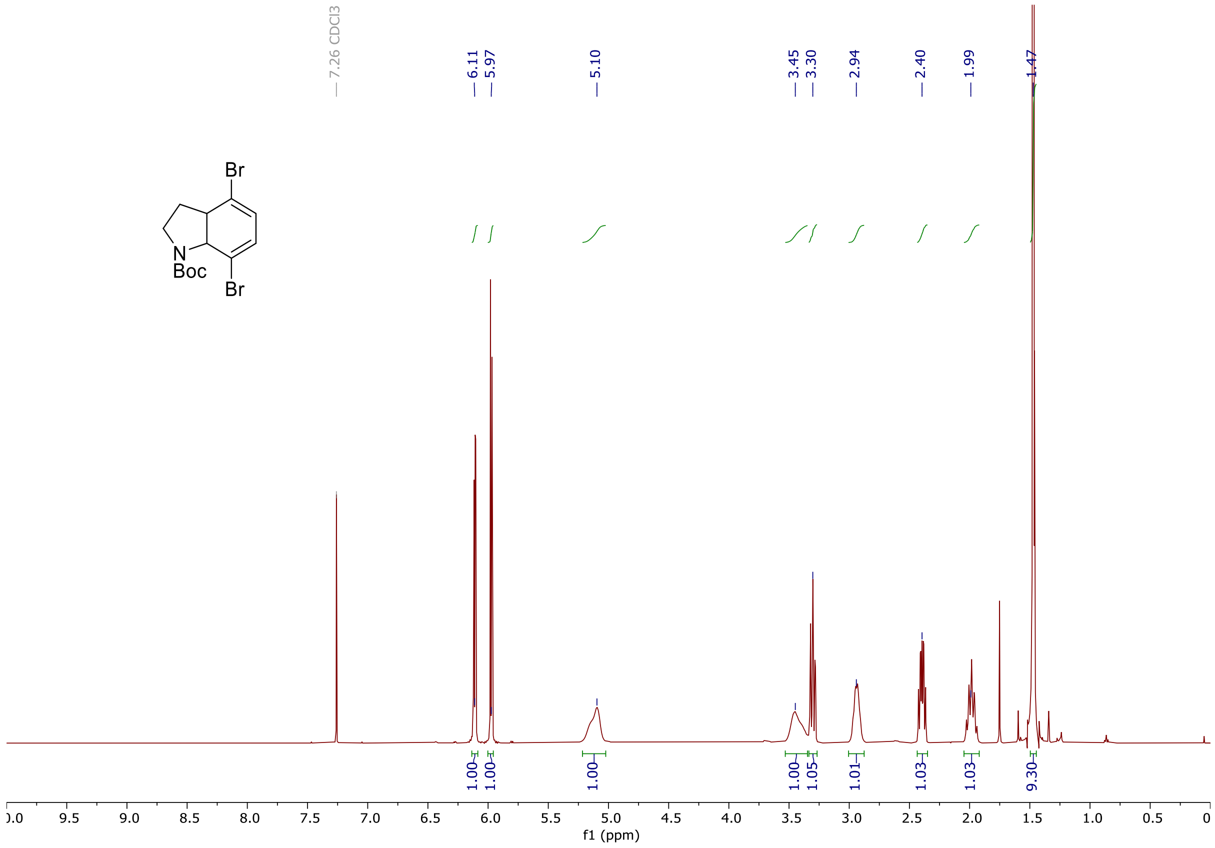

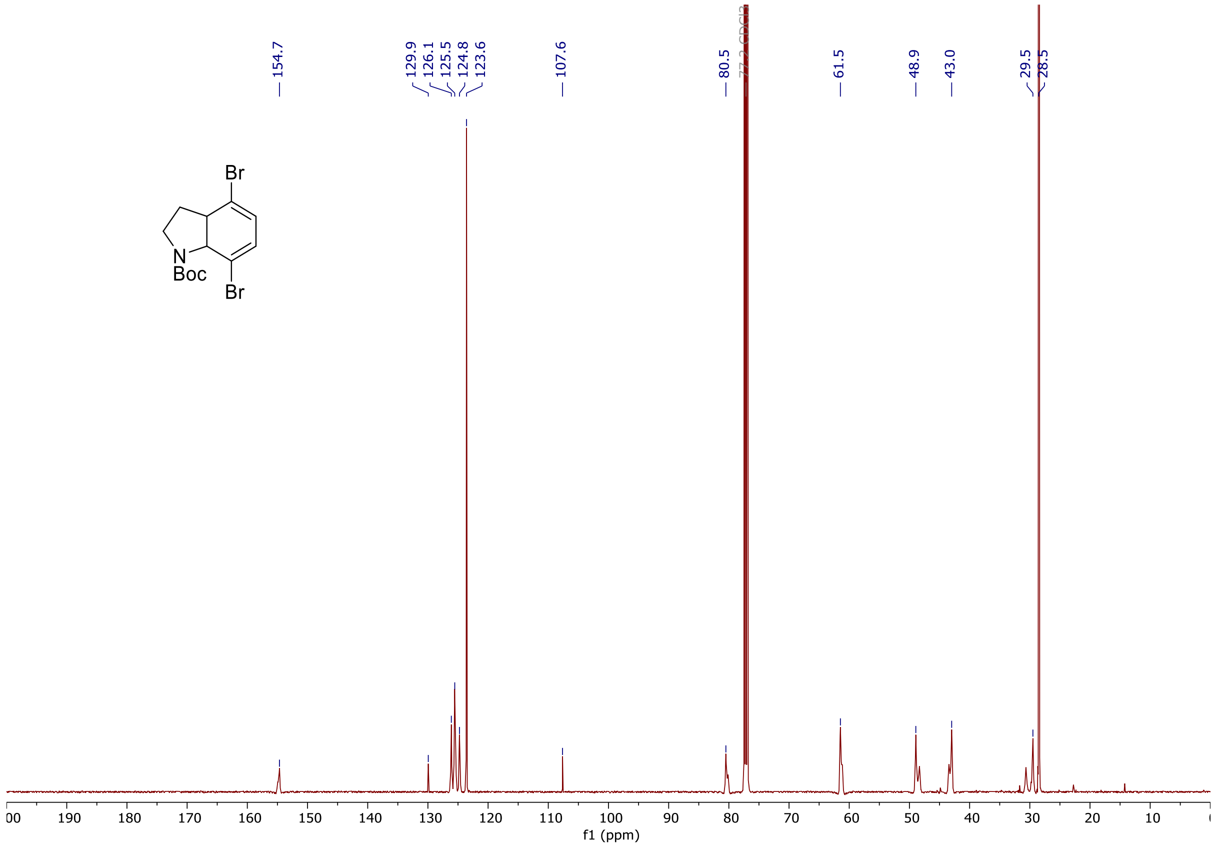

Compound 33b:

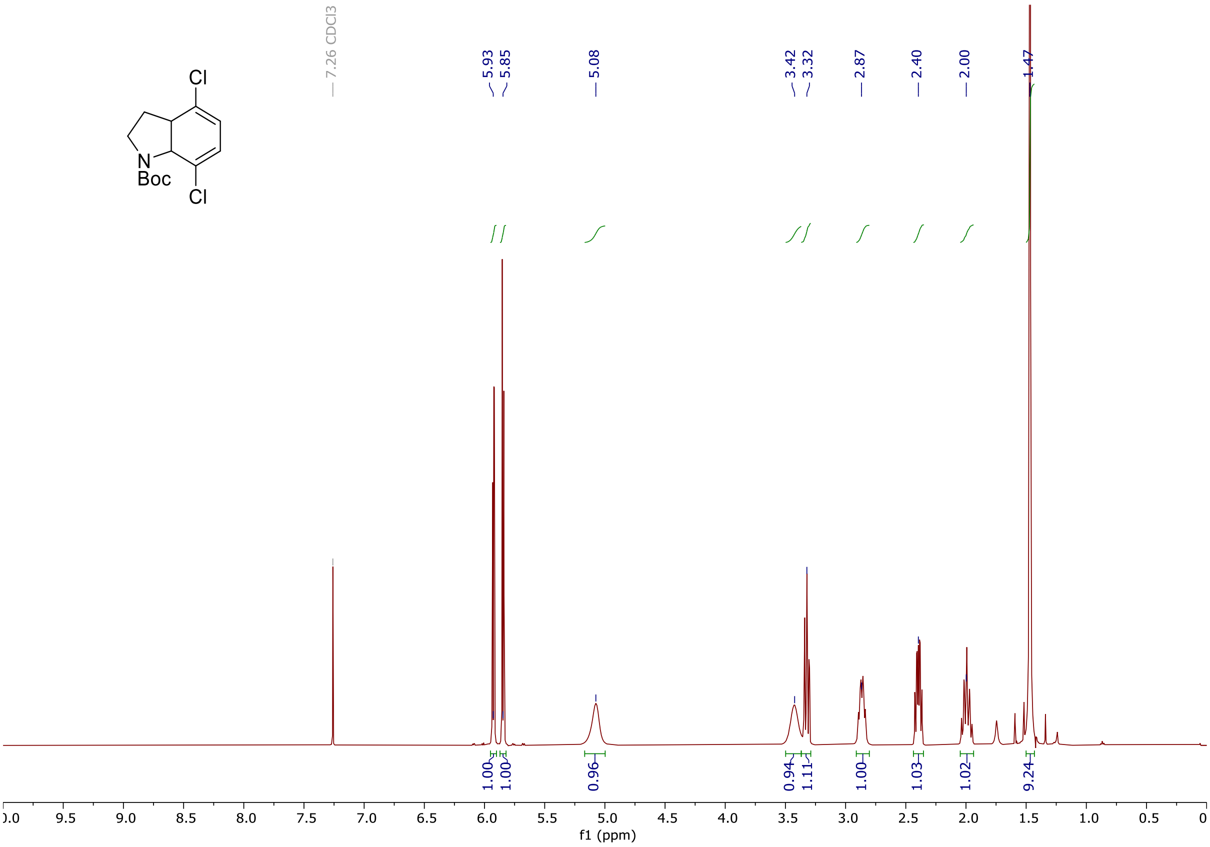

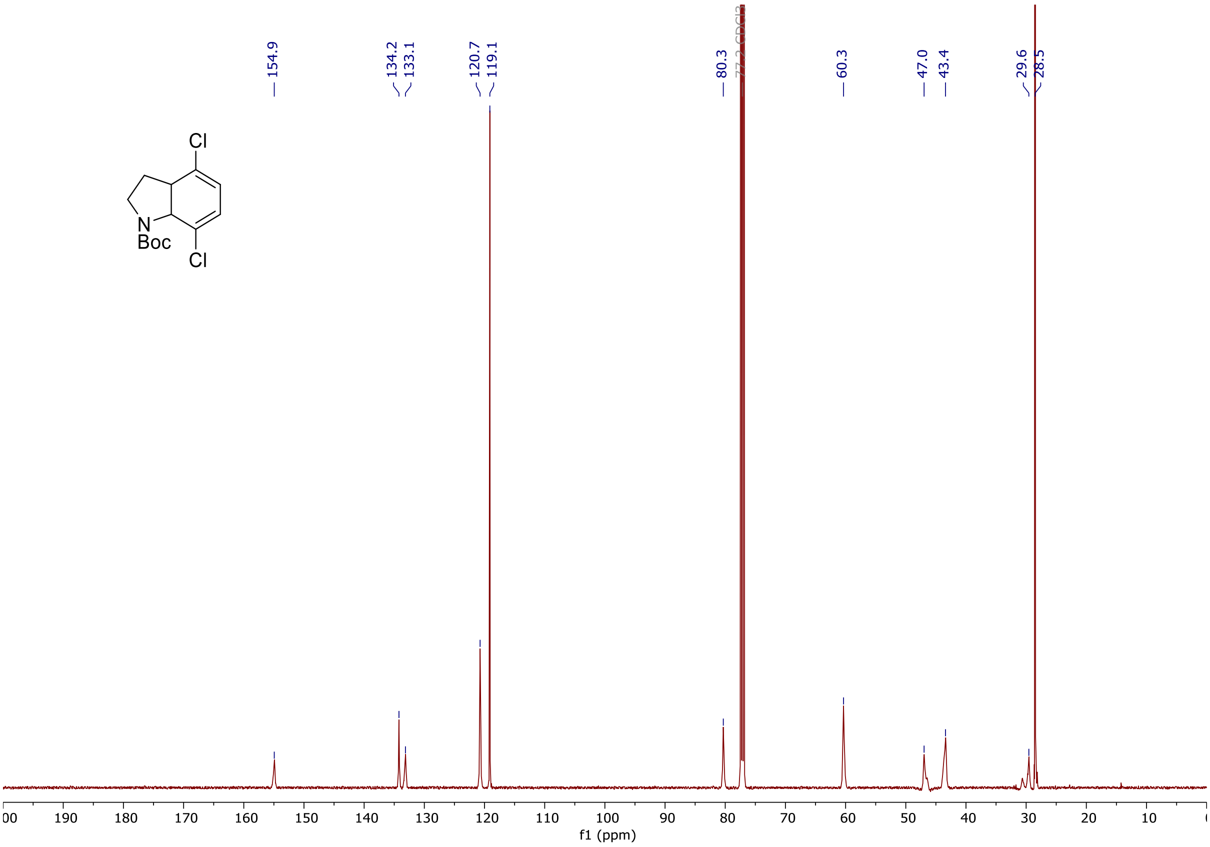

Compound 36a:

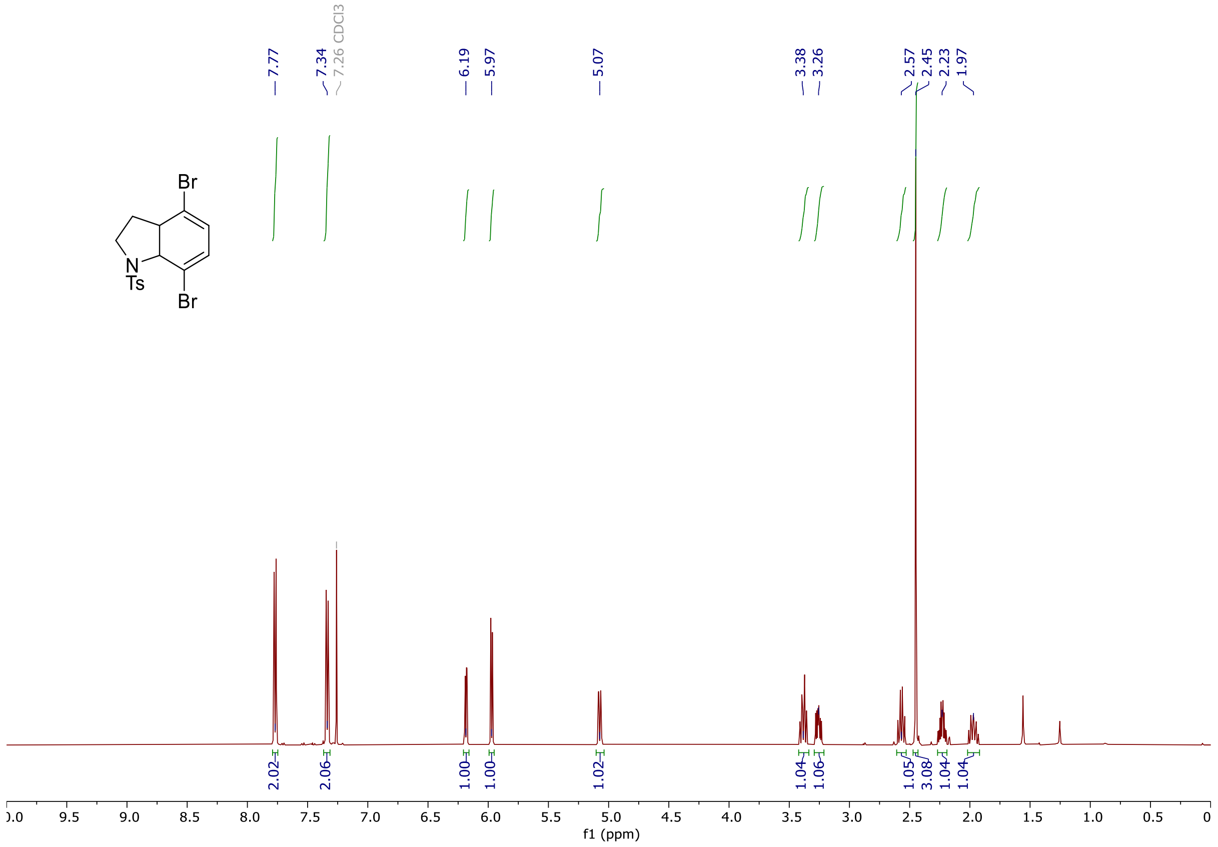

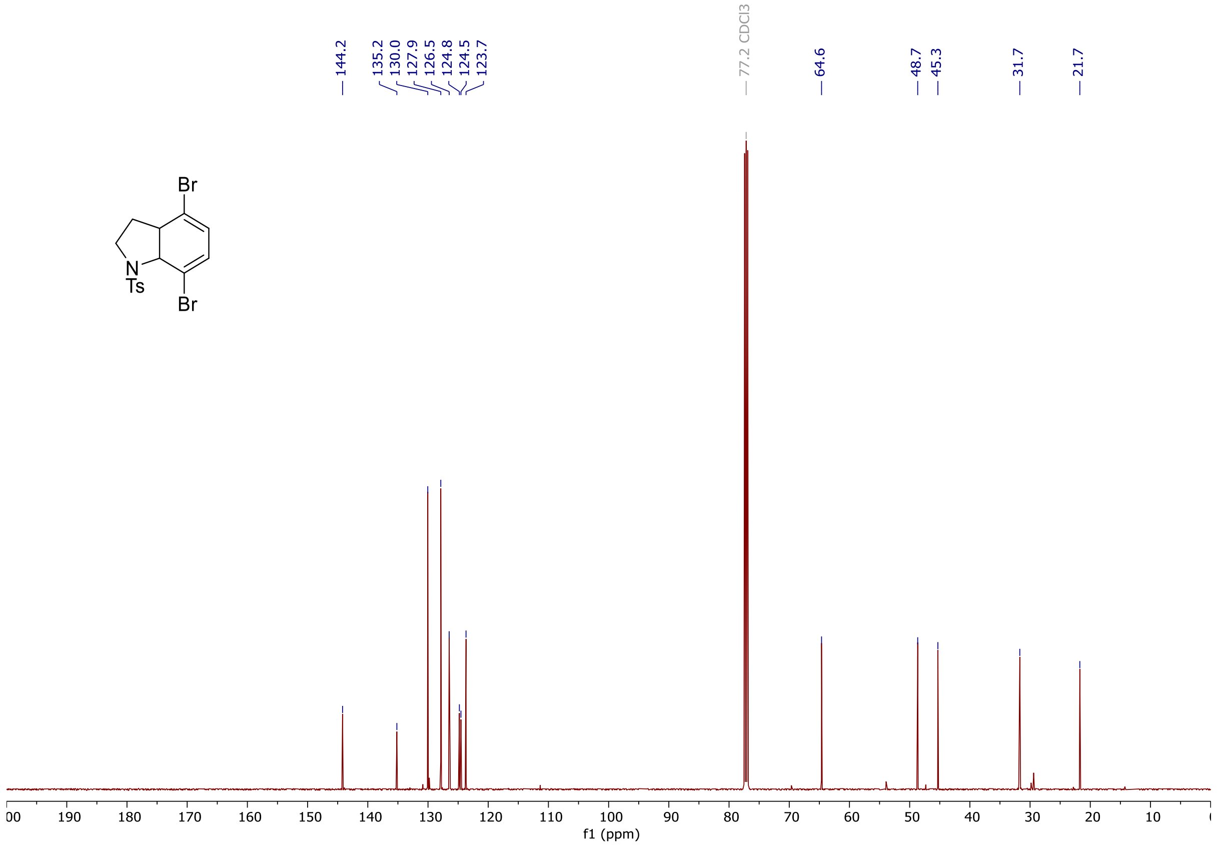

Compound 36b:

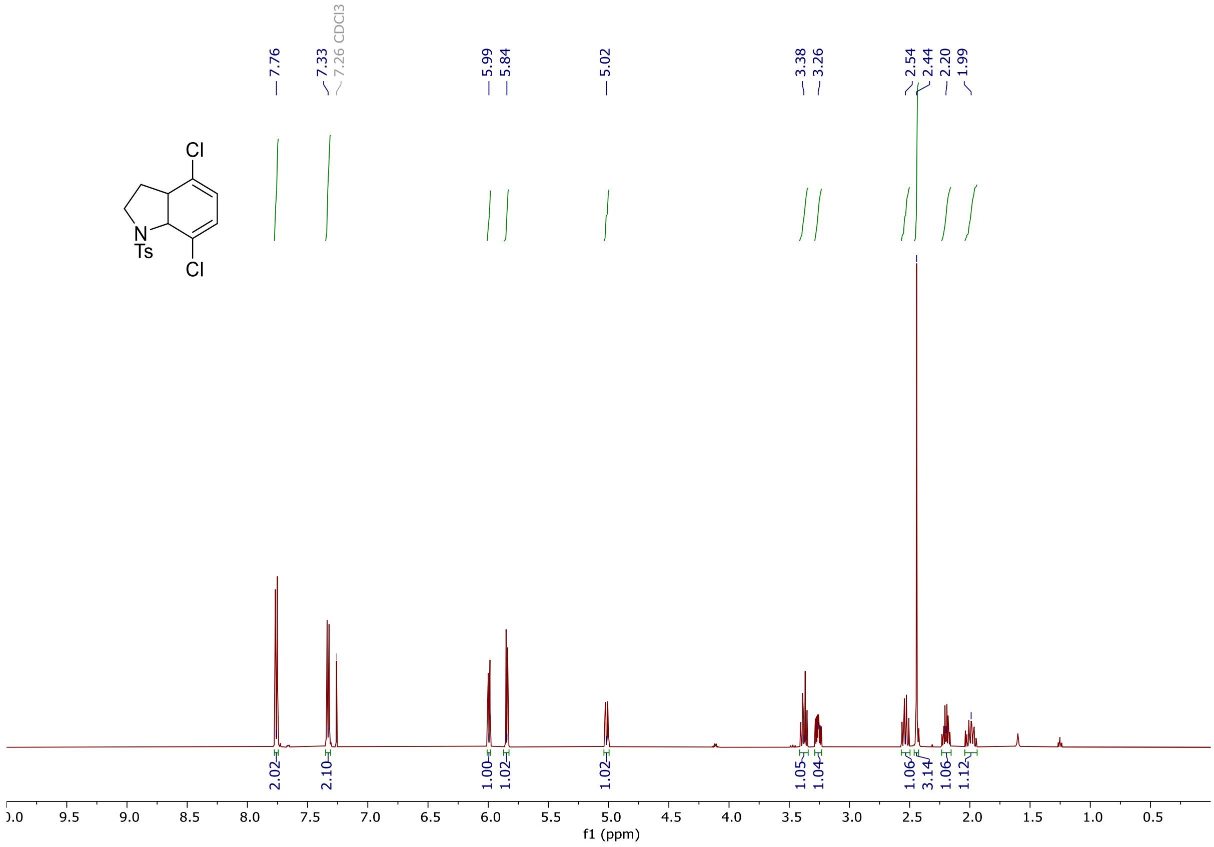

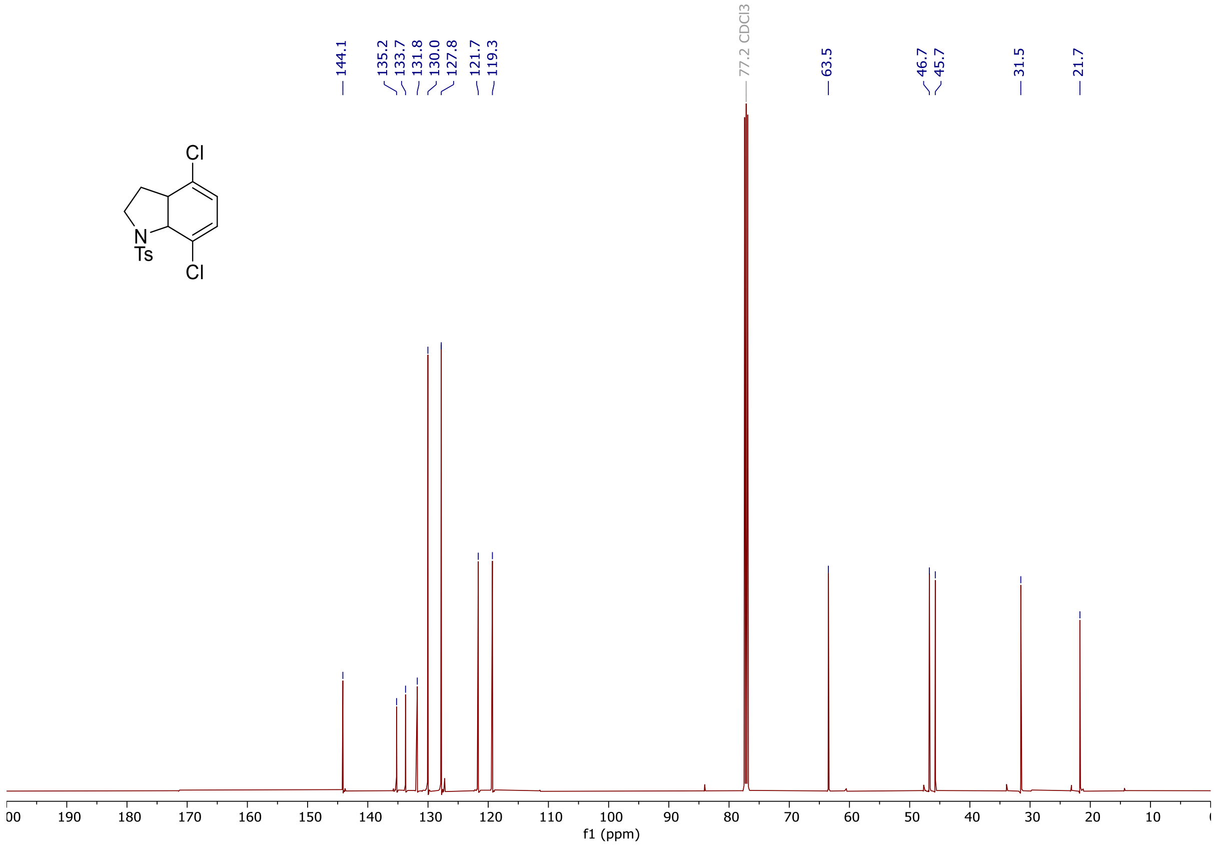

Compound 34a:

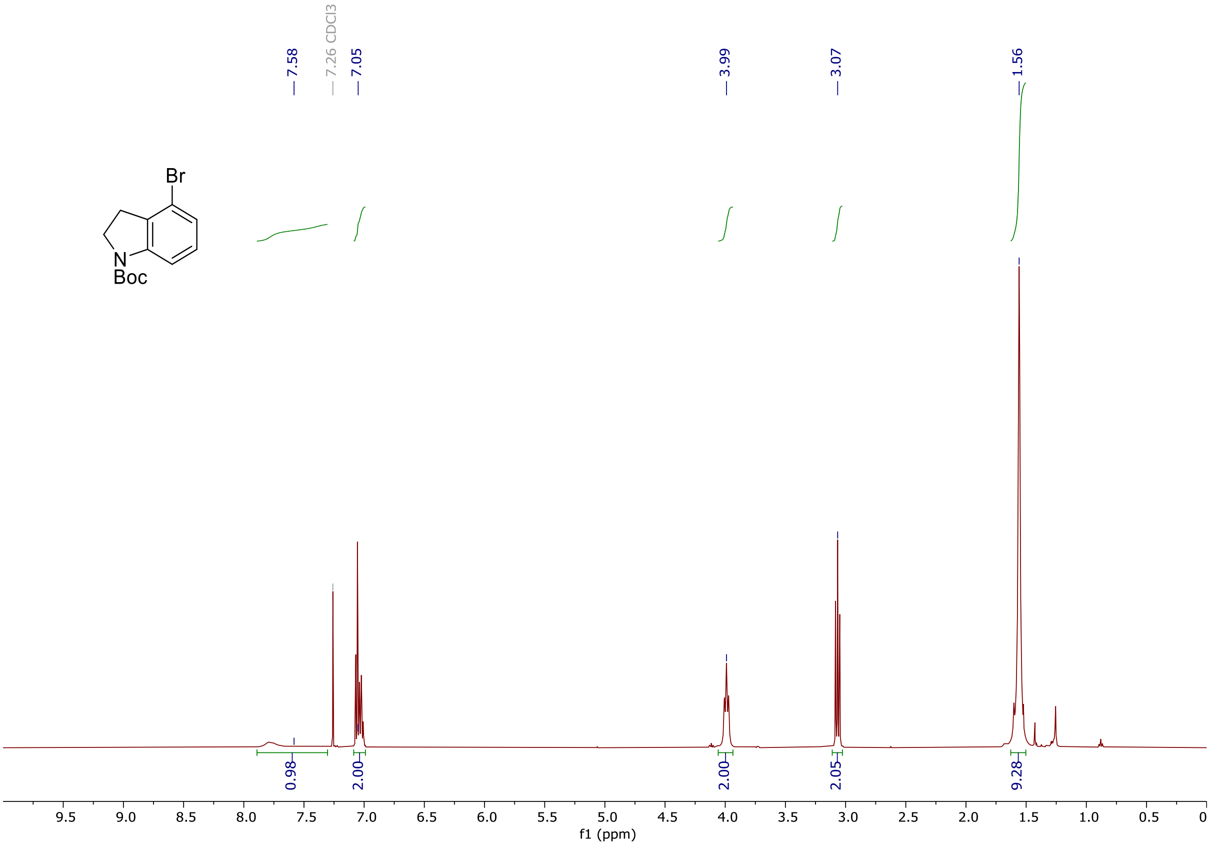

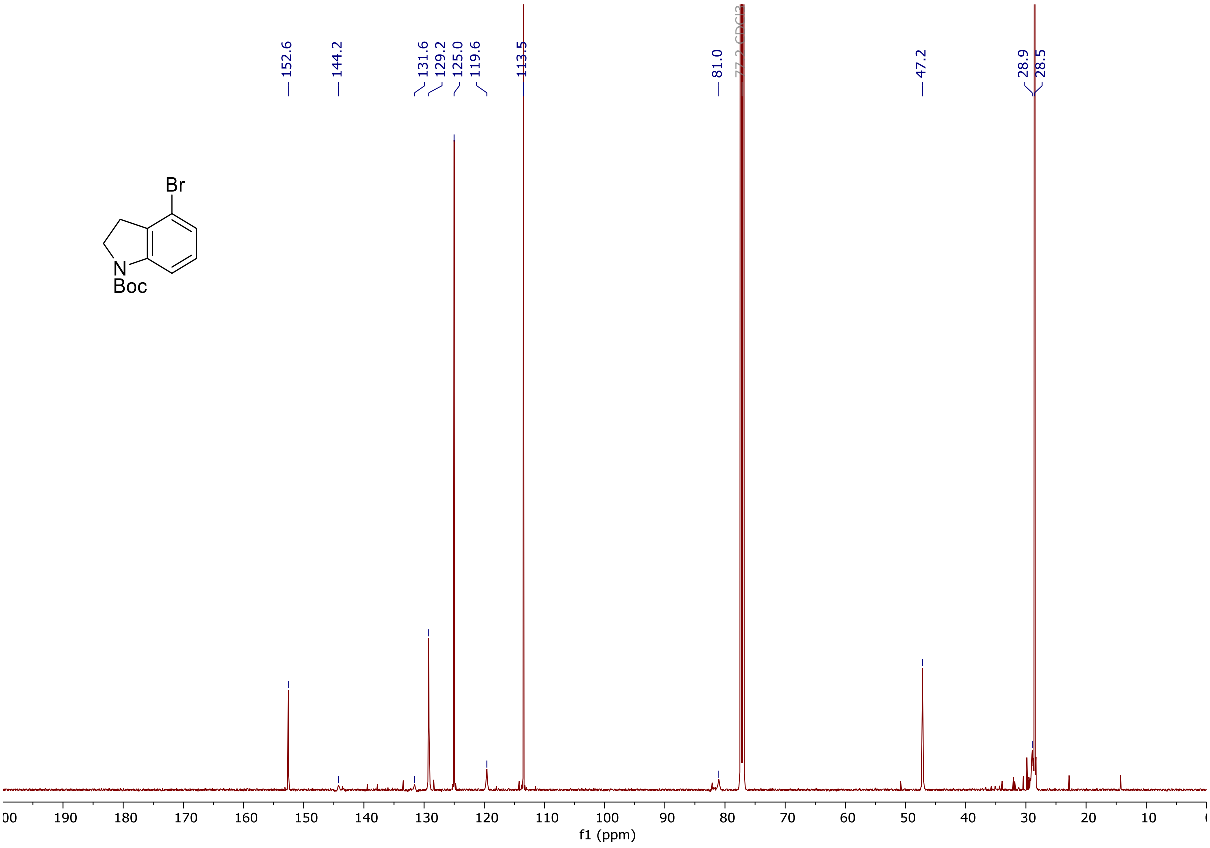

Compound 34b:

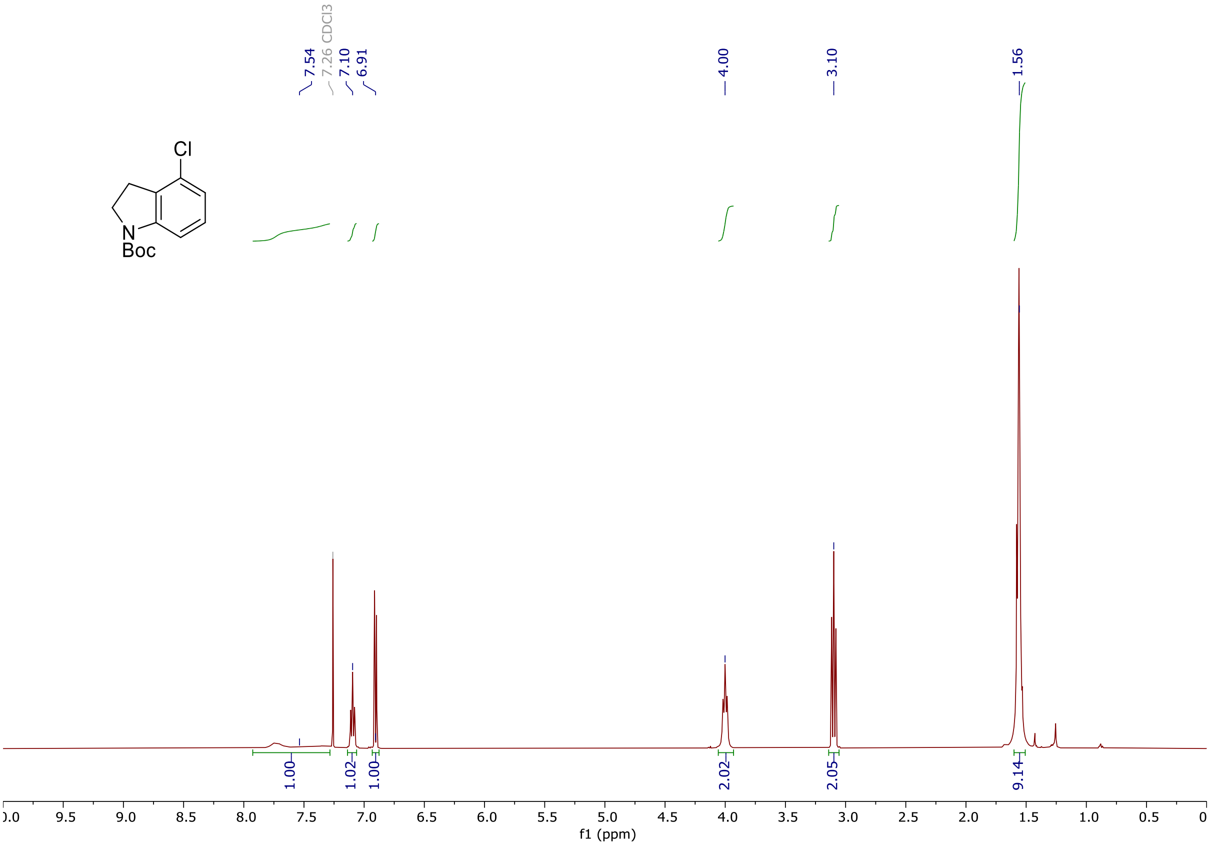

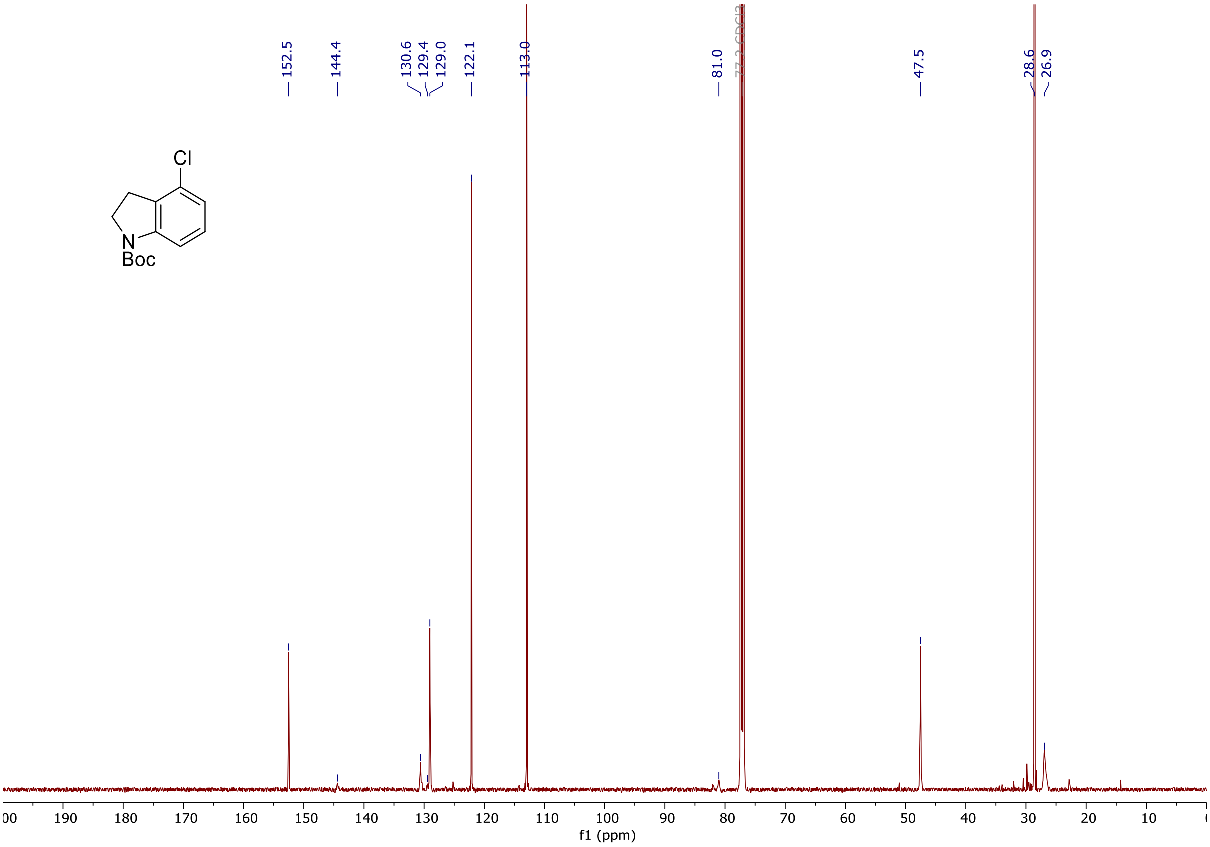

Compound 37a:

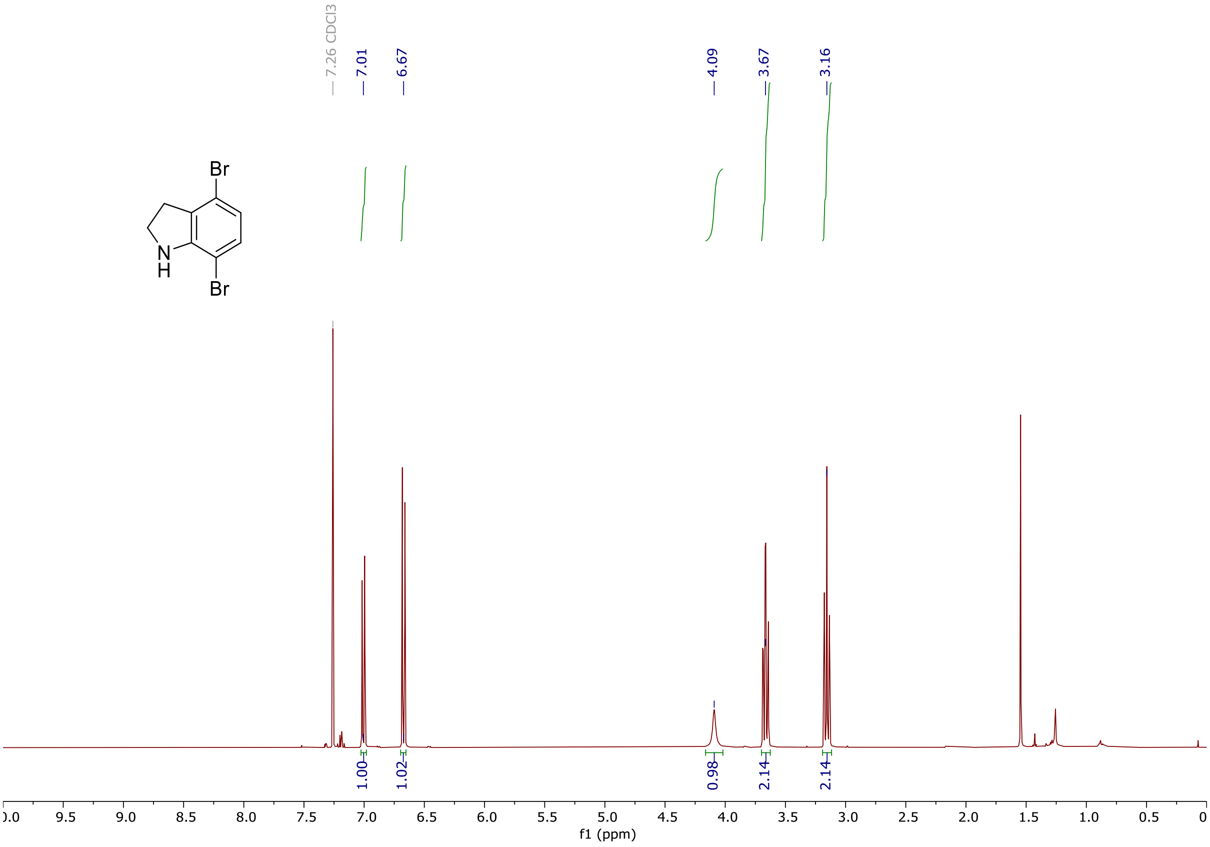

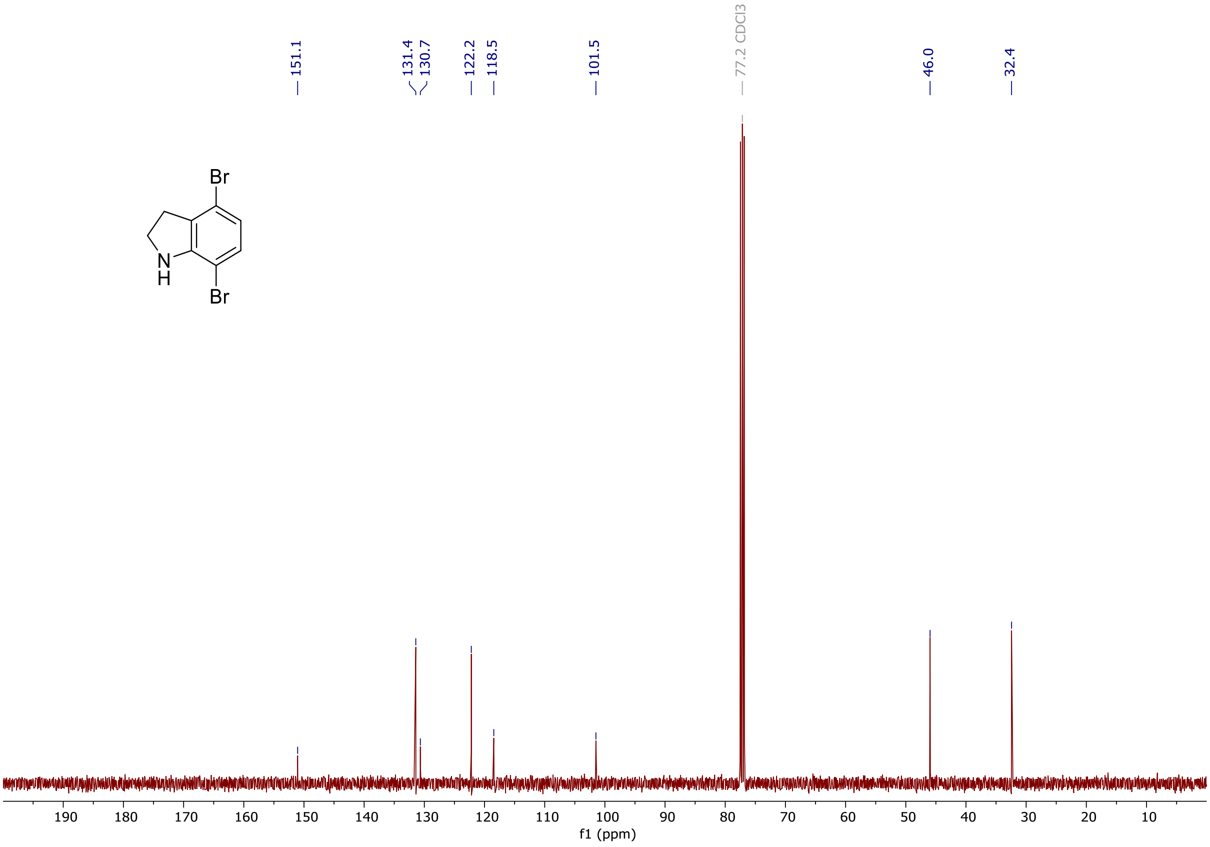

Compound 37b:

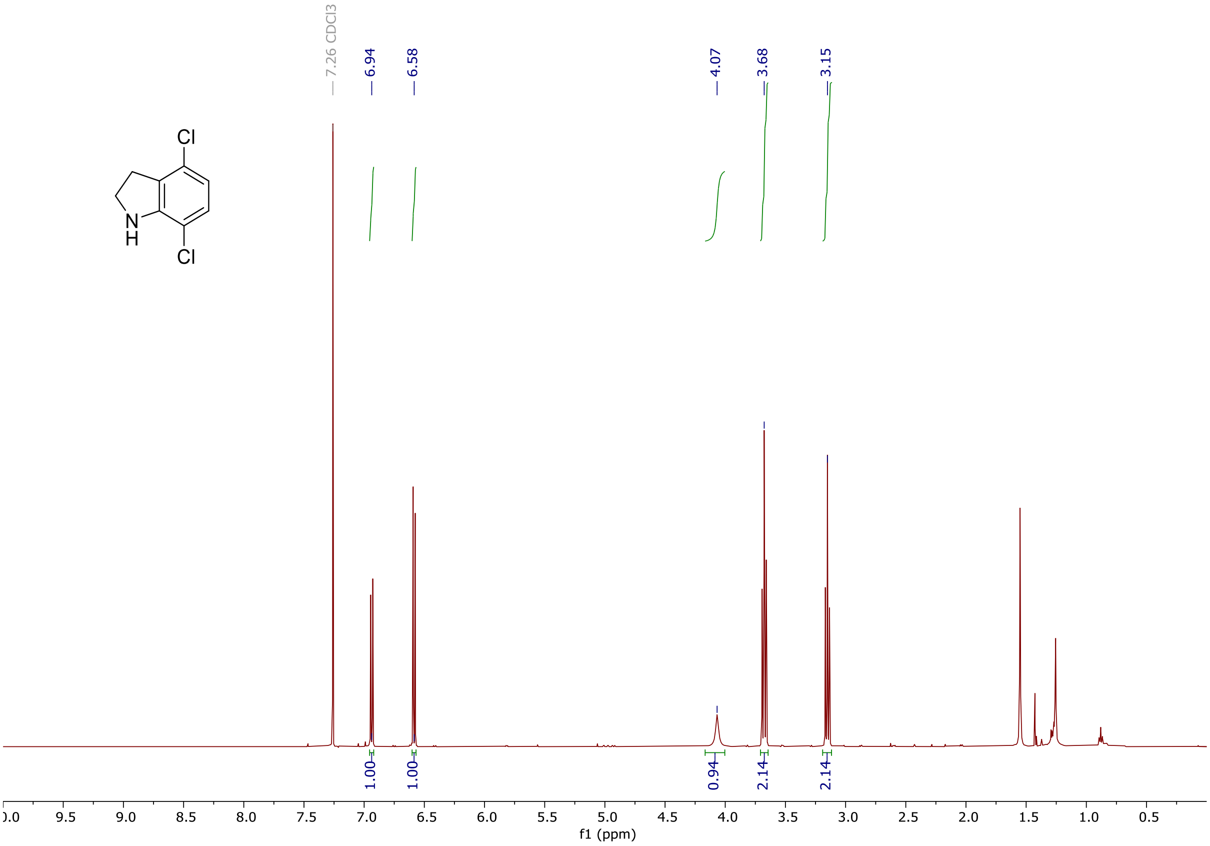

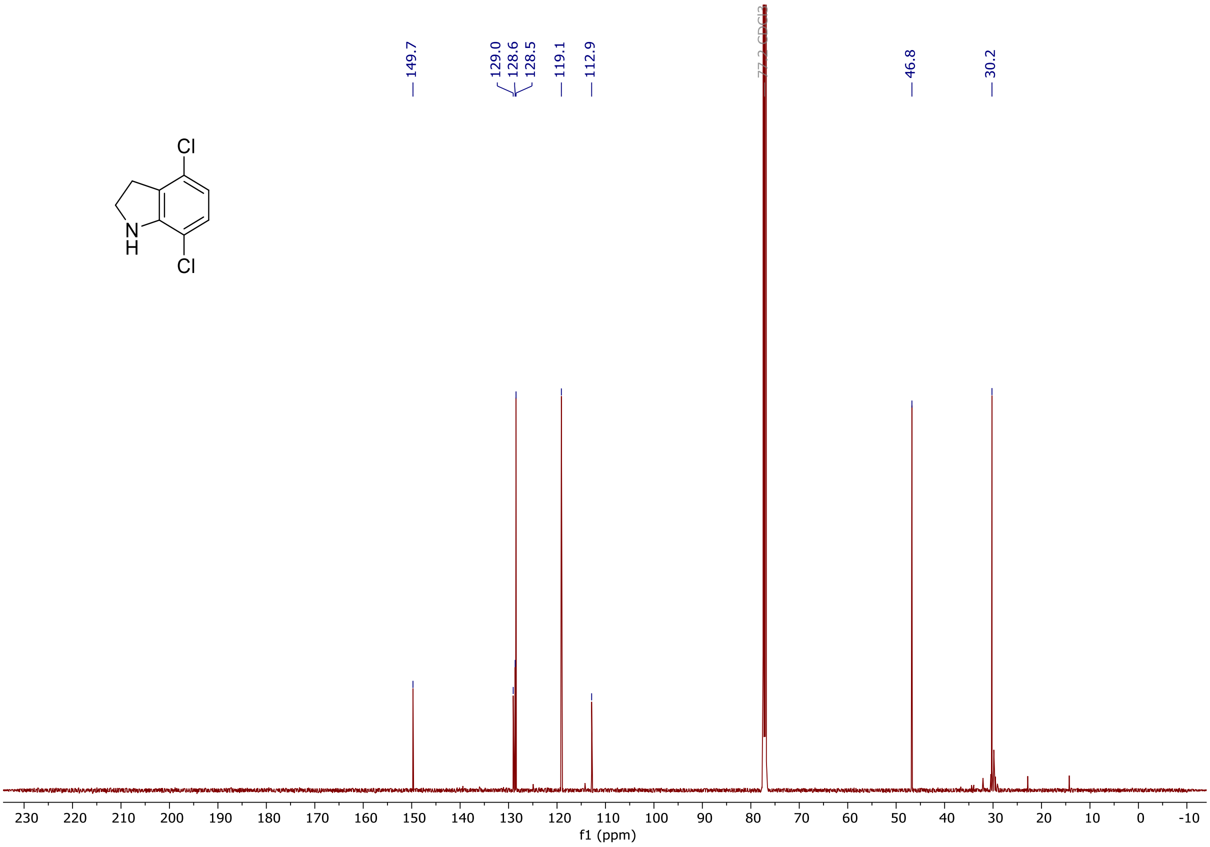

Compound 38a:

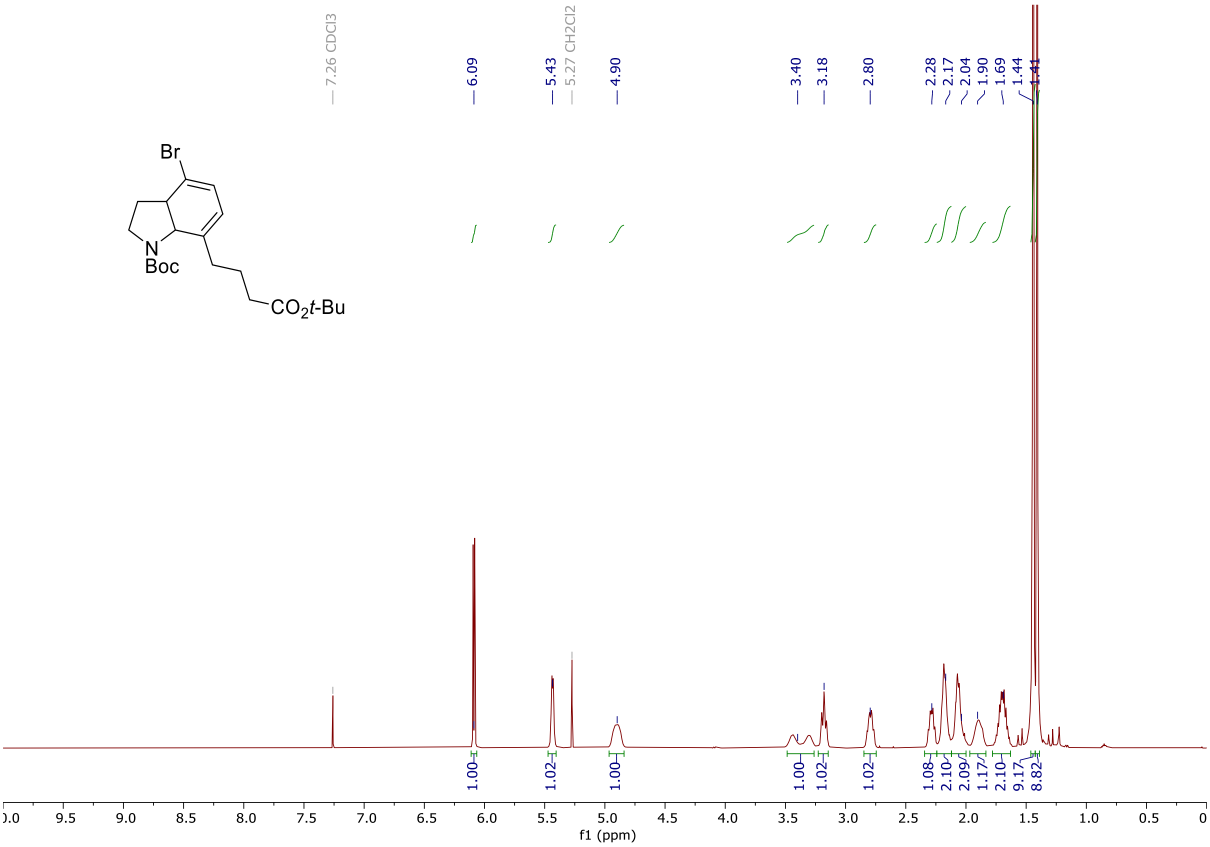

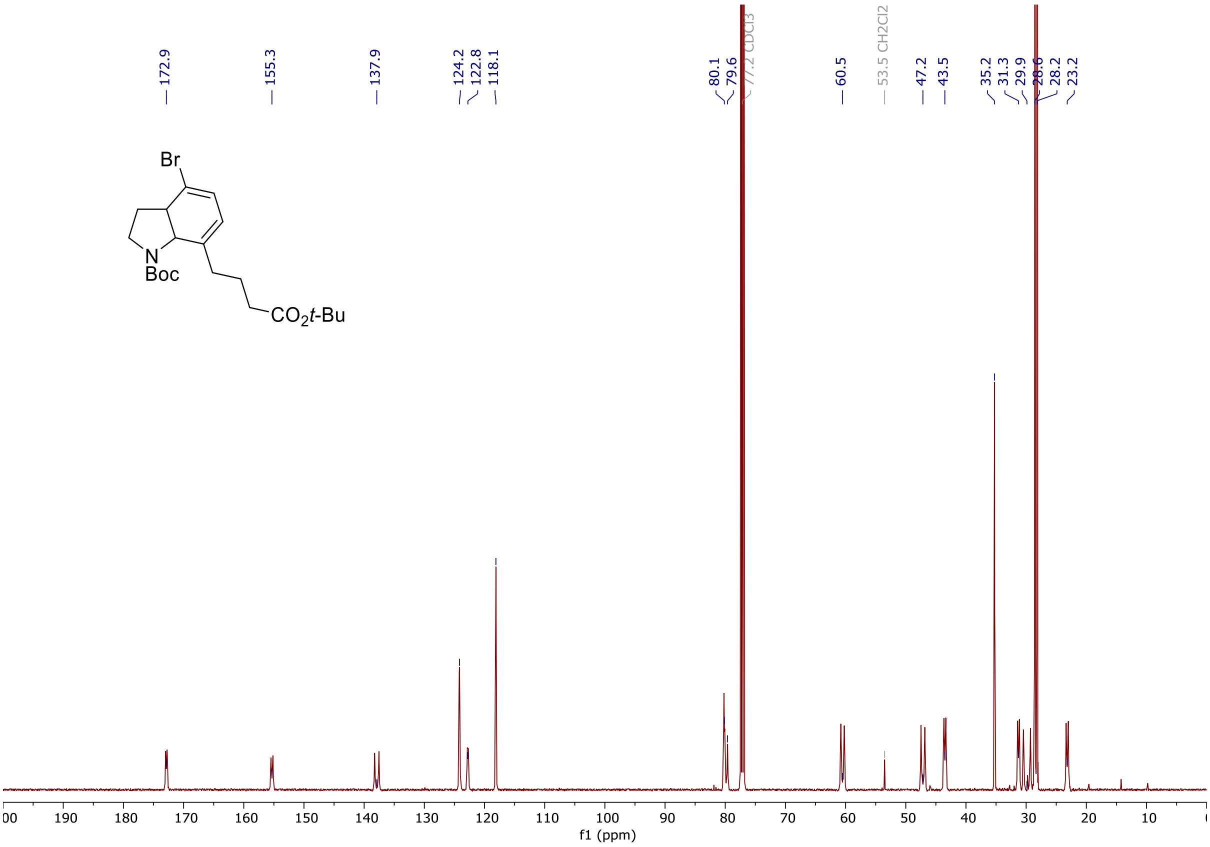

HMBC: Single regioisomer HMBC correlations

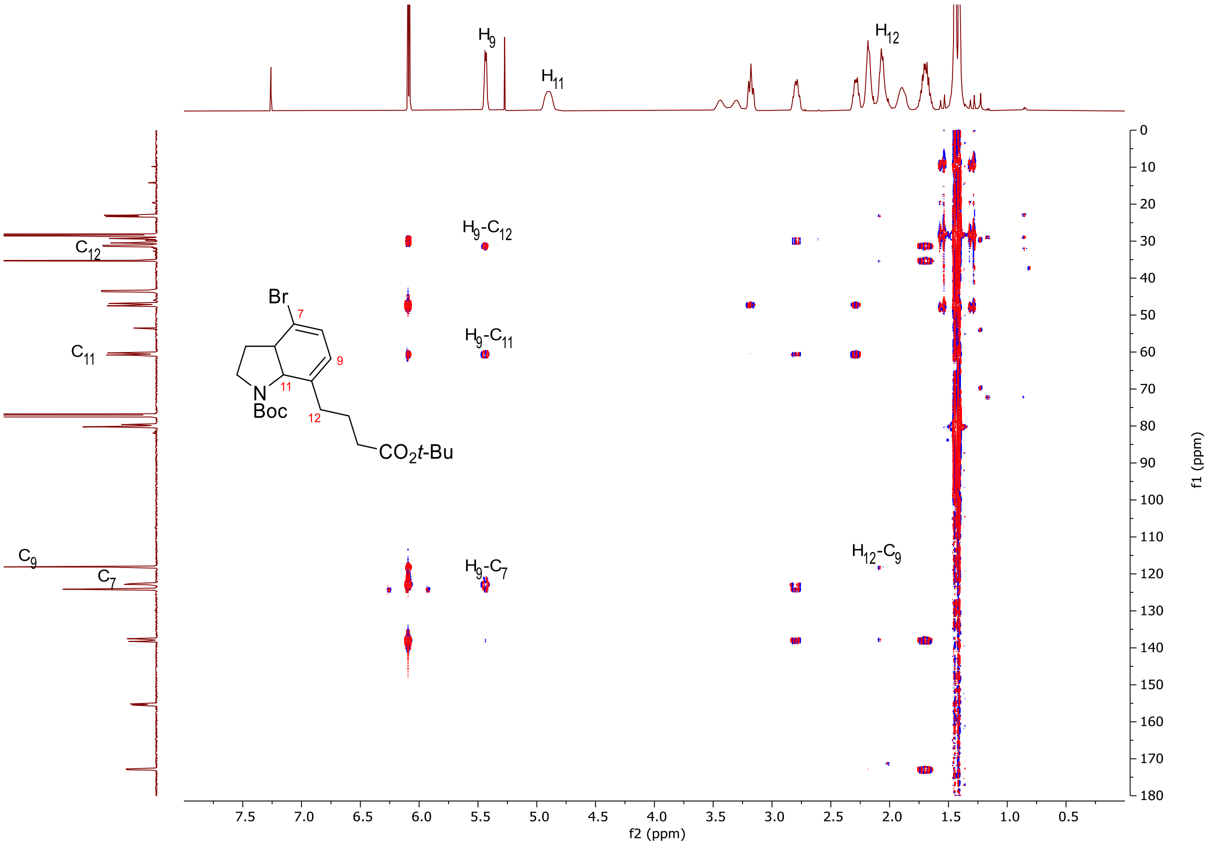

Compound 38b:

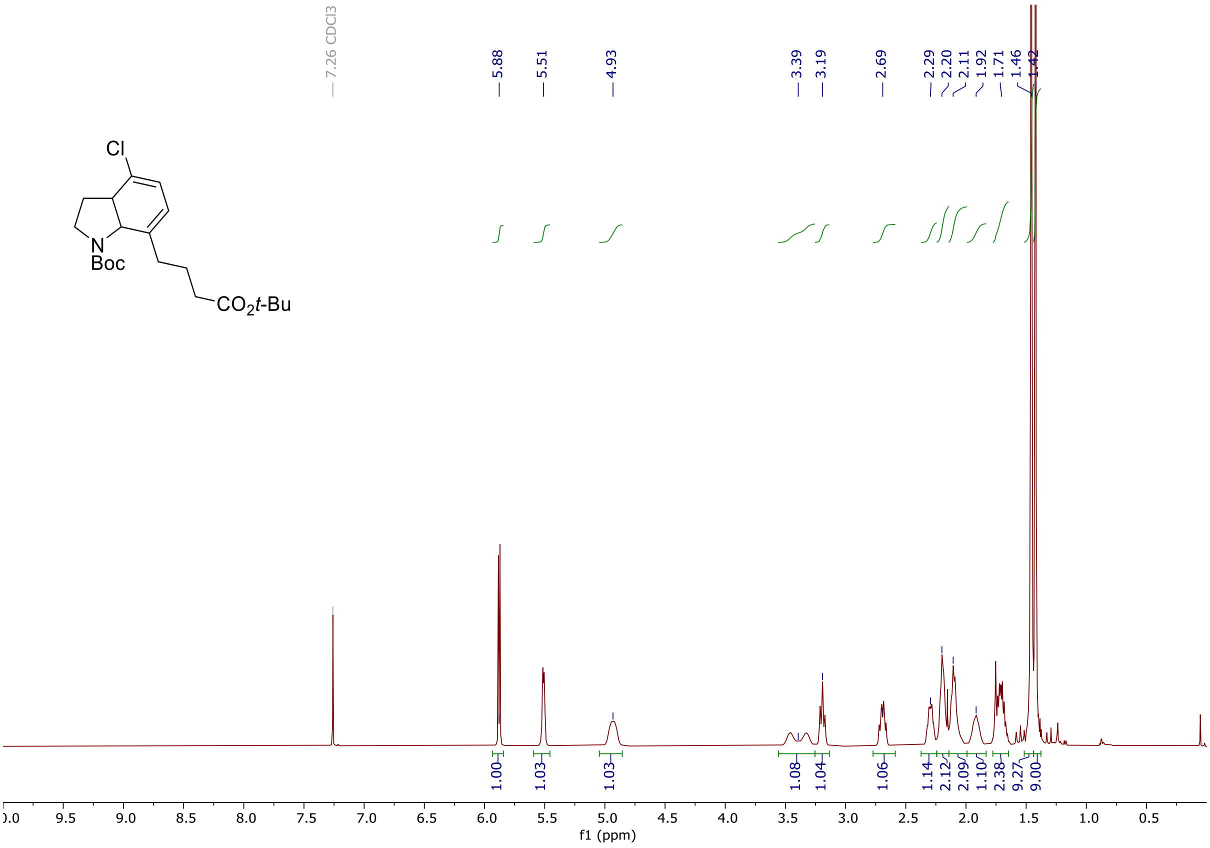

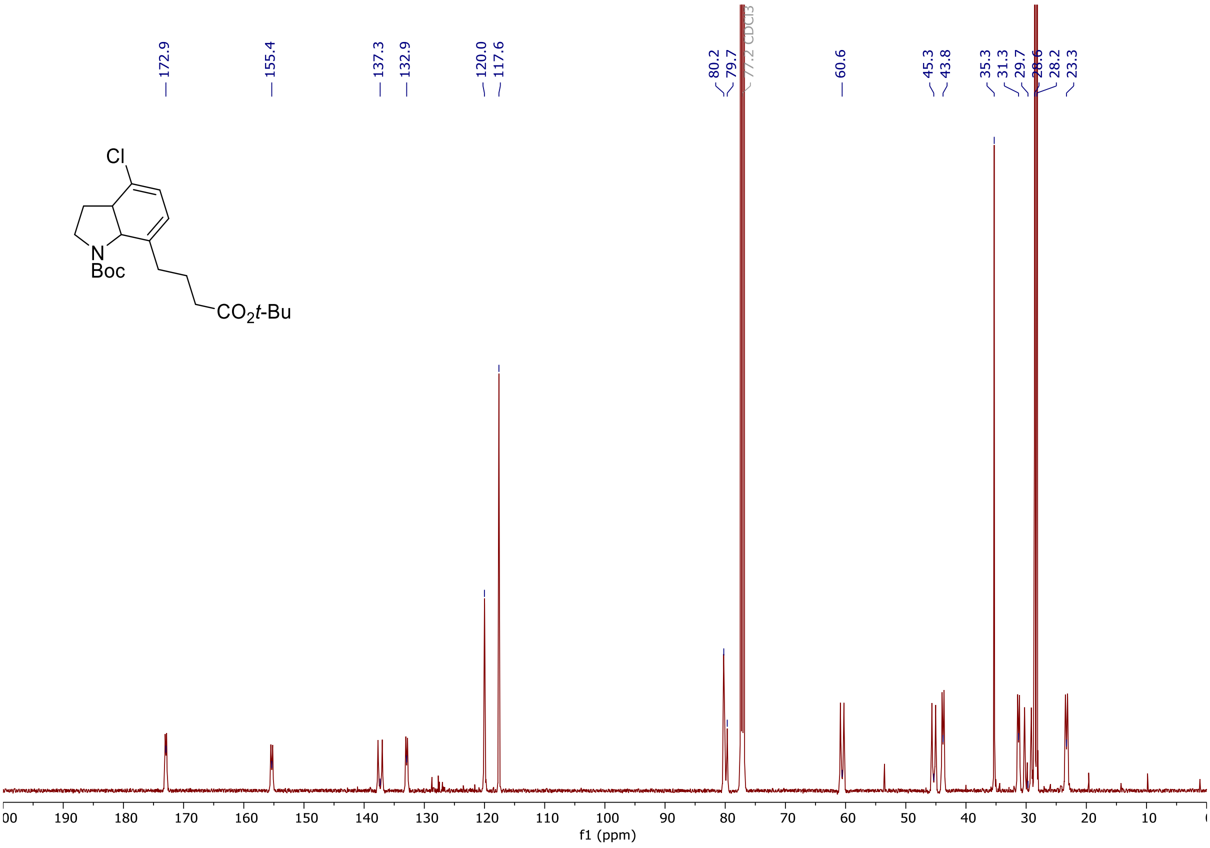

Compound 39:

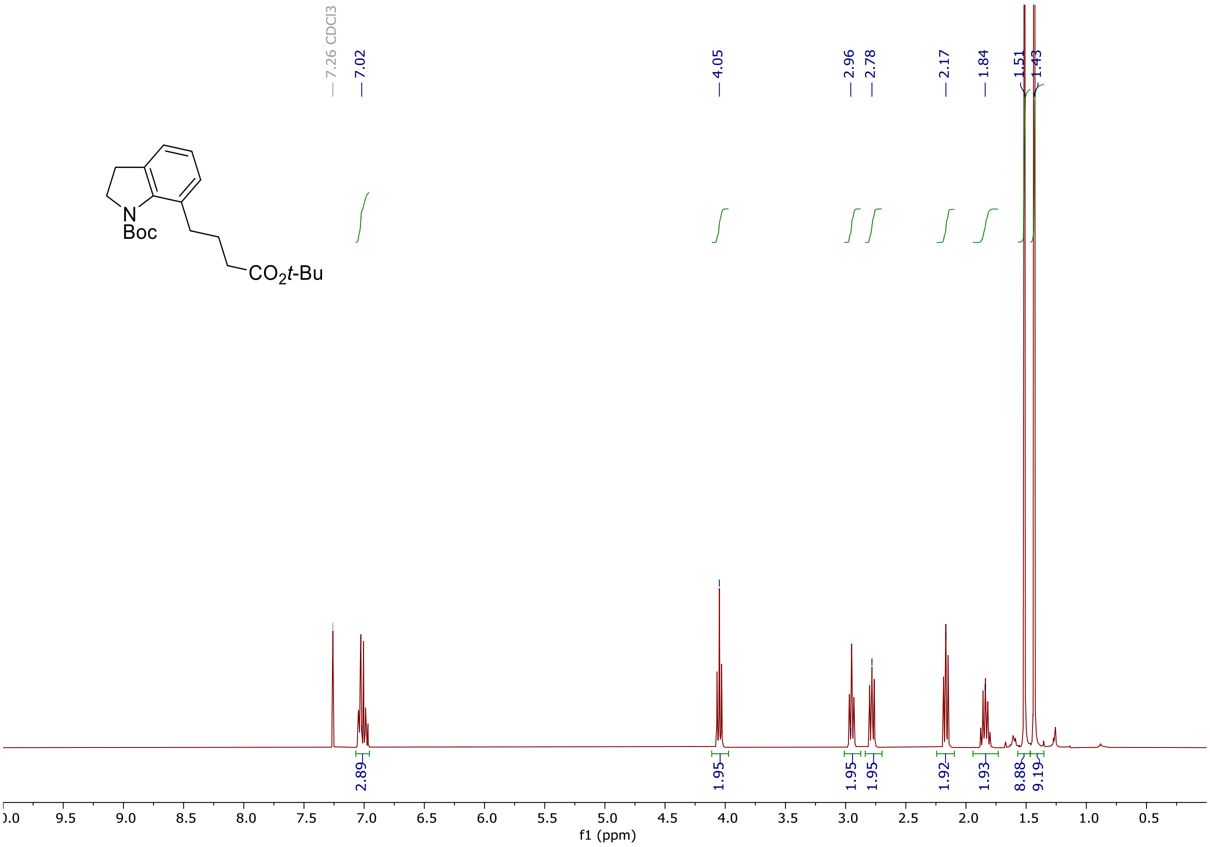
7.26 CDCl3
7.02
4.05
2.96
2.78
2.17
1.84
1.51
1.43
N
Boc
CO2t-Bu
2.89
1.95
1.95
1.95
1.92
1.93
8.88
9.19
f1 (ppm)

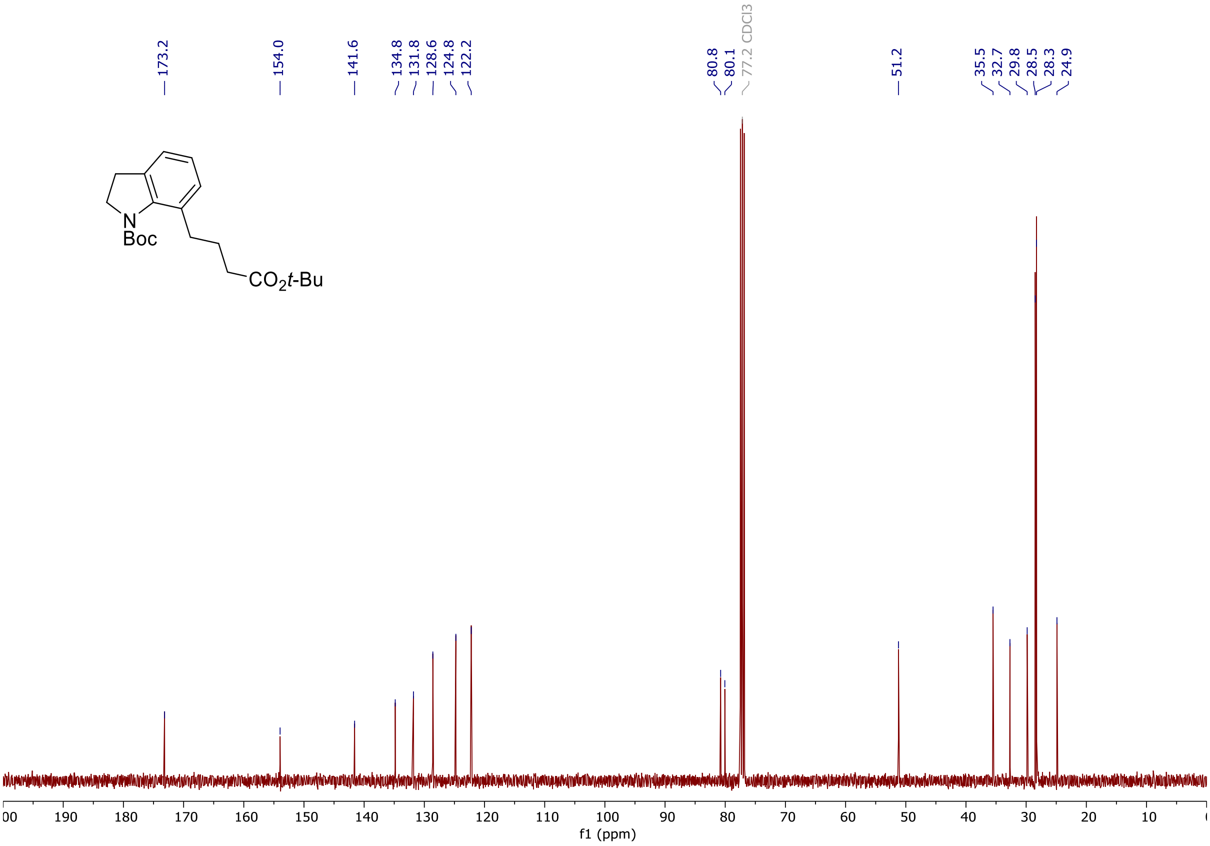
173.2
154.0
141.6
134.8
131.8
128.6
124.8
122.2
80.8
80.1
77.2 CDCl3
51.2
35.5
32.7
29.8
28.5
28.3
24.9
N
Boc
CO2t-Bu
f1 (ppm)

Compound 40:

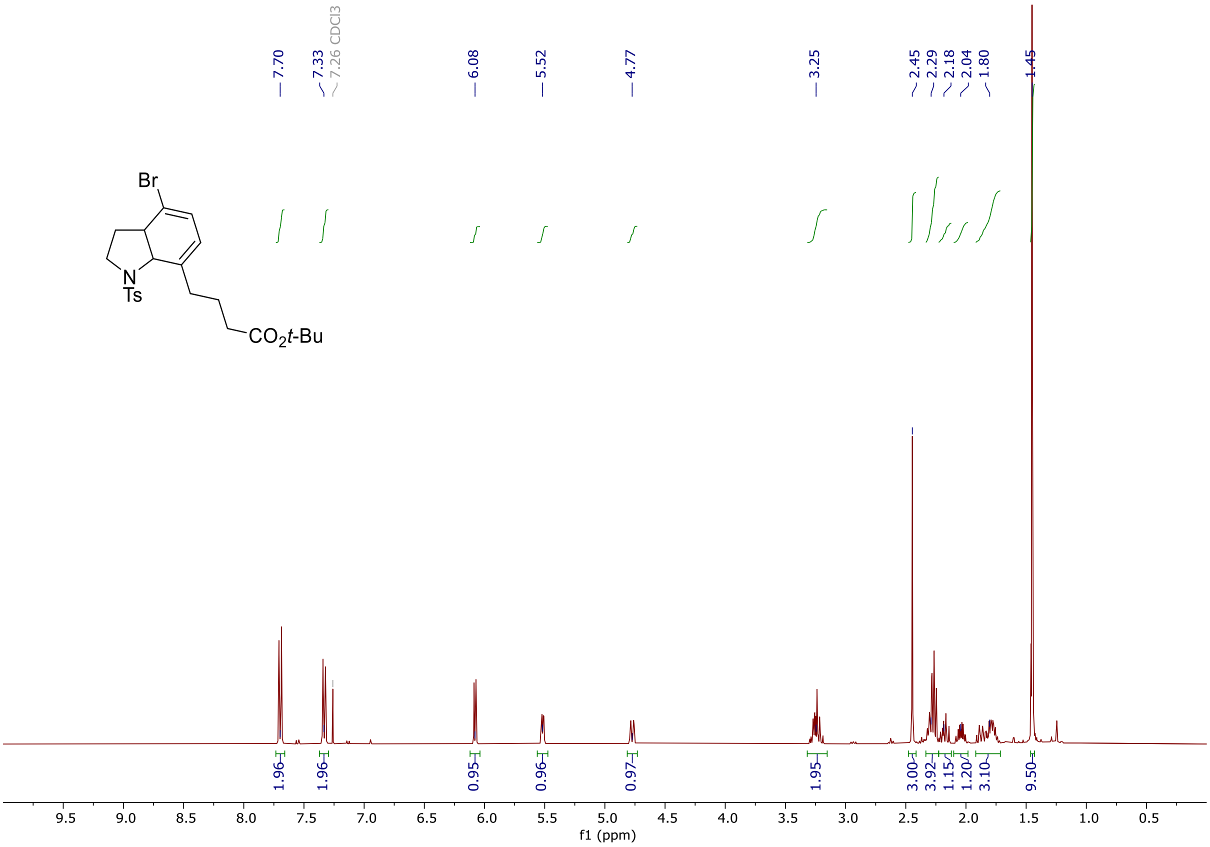

7.70
7.33
7.26 CDCl3
6.08
5.52
4.77
3.25
2.45
2.29
2.18
2.04
1.80
1.45
Br
N
Ts
CO_2t-Bu
1.96
1.96
0.95
0.96
0.97
1.95
3.00
3.92
1.15
1.20
3.10
9.50
9.5
9.0
8.5
8.0
7.5
7.0
6.5
6.0
5.5
5.0
4.5
4.0
3.5
3.0
2.5
2.0
1.5
1.0
0.5
f1 (ppm)

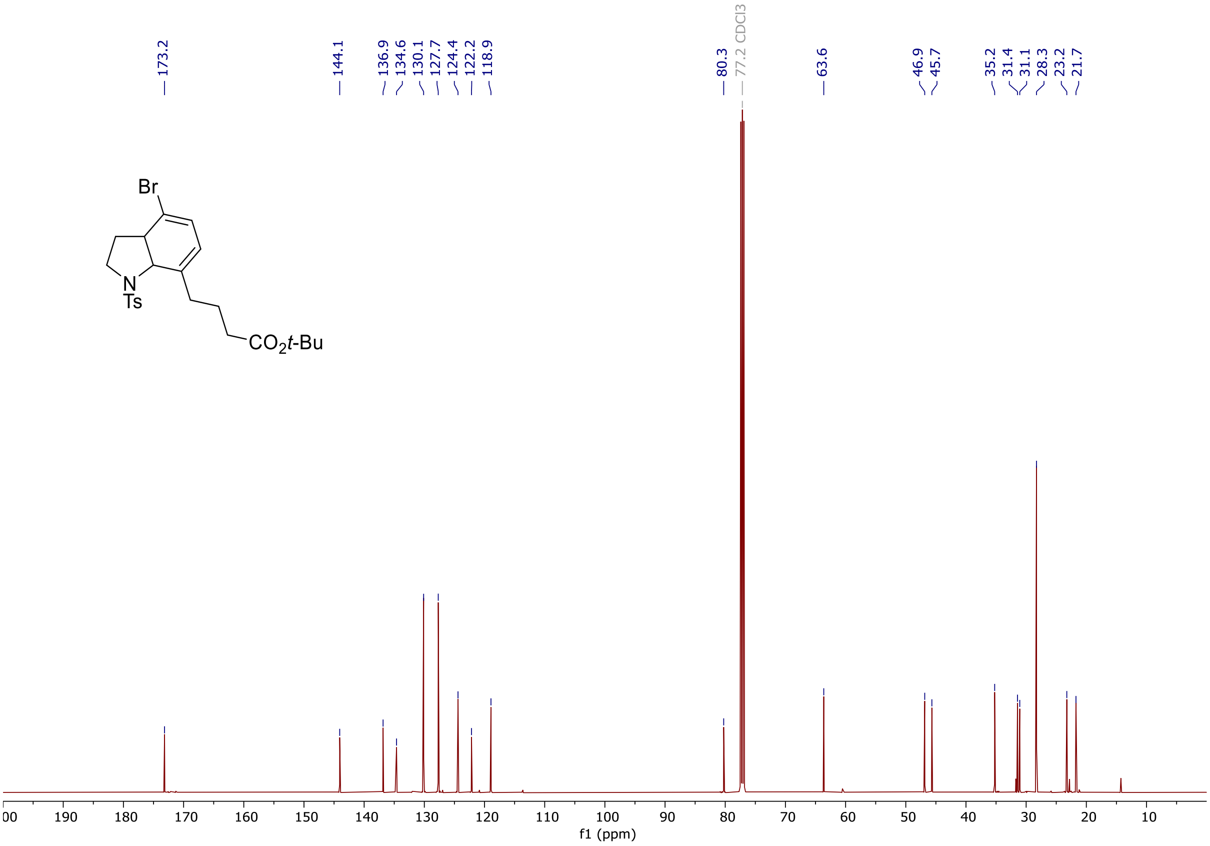

173.2
144.1
136.9
134.6
130.1
127.7
124.4
122.2
118.9
80.3
77.2 CDCl3
63.6
46.9
45.7
35.2
31.4
31.1
28.3
23.2
21.7
Br
N
Ts
CO_2t-Bu
00
190
180
170
160
150
140
130
120
110
100
90
80
70
60
50
40
30
20
10
f1 (ppm)

Compound 41:

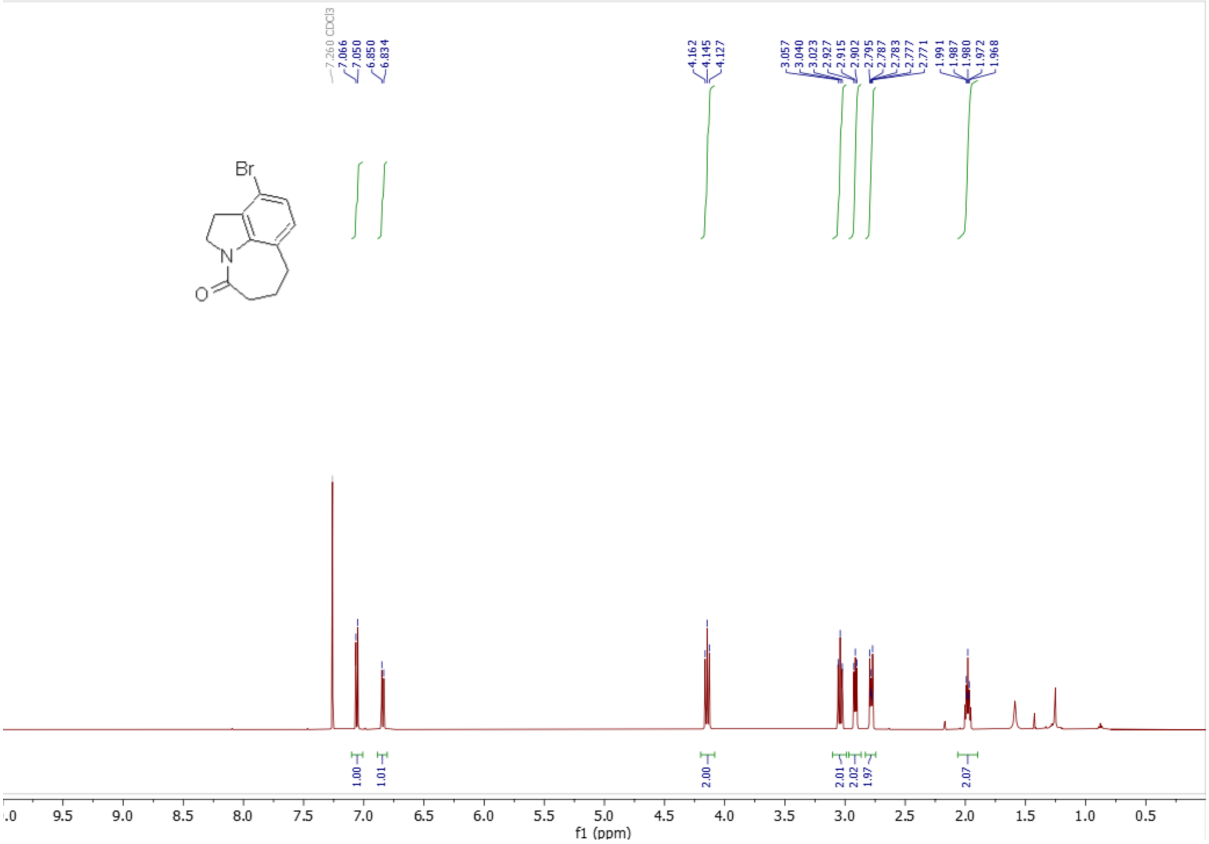

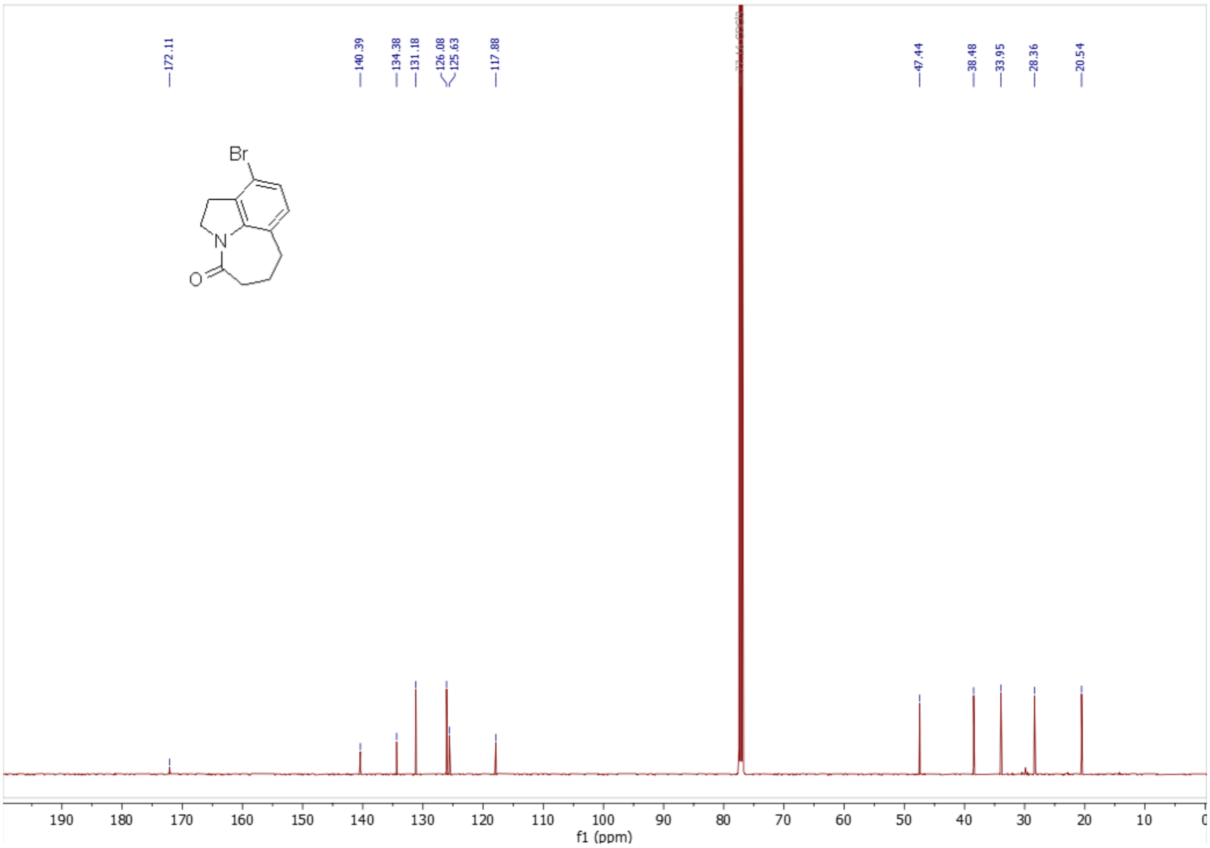

Compound 43a:

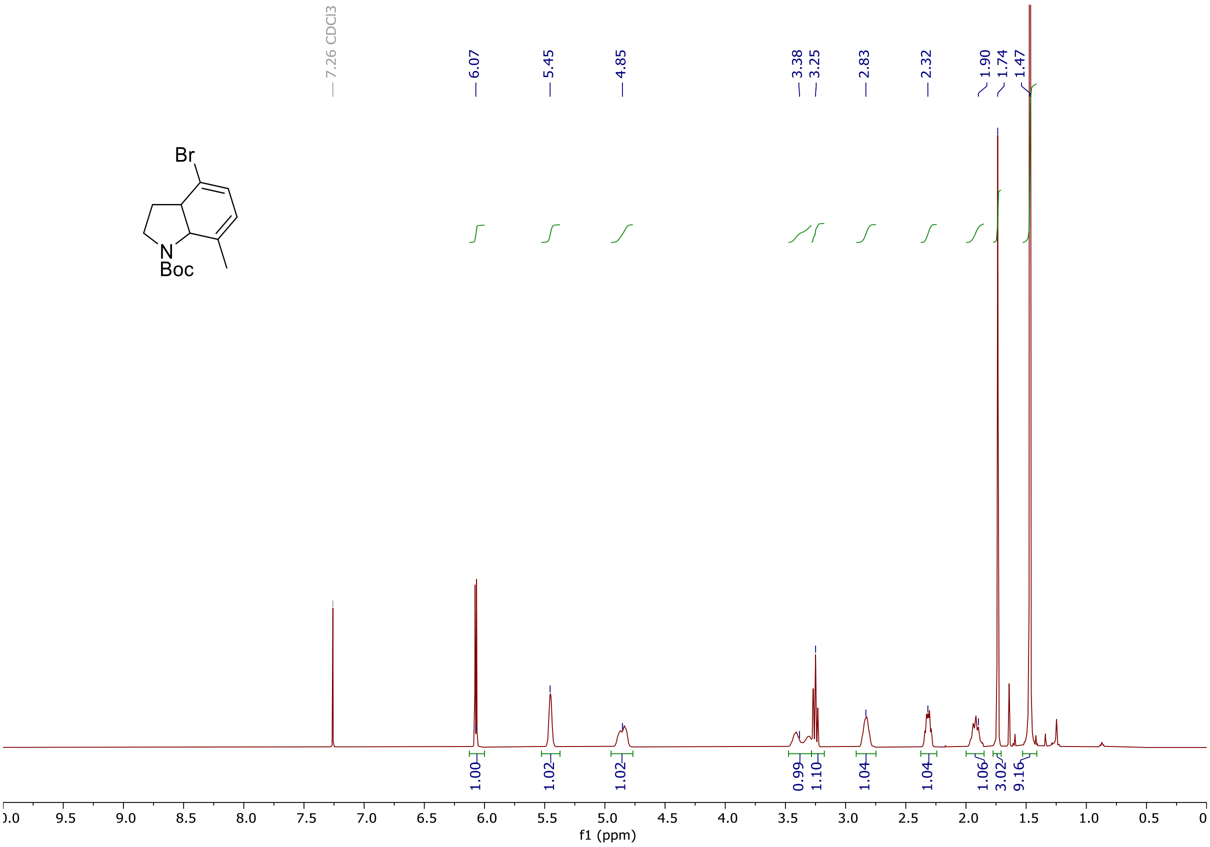

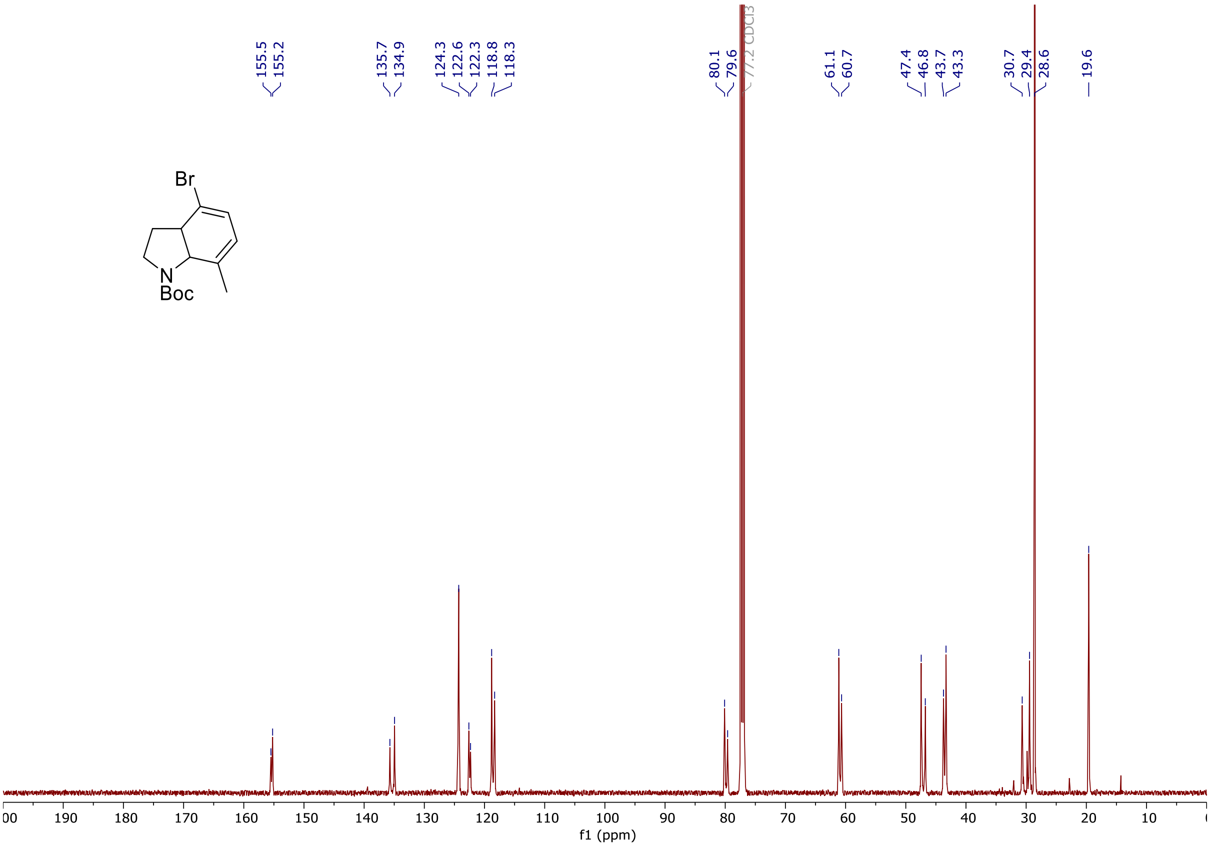

Compound 43b:

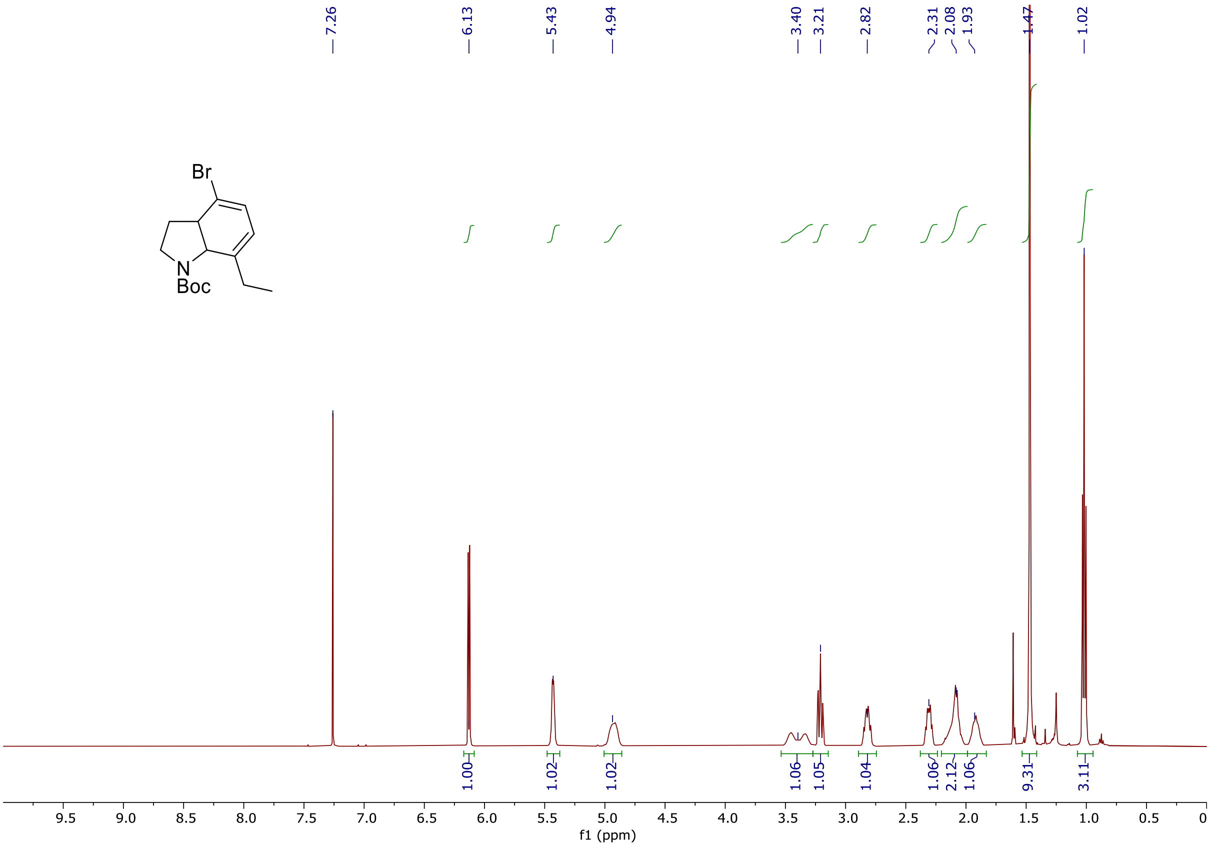

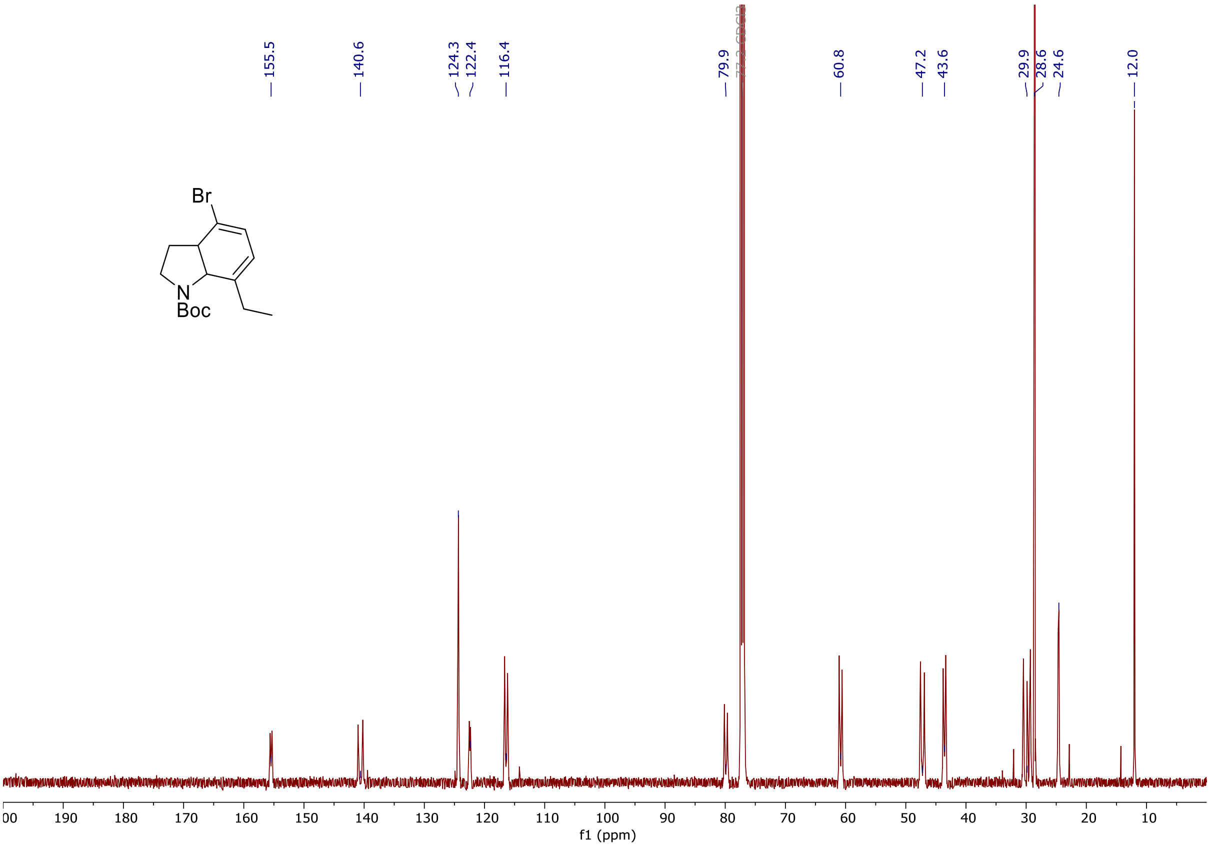

9. References

1) Burchat, A. F.; Chong, J. M.; Nielsen, N. *J. Organomet. Chem.* **1997**, *542*, 281–283.
2) Armarego, W. L. F.; Perrin, D. D. *Purification of Laboratory Chemicals, 4th Ed.*, Reed Educational And Professional Publishing, Oxford, **1996**.
3) Bruker, *SAINT+ v8.38A Integration Engine, Data Reduction Software, Bruker Analytical X-ray Instruments Inc., Madison, WI, USA*, 2015.
4) Bruker, *SADABS 2014/5, Bruker AXS area detector scaling and absorption correction, Bruker Analytical X-ray Instruments Inc., Madison, Wisconsin, USA*, 2014/5.
5) Sheldrick, G. M. *Acta Crystallographica α-Foundation and Advances*, **2015**, *71*, 3–8.
6) Sheldrick, G. M. *Acta Crystallogr., Sect. A: Found. Crystallogr.*, **2008**, *64*, 112–122.
7) Sheldrick, G. M. *Acta Crystallogr. C*, **2015**, *71*, 3–8.
8) Dolomanov, O. V.; Bourhis, L. J.; Gildea, R. J.; Howard, J. A. K.; Puschmann, H. *J. Appl. Crystallogr.*, **2009**, *42*, 339–341.
9) Mukherjee, S.; Lee, D. *Org. Lett.* **2009**, *11*, 2916–2919.
10) Kumar, V. P.; Babu, V. S.; Yahata, K.; Kishi, Y. *Org. Lett.* **2017**, *19*, 2766–2769.
11) Poplata, S.; Bauer, A.; Storch, G.; Bach, T. *Chem.- Eur. J.* **2019**, *25*, 8135–8148.
12) (a) Prévost, S.; Thai, K.; Schützenmeister, N.; Coulthard, G.; Erb, W.; Aggarwal, V. K. *Org. Lett.* **2015**, *17*, 504–507; (b) Pelšs, A.; Gandhamsetty, N.; Smith, J. R.; Mailhol, D.; Silvi, M.; Watson, A. J. A.; Perez-Powell, I.; Prévost, S.; Schützenmeister, N.; Moore, P. R.; Aggarwal, V. K. *Chem.- Eur. J.* **2018**, *24*, 9542–9545.
13) Salituro, L. J.; Pazienza, J. E.; Rychnovsky, S. D. *Org. Lett.* **2022**, *24*, 1190–1194.
14) Umeniya, S.; Sakamoto, D.; Kawauchi, G.; Hayashi, Y. *Org. Lett.* **2017**, *19*, 1112–1115.
15) Sommer, H.; Fürstner, A. *Chem.- Eur. J.* **2017**, *23*, 558–562.
16) Chen, W.; Chen, Y.; Gu, X.; Chen, Z.; Ho, C.-Y. *Nat. Commun*. **2022**, *13*, 5507.
17) Minatti, A.; Buchwald, S. L. *Org. Lett.* **2008**, *10*, 2721–2724.
18) Yang, Y.; Wang, K.; Chen, H.; Feng, Z. *Eur. J. Med. Chem.* **2021**, *211*, 113001–113021.
19) Gaussian 09, Revision C1, Frisch, M. J.; Trucks, G. W.; Schlegel, H. B.; Scuseria, G. E.; Robb, M. A.; Cheeseman, J. R.; Scalmani, G.; Barone, V.; Mennucci, B.; Petersson, G. A.; Nakatsuji, H.; Caricato, M.; Li, X.; Hratchian, H. P.; Izmaylov, A. F.; Bloino, J.; Zheng, G.; Sonnenberg, J. L.; Hada, M.; Ehara, M.; Toyota, K.; Fukuda, R.; Hasegawa, J.; Ishida, M.; Nakajima, T.; Honda, Y.; Kitao, O.; Nakai, H.; Vreven, T.; Montgomery, Jr., J. A.; Peralta, J. E.; Ogliaro, F.; Bearpark, M.; Heyd, J. J.; Brothers, E.; Kudin, K. N.; Staroverov, V. N.; Kobayashi, R.; Normand, J.; Raghavachari, K.; Rendell, A.; Burant, J. C.; Iyengar, S. S.; Tomasi, J.; Cossi, M.; Rega, N.; Millam, J. M.; Klene, M.; Knox, J. E.; Cross, J. B.; Bakken, V.; Adamo, C.; Jaramillo, J.; Gomperts, R.; Stratmann, R. E.; Yazyev, O.; Austin, A. J.; Cammi, R.; Pomelli, C.; Ochterski, J. W.; Martin, R. L.; Morokuma, K.; Zakrzewski, V. G.; Voth, G. A.; Salvador, P.; Dannenberg, J. J.; Dapprich, S.; Daniels, A. D.; Farkas, Ö.; Foresman, J. B.; Ortiz, J. V.; Cioslowski, J.; Fox, D. J. Gaussian, Inc., Wallingford CT, **2009**.
20) Gaussian 16, Revision A.03, Frisch, M. J.; Trucks, G. W.; Schlegel, H. B.; Scuseria, G. E.; Robb, M. A.; Cheeseman, J. R.; Scalmani, G.; Barone, V.; Petersson, G. A.; Nakatsuji, H.; Li, X.; Caricato, M.; Marenich, A. V.; Bloino, J.; Janesko, B. G.; Gomperts, R.; Mennucci, B.; Hratchian, H. P.; Ortiz, J. V.; Izmaylov, A. F.; Sonnenberg, J. L.; Williams-Young, D.; Ding, F.; Lipparini, F.; Egidi, F.; Goings, J.;

Peng, B.; Petrone, A.; Henderson, T.; Ranasinghe, D.; Zakrzewski, V. G.; Gao, J.; Rega, N.; Zheng, G.; Liang, W.; Hada, M.; Ehara, M.; Toyota, K.; Fukuda, R.; Hasegawa, J.; Ishida, M.; Nakajima, T.; Honda, Y.; Kitao, O.; Nakai, H.; Vreven, T.; Throssell, K.; Montgomery, Jr., J. A;. Peralta, J. E.; Ogliaro, F.; Bearpark, M. J.; Heyd, J. J.; Brothers, E. N.; Kudin, K. N.; Staroverov, V. N.; Keith, T. A.; Kobayashi, R.; Normand, J.; Raghavachari, K.; Rendell, A. P.; Burant, J. C.; Iyengar, S. S.; Tomasi, J.; Cossi, M.; Millam, J. M.; Klene, M.; Adamo, C.; Cammi, R.; Ochterski, J. W.; Martin, R. L.; Morokuma, K.; Farkas, O.; Foresman, J. B.; Fox, D. J. Gaussian, Inc., Wallingford CT, 2016.

21) Zhao, Y.; Truhlar, D. G. *Theor. Chem. Acc.* **2008**, *120*, 215–241.

22) Krishnan, R.; Binkley, J. S.; Seeger, R.; Pople, J. A. *J.Chem.Phys*. **1980** 72, 650–654.

23) a) Ess, D.; Houk, K. *J. Am. Chem. Soc*. **2008**, *31*, 10187–10198. b) Gordon, M. S.; *Chem. Phys. Lett*. **1980**, 76, 163–168. c) Roothaan, C. C. J. *Rev. Mod. Phys*., **1951**, *23*, 69–89.

24) (a) Becke, A. D. *J. Chem. Phys*. **1993**, *98*, 1372–1377. (b) Becke, A. D. *J. Chem. Phys*. **1993**, *98*, 5648–5652. (c) Lee, C.; Yang, W.; Parr, R. G. *Phys. Rev. B* **1988**, *37*, 785–789.

25) Sengupta, A.; Raghavachari, K. *Org. Lett*. **2017**, *19*, 2576–2589.

26) Grimme, S.; Antony, J.; Ehrlich, S. Krieg, S. *J. Chem. Phys*. **2010**, *132*, 154104.

27) (a) Becke, A. D.; Johnson, E. R. *J. Chem. Phys.* **2005**, *122*, 154101. b) Johnson, E. R.; Becke, A. D. *J. Chem. Phys.* **2005**, *123*, 024101. c) Johnson, E. R.; Becke, A. D. *J. Chem. Phys.* **2006**, *124*, 174104.

28) (a) Tomasi, J.; Mennucci, B.; Cammi, R. *Chem. Rev*. **2005**, *105*, 2999–3093. (b) Miertuš, S.; Tomasi, J. *Chem. Phys*. **1982**, *65* 239–245. (c) Pascual-Ahuir, J. L.; Silla, E.; Tuñón, I. *J. Comp. Chem*. **1994**, *15*, 1127–1138.

29) http://comp.chem.umn.edu/info/DFT.htm.

30) Wentrup, C. *J. Org. Chem*. **2013**, *78*, 7565–7574.

31) Spartan 14v118 (2013) Wavefunction, Inc., Irvine.

32) Lu, T.; Chen, F. *J. Comput. Chem*. **2012**, *33*, 580–592.

33) Johnson, E. R.; Keinan, S.; Mori-Sánchez, P.; Contreras-García, J.; Cohen, A. J.; Yang, W. *J. Am. Chem. Soc*. **2010**, *132*, 6498–6506

34) Humphrey, W.; Dalke, A.; Schulten, K. "VMD - Visual Molecular Dynamics", *J. Molec. Graphics*, **1996**, *14*, 33–38.
